# C–H Functionalization-Enabled 11-Step Semisynthesis of (−)-Veragranine A and Characterization of Synthetic Analogs in Osteoarthritis-related Pain Treatment

**DOI:** 10.1021/jacs.4c04025

**Published:** 2024-06-06

**Authors:** Donghui Ma, Paz Duran, Reem Al-Ahmad, Sara Hestehave, Margarita Joa, Omar Alsbiei, Erick J. Rodríguez-Palma, Yanrong Li, Shilin Wang, Rajesh Khanna, Mingji Dai

**Affiliations:** † Department of Chemistry, Emory University, Atlanta, Georgia 30322, United States; ‡ Department of Molecular Pathobiology, College of Dentistry, New York University, New York, New York 10010, United States; § Department of Chemistry, Purdue University, West Lafayette, Indiana 47906, United States; ∥ Department of Pharmacology and Therapeutics, College of Medicine, University of Florida, Gainesville, Florida 32610, United States; ⊥ Department of Pharmacology and Chemical Biology, School of Medicine, Emory University, Atlanta, Georgia 30322, United States

## Abstract

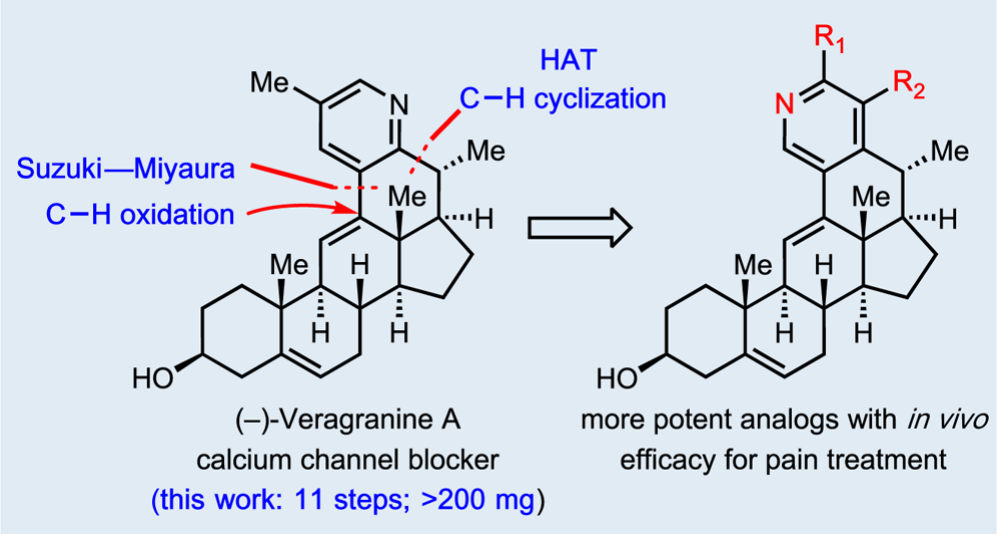

We report an efficient semisynthesis of the cholestane steroidal alkaloid (−)-veragranine A with a 6/6/6/5/6/6 hexacyclic ring system, eight stereocenters, and a unique C12–C23 linkage. Our synthesis features a Schönecker–Baran C–H oxidation at C12, a Suzuki–Miyaura cross-coupling to form the C12–C23 bond, and a hydrogen atom transfer (HAT)-initiated Minisci C–H cyclization to forge the C20–C22 bond with desired stereochemistry at C20. These enabling transformations significantly enhanced the overall synthetic efficiency and delivered (−)-veragranine A in 11 steps and over 200 mg from cheap and readily available dehydroepiandrosterone. In addition, this approach allowed flexible syntheses of novel synthetic analogs for biological evaluations in sensory neurons *in vitro* and in an *in vivo* model of arthritic pain, from which two novel lead compounds were identified for further development.

## Introduction

**Figure 1:**
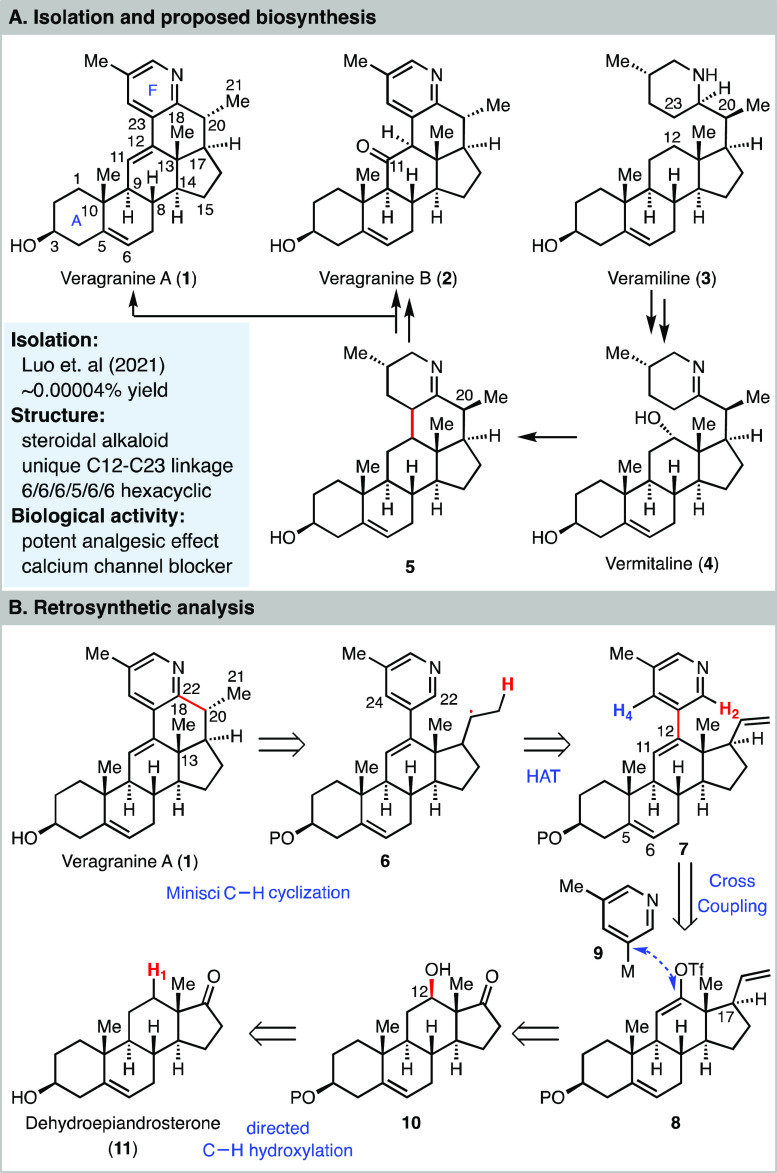
Veragranines, plausible biosynthesis, and synthetic plan.

Chronic pain affects 20–30% of the global population.^1^ Traditional remedies from plants, such as opium poppy-derived morphine, willow bark-based aspirin, and cannabis-origin tetrahydrocannabinol (THC), have historically treated various types of pain.^2^ Despite opioids being central to pain management, they have limitations and serious side effects,^3^ prompting the search for alternative antinociceptive therapies. Extensive pain research has elucidated crucial neuronal mechanisms that underlie clinically significant pain conditions.^4^ Both the peripheral and the central nociceptive systems play substantial roles in pain generation during inflammation and nerve injuries. Peripheral nociceptors, specialized pain cells, undergo sensitization in response to inflammation, and peripheral nerve fibers exhibit abnormal discharges following injury or disease. Chronic pain resulting from a nervous system injury or disease, known as neuropathic pain, is associated with persistent electrical hyperactivity in nociceptors specialized for detecting damaging stimuli and inflammation. Ion channels, specifically voltage-gated sodium and calcium channels, play crucial roles in transmitting noxious stimuli. Sustained stimuli or chronic diseases can alter this process, leading to hyperactivity in the damaged nerves. Discovering molecules that block the activities of key ion channels that regulate the hyperexcitability of neurons is important for developing antinociceptive agents.

(−)-Veragranines A (**1**, Figure 1A) and B (**2**) are two cholestane steroidal alkaloids isolated by Luo and co-workers from *Veratrum grandiflorum*, an important ingredient of traditional herbal medicine often used for pain and inflammation treatment.^5^ Biologically, veragranines A and B were reported to block the N-type calcium channels (Ca_v_2.2) with IC_50_ values of 45.76 ± 1.14 and 7.82 ± 1.10 μM, respectively, and have analgesic effect at 1.0 and 0.5 mg/kg in male mice models with acetic acid-induced writhing pain. Thus, both veragranines A and B are promising lead compounds deserving further development for pain treatment. However, the isolation yields of (−)-veragranines A and B from *V. grandiflorum* are extremely low, about 0.00004%, limiting their comprehensive biological evaluations. Thus, efficient and flexible chemical syntheses of the veragranines and their analogs are important and necessary.

Structurally, veragranines A and B feature a complex 6/6/6/5/6/6 hexacyclic ring system, a unique C12–C23 linkage, and a trisubstituted pyridine ring. Veragranine A and veragranine B differ at the C11 and C12 oxidation states. All of these structural features render veragranines A and B challenging targets. So far, no complete chemical syntheses of veragranines A and B have been reported. In 2022, Shi and co-workers reported efficient syntheses of 5α,6-dihydroveragranines A and B from hecogenin acetate.^6^ Herein, we report a scalable semisynthesis of (−)-veragranine A from dehydroepiandrosterone. Our approach also enabled access to new synthetic analogs for biological evaluations in sensory neurons *in vitro* and in an *in vivo* model of arthritic pain, from which two novel lead compounds were identified for further development.

Biosynthetically, veragranines A and B could be derived from veramiline (**3**, Figure 1A), a major steroidal alkaloid of *V. grandiflorum*. Veramiline could be converted to vermitaline (**4**) via an enzymatic C–H hydroxylation at C12 and dehydrogenation of the piperidine ring. A subsequent C12–C23 bond formation would give intermediate **5**, which could be further elaborated to veragranines A and B via a series of oxidations and epimerization of the C20 stereochemistry.

Retrosynthetically, we first disconnected the C20–C22 bond and envisioned a Minisci-type^7^ radical C–H cyclization to close the E ring (cf. **6** → **1**, Figure 1B). Radical intermediate **6** could be generated from **7** via a HAT process.^8^ While such HAT-initiated Minisci C–H cyclization would be efficient to construct the C20–C22 bond, we were aware of a few challenges. First, in addition to the terminal olefin, there are two trisubstituted olefins in the presence, which can complicate the HAT process. Over-reduction of these double bonds must be avoided. Second, the radical cyclization could happen at both the *ortho* (C22) and the *para* (C24) positions of the pyridine ring, thus regioselectivity needs to be controlled. Third, a new stereocenter at C20 would be generated during the Minisci radical cyclization process. We hypothesized that during the cyclization, the C21 methyl group would prefer to be in the pseudo-equatorial position to avoid strong 1,3-diaxial interaction with the C18 methyl group at C13, thus giving the desired stereochemistry at C20. Fourth, the HAT process would generate a secondary radical, which is less stable and less nucleophilic than the tertiary radical involved in most of the HAT reactions, and such a secondary radical is prone to early reduction to an alkane before cyclizing with pyridine. Despite these challenges, this HAT-initiated C–H cyclization strategy was expected to improve synthetic efficiency and flexibility and is thus worth developing. Compound **7** could be synthesized from vinyltriflate **8** via a transition-metal-catalyzed cross-coupling reaction^9^ with a pyridyl nucleophile (cf. **9**). Compound **8** could be synthesized from **10** via oxidation adjustment at C12 and the introduction of a vinyl group at C17. To access **10**, we envisioned a Schönecker–Baran C–H oxidation^10^ at the C12 of a protected form of dehydroepiandrosterone (**11**, **∼$**2/g), a cheap and readily available starting material.

## Results and Discussion

### Chemical Synthesis

**Scheme 1:**
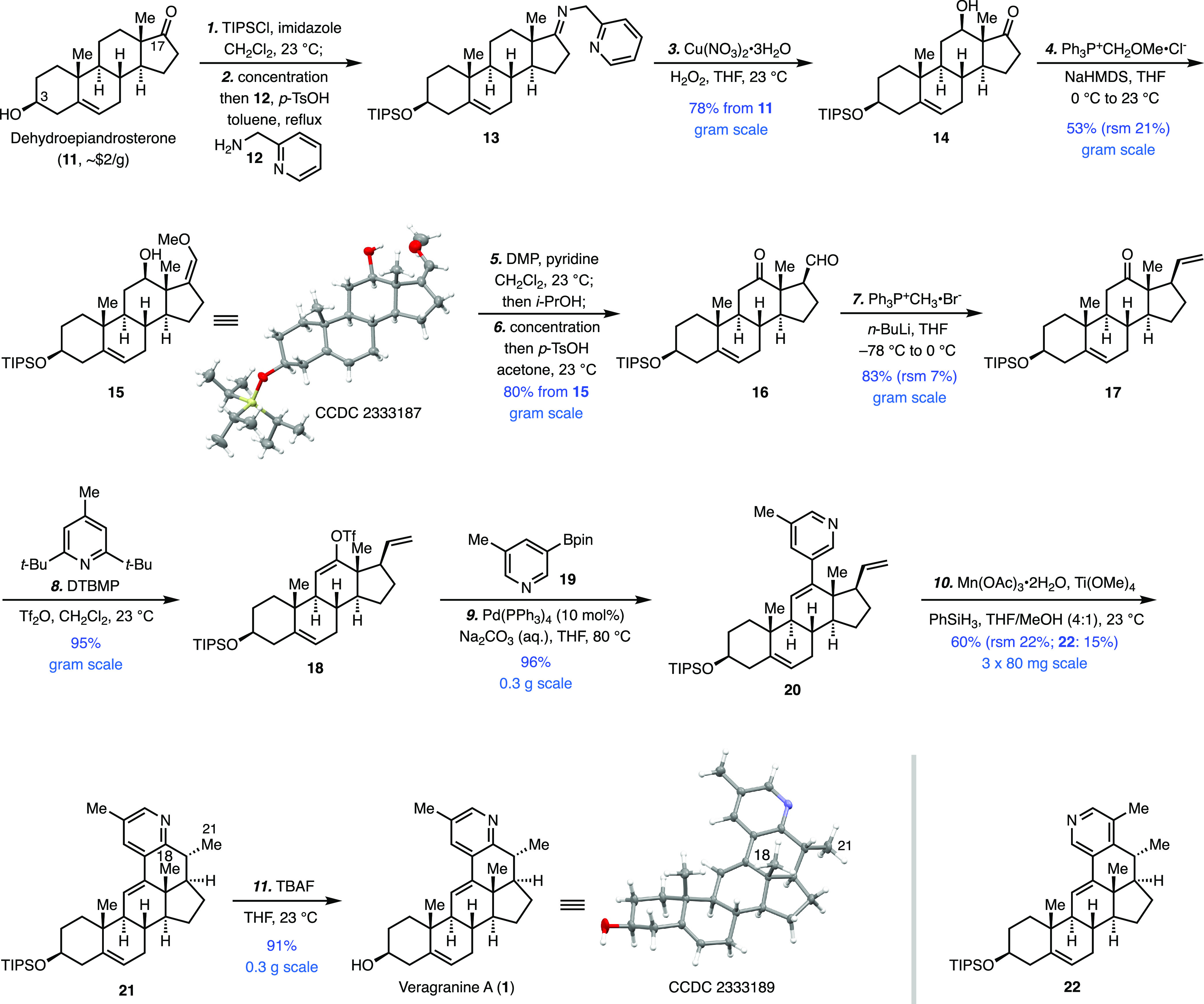
11-Step Synthesis of (−)-Veragranine A

Our synthesis started from dehydroepiandrosterone (**11**, Scheme 1). Its C3 alcohol was first protected as a TIPS ether. In the same reaction pot, a subsequent condensation of the C17 ketone with 2-picolylamine (**12**) gave imine **13** for the next Schönecker–Baran C–H oxidation with a combination of Cu(NO_3_)_2_ and H_2_O_2_. Product **14** was obtained in 78% yield from **11** on a gram scale. To introduce a vinyl group at C17, we employed two Wittig one-carbon homologations. First, Wittig olefination of **14** with the phosphonium ylide derived from treating (methoxymethyl)triphenylphosphonium chloride with NaHMDS gave methyl enol ether **15** in 53% yield on a multigram scale with 21% recycled **14**. Owing to the steric hindrance of the C17 ketone and the free alcohol at C12, it was difficult to push the reaction to a full conversion without decreasing the overall yield. Additionally, the structure of **15** was unambiguously established by X-ray crystallographic analysis (CCDC 2333187). Compound **15** was then converted to ketoaldehyde **16** in 80% yield on a gram scale via a one-pot DMP oxidation of the C12 alcohol followed by methyl enol ether hydrolysis with *p*-toluenesulfonic acid (*p*-TsOH). The second chemoselective Wittig olefination occurred on the aldehyde to convert **16** to **17** in 83% yield on a gram scale. Compound **17** was then advanced to vinyltriflate **18** in 95% yield on a gram scale with triflic anhydride (Tf_2_O) in the presence of 2,6-di-*tert*-butyl-4-methylpyridine (DTBMP). For the subsequent transition-metal-catalyzed cross-coupling reaction to introduce the pyridine functionality, we first explored the Suzuki–Miyaura cross-coupling with commercially available boronate **19**. To our delight, the cross-coupling product **20** could be produced in 96% yield with Pd(PPh_3_)_4_ as a catalyst and aqueous Na_2_CO_3_ as a base in THF at an elevated temperature (80 °C).

With compound **20** in hand, we next focused on the HAT-initiated Minisci C–H cyclization. Starting from the previously reported conditions^11−13^ and after comprehensive optimizations (see the Supporting Information), we identified a combination of Mn(OAc)_3_, PhSiH_3_, and Ti(OMe)_4_ in mixed THF/MeOH as the optimal conditions to deliver the desired product **21** in 60% yield together with its isomer **22** in 15% yield on 3 × 80 mg scale. 22% yield of **20** was recovered as well. Notably, the use of titanium tetra-alkoxide such as Ti(OMe)_4_ as a Lewis acid to activate the pyridine is important for the cyclization. The desired stereochemistry at C20 was obtained, consistent with a cyclization transition state that minimizes 1,3-diaxial interactions between C21 and the axial C18 methyl groups. To complete the synthesis, the TIPS group was removed with TBAF to give (−)-veragranine A in 91% yield. Its structure was unambiguously confirmed by an X-ray crystallographic analysis (CCDC 2333189). Overall, (−)-veragranine A was prepared in 11 steps and over a 200 mg scale.

In addition, we wondered whether the HAT-initiated Minisci C–H cyclization is general and can be used to prepare veragranine analogs for biological evaluations. Substrates **24b**–**h** were prepared in 71–97% yields using the Suzuki–Miyaura cross-coupling (Table 1). Notably, a mixture of inseparable rotamers was obtained for the cases of **24b** (2/1), **24d** (3/1), and **24h** (3/1) as well as the corresponding TIPS removal products **26b** (2/1), **26d** (3/1), and **26h** (3/1). While **24b**–**h** all underwent the HAT-initiated Minisci C–H cyclization, different results were obtained. For **24b** and **24d**, since the *para* position is blocked by a methyl group, only **25b** and **25d** were obtained in 60% and 49% yields, respectively, with about 40% of the starting material recycled. For **24c** and **24e** with a methyl or ester group at the *ortho* position, a 1.7/1 or 1/2.2 mixture of **25ca** and **25cb** or **25ea** and **25eb** was obtained, albeit in 19 or 32% yield, respectively. The low yields for these two cases were presumably due to the steric hindrance generated by the methyl or ester group, which could negatively influence coordination of the pyridine nitrogen with Ti(OMe)_4_. For **24f** with an ester at the *meta* position, only regioisomer **25f** was obtained in 50% yield with 34% of starting material recycled. In addition, both quinoline (**24g**)- and isoquinoline (**24h**)-containing substrates are effective for the HAT-initiated Minisci C–H cyclization. Notably, for **24g**, the *para* cyclization product (**25gb**) turned out to be major and a 1/7.4 mixture of **25ga** and **25gb** was obtained in 59% yield. For **24h**, product **25h** was obtained in 30% yield with a 34% yield of starting material recycled. For biological evaluations, the TIPS group of the Suzuki–Miyaura cross-coupling products and the HAT cyclization products were removed with TBAF.

**Table 1:**
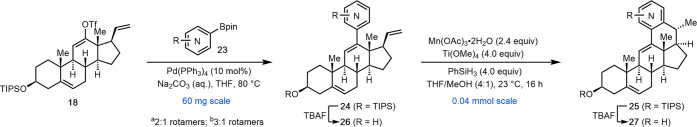

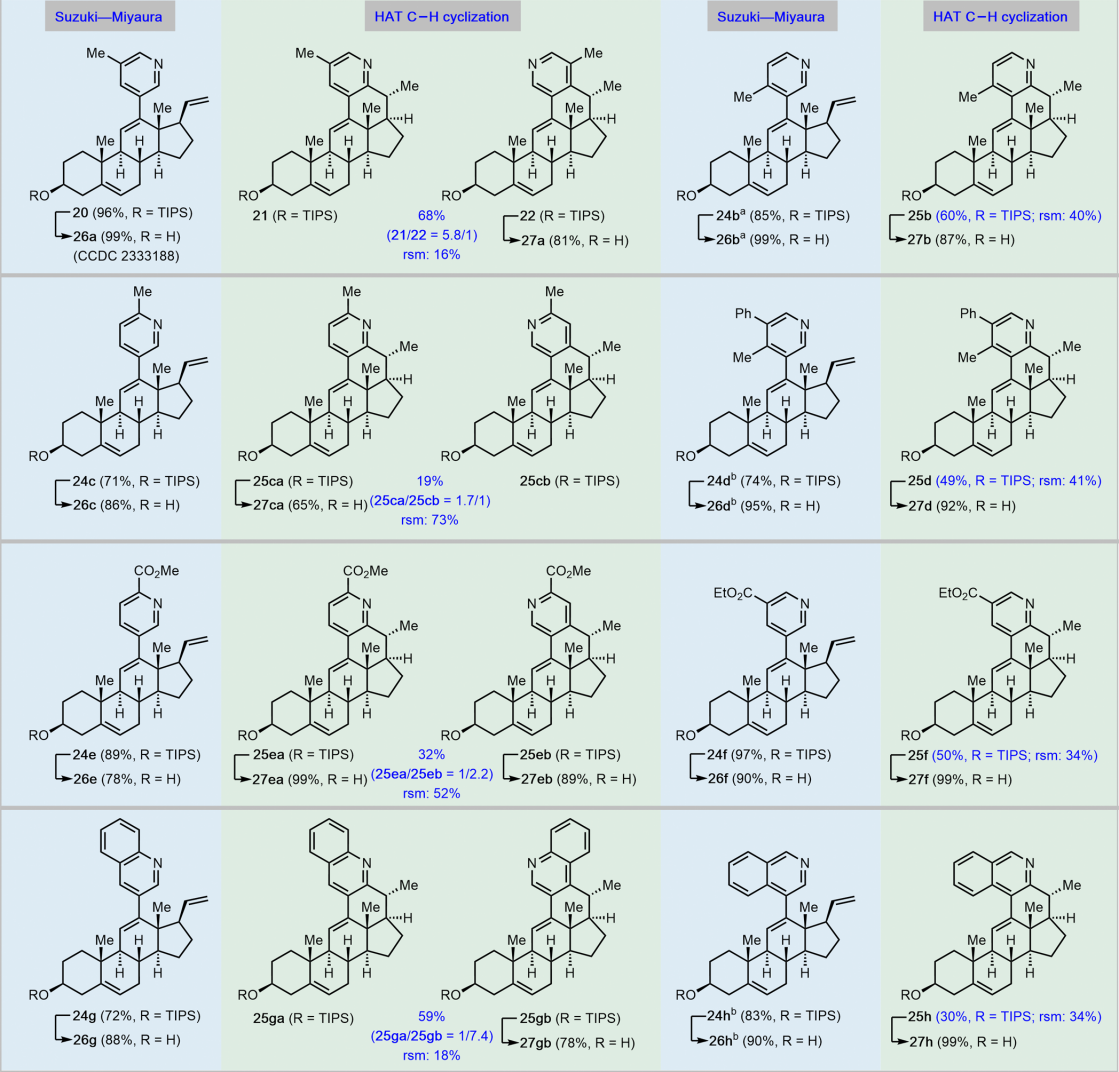
Substrate Scope and Analog Synthesis

### Biological Evaluations

**Figure 2:**
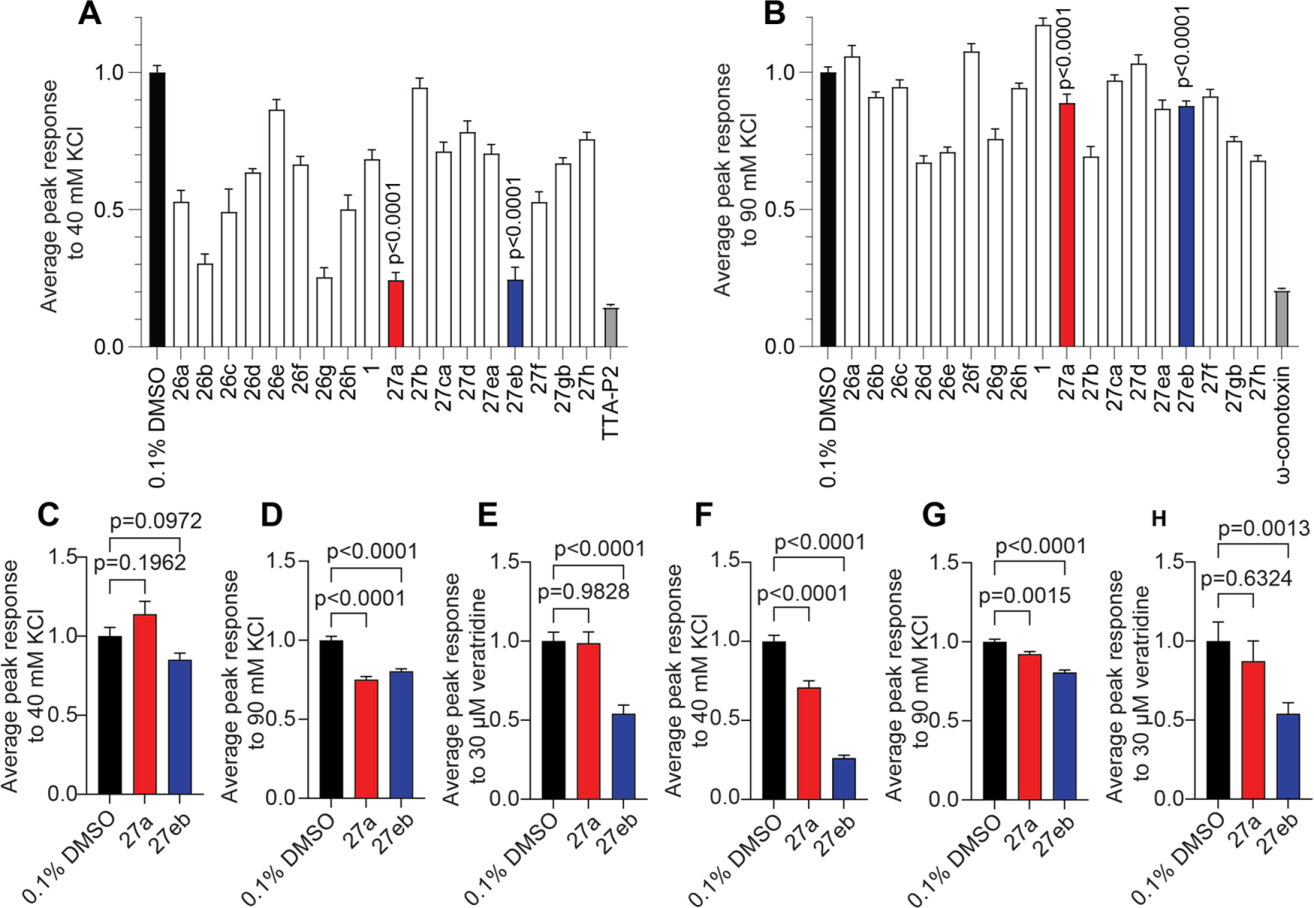
Effects of veragranine A analogs on depolarization-induced calcium influx in rat DRG neurons. Bar graphs of normalized average peak response of rat DRG neurons incubated overnight with 20 μM veragranine A analogs or 0.1% DMSO as a control in response to 40 mM KCl (A) and 90 mM KCl (B) trigger. TTA-P2 (1 μM) and ω-conotoxin GVIA (500 nM) were used as positive controls, respectively. Bar graphs of normalized average peak response of rat DRG neurons incubated overnight with 10 μM **27a** and **27eb** or 0.1% DMSO as a control in response to 40 mM KCl (C), 90 mM KCl (D), and 30 μM veratridine (E) trigger. Bar graphs of normalized average peak response of rat DRG neurons incubated 30 min with 10 μM **27a** and **27eb** or 0.1% DMSO as a control in response to 40 mM KCl (F), 90 mM KCl (G), and 30 μM veratridine (H) trigger. All data are mean ± SEM; one-way ANOVA with Dunnett’s post hoc test.

To evaluate the functional effects of veragranine A and its analogs on voltage-gated ion channels, we used calcium imaging to measure changes in depolarization-evoked Ca^2+^ influx.^14^ Primary cultures of rat dorsal root ganglia (DRG) neurons were seeded on coverslips and treated overnight with 20 μM corresponding compounds or 0.1% DMSO as a vehicle (negative) control. The next day, DRG neurons were loaded with Fura-2-acetoxymethyl ester (Fura2-AM) for 30 min before being mounted in a perfusion chamber for the imaging assay. To test the inhibitory effects of the compounds on voltage-gated calcium channels, which are critically important for pain sensation, we used KCl as a trigger. Peak calcium influx was recorded within 15 s of stimulation with two concentrations of KCl; 40 mM KCl trigger to test low-voltage-activated (LVA) and 90 mM KCl trigger to test high-voltage-activated (HVA) calcium channels. We found that, when compared with 0.1% DMSO, veragranine A at 20 μM inhibited calcium influx by about 30%. To our delight, its analogs **26b**, **26c**, **26g**, **27a**, and **27eb** inhibited calcium influx by more than 50% when challenged with 40 mM KCl (Figure 2A). Three of these analogs, **26g**, **27a**, and **27eb**, also exhibited inhibition of ∼12–25% when challenged with 90 mM KCl (Figure 2B). At this stage, we selected **27a** and **27eb** for further investigation. Overnight incubation with 10 μM **27a** and **27eb** did not inhibit calcium influx via LVA channels (Figure 2C). However, both analogs produced a significant decrease in KCl-triggered calcium channel activity when neurons were challenged with 90 mM KCl (Figure 2D). To evaluate the effect of overnight incubation with 10 μM **27a** and **27eb** on voltage-gated sodium channels, we used a general sodium channel activator, veratridine (30 μM), as a depolarizing agent.^15^ We found that **27a** had no significant effect on calcium influx, whereas **27eb** inhibited veratridine-evoked calcium influx compared with the control condition (0.1% DMSO) (Figure 2E).

Next, we examined the effects of acute application (30 min) of **27a** and **27eb** on the calcium influx. When compared with control conditions (0.1% DMSO), both analogs significantly suppressed the average peak response when neurons were challenged with 40 and 90 mM KCl (Figure 2F,G). However, only analog **27eb** significantly reduced veratridine-evoked calcium influx (Figure 2H). Together, these data suggest that these veragranine A analogs inhibit calcium influx in rat DRG sensory neurons: **27a** by blocking voltage-gated calcium channels, and **27eb** by blocking voltage-gated calcium and sodium channels.

**Figure 3:**
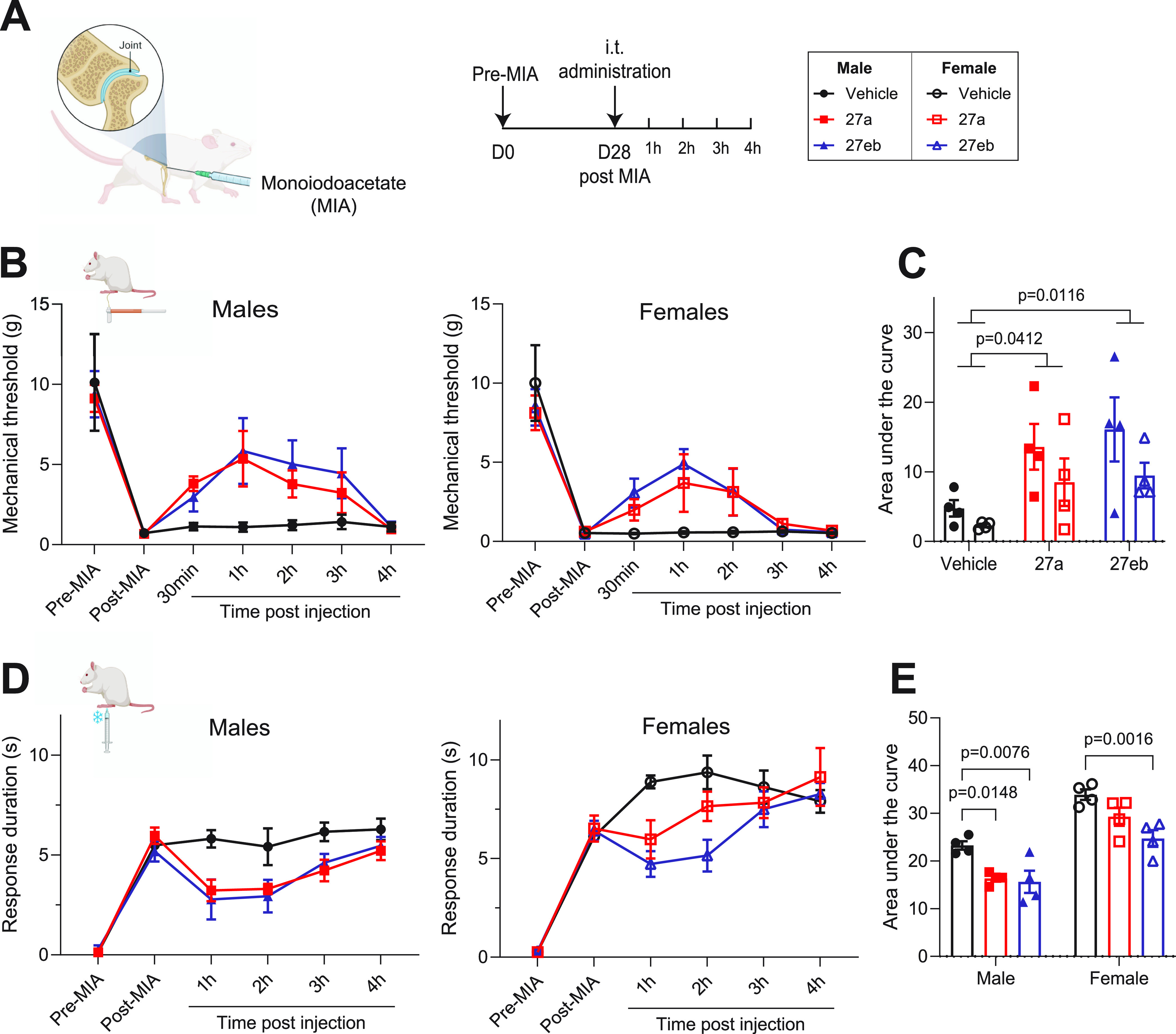
Intrathecal administration of veragranine A analogs **27a** and **27eb** reverse mechanical and cold allodynia induced by the monoiodoacetate (MIA) model of osteoarthritis pain. (A) Study-design schematic and treatment conditions. (B) Baseline paw-withdrawal threshold was measured before (pre-MIA) and after (post-MIA) in both male and female rats. The response paw-withdrawal threshold was then assessed every hour following a single administration of vehicle, **27a** or **27eb** (20 μg/5 μL, i.t.) up to 4 h after administration. (C) Quantification of area under the curve of the paw-withdrawal threshold reported in panel B from post-MIA to 4 h post administration. **27a** and **27eb** significantly improved MIA-induced mechanical allodynia, when compared with vehicle across sex. (D) Baseline response duration to an application of an acetone drop was measured before (pre-MIA) and after (post-MIA) in male and female rats. The response duration was then assessed every hour following a single administration of vehicle, **27a** or **27eb** (20 μg/5 μL, i.t.) up to 4 h after administration. (E) Quantification of area under the curve of the response duration reported in panel D from post-MIA to 4 h post administration. For panels C and E, results were compared using 2-way ANOVA and postcomparison using Tukey’s multiple comparison test. Postcomparison was made to the vehicle-treated groups across sex (C) or the sex-specific vehicle group when significant sex-specific effects were detected on the ANOVA (E). For all panels, *n* = 4 rats per sex/group. Results are displayed as mean ± SEM. Schematics in panel A were generated using Biorender.com.

To assess the potential effects of these compounds on pain related to osteoarthritis, we tested the antinociceptive effects of **27a** and **27eb** on mechanical and cold allodynia in the monoiodoacetate (MIA) model (Figure 3).^16^ MIA was induced via injection into the left knee joint in male and female rats, and 4 weeks after injury, they were injected with **27a** and **27eb** (20 μg/5 μL, i.t.), or vehicle, and assessed hourly over a 4 h period. MIA induced a marked increase in mechanical sensitivity on the plantar surface of the paw in both male and female rats (post-MIA time point in Figure 3B), which was significantly improved by administration of **27a** and **27eb** across males and females (Figure 3C). Statistical analysis showed no significant effects of sex on the results, and across sex at peak effect the %MPE was found to be 48.5 ± 18.6% (mean ± SEM) and 55.0 ± 13.0% for **27a** and **27eb**, respectively, compared with 83.03 ± 20.4% for Gabapentin in this model (see the Supporting Information). In addition, MIA induced clear cold allodynia in both males and females, which was seen as an increase in the duration of aversive response following the application of a drop of acetone to the plantar surface of the ipsilateral paw (post-MIA time point, Figure 3D). Administration of **27eb** significantly improved the cold allodynia in both genders (%MPE: 48.7 ± 7.8% and 56.1 ± 19.8% for females and males, respectively), while **27a** showed robust effects in males (45.4 ± 9.5%), but not significantly in females (33.7 ± 11.1%) (Figure 3E). This compares with 72.8 ± 8.2% for Gabapentin across sex (see the Supporting Information). In summary, these results suggest the overall beneficial effects of the two compounds in improving pain-like behavioral symptoms from an osteoarthritis model.

## Conclusions

In summary, chemical synthesis and biological evaluation of (−)-veragranine A and its analogs were reported. Starting from cheap and readily available starting material dehydroepiandrosterone, (−)-veragranine A was synthesized in 11 steps and over 200 mg. The key steps include Schönecker–Baran C–H oxidation at C12, Suzuki–Miyaura cross-coupling to form the C12–C23 bond, and stereoselective HAT-initiated Minisci C–H cyclization to forge the C20–C22 bond. We further evaluated the substrate scope of the HAT-initiated Minisci C–H cyclization and prepared a small collection of veragranine A analogs. Our biological evaluation identified several analogs including **27a** and **27eb** as novel calcium and sodium channel blockers with *in vivo* efficacy. Further analog synthesis and biological evaluations are ongoing and will be reported in due course.

## Supporting Information Available

Experimental procedures and NMR spectra for all new compounds (PDF)

## Abbreviations

HAT: hydrogen atom transfer
TIPS: triisopropylsilyl
NaHMDS: sodium bis(trimethylsilyl)amide
DMP: Dess–Martin periodinane
*p*-TsOH: *p*-toluenesulfonic acid
Tf_2_O: triflic anhydride
DTBMP: 2,6-di-*tert*-butyl-4-methylpyridine
TBAF: tetra-*n*-butylammonium fluoride
Cav2.2: N-type voltage-gated calcium channel
DRG: dorsal root ganglia
MIA: monoiodoacetate
PWT: paw-withdrawal threshold
vF: von Frey
VGCC: voltage-gated calcium channels
