# Supplementary material for: C–H Functionalization-Enabled 11-Step Semisynthesis of (−)-Veragranine A and Characterization of Synthetic Analogs in Osteoarthritis-related Pain Treatment
Source: J Am Chem Soc. 2024 Jun 6;146(24):16698–705. Online ahead of print. doi: 10.1021/jacs.4c04025 (PMC11191690; doi:10.1021/jacs.4c04025)
Supplement: Supplementary file 1 — ja4c04025_si_001.pdf [file ja4c04025_si_001.pdf]

Supporting Information

C–H Functionalization-Enabled 11-Step Semi-synthesis of (−)-Veragranine A and Characterization of Synthetic Analogs in Osteoarthritis-related Pain Treatment

Donghui Ma,[1] Paz Duran,[2] Reem Al-Ahmad,[1] Sara Hestehave,[2] Margarita Joa,[2] Omar Alsbiei,[2] Erick J. Rodríguez-Palma,[2] Yanrong Li,[3] Shilin Wang,[1] Rajesh Khanna,[4,*] and Mingji Dai[1,5,*]

[1]Department of Chemistry, Emory University, Atlanta, GA 30322, USA; [2]Department of Molecular Pathobiology, College of Dentistry, New York University, New York, New York 10010, USA; [3]Department of Chemistry, Purdue University, West Lafayette, IN 47906; [4]Department of Pharmacology and Therapeutics, College of Medicine, University of Florida, Gainesville, FL 32610, USA; [5]Department of Pharmacology and Chemical Biology, School of Medicine, Emory University, Atlanta, GA 30322, USA.

Table of Contents

1. Supplementary Table S1 and Figure S1..3

2. General Information 6

3. Synthesis Experimental Procedures and Spectra Data………...6

4. Natural Product NMR Data Comparison…………………...……………………………….....27

5. X-ray Structure and Analysis Data...29

6. Methods and Results for Biological Evaluations...32

7. References..34

8. ^{1}H, and ^{13}C NMR Spectra...35

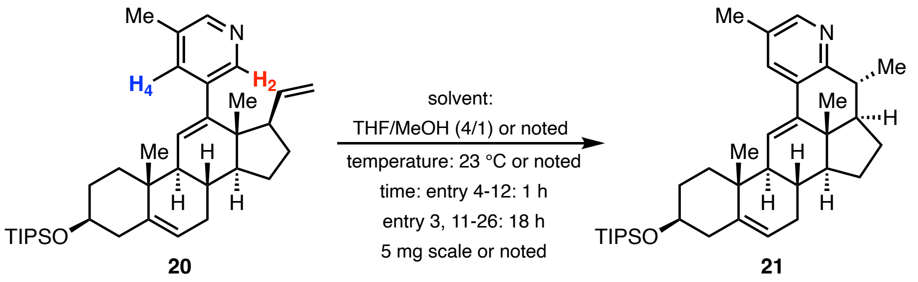

entry	reaction conditions (equiv.)	results
1	$Fe(acac)_3$ (1.0), TFA (1.0), $PhSiH_3$ (2.5), *i*-PrOH, 60 °C, 20 h	**21** (trace); **20** (74%)
2	$Fe(NO_3)_3 \cdot 9H_2O$ (6.0), $NaBH_4$ (2.0), CH_3CN/EtOH (1/1), 0 °C, 30 min	**21** (trace) & over reduction
3	$Co(acac)_3$ (1.0), TBHP (1.0), Et_3SiH (5.0), CH_2Cl_2	**21** & **22** (trace); **20** (18%)
4	$Fe(acac)_3$ (1.0), $BF_3 \cdot OEt_2$ (4.0), $PhSiH_3$ (4.0), 60 °C	**21** (~10%); **20** (52%)
5	$Co(acac)_3$ (1.0), $BF_3 \cdot OEt_2$ (4.0), $PhSiH_3$ (4.0), 60 °C	**21** (~2%); **20** (88%)
6	$Mn(acac)_3$ (1.0), $BF_3 \cdot OEt_2$ (4.0), $PhSiH_3$ (4.0), 60 °C	**21** (20%); **20** (76%)
7	MnF_3 (1.0), $BF_3 \cdot OEt_2$ (4.0), $PhSiH_3$ (4.0), 60 °C	**21** (24%); **22** (6%); **20** (56%)
8	$Mn(dpm)_3$ (1.0), $BF_3 \cdot OEt_2$ (4.0), $PhSiH_3$ (4.0), 60 °C	**21** (22%); **22** (6%); **20** (46%)
9	$MnPO_4 \cdot H_2O$ (1.0), $BF_3 \cdot OEt_2$ (4.0), $PhSiH_3$ (4.0), 60 °C	**21** (2%); **20** (78%)
10	$Mn(OAc)_3 \cdot H_2O$ (1.0), $BF_3 \cdot OEt_2$ (4.0), $PhSiH_3$ (4.0), 60 °C	**21** (36%); **22** (4%); **20** (44%)
11	$Mn(OAc)_3 \cdot H_2O$ (1.0), $BF_3 \cdot OEt_2$ (4.0), $PhSiH_3$ (4.0)	**21** (38%); **22** (4%); **20** (58%)
12	$Mn(OAc)_3 \cdot H_2O$ (1.0), $BF_3 \cdot OEt_2$ (4.0), $PhSiH_3$ (4.0), 0 °C	**21** (36%); **22** (6%); **20** (58%)
13	$Mn(OAc)_3 \cdot H_2O$ (1.0), $BF_3 \cdot OEt_2$ (4.0), Ph_2SiH_2 (4.0)	**21** (8%); **22** (2%); **20** (86%)
14	$Mn(OAc)_3 \cdot H_2O$ (1.0), $BF_3 \cdot OEt_2$ (4.0), $PhMeSiH_2$ (4.0)	**21** (32%); **22** (6%); **20** (60%)
15	$Mn(OAc)_3 \cdot H_2O$ (1.0), $BF_3 \cdot OEt_2$ (4.0), *n*-$BuSiH_3$ (4.0)	**21** (36%); **22** (6%); **20** (58%)
16	$Mn(OAc)_3 \cdot H_2O$ (1.0), $BF_3 \cdot OEt_2$ (4.0), Et_2SiH_2 (4.0)	**21** (28%); **22** (4%); **20** (68%)
17	$Mn(OAc)_3 \cdot H_2O$ (1.0), $BF_3 \cdot OEt_2$ (4.0), (*i*-PrO)$PhSiH_2$ (4.0)	**21** (18%); **22** (4%); **20** (78%)
18	$Mn(OAc)_3 \cdot H_2O$ (1.0), BCl_3 (4.0), $PhSiH_3$ (4.0)	**21** & **22** (trace); **20** (16%)
19	$Mn(OAc)_3 \cdot H_2O$ (1.0), TMSOTf (4.0), $PhSiH_3$ (4.0)	**20** (10%) and decomposition
20	$Mn(OAc)_3 \cdot H_2O$ (1.0), TFA (4.0), $PhSiH_3$ (4.0)	**21** (7%); **22** (7%); **20** (85%)
21	$Mn(OAc)_3 \cdot H_2O$ (1.0), $B(OMe)_3$ (4.0), $PhSiH_3$ (4.0)	**21** (36%); **22** (14%); **20** (48%)
22	$Mn(OAc)_3 \cdot H_2O$ (1.0), BiF_3 (4.0), $PhSiH_3$ (4.0)	**21** (36%); **22** (8%); **20** (52%)
23	$Mn(OAc)_3 \cdot H_2O$ (1.0), $Ti(OMe)_4$ (4.0), $PhSiH_3$ (4.0)	**21** (56%); **22** (12%)
24	$Mn(OAc)_3 \cdot H_2O$ (1.0), Ti(O*i*-Pr)$_4$ (4.0), $PhSiH_3$ (4.0)	**21** (52%); **22** (8%)
25	$Mn(OAc)_3 \cdot H_2O$ (1.0), $Ti(OEt)_4$ (4.0), $PhSiH_3$ (4.0)	**21** (52%); **22** (14%)
26	$Mn(OAc)_3 \cdot H_2O$ (1.0), $TiCl_4$ (4.0), $PhSiH_3$ (4.0)	**21** (12%); **22** (2%); **20** (50%)[a]
27	$Mn(OAc)_3 \cdot H_2O$ (1.0), $Ti(OMe)_4$ (4.0), $PhSiH_3$ (4.0), 8 h	**21** (64%); **22** (16%)
28	$Mn(OAc)_3 \cdot H_2O$ (1.0), $Ti(OMe)_4$ (4.0), $PhSiH_3$ (4.0), 12 h	**21** (60%); **22** (12%)
29	$Mn(OAc)_3 \cdot H_2O$ (1.0), $Ti(OMe)_4$ (4.0), $PhSiH_3$ (4.0), 24 h	**21** (40%); **22** (10%)
30	$Mn(OAc)_3 \cdot H_2O$ (1.0), $Ti(OMe)_4$ (4.0), $PhSiH_3$ (4.0), 8 h	**21** (37%); **22** (8%); **20** (50%)[b]
31	$Mn(OAc)_3 \cdot H_2O$ (2.4), $Ti(OMe)_4$ (4.0), $PhSiH_3$ (4.0), 16 h	**21** (58%); **22** (10%); **20** (16%)[b]
32	$Mn(OAc)_3 \cdot H_2O$ (2.4), $Ti(OMe)_4$ (4.0), $PhSiH_3$ (4.0), 16 h	**21** (37%); **22** (7%); **20** (56%)[c]
33	$Mn(OAc)_3 \cdot H_2O$ (4.5), $Ti(OMe)_4$ (4.0), $PhSiH_3$ (4.0), 16 h	**21** (57%); **22** (14%); **20** (29%)[c]
34	$Mn(OAc)_3 \cdot H_2O$ (5.0), $Ti(OMe)_4$ (4.0), $PhSiH_3$ (4.0), 16 h	**21** (54%); **22** (14%); **20** (26%)[d]
35	$Mn(OAc)_3 \cdot H_2O$ (4.5), $Ti(OMe)_4$ (4.0), $PhSiH_3$ (4.0), 16 h	**21** (60%); **22** (15%); **20** (22%)[e]

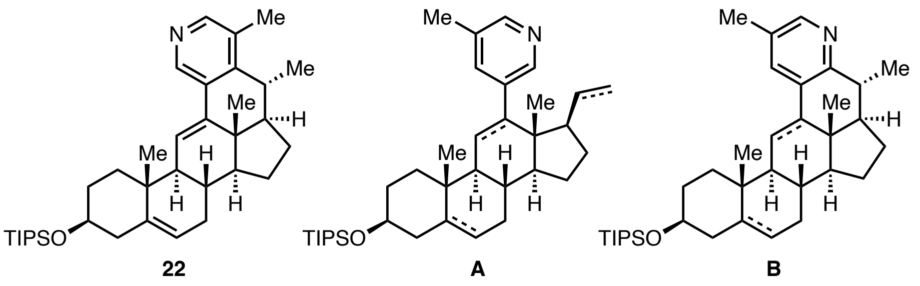

[a] with 34% of TIPS removal of **20**; [b]20 mg scale; [c]80 mg scale; [d]100 mg scale; [e]3 x 80 mg scale

Table S1. Reaction optimization of the HAT-cyclization.

We first examined the combination of $Fe(acac)_3$ and $PhSiH_3$ with trifluoroacetic acid (TFA) as additive to activate the pyridine, but only a trace amount of **21** was detected and most of the starting material **20** was recycled (entry 1). The combination of $Fe(NO_3)_3$ and $NaBH_4$ gave a trace amount of **21** as well, but with a mixture of over reduction byproducts such **A** and **B** (entry 2). Little product was obtained with the combination of $Co(acac)_3$ and *tert*-butyl hydroperoxide (TBHP) conditions (entry 3) while most of the starting material was consumed. When $BF_3{\bullet}OEt_2$ was used to activate the pyridine, about 10% of **21** was obtained with 52% of **20** recovered (entry 4). We then focused on using $BF_3{\bullet}OEt_2$ as an activator and evaluated different transition metal complexes (entry 5-10). With $Mn(OAc)_3$ (entry 10), 36% yield of desired product **21** was obtained together with 4% yield of the *para* cyclization isomer **22**. While lower the reaction temperature from 60 °C to 23 °C (entry 11) or 0 °C (entry 12) didn't change the yield of **21** much, more starting material can be recovered. Other silane reducing reagents including Ph_2SiH_2, $PhMeSiH_2$, *n*-$BuSiH_3$, and (*i*-PrO)$PhSiH_2$ were then investigated (entry 13-17). $PhSiH_3$ remained to be the optimal choice. We then examined various Lewis acids such as BCl_3, TMSOTf, TFA, $B(OMe)_3$, BiF_3, $Ti(OMe)_4$, Ti(O*i*-Pr)$_4$, $Ti(OEt)_4$, and $TiCl_4$ (entry 18-26) as the pyridine activators. To our delight, the use of titanium(IV) alkoxides (entry 23-25) gave above 50% yield of **21** and around 10% yield of **22**. $Ti(OMe)_4$ was selected as the optimal activator. Notably, reducing the reaction time has beneficial effect (entry 27-29), but increasing the scale of the starting material decreased the reaction yields, which can be countered by increasing the amount of $Mn(OAc)_3$ (entry 30-35). In the end, with the optimal reaction conditions (entry 35), 60% yield of **21** and 15% yield of **22** can be obtained with 22% yield of **20** recycled at 3 x 80 mg scale.

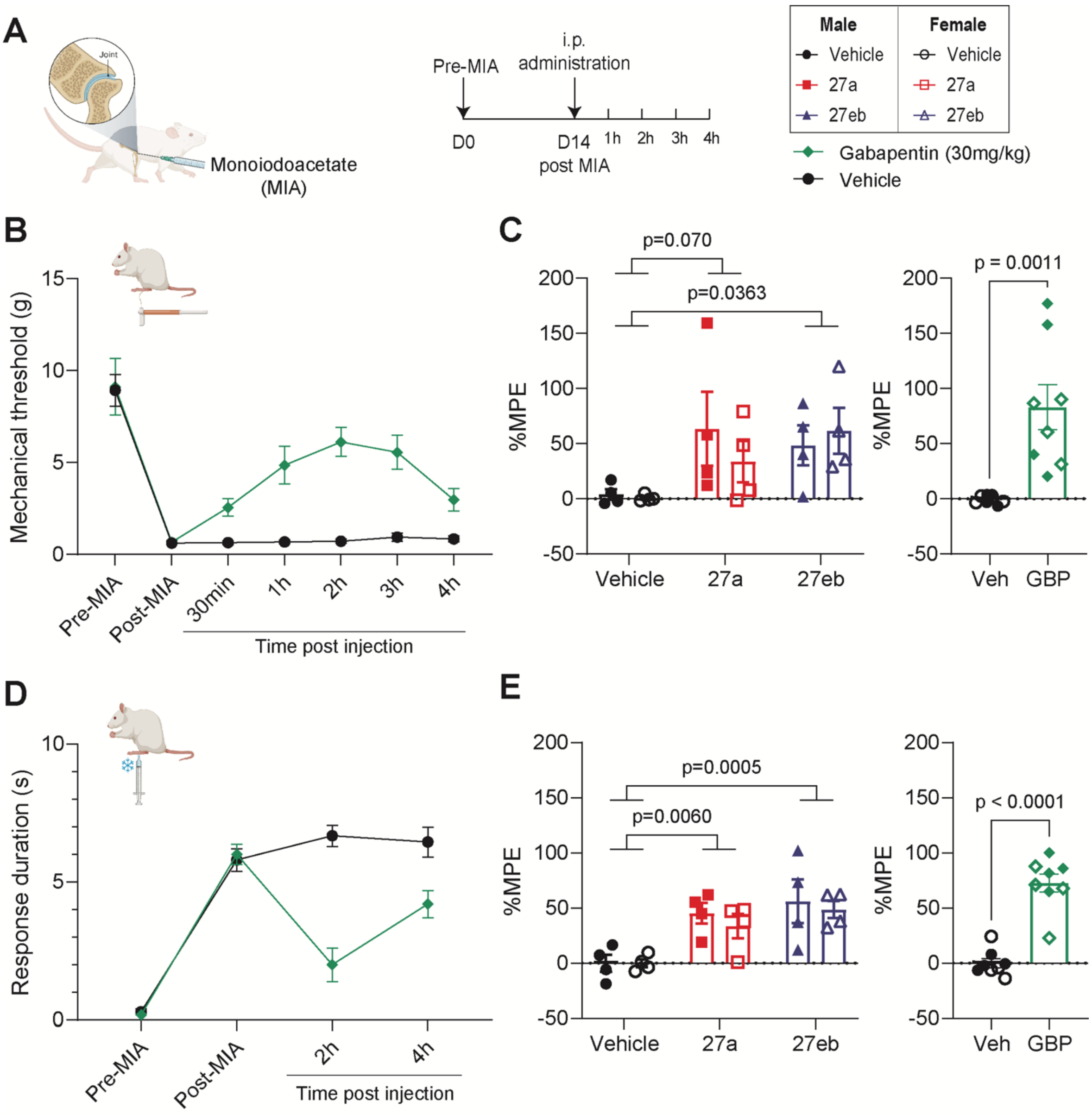

Figure S1. Intraperitoneal administration of gabapentin reverse mechanical and cold allodynia induced by the monoiodoacetate (MIA) model of osteoarthritis pain. A) Study-design schematic and treatment conditions for Gabapentin-validation. B) Baseline paw-withdrawal threshold was measured before (pre-MIA) and after (post-MIA) in both male and female rats (N=8 across sex). The response paw-withdrawal threshold was then assessed every hour following a single administration of vehicle or gabapentin (30mg/kg, i.p.) up to 4h after administration. C) Quantification of %MPE of the paw-withdrawal threshold at 2h post administration for Gabapentin(i.p)- and 1h for veragranine A (i.t.)-experiments. Gabapentin, 27a and 27eb (20µg/5µl, i.t.) significantly improved MIA-induced mechanical allodynia, when compared with vehicle across sex. D) Baseline response duration to an application of an acetone drop was measured before (pre-MIA) and after (post-MIA) in male and female rats. The response duration was then assessed every hour following a single administration of vehicle (30mg/kg, i.p.) up to 4h after administration. E) Quantification of %MPE of the response duration following the application of an acetone drop at 2h post administration for Gabapentin(i.p)- and 1h for veragranine A (i.t.)-experiments. Gabapentin, 27a and 27eb significantly improved MIA-induced cold allodynia, when compared with vehicle across sex. For panels C and E, results were compared using 2-way ANOVA and post-comparison with the sex-specific vehicle-group using Turkey's multiple comparison test, or t-test for vehicle vs GBP. For all panels, n=4 rats pr sex/group, but males and females are displayed combined in panel B+D. Results are displayed as Mean ± S.E.M.

2. General Information

All reactions sensitive to air or moisture were conducted under an argon atmosphere in dry and freshly distilled solvents under anhydrous conditions, unless otherwise noted. Anhydrous tetrahydrofuran (THF), dichloromethane (DCM), Dimethylformamide (DMF) and toluene were purified by passing the pre-degassed solvents through activated alumina columns. All other solvents and reagents were used as obtained from commercial sources (Aldrich, TCI, Alfa Aeser, Acros) without further purification unless otherwise noted. The room temperature (r.t.) is around 23 °C. Flash column chromatography was performed using silica gel (230-400 mesh). Thin layer chromatography (TLC) was performed using glass-backed silica plates (Silicycle). NMR spectra were recorded on a Bruker AV-500 spectrometer (^{1}H at 500 MHz, ^{13}C at 126 MHz, and ^{19}F at 471 MHz), or Varian INOVA400 (^{1}H at 400 MHz, and ^{13}C at 101 MHz), or Bruker AVANCE III HD 600 (^{1}H at 600 MHz, and ^{13}C at 151 MHz), or Bruker NEO 800 (^{1}H at 800 MHz, and ^{13}C at 201 MHz) at room temperature. Chemical shifts (δ) were given in ppm with reference to the solvent signal [^{1}H NMR: $CDCl_3$ (7.26), C_6D_5N (pyridine-*d*$_5$) (8.73, 7.59, **7.22**); ^{13}C NMR: $CDCl_3$ (77.16), C_6D_5N (pyridine-*d*$_5$) (150.35, **136.03**, 124.00)]. ^{1}H NMR data were reported as follows: chemical shifts (δ ppm), multiplicity (s = singlet, d = doublet, t = triplet, q = quartet, quin = quintuplet, m = multiplet, br = broad), coupling constant (Hz), and integration. ^{13}C NMR data were reported in terms of chemical shift and multiplicity. High-resolution mass measurements for compound characterization were carried out using a Waters SYNAPT G2-Si system with QuanTof analyzer or an Agilent 6550 QTOF system. IR data were recorded on a Thermo Nicolet iS50 or iS10 FT-IR.

3. Synthesis Experimental Procedures and Spectra Data

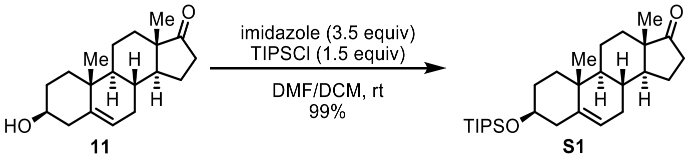

Synthesis of **S1**: Dehydroepiandrosterone (DHEA, 3.0 g, 10.4 mmol) was dissolved in 40 mL of DMF and 10 mL of DCM under argon, and imidazole (2.48 g, 36.4 mmol, 3.5 equiv) and chlorotriisopropylsilane (3.4 mL, 15.6 mmol, 1.5 equiv) were added. The reaction mixture was stirred at room temperature under argon for 20 h. The reaction mixture was quenched with saturated $NaHCO_3$ aqueous solution (50 mL), extracted with ether (3 × 40 mL), washed with brine (50 mL), dried over Na_2SO_4, filtered, and concentrated. The resulting residue was purified by flash chromatography on silica gel using EtOAc in hexanes (0~5%) as eluent to give TIPS-protected DHEA **S1** (4.96 g, 99%, 93% purity) as a white solid.

^{1}H NMR (500 MHz, $CDCl_3$) δ 5.34 (d, *J* = 4.8 Hz, 1H), 3.60 – 3.52 (m, 1H), 2.46 (dd, *J* = 19.3, 8.8 Hz, 1H), 2.34 – 2.22 (m, 2H), 2.15 – 2.03 (m, 2H), 1.94 (ddd, *J* = 12.5, 8.8, 5.8 Hz, 1H), 1.88 – 1.77 (m, 3H), 1.72 – 1.42 (m, 6H), 1.35 – 1.22 (m, 2H), 1.12 – 0.95 (m, 26H), 0.88 (s, 3H).

^{13}C NMR (126 MHz, $CDCl_3$) δ 221.4, 142.0, 120.4, 72.5, 52.0, 50.5, 47.7, 43.2, 37.5, 36.9, 36.0, 32.4, 31.7, 31.6, 31.0, 22.0, 20.5, 19.6, 18.2, 13.7, 12.5.

HRMS (ESI): *m/z* Calc. for $C_{28}H_{49}O_2Si^+$ $[M+H]^+$: 445.3496, found: 445.3487.

IR (film): 2940, 2864, 1741, 1463, 1372, 1100, 1059, 1010, 883, 798, 682 cm^{-1}.

$[\alpha]_D^{22}$ = + 6.6 (*c* = 1.0, $CHCl_3$).

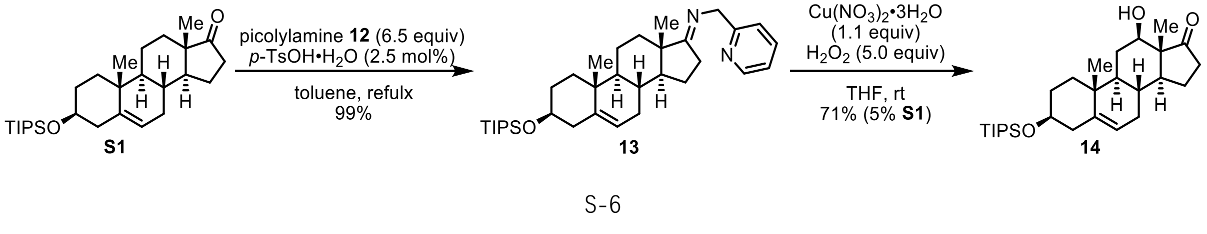

Synthesis of **13**[1]: A solution of TIPS-protected DHEA **S1** (2.22 g, 5.0 mmol), 2-picolylamine **12** (2.6 mL, 25.0 mmol, 5 equiv) and *p*-toluenesulfonic acid monohydrate (24.0 mg, 0.125 mmol, 2.5 mol%) in toluene (45 mL) was heated to reflux (145 °C) for 3 h with azeotropic removal of water using a Dean-Stark apparatus and reflux condenser. After that, another portion of 2-picolylamine **12** (0.78 mL, 7.5 mmol, 1.5 equiv) was added and reflux was continued for 2 h. After cooling to room temperature, the reaction mixture was diluted with EtOAc (50 mL), washed with saturated $NaHCO_3$ aqueous solution (2 × 20 mL) and brine (20 mL), dried over Na_2SO_4, filtered, and concentrate. The resulting residue was recrystallized from EtOAc/hexanes (1:1, 10 mL) to give pure imine **13** (150 mg, 6%) as a white solid and the filtrate was concentrated to give imine (2.597 g, 93%) as a yellow solid. The product from recrystallization or non-recrystallization are both pure, and total weight is 2.747 g.

^{1}H NMR (500 MHz, $CDCl_3$) δ 8.52 (d, *J* = 4.0 Hz, 1H), 7.65 (td, *J* = 7.7, 1.8 Hz, 1H), 7.43 (d, *J* = 7.9 Hz, 1H), 7.13 (dd, *J* = 7.5, 4.9 Hz, 1H), 5.34 (d, *J* = 4.8 Hz, 1H), 4.67 – 4.52 (m, 2H), 3.57 (tt, *J* = 10.4, 5.2 Hz, 1H), 2.45 (dd, *J* = 18.3, 8.9 Hz, 1H), 2.35 – 2.22 (m, 3H), 2.14 – 2.00 (m, 2H), 1.92 – 1.77 (m, 3H), 1.73 – 1.34 (m, 8H), 1.18 (ddd, *J* = 12.6, 10.1, 5.8 Hz, 1H), 1.12 – 0.98 (m, 25H), 0.92 (s, 3H).

^{13}C NMR (126 MHz, $CDCl_3$) δ 186.1, 160.8, 149.1, 142.1, 136.7, 121.69, 121.66, 120.7, 72.5, 58.2, 53.5, 50.8, 45.7, 43.3, 37.6, 36.9, 34.2, 32.5, 31.7, 31.5, 28.2, 23.5, 20.9, 19.7, 18.2, 16.4, 12.5.

HRMS (ESI): *m/z* Calc. for $C_{34}H_{55}N_2OSi^+$ $[M+H]^+$: 535.4078, found: 535.4067.

IR (film): 2939, 2864, 1683, 1591, 1464, 1435, 1370, 1099, 1070, 883, 798, 754, 681 cm^{-1}.

$[\alpha]_D^{23}$ = − 19.6 (*c* = 0.2, $CHCl_3$).

Synthesis of **14**[2]: A heterogeneous mixture of imine **13** (1.436 g, 2.685 mmol), $Cu(NO_3)_2{\bullet}3H_2O$ (714 mg, 2.953 mmol, 1.1 equiv) in THF (14 mL, 0.2 M) was stirred vigorously for 45 min at room temperature, and then 50 °C for 30 min to give a pale blue solution. After cooling to room temperature, hydrogen peroxide (1.4 mL, 13.4 mmol, 5.0 equiv, 30 wt % in H_2O) was then added to the reaction mixture dropwise and this was accompanied by a slight exotherm and formation of gas. The reaction mixture was then stirred for 3 h at room temperature. Then, EtOAc (14 mL) and saturated Na_4EDTA aqueous solution (14 mL) were added, and the reaction mixture was stirred for 48 h. The reaction mixture was extracted with EtOAc (3 × 50 mL), washed with brine (50 mL), dried over Na_2SO_4, filtered, and concentrated. The resulting residue was purified by flash chromatography on silica gel using EtOAc in hexanes (5%~10%) as eluent to give TIPS-protected DHEA **S1** (62.6 mg, 5%) as a white solid, and alcohol **14** (877 mg, 71%) as a white solid. **Note: the 30 wt % in H_2O hydrogen peroxide is a strong oxidizing reagent capable of reacting explosively, thus should be handled with caution**.

^{1}H NMR (500 MHz, $CDCl_3$) δ 5.34 (d, *J* = 4.5 Hz, 1H), 3.80 (dd, *J* = 11.3, 4.7 Hz, 1H), 3.55 (dtd, *J* = 11.4, 8.1, 4.0 Hz, 1H), 3.06 (br s, 1H), 2.48 (dd, *J* = 19.5, 8.6 Hz, 1H), 2.28 (d, *J* = 6.9 Hz, 2H), 2.18 – 2.05 (m, 2H), 1.99 (dddd, *J* = 12.3, 8.8, 5.9, 1.1 Hz, 1H), 1.86 – 1.77 (m, 3H), 1.72 – 1.50 (m, 4H), 1.45 (td, *J* = 12.9, 11.3 Hz, 1H), 1.25 (ddd, *J* = 12.6, 10.5, 5.9 Hz, 1H), 1.15 – 0.99 (m, 26H), 0.96 (s, 3H).

^{13}C NMR (126 MHz, $CDCl_3$) δ 223.0, 142.0, 120.3, 72.9, 72.4, 51.6, 49.7, 49.4, 43.1, 37.5, 37.0, 35.9, 32.4, 30.8, 30.6, 28.4, 21.9, 19.6, 18.2, 12.5, 8.2.

HRMS (ESI): *m/z* Calc. for $C_{28}H_{49}O_3Si^+$ $[M+H]^+$: 461.3445, found: 461.3429.

IR (film): 2940, 2865, 1731, 1464, 1102, 1065, 1047, 1018, 996, 883, 799, 684 cm^{-1}.

$[\alpha]_D^{22}$ = 0 (*c* = 0.2, $CHCl_3$).

A two-pot one-purification procedure for the synthesis of 14:

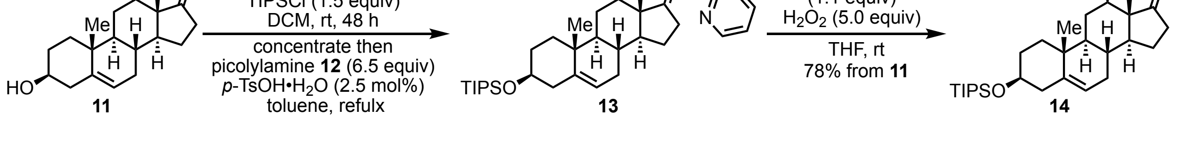

Synthesis of **14**: Dehydroepiandrosterone (DHEA, 2.88 g, 10.0 mmol) was dissolved in 10 mL of DCM under argon, and imidazole (1.09 g, 16 mmol, 1.6 equiv) and chlorotriisopropylsilane (3.3 mL, 15.0 mmol, 1.5 equiv) were added. The reaction mixture was stirred at room temperature under argon for 48 h. The reaction mixture was concentrated to give crude TIPS-protected DHEA **S1** as a white solid (100% conversion by NMR, including imidazole hydrochloride and TIPSOH), which was then dissolved with 2-picolylamine **12** (5.2 mL, 50.0 mmol, 5 equiv), and *p*-toluenesulfonic acid monohydrate (48.0 mg, 0.250 mmol, 2.5 mol%) in toluene (90 mL). The reaction mixture heated to reflux (145 °C) for 3 h with azeotropic removal of water using a dean-stark apparatus and reflux condenser. After that, another portion of 2-picolylamine **12** (1.6 mL, 15.0 mmol, 1.5 equiv) was added and reflux was continued for 2 h. After cooling to room temperature, the reaction mixture was diluted with EtOAc (90 mL), washed with saturated $NaHCO_3$ aqueous solution (2 × 50 mL) and brine (50 mL), dried over Na_2SO_4, filtered, and concentrate to give imine **13** (100% conversion by NMR, including TIPSOH) as a yellow solid.

A heterogeneous mixture of imine **13** (10.0 mmol), $Cu(NO_3)_2$•$3H_2O$ (2.66 g, 11.0 mmol, 1.1 equiv) in THF (50 mL, 0.2 M) was stirred vigorously for 45 min at room temperature, and then 50 °C for 30 min to give a pale blue solution. After cooling to room temperature, hydrogen peroxide (5.2 mL, 50.0 mmol, 5.0 equiv, 30 wt % in H_2O) was then added to the reaction mixture dropwise and this was accompanied by a slight exotherm and formation of gas. The reaction mixture was then stirred for 3 h at room temperature. Then, EtOAc (50 mL) and saturated Na_4EDTA aqueous solution (50 mL) were added, and the reaction mixture was stirred for 24 h. The reaction mixture was extracted with EtOAc (3 × 80 mL), washed with brine (50 mL), dried over Na_2SO_4, filtered, and concentrated. The resulting residue was purified by flash chromatography on silica gel using EtOAc in hexanes (5%~10%) as eluent to give TIPS-protected DHEA **S1** (43.2 mg, 1%) as a white solid, and alcohol **14** (3.58 g, 78%) as a white solid.

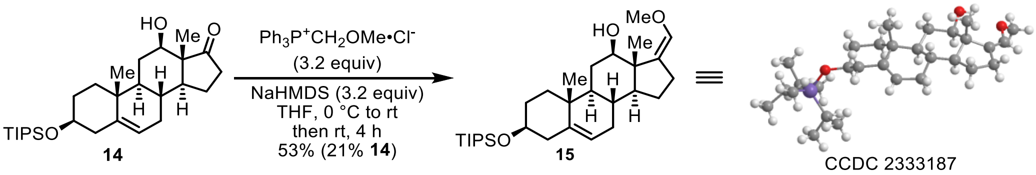

Synthesis of **15**: To a suspension of (methoxymethyl)triphenylphosphonium chloride (8.38 g, 24.5 mmol, 3.2 equiv) in THF (65 mL) was added NaHMDS (1 M in THF, 24.5 mL, 24.5 mmol, 3.2 equiv) under argon at 0 °C, and the mixture was stirred at the same temperature for 1 h. To the reaction mixture was added a solution of **14** (3.52 g, 7.64 mmol) in THF (15 mL) at 0 °C, and the resulting mixture was stirred at room temperature for 4 h. The reaction mixture was quenched with saturated NH_4Cl aqueous solution (60 mL), extracted with EtOAc (3 × 50 mL), dried over Na_2SO_4, filtered, and concentrated. The resulting residue was purified by flash chromatography on silica gel using EtOAc in hexanes (5%~7%) as eluent to give enol ether **15** (1.99 g, 53%) as a white solid and the recovered alcohol **14** (735 mg, 21%) as a white solid.

^{1}H NMR (500 MHz, $CDCl_3$) δ 5.74 (t, *J* = 1.9 Hz, 1H), 5.31 (d, *J* = 5.1 Hz, 1H), 4.85 (d, *J* = 1.1 Hz, 1H), 3.65 (dd, *J* = 11.2, 4.8 Hz, 1H), 3.61 – 3.50 (m, 4H), 2.36 (ddt, *J* = 16.1, 9.7, 1.9 Hz, 1H), 2.32 – 2.22 (m, 2H), 2.16 (dtd, *J* = 16.1, 9.0, 1.9 Hz, 1H), 2.01 (dtd, *J* = 17.2, 5.2, 2.0 Hz, 1H), 1.89 – 1.77 (m, 3H), 1.71 (dddd, *J* = 12.0, 8.5, 6.4, 1.7 Hz, 1H), 1.60 – 1.40 (m, 4H), 1.36 (tt, *J* = 12.3, 7.5 Hz, 1H), 1.11 – 0.99 (m, 27H), 0.87 (s, 3H).

^{13}C NMR (126 MHz, $CDCl_3$) δ 142.0, 136.9, 128.0, 120.8, 74.7, 72.6, 59.8, 54.5, 49.7, 49.3, 43.2, 37.4, 36.8, 32.5, 31.6, 30.4, 29.1, 26.5, 24.0, 19.5, 18.2, 12.5, 12.0.

HRMS (ESI): *m/z* Calc. for $C_{30}H_{51}O_2Si^+$ $[M-H_2O+H]^+$: 471.3653, found: 471.3642.

HRMS (ESI): *m/z* Calc. for $C_{30}H_{51}O_2Si^+$ $[M-H_2O+H]^+$: 471.3653, found: 471.3655 (2nd time); *m/z* Calc. for $C_{30}H_{53}O_3Si^+$ $[M+H]^+$: 489.3758, found: 489.3760 (very little, 2nd time).

IR (film): 3493, 2940, 2865, 1682, 1464, 1137, 1105, 1068, 996, 884, 797, 684 cm^{-1}.

$[\alpha]_D^{22}$ = − 51.5 (*c* = 0.1, $CHCl_3$).

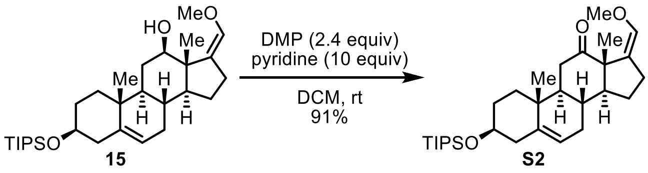

Synthesis of **S2**: To a stirred solution of enol ether **15** (195.4 mg, 0.400 mmol) in DCM (10 mL) at 0 °C were sequentially added pyridine (330 μL, 4.00 mmol, 10 equiv) and Dess-Martin periodinane (DMP, 407 mg, 0.96 mmol, 2.4 equiv). The resulting mixture was warmed to room temperature and stirred for 2 h. After that, the reaction mixture was quenched with saturated $NaHCO_3$ aqueous solution (5 mL) and saturated $Na_2S_2O_3$ aqueous solution (5 mL), extracted with DCM (3 × 10 mL), dried over Na_2SO_4, filtered and concentrated. The resulting residue was purified by flash chromatography on silica gel using EtOAc in hexanes (5%~7%~20%) as eluent to give ketone **S2** (176.5 mg, 91%) as a white solid.

^{1}H NMR (500 MHz, $CDCl_3$) δ 5.97 (s, 1H), 5.34 (d, *J* = 5.2 Hz, 1H), 3.59 – 3.48 (m, 4H), 2.72 (t, *J* = 13.0 Hz, 1H), 2.36 – 2.14 (m, 5H), 2.09 (dtd, *J* = 17.1, 5.3, 2.3 Hz, 1H), 1.95 (qd, *J* = 10.7, 5.1 Hz, 1H), 1.85 – 1.71 (m, 2H), 1.71 – 1.59 (m, 2H), 1.58 – 1.35 (m, 4H), 1.28 (s, 3H), 1.11 (s, 3H), 1.09 – 0.98 (m, 22H).

^{13}C NMR (126 MHz, $CDCl_3$) δ 210.7, 141.80, 141.77,120.8, 120.2, 72.2, 59.6, 58.0, 57.7, 53.8, 43.0, 38.0, 37.7, 37.1, 32.2, 31.7, 30.7, 27.9, 25.4, 19.1, 18.2, 17.8, 12.4.

HRMS (ESI): *m/z* Calc. for $C_{30}H_{51}O_3Si^+$ $[M+H]^+$: 487.3602, found: 487.3591.

IR (film): 2941, 2865, 1716, 1687, 1463, 1228, 1110, 1069, 997, 884, 798, 686 cm^{-1}.

$[\alpha]_D^{23}$ = + 77.9 (*c* = 0.2, $CHCl_3$).

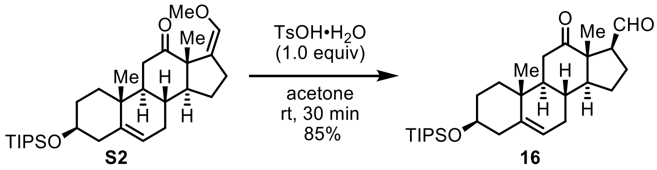

Synthesis of **16**: To a solution of ketone **S2** (311.5 mg, 0.640 mmol) in acetone (4.8 mL) was added dropwise *p*-toluenesulfonic acid monohydrate (122 mg, 0.640 mmol, 1.0 equiv) in acetone (1.6 mL). After stirring at room temperature for 0.5 h, the reaction mixture was quenched with saturated $NaHCO_3$ aqueous solution (4 mL) and then most of the acetone was removed under reduced pressure. The resulting mixture was extracted with EtOAc (3 × 2 mL), dried over Na_2SO_4, filtered, and concentrated. The resulting residue was purified by flash chromatography on silica gel using EtOAc in hexanes (5%~10%~20%~100%) as eluent to give aldehyde **16** (256 mg, 85%) as a colorless oil.

^{1}H NMR (500 MHz, $CDCl_3$) δ 9.88 (s, 1H), 5.38 – 5.32 (m, 1H), 3.56 (tt, *J* = 10.4, 4.9 Hz, 1H), 3.15 (t, *J* = 9.4 Hz, 1H), 2.58 (dd, *J* = 14.5, 12.8 Hz, 1H), 2.36 (dd, *J* = 14.5, 5.7 Hz, 1H), 2.34 – 2.23 (m, 2H), 2.19 (dddd, *J* = 14.0, 11.1, 8.9, 2.7 Hz, 1H), 2.10 (dtd, *J* = 17.3, 5.2, 2.4 Hz, 1H), 1.92 – 1.78 (m, 3H), 1.74 – 1.38 (m, 8H), 1.10 (s, 3H), 1.09 – 1.00 (m, 21H), 0.97 (s, 3H).

^{13}C NMR (126 MHz, $CDCl_3$) δ 213.7, 203.0, 141.5, 120.6, 72.2, 56.8, 56.4, 55.5, 52.8, 43.0, 37.6, 37.5, 37.2, 32.2, 31.4, 31.1, 24.0, 19.3, 19.2, 18.2, 14.0, 12.5.

HRMS (ESI): *m/z* Calc. for $C_{29}H_{49}O_3Si^+$ $[M+H]^+$: 473.3445, found: 473.3434.

IR (film): 2940, 2865, 1720, 1711, 1463, 1382, 1104, 1069, 885, 684 cm^{-1}.

$[\alpha]_D^{24}$ = + 72.2 (*c* = 0.2, $CHCl_3$).

One-Pot and Gram-Scale Synthesis of 16:

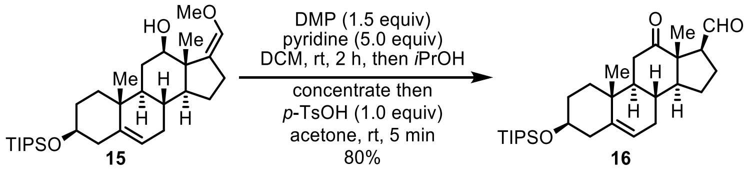

Synthesis of **16**: To a stirred solution of enol ether **15** (1.96 g, 4.00 mmol) in DCM (80 mL) at 0 °C were sequentially added pyridine (1.62 mL, 20.0 mmol, 5 equiv) and Dess-Martin periodinane (DMP, 2.54 g, 6.00 mmol, 1.5 equiv). The resulting mixture was warmed to room temperature and stirred for 2 h followed by addition of isopropyl alcohol (1.6 mL, 20.0 mmol, 5 equiv) to quench excess DMP. After that, the reaction mixture was stirred at room temperature for 3 h and concentrated to give crude ketone **S2** as a white solid (100% conversion by NMR), which was directly dissolved in acetone (30 mL) followed by a quick addition of a solution of *p*-toluenesulfonic acid monohydrate (761 mg, 4.00 mmol, 1.0 equiv) in acetone (10 mL). After stirring at room temperature for 5 min, the reaction mixture was quenched with saturated $NaHCO_3$ aqueous solution (10 mL) and saturated $Na_2S_2O_3$ aqueous solution (10 mL), and then most of the acetone was removed under reduced pressure. The resulting mixture was extracted with EtOAc (3 × 20 mL), dried over Na_2SO_4, filtered, and concentrated. The resulting residue was purified by flash chromatography on silica gel using EtOAc in hexanes (5%~7%~30%~100%) as eluent to give aldehyde **16** (1.52 g, 80%) as a colorless oil.

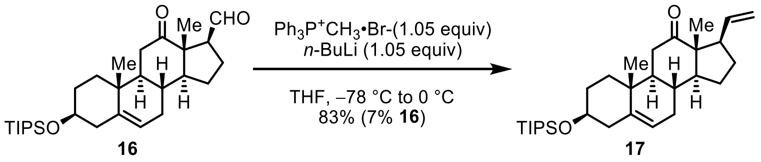

Synthesis of **17**: To a stirred suspension of methyltriphenylphosphonium bromide (1.57 g, 4.4 mmol) in THF (6.0 mL) was added dropwise *n*-BuLi (2.5 M in hexanes, 1.6 mL, 4.0 mol) at –78 °C under argon. The reaction mixture was warmed to 0 °C and stirred for 1 h. The methylenetriphenylphosphorane solution (*c* = 0.48 M = 4 mmol/8.4 mL) was obtained.

To a stirred solution of aldehyde **16** (1.76 g, 3.72 mmol) in THF (29 mL) was added the methylenetriphenylphosphorane solution (0.48 M in THF/hexanes, 8.1 mL, 1.05 equiv) at –78 °C, and the reaction was allowed to 0 °C and stirred at the same temperature for 4 h. The reaction mixture was quenched with saturated NH_4Cl aqueous solution (30 mL), extracted with EtOAc (3 × 30 mL), dried over Na_2SO_4, filtered, and concentrated. The resulting residue was purified by flash chromatography on silica gel using EtOAc in hexanes (2%~5%~10%) as eluent to give olefin **17** (1.45 g, 83%) as a white solid and the recovered impure aldehyde **16** (126.2 mg, 7%) as a colorless oil.

^{1}H NMR (500 MHz, $CDCl_3$) δ 5.94 (ddd, *J* = 17.0, 10.6, 5.9 Hz, 1H), 5.37 – 5.32 (m, 1H), 5.13 – 5.03 (m, 2H), 3.55 (tt, *J* = 10.2, 5.0 Hz, 1H), 2.84 – 2.74 (m, 1H), 2.55 (dd, *J* = 14.0, 12.8 Hz, 1H), 2.35 – 2.18 (m, 3H), 2.08 (dtd, *J* = 17.4, 5.2, 2.4 Hz, 1H), 1.90 – 1.76 (m, 4H), 1.72 – 1.36 (m, 7H), 1.10 (s, 3H), 1.09 – 0.99 (m, 22H), 0.94 (s, 3H).

^{13}C NMR (126 MHz, $CDCl_3$) δ 215.0, 141.6, 139.0, 120.9, 115.3, 72.3, 56.5, 56.2, 53.2, 46.2, 43.1, 38.0, 37.5, 37.2, 32.2, 31.7, 31.6, 25.0, 24.1, 19.2, 18.2, 13.0, 12.5.

HRMS (ESI): *m/z* Calc. for $C_{30}H_{51}O_2Si^+$ $[M+H]^+$: 471.3653, found: 471.3642.

IR (film): 2941, 2866, 1710, 1462, 1379, 1260, 1106, 1069, 884, 798, 758, 751, 685 cm^{-1}.

$[\alpha]_D^{23}$ = + 20.8 (*c* = 0.2, $CHCl_3$).

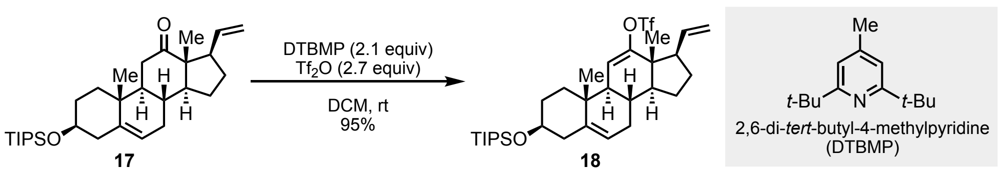

Synthesis of **18**[3]: To a solution of olefin **17** (1.10 g, 2.34 mmol) and 2,6-di-*tert*-butyl-4-methylpyridine (DTBMP, 1.30 g, 6.31 mmol, 2.7 equiv) in DCM (14 mL) at room temperature was added trifluoromethanesulfonic anhydride (0.83 mL, 4.91 mmol, 2.1 equiv). After the reaction mixture was stirred for 4 h, pentane was added and stirring was continued at 0 °C for an additional 10 min, whereupon the pyridinium salt was removed by filtration through a pad of Celite. The solids were washed with pentane and the combined organic filtrates were concentrated to afford a tan solid. The resulting residue was purified by flash chromatography on silica gel using DCM in hexanes (0%~20%~50%) as eluent to give vinyl triflate **18** (1.34g, 95%) as a white solid.

^{1}H NMR (500 MHz, $CDCl_3$) δ 5.94 – 5.83 (m, 1H), 5.54 (d, *J* = 2.4 Hz, 1H), 5.39 (d, *J* = 5.4 Hz, 1H), 5.08 (d, *J* = 6.1 Hz, 1H), 5.05 (s, 1H), 3.57 (tt, *J* = 10.7, 4.6 Hz, 1H), 2.58 (q, *J* = 8.8 Hz, 1H), 2.30 (ddd, *J* = 13.2, 4.9, 2.2 Hz, 1H), 2.26 – 2.16 (m, 1H), 2.06 – 1.57 (m, 11H), 1.43 – 1.30 (m, 1H), 1.16 – 1.03 (m, 22H), 1.02 (s, 3H), 0.96 (s, 3H).

^{13}C NMR (126 MHz, $CDCl_3$) δ 157.3, 142.2, 138.3, 121.2, 118.5 (q, *J* = 319.7 Hz), 116.3, 115.8, 72.5, 54.3, 54.0, 49.9, 47.4, 43.0, 38.0, 37.0, 32.1, 30.4, 30.2, 28.0, 23.3, 19.8, 18.2, 15.4, 12.5.

^{19}F NMR (471 MHz, $CDCl_3$) δ = –75.5.

HRMS (ESI): *m/z* Calc. for $C_{22}H_{28}F_3O_2S^+$ [M−TIPSOH+H]$^+$: 429.1706, found: 429.1696.

IR (film): 2941, 2867, 1418, 1248, 1211, 1144, 1106, 995, 902, 886, 822, 765, 751, 682 cm^{-1}.

$[\alpha]_D^{22}$ = − 26.1 (*c* = 0.1, $CHCl_3$).

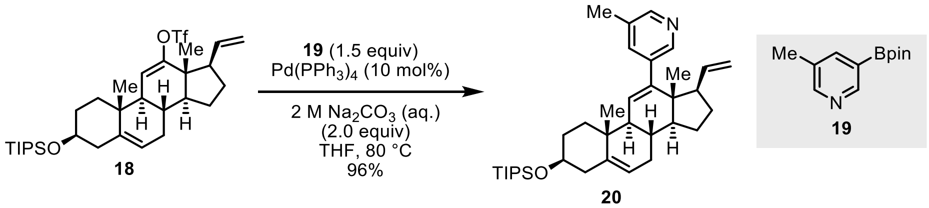

Synthesis of **20**: To a mixture of vinyl triflate **18** (302 mg, 0.500 mmol, 1.0 equiv), pinacol boronate **19** (164 mg, 0.750 mmol, 1.5 equiv), $Pd(PPh_3)_4$ (57.8 mg, 0.050 mmol, 10 mol%) were added argon degassed THF (10 mL) and argon degassed Na_2CO_3 aqueous solution (2 M in water, 1.0 mL, 2.0 mmol, 4.0 equiv). After stirring at 80 °C for 3 h, the reaction mixture was cooled to 0 °C and diluted with water (2 mL), extracted with EtOAc (3 × 4 mL), dried over Na_2SO_4, filtered, and concentrated. The resulting residue was purified by flash chromatography on silica gel using EtOAc in hexanes (5%~10%~20%) as eluent to give 3-subtituted pyridine **20** (262 mg, 96%) as a white solid.

^{1}H NMR (500 MHz, $CDCl_3$) δ 8.27 (s, 1H), 8.17 (s, 1H), 7.23 (s, 1H), 5.45 – 5.38 (m, 1H), 5.35 (d, *J* = 1.7 Hz, 1H), 5.31 (ddd, *J* = 17.1, 10.4, 8.2 Hz, 1H), 4.55 (dd, *J* = 10.4, 2.0 Hz, 1H), 4.46 (ddd, *J* = 17.1, 2.1, 1.0 Hz, 1H), 3.58 (tt, *J* = 10.8, 4.6 Hz, 1H), 2.40 (q, *J* = 9.0 Hz, 1H), 2.34 – 2.26 (m, 4H), 2.23 (tq, *J* = 13.3, 2.5 Hz, 1H), 2.07 – 1.97 (m, 1H), 1.94 – 1.64 (m, 8H), 1.63 – 1.46 (m, 2H), 1.32 (qd, *J* = 11.8, 6.5 Hz, 1H), 1.20 – 0.99 (m, 25H), 0.97 (s, 3H).

^{13}C NMR (126 MHz, $CDCl_3$) δ 148.1, 147.0, 142.6, 139.6, 137.8, 137.4, 131.5, 127.5, 121.5, 114.9, 72.7, 54.4, 53.5, 50.5, 47.8, 43.2, 37.8, 37.1, 32.2, 31.2, 30.9, 28.7, 23.4, 19.7, 18.5, 18.2, 17.1, 12.5.

HRMS (ESI): *m/z* Calc. for $C_{36}H_{56}NOSi^+$ [M+H]$^+$: 546.4126, found: 546.4111.

IR (film): 2941, 2865, 1463, 1379, 1101, 1068, 995, 911, 882, 859, 797, 751, 683 cm^{-1}.

$[\alpha]_D^{22}$ = − 25.0 (*c* = 0.2, $CHCl_3$).

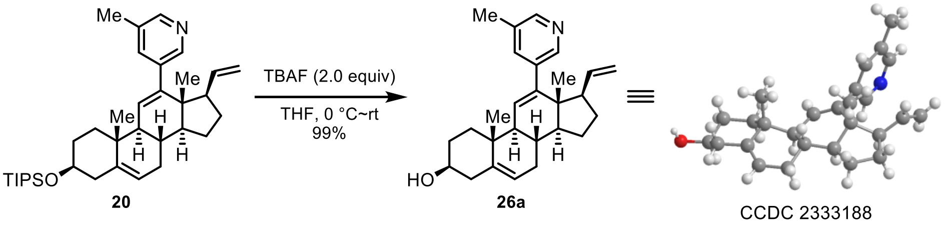

Synthesis of **26a**: To a solution of 3-subtituted pyridine **20** (21.8 mg, 40.0 μmol) in THF (1 mL) at 0 °C was added TBAF (1 M in THF, 80 μL, 80.0 μmol, 2 equiv). After stirring at room temperature for 18 h, the reaction mixture was concentrated *in vacuo*. The resulting residue was purified by flash chromatography on silica gel using EtOAc in hexanes (35%~50%) as eluent to give secondary alcohol **26a** (16.0 mg, 99%) as a white solid. The absolute configuration of **26a** has been confirmed by X-ray crystallographic analysis.

^{1}H NMR (500 MHz, CDCl$_3$) δ 8.28 (s, 1H), 8.17 (s, 1H), 7.24 (s, 1H), 5.46 (dt, *J* = 5.6, 1.9 Hz, 1H), 5.36 (d, *J* = 1.6 Hz, 1H), 5.31 (ddd, *J* = 17.1, 10.4, 8.2 Hz, 1H), 4.56 (ddd, *J* = 10.3, 2.0, 0.8 Hz, 1H), 4.47 (ddd, *J* = 17.1, 2.0, 1.0 Hz, 1H), 3.56 (ddd, *J* = 15.3, 9.9, 3.6 Hz, 1H), 2.41 (q, *J* = 9.0 Hz, 1H), 2.36 (ddd, *J* = 12.9, 4.9, 2.3 Hz, 1H), 2.29 (s, 3H), 2.24 – 2.15 (m, 1H), 2.03 (t, *J* = 11.4 Hz, 1H), 1.95 – 1.81 (m, 5H), 1.81 – 1.65 (m, 4H), 1.59 – 1.48 (m, 2H), 1.33 (qd, *J* = 11.8, 6.4 Hz, 1H), 1.18 (td, *J* = 13.5, 3.6 Hz, 1H), 1.06 (s, 3H), 0.98 (s, 3H).

^{13}C NMR (126 MHz, CDCl$_3$) δ 148.0, 147.1, 146.8, 141.8, 139.5, 137.8, 137.5, 131.6, 127.4, 122.2, 115.0, 72.0, 54.4, 53.5, 50.5, 47.8, 42.4, 37.8, 36.9, 31.5, 31.2, 30.9, 28.8, 23.4, 19.7, 18.5, 17.2.

HRMS (ESI): *m/z* Calc. for $C_{27}H_{36}NO^+$ [M+H]$^+$: 390.2791, found: 390.2776.

IR (film): 3252, 2959, 2927, 2898, 2862, 1455, 1420, 1371, 1078, 992, 920, 868, 845, 802, 723, 707, 677 cm^{-1}.

$[\alpha]_D^{23}$ = − 17.8 (*c* = 0.05, CHCl$_3$).

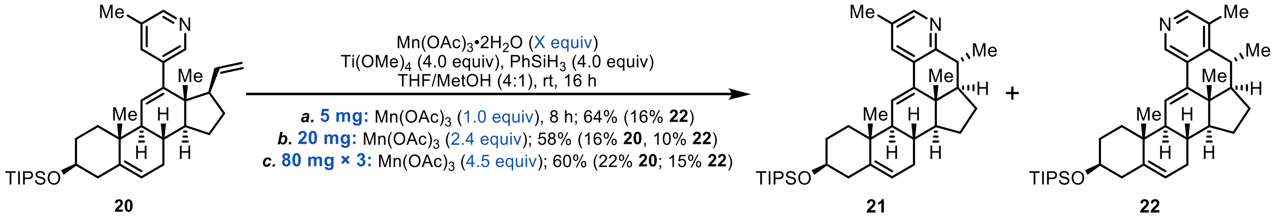

Synthesis of **21** (**5.0 mg scale**): To a solution of $Mn(OAc)_3•2H_2O$ (2.5 mg, 9.16 μmol, 1.0 equiv), 3-subtituted pyridine **20** (5.0 mg, 9.16 μmol), and Lewis acid $Ti(OMe)_4$ (6.3 mg, 36.6 μmol, 4.0 equiv) in THF/MeOH (4:1, 0.5 mL) was added $PhSiH_3$ (5.0 μL, 36.6 μmol, 4.0 equiv) at room temperature under argon. The mixture was stirred at the same temperature for 8 h. The reaction mixture was quenched with saturated $NaHCO_3$ aqueous solution (0.5 mL) and brine (0.5 mL), extracted with EtOAc (3 × 2 mL), dried over Na_2SO_4, filtered, and concentrated. The resulting residue was purified by preparative thin layer chromatography (hexanes/EtOAc = 4:1) to give TIPS-protected Veragranine A (**21**) (3.2 mg, 64%) as a white solid and TIPS-protected para-isomeric Veragranine A (**22**) (0.8 mg, 16%) as a white solid.

Synthesis of **21** (**20 mg scale**): To a solution of $Mn(OAc)_3•2H_2O$ (23.6 mg, 87.9 μmol, 2.4 equiv), 3-subtituted pyridine **20** (20.0 mg, 36.6 μmol), and Lewis acid $Ti(OMe)_4$ (25.2 mg, 146.5 μmol, 4.0 equiv) in THF/MeOH (4:1, 2.0 mL) was added $PhSiH_3$ (22 μL, 146.5 μmol, 4.0 equiv) at room temperature under argon. The mixture was stirred at the same temperature for 16 h. The reaction mixture was quenched with saturated $NaHCO_3$ aqueous solution (2 mL) and brine (2 mL), extracted with EtOAc (3 × 4 mL), dried over Na_2SO_4, filtered, and concentrated. The resulting residue was purified by preparative thin layer chromatography (4 plates, hexanes/EtOAc = 4:1) to give TIPS-protected Veragranine A (**21**) (11.7 mg, 58%) as a white solid, TIPS-protected *para*-isomeric Veragranine A (**22**) (2.0 mg, 10%) as a white solid, and the recovered 3-subtituted pyridine **20** (3.3 mg, 16%) as a white solid.

Synthesis of **21** (**80 mg** × 3 **scale**): To a solution of $Mn(OAc)_3$•$2H_2O$ (182 mg, 659 µmol, 2.4 equiv), 3-subtituted pyridine **20** (80.0 mg, 147 µmol), and Lewis acid $Ti(OMe)_4$ (106 mg, 586 µmol, 4.0 equiv) in THF/MeOH (4:1, 8.0 mL) was added $PhSiH_3$ (80 µL, 586 µmol, 4.0 equiv) at room temperature under argon. The mixture was stirred at the same temperature for 16 h. The reaction mixture was quenched with saturated $NaHCO_3$ aqueous solution (8 mL) and brine (8 mL), extracted with EtOAc (3 × 20 mL), dried over Na_2SO_4, filtered, and concentrated. The combined resulting residue was purified by flash chromatography on silica gel using EtOAc in hexanes (5%~7%~30%) as eluent to give TIPS-protected Veragranine A (**21**) (145 mg, 60%) as a white solid, TIPS-protected *para*-isomeric Veragranine A (**22**) (36.2 mg, 15%) as a white solid, and the recovered 3-subtituted pyridine **20** (52.9 mg, 22%) as a white solid.

TIPS-protected Veragranine A (**21**):

^{1}H NMR (500 MHz, $CDCl_3$) δ 8.29 (d, *J* = 2.1 Hz, 1H), 7.70 (d, *J* = 2.2 Hz, 1H), 6.07 (d, *J* = 2.8 Hz, 1H), 5.43 (dt, *J* = 5.7, 1.8 Hz, 1H), 3.62 (tt, *J* = 10.9, 4.6 Hz, 1H), 2.76 (dq, *J* = 13.3, 6.7 Hz, 1H), 2.37 – 2.29 (m, 4H), 2.29 – 2.20 (m, 1H), 2.14 – 1.97 (m, 4H), 1.90 (qd, *J* = 10.6, 4.4 Hz, 2H), 1.85 – 1.35 (m, 10H), 1.32 – 1.21 (m, 1H), 1.12 – 1.06 (m, 21H), 1.04 (s, 3H), 0.74 (s, 3H).

^{13}C NMR (126 MHz, $CDCl_3$) δ 157.7, 148.2, 142.7, 142.5, 131.5, 130.2, 128.0, 121.7, 117.7, 72.7, 56.3, 51.8, 51.6, 43.3, 41.7, 38.5, 37.9, 37.6, 32.4, 31.4, 31.3, 26.0, 23.8, 20.2, 18.3, 18.2, 18.1, 15.2, 12.5.

HRMS (ESI): *m/z* Calc. for $C_{36}H_{56}NOSi^+$ $[M+H]^+$: 546.4126, found: 546.4108.

IR (film): 2941, 2865, 1463, 1379, 1275, 1260, 1102, 1069, 884, 860, 797, 751, 682 cm^{-1}.

$[\alpha]_D^{22}$ = − 83.1 (*c* = 0.2, $CHCl_3$).

TIPS-protected *para*-isomeric Veragranine A (**22**):

^{1}H NMR (400 MHz, $CDCl_3$) δ 8.69 (s, 1H), 8.16 (s, 1H), 6.19 (d, *J* = 2.8 Hz, 1H), 5.42 (dt, *J* = 5.6, 1.8 Hz, 1H), 3.62 (tt, *J* = 10.8, 4.7 Hz, 1H), 2.74 (dq, *J* = 10.1, 6.5 Hz, 1H), 2.40 – 2.18 (m, 5H), 2.16 – 1.98 (m, 4H), 1.96 – 1.39 (m, 9H), 1.31 – 1.20 (m, 4H), 1.14 – 1.02 (m, 21H), 1.01 (s, 3H), 0.63 (s, 3H).

^{13}C NMR (101 MHz, $CDCl_3$) δ 149.8, 149.0, 144.4, 142.9, 142.5, 132.7, 128.6, 121.5, 116.7, 72.7, 56.4, 52.2, 52.0, 43.3, 41.3, 38.6, 37.5, 34.2, 32.4, 31.3, 31.0, 26.6, 23.5, 20.1, 19.5, 19.0, 18.3, 14.3, 12.5.

HRMS (ESI): *m/z* Calc. for $C_{36}H_{56}NOSi^+$ $[M+H]^+$: 546.4126, found: 546.4130.

IR (film): 2958, 2940, 2865, 1463, 1435, 1380, 1368, 1100, 1068, 1012, 995, 983, 883, 860, 796, 755, 682, 657 cm^{-1}.

$[\alpha]_D^{23}$ = − 89.4 (*c* = 0.5, $CHCl_3$).

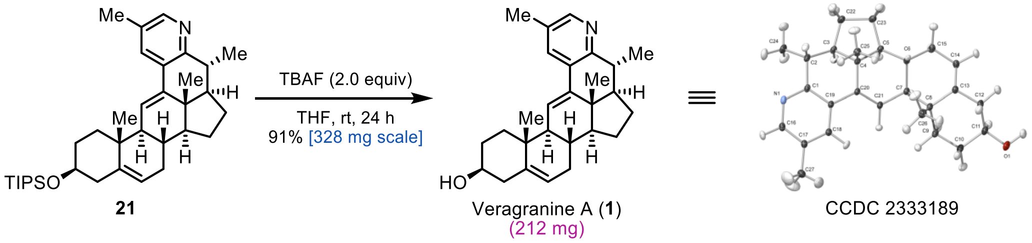

Synthesis of Veragranine A **(1)**: To a solution of TIPS-protected Veragranine A (**21**) (328 mg, 600 µmol) in THF (6 mL) at 0 °C was added TBAF (1 M in THF, 1.2 mL, 1.2 mmol, 2 equiv). After stirring at room temperature for 24 h, the reaction mixture was concentrated *in vacuo*. The resulting residue was purified by flash chromatography on silica gel using EtOAc in hexanes (35%~50%) as eluent to give veragranine A **(1)** (212.3 mg, 91%) as a white solid. The absolute configuration of veragranine A **(1)** has been confirmed by X-ray crystallographic analysis.

^{1}H NMR (500 MHz, C_6D_5N (pyridine-*d_5*)) δ 8.52 (d, *J* = 2.1 Hz, 1H), 8.06 (d, *J* = 2.2 Hz, 1H), 6.31 (s, 1H), 6.26 (d, *J* = 2.8 Hz, 1H), 5.53 (d, *J* = 5.5 Hz, 1H), 3.93 (tt, *J* = 10.9, 4.6 Hz, 1H), 2.87 (dt, *J* = 13.2, 6.7 Hz, 1H), 2.72 (ddd, *J* = 12.9, 4.9, 2.2 Hz, 1H), 2.64 – 2.54 (m, 1H), 2.27 (s, 3H), 2.17 (d, *J* = 12.3 Hz, 1H), 2.12 – 1.97 (m, 3H), 1.97 – 1.82 (m, 3H),

1.78 (d, J = 13.8 Hz, 1H), 1.75 – 1.67 (m, 1H), 1.62 (d, J = 6.7 Hz, 3H), 1.55 – 1.39 (m, 4H), 1.34 (td, J = 13.7, 3.8 Hz, 1H), 1.08 (s, 3H), 0.76 (s, 3H).

^{13}C NMR (126 MHz, C_6D_5N (pyridine-*d*$_5$)) δ 158.2, 149.0, 143.5, 143.1, 132.3, 131.0, 128.8, 122.2, 118.8, 72.0, 57.0, 52.3, 52.2, 44.0, 42.4, 39.2, 38.8, 38.1, 33.0, 32.0, 31.8, 26.6, 24.4, 20.5, 18.8, 18.3, 15.9.

HRMS (ESI): *m/z* Calc. for $C_{27}H_{36}NO^+$ $[M+H]^+$: 390.2791, found: 390.2781.

IR (film): 3432, 2975, 2955, 2919, 2858, 1458, 1436, 1399, 1376, 1347, 1289, 1263, 1221, 1120, 1068, 1047, 1007, 979, 879, 849, 808, 666, 635 cm^{-1}.

$[\alpha]_D^{24}$ = − 137.9 (c = 0.1, MeOH).

Suzuki-Miyaura Cross-Coupling with Different Pinacol Boronate:

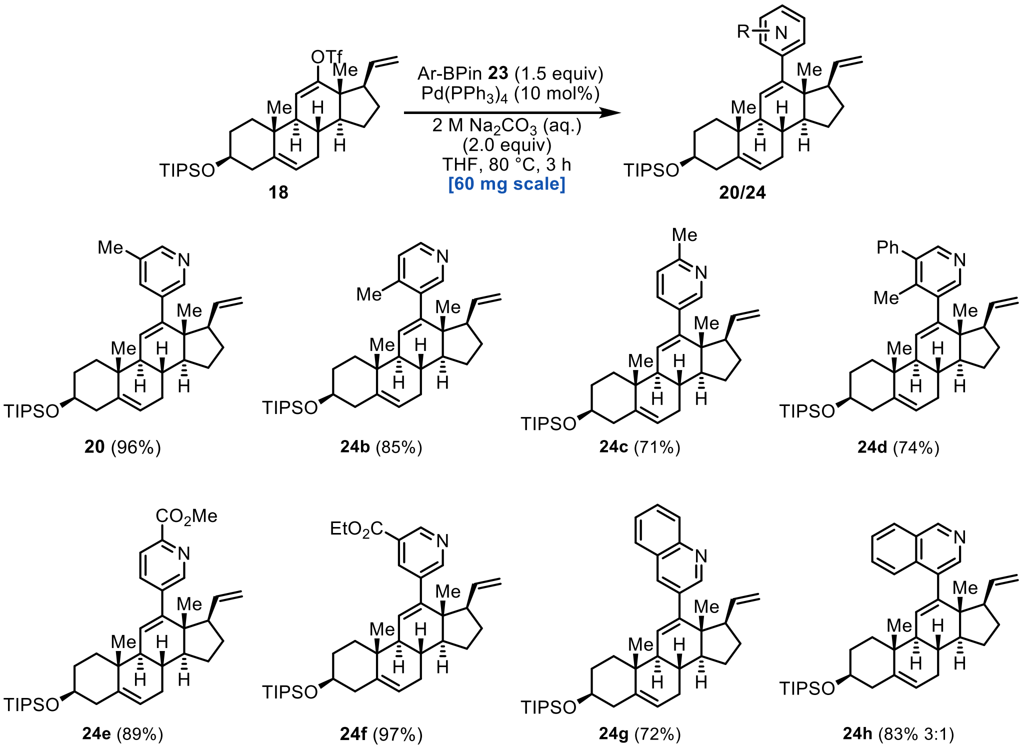

Synthesis of **24**: To a mixture of vinyl triflate **18** (60.3 mg, 0.100 mmol, 1.0 equiv), pinacol boronate (0.150 mmol, 1.5 equiv), $Pd(PPh_3)_4$ (11.6 mg, 0.010 mmol, 10 mol%) were added argon degassed THF (2 mL) and argon degassed Na_2CO_3 aqueous solution (2 M in water, 0.2 mL, 2.0 mmol, 4.0 equiv). After stirring at 80 °C for 3 h, the reaction mixture was cooled to 0 °C and diluted with water (1 mL), extracted with EtOAc (3 × 2 mL), dried over Na_2SO_4, filtered, and concentrated. The resulting residue was purified by flash chromatography on silica gel using EtOAc in hexanes as eluent to give the desired coupling product **24**.

Compound **24b** (46.4 mg, 85%, 2:1 rotamers) as a white solid; purification eluent: EtOAc in hexanes (10%~15%).

^{1}H NMR (400 MHz, $CDCl_3$) δ 8.29 (d, J = 5.0 Hz, 1H), 8.22 (s, 1H), 7.04 (d, J = 5.0 Hz, 1H), 5.42 (d, J = 5.3 Hz, 1H), 5.32 (s, 1H), 5.27 – 5.07 (m, 1H), 4.72 – 4.29 (m, 2H), 3.58 (tt, J = 10.3, 4.6 Hz, 1H), 2.71 – 2.14 (m, 6H), 2.12 – 1.99 (m, 1H), 1.98 – 1.65 (m, 8H), 1.64 – 0.78 (m, 31H).

^{13}C NMR (101 MHz, $CDCl_3$) δ 151.2, 149.7, 147.5, 145.3, 144.9, 142.7, 138.9, 138.3, 127.8, 124.8, 121.5, 113.9, 72.7, 54.5, 53.2, 50.1, 48.8, 43.3, 37.8, 37.2, 32.2, 31.25, 31.16, 28.2, 23.5, 20.3, 19.8, 18.2, 17.0, 12.5.

HRMS (ESI): *m/z* Calc. for $C_{36}H_{56}NOSi^+$ $[M+H]^+$: 546.4126, found: 546.4131.

IR (film): 2959, 2941, 2866, 1463, 1399, 1379, 1101, 1069, 1013, 995, 911, 882, 796, 680, 656 cm^{-1}.
$[\alpha]_D^{23}$ = − 13.4 (c = 0.5, $CHCl_3$).

Compound **24c** (38.9 mg, 71%) as a white solid; purification eluent: EtOAc in hexanes (5%~10%).
^{1}H NMR (400 MHz, $CDCl_3$) δ 8.24 (d, J = 2.2 Hz, 1H), 7.31 (dd, J = 7.9, 2.3 Hz, 1H), 7.00 (d, J = 7.9 Hz, 1H), 5.41 (dt, J = 5.6, 1.8 Hz, 1H), 5.38 – 5.25 (m, 2H), 4.56 (dd, J = 10.5, 2.0 Hz, 1H), 4.50 (ddd, J = 17.2, 2.0, 1.0 Hz, 1H), 3.58 (tt, J = 10.7, 4.7 Hz, 1H), 2.52 (s, 3H), 2.39 (q, J = 8.8 Hz, 1H), 2.30 (ddd, J = 13.2, 5.1, 2.0 Hz, 1H), 2.22 (ddd, J = 13.4, 10.7, 2.5 Hz, 1H), 2.08 – 1.95 (m, 1H), 1.94 – 1.46 (m, 10H), 1.42 – 0.99 (m, 26H), 0.97 (s, 3H).
^{13}C NMR (101 MHz, $CDCl_3$) δ 156.2, 149.0, 146.9, 142.7, 139.5, 137.0, 135.5, 127.6, 121.7, 121.5, 115.1, 72.7, 54.4, 53.5, 50.5, 47.8, 43.3, 37.8, 37.1, 32.2, 31.2, 30.9, 28.6, 24.2, 23.4, 19.7, 18.2, 17.1, 12.5.
HRMS (ESI): m/z Calc. for $C_{36}H_{56}NOSi^+$ $[M+H]^+$: 546.4126, found: 546.4128.
IR (film): 2958, 2940, 2865, 1486, 1463, 1437, 1378, 1101, 1068, 1012, 995, 910, 882, 861, 796, 755, 739, 681, 658, 632 cm^{-1}.
$[\alpha]_D^{23}$ = − 23.6 (c = 0.5, $CHCl_3$).

Compound **24d** (44.9 mg, 74%, 3:1 rotamers) as a colorless oil; purification eluent: EtOAc in hexanes (5%~7%).
^{1}H NMR (400 MHz, $CDCl_3$) δ 8.37 – 8.12 (m, 2H), 7.50 – 7.34 (m, 3H), 7.34 – 7.22 (m, 2H), 5.52 – 5.36 (m, 2H), 5.36 – 5.17 (m, 1H), 4.68 – 4.43 (m, 2H), 3.59 (tt, J = 10.7, 4.4 Hz, 1H), 2.70 – 2.10 (m, 6H), 2.10 – 1.67 (m, 9H), 1.67 – 1.44 (m, 2H), 1.44 – 0.95 (m, 29H).
^{13}C NMR (101 MHz, $CDCl_3$) δ 149.9, 148.4, 147.8, 147.7, 145.1, 145.0, 143.2, 142.72, 142.68, 142.4, 139.1, 138.9, 138.8, 138.2, 138.1, 137.84, 137.80, 129.6, 129.4, 128.5, 128.4, 127.9, 127.6, 121.7, 121.5, 115.4, 113.8, 72.73, 72.67, 54.8, 54.4, 53.9, 53.1, 50.1, 49.8, 49.0, 48.9, 43.3, 43.2, 38.1, 37.8, 37.22, 37.15, 32.2, 31.3, 31.2, 31.1, 29.8, 28.6, 28.3, 23.5, 22.8, 20.2, 19.8, 18.7, 18.5, 18.2, 17.9, 17.0, 12.5.
HRMS (ESI): m/z Calc. for $C_{42}H_{60}NOSi^+$ $[M+H]^+$: 622.4439, found: 622.4448.
IR (film): 2980, 2970, 2943, 2866, 1463, 1435, 1380, 1251, 1152, 1101, 1069, 991, 967, 956, 883, 797, 766, 702, 683, 655 cm^{-1}.
$[\alpha]_D^{23}$ = − 20.1 (c = 0.5, $CHCl_3$).

Compound **24e** (53.5 mg, 89%) as a white foam; purification eluent: EtOAc in hexanes (5%~10%).
^{1}H NMR (400 MHz, $CDCl_3$) δ 8.49 (dd, J = 2.2, 0.7 Hz, 1H), 7.99 (dd, J = 8.0, 0.8 Hz, 1H), 7.57 (dd, J = 8.0, 2.2 Hz, 1H), 5.50 – 5.36 (m, 2H), 5.30 (ddd, J = 17.0, 10.3, 8.6 Hz, 1H), 4.50 (dd, J = 10.3, 1.9 Hz, 1H), 4.41 (dd, J = 17.5, 1.7 Hz, 1H), 4.00 (s, 3H), 3.58 (tt, J = 10.7, 4.6 Hz, 1H), 2.42 – 2.16 (m, 3H), 2.02 (dt, J = 16.5, 3.6 Hz, 1H), 1.96 – 1.44 (m, 11H), 1.41 – 0.91 (m, 29H).
^{13}C NMR (101 MHz, $CDCl_3$) δ 166.0, 149.8, 146.4, 145.8, 142.5, 142.1, 139.0, 137.4, 128.8, 124.0, 121.5, 115.8, 72.6, 54.6, 53.5, 52.9, 50.8, 47.8, 43.2, 37.8, 37.1, 32.2, 31.1, 30.8, 28.9, 23.3, 19.8, 18.2, 17.2, 12.5.
HRMS (ESI): m/z Calc. for $C_{37}H_{56}NO_3Si^+$ $[M+H]^+$: 590.4024, found: 590.4029.
IR (film): 2941, 2865, 1749, 1725, 1463, 1436, 1379, 1368, 1307, 1234, 1132, 1104, 1068, 1012, 996, 913, 882, 859, 795, 753, 680, 656 cm^{-1}.
$[\alpha]_D^{22}$ = − 16.4 (c = 0.5, $CHCl_3$).

Compound **24f** (58.5 mg, 97%) as a light-yellow oil; purification eluent: EtOAc in hexanes (5%~7%).
^{1}H NMR (400 MHz, $CDCl_3$) δ 9.05 (d, J = 2.1 Hz, 1H), 8.50 (d, J = 2.2 Hz, 1H), 8.01 (t, J = 2.1 Hz, 1H), 5.49 – 5.37 (m, 2H), 5.31 (ddd, J = 17.0, 10.3, 8.7 Hz, 1H), 4.50 (dd, J = 10.3, 2.0 Hz, 1H), 4.46 – 4.31 (m, 3H), 3.58 (ddd, J = 10.8, 6.1,

4.6 Hz, 1H), 2.40 (q, J = 9.1 Hz, 1H), 2.30 (ddd, J = 13.2, 5.1, 2.0 Hz, 1H), 2.22 (ddd, J = 13.2, 10.8, 2.5 Hz, 1H), 2.03 (ddt, J = 17.0, 5.9, 2.5 Hz, 1H), 1.97 – 1.65 (m, 8H), 1.64 – 0.93 (m, 34H).

^{13}C NMR (101 MHz, $CDCl_3$) δ 165.7, 153.2, 148.8, 146.1, 142.5, 139.2, 138.0, 137.6, 128.7, 124.9, 121.5, 115.6, 72.6, 61.5, 54.6, 53.5, 50.8, 47.8, 43.2, 37.8, 37.1, 32.2, 31.1, 30.9, 29.1, 23.4, 19.8, 18.2, 17.2, 14.4, 12.5.

HRMS (ESI): *m/z* Calc. for $C_{38}H_{58}NO_3Si^+$ $[M+H]^+$: 604.4180, found: 604.4187.

IR (film): 2960, 2941, 2866, 1725, 1463, 1444, 1380, 1368, 1297, 1241, 1105, 1069, 1026, 1013, 995, 912, 883, 769, 680 cm^{-1}.

$[\alpha]_D^{22}$ = − 18.0 (c = 0.5, $CHCl_3$).

Compound **24g** (41.8 mg, 72%) as a white solid; purification eluent: EtOAc in hexanes (5%).

^{1}H NMR (400 MHz, $CDCl_3$) δ 8.73 (d, J = 2.2 Hz, 1H), 8.07 (dd, J = 8.5, 1.1 Hz, 1H), 7.85 (d, J = 2.1 Hz, 1H), 7.76 (dd, J = 8.1, 1.4 Hz, 1H), 7.67 (ddd, J = 8.5, 6.9, 1.5 Hz, 1H), 7.52 (ddd, J = 8.1, 6.9, 1.2 Hz, 1H), 5.48 (d, J = 1.6 Hz, 1H), 5.44 (dt, J = 5.7, 1.8 Hz, 1H), 5.30 (ddd, J = 17.1, 10.3, 8.3 Hz, 1H), 4.46 – 4.35 (m, 2H), 3.60 (tt, J = 10.7, 4.7 Hz, 1H), 2.51 (q, J = 8.9 Hz, 1H), 2.32 (ddd, J = 13.2, 5.0, 2.0 Hz, 1H), 2.24 (ddd, J = 13.2, 10.8, 2.5 Hz, 1H), 2.12 – 1.46 (m, 11H), 1.44 – 0.98 (m, 29H).

^{13}C NMR (101 MHz, $CDCl_3$) δ 151.9, 147.2, 146.9, 142.6, 139.3, 135.9, 135.2, 129.2, 128.9, 128.6, 127.8, 127.5, 126.6, 121.6, 115.4, 72.7, 54.6, 53.6, 50.7, 48.1, 43.3, 37.9, 37.1, 32.2, 31.2, 31.0, 28.8, 23.5, 19.8, 18.3, 17.3, 12.5.

HRMS (ESI): *m/z* Calc. for $C_{39}H_{56}NOSi^+$ $[M+H]^+$: 582.4126, found: 582.4131.

IR (film): 2940, 2865, 1464, 1379, 1103, 1068, 1013, 996, 910, 883, 859, 796, 786, 751, 679, 657 cm^{-1}.

$[\alpha]_D^{22}$ = − 11.2 (c = 0.5, $CHCl_3$).

Compound **24h** (48.5 mg, 83%, 3:1 rotamers) as a white foam; purification eluent: EtOAc in hexanes (5%~10%).

^{1}H NMR (400 MHz, $CDCl_3$) δ 9.19 – 9.05 (m, 1H), 8.34 – 8.19 (m, 1H), 7.99 – 7.85 (m, 2H), 7.67 (ddd, J = 8.3, 6.8, 1.3 Hz, 1H), 7.57 (ddd, J = 8.1, 6.8, 1.2 Hz, 1H), 5.51 (d, J = 1.8 Hz, 1H), 5.49 – 5.41 (m, 1H), 5.05 – 4.88 (m, 1H), 4.60 – 3.32 (m, 3H), 2.75 – 2.19 (m, 3H), 2.16 – 1.66 (m, 9H), 1.65 – 0.97 (m, 31H).

^{13}C NMR (101 MHz, $CDCl_3$) δ 151.1, 151.0, 143.8, 143.7, 143.6, 142.8, 142.4, 142.0, 138.9, 138.4, 136.4, 135.9, 133.6, 133.5, 129.9, 129.8, 129.5, 128.6, 128.40, 128.36, 127.7, 126.85, 126.75, 126.0, 125.8, 121.8, 121.5, 115.3, 113.2, 72.74, 72.70, 54.8, 54.5, 53.8, 53.2, 50.2, 49.9, 48.9, 48.8, 43.29, 43.27, 38.1, 37.9, 37.2, 37.1, 32.2, 31.7, 31.4, 31.30, 31.27, 31.2, 28.4, 28.3, 23.7, 23.6, 22.8, 20.3, 19.8, 18.2, 18.1, 16.7, 14.3, 12.5.

HRMS (ESI): *m/z* Calc. for $C_{39}H_{56}NOSi^+$ $[M+H]^+$: 582.4126, found: 582.4132.

IR (film): 2940, 2865, 1463, 1379, 1373, 1101, 1067, 1013, 995, 913, 882, 860, 795, 753, 683, 666, 658 cm^{-1}.

$[\alpha]_D^{22}$ = − 12.0 (c = 0.5, $CHCl_3$).

HAT Reaction for the Synthesis of TIPS-Protected Veragranine A Analogs

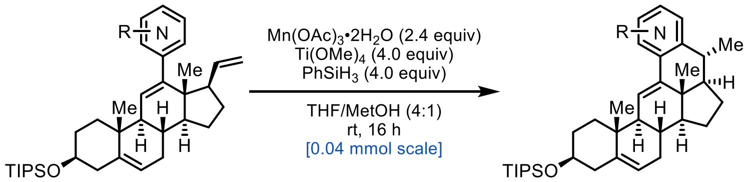

24 → **25**

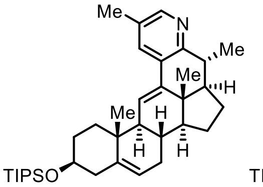

21 (58%; 16% SM)

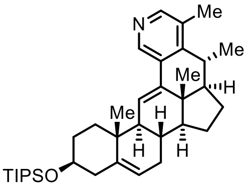

22 (10%)

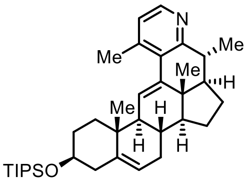

25b (60%; 40% SM)

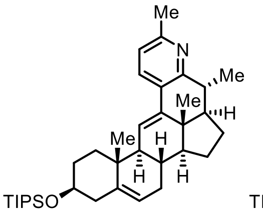

25ca (12%, 73% SM)

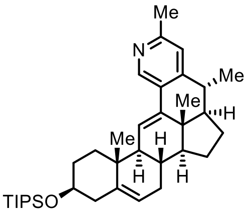

25cb (7%)

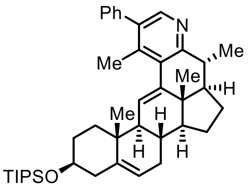

25d (49%, 41% SM)

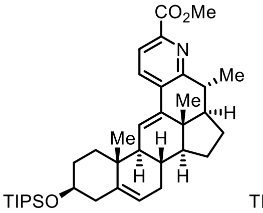

25ea (10%, 52% SM)

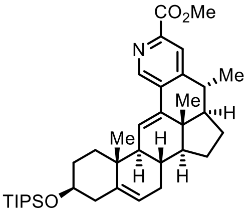

25eb (22%)

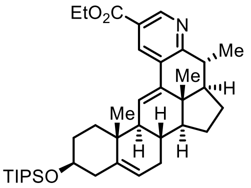

25f (50%, 34% SM)

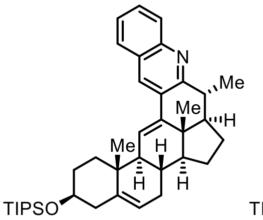

25ga (7%, 18% SM)

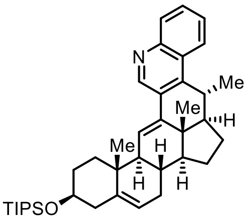

25gb (52%)

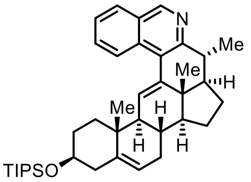

25h (30%, 34% SM)

Synthesis of **25** (0.04 mmol scale): To a solution of $Mn(OAc)_3•2H_2O$ (26.5 mg, 96.0 µmol, 2.4 equiv), compound **24** (40.0 µmol), and Lewis acid $Ti(OMe)_4$ (29.0 mg, 160 µmol, 4.0 equiv) in THF/MeOH (4:1, 2.0 mL) was added $PhSiH_3$ (22 µL, 160 µmol, 4.0 equiv) at room temperature under argon. The reaction stirred at the same temperature for 16 h. The reaction mixture was quenched with saturated $NaHCO_3$ aqueous solution (2 mL) and brine (2 mL), extracted with EtOAc (3 × 4 mL), dried over Na_2SO_4, filtered, and concentrated. The resulting residue was purified by preparative thin layer chromatography (4 plates, hexanes/EtOAc) or flash chromatography on silica gel using EtOAc in hexanes as eluent to give TIPS-protected Veragranine A analog (**25**) and the recovered **24**.

Compound **25b** (13.1 mg, 60%; 8.7 mg, 40% SM) as a white solid.
Flash chromatography: EtOAc in hexanes (5%~10%~15%).

¹H NMR (400 MHz, CDCl₃) δ 8.28 (d, *J* = 4.8 Hz, 1H), 6.95 (d, *J* = 4.8 Hz, 1H), 6.05 (d, *J* = 2.8 Hz, 1H), 5.44 (dt, *J* = 5.7, 1.8 Hz, 1H), 3.62 (tt, *J* = 10.8, 4.7 Hz, 1H), 2.72 (dq, *J* = 12.6, 6.5 Hz, 1H), 2.53 (s, 3H), 2.34 (ddd, *J* = 13.1, 5.1, 2.1 Hz, 1H), 2.31 – 2.21 (m, 1H), 2.14 – 1.38 (m, 16H), 1.35 – 0.99 (m, 25H), 0.69 (s, 3H).
¹³C NMR (101 MHz, CDCl₃) δ 161.8, 145.8, 145.7, 143.1, 142.7, 128.8, 125.9, 125.2, 121.8, 72.7, 57.5, 53.2, 51.6, 43.4, 42.4, 39.3, 38.5, 37.8, 32.5, 31.3, 30.4, 26.3, 25.5, 23.3, 20.6, 18.9, 18.3, 14.8, 12.5.
HRMS (ESI): *m/z* Calc. for $C_{36}H_{56}NOSi^+$ $[M+H]^+$: 546.4126, found: 546.4123.
IR (film): 2940, 2865, 1462, 1379, 1260, 1101, 1068, 1013, 996, 882, 862, 823, 796, 755, 679, 660 cm^{-1}.
$[\alpha]_D^{23}$ = − 148.0 (*c* = 0.2, $CHCl_3$).

Compound **25ca** (2.6 mg, 12%; 16.0 mg, 73% SM) as a colorless oil and **25cb** (1.6 mg, 7%) as a white solid.
Preparative TLC: hexanes/EtOAc = 9:1.
Compound **25ca**:
¹H NMR (600 MHz, CDCl₃) δ 7.79 (d, *J* = 8.0 Hz, 1H), 6.94 (d, *J* = 8.0 Hz, 1H), 6.01 (d, *J* = 2.8 Hz, 1H), 5.43 (dt, *J* = 5.7, 1.8 Hz, 1H), 3.62 (tt, *J* = 10.9, 4.6 Hz, 1H), 2.76 (td, *J* = 12.1, 5.5 Hz, 1H), 2.52 (s, 3H), 2.32 (ddd, *J* = 13.1, 4.9, 2.3 Hz, 1H), 2.24 (ddq, *J* = 13.3, 11.0, 2.5 Hz, 1H), 2.10 – 1.95 (m, 4H), 1.94 – 1.86 (m, 2H), 1.84 – 1.70 (m, 2H), 1.70 – 1.35 (m, 8H), 1.35 – 0.94 (m, 25H), 0.74 (s, 3H).
¹³C NMR (151 MHz, CDCl₃) δ 159.7, 156.2, 142.8, 142.6, 131.4, 125.6, 121.7, 120.7, 116.6, 72.8, 56.2, 51.8, 51.6, 43.3, 41.7, 38.5, 38.3, 37.6, 32.4, 31.4, 31.3, 26.1, 24.6, 23.8, 20.1, 18.4, 18.3, 15.2, 12.5.
HRMS (ESI): *m/z* Calc. for $C_{36}H_{56}NOSi^+$ $[M+H]^+$: 546.4126, found: 546.4124.
IR (film): 2940, 2865, 1463, 1437, 1378, 1099, 1068, 1012, 995, 882, 860, 820, 795, 755, 740, 680, 659 cm^{-1}.
$[\alpha]_D^{23}$ = − 82.8 (*c* = 0.05, $CHCl_3$).
Compound **25cb**:
¹H NMR (800 MHz, CDCl₃) δ 8.75 (s, 1H), 7.12 (s, 1H), 6.14 (d, *J* = 2.9 Hz, 1H), 5.42 (d, *J* = 5.4 Hz, 1H), 3.62 (tt, *J* = 10.6, 4.6 Hz, 1H), 2.64 (dq, *J* = 13.4, 6.8 Hz, 1H), 2.54 (s, 3H), 2.32 (ddd, *J* = 13.1, 4.9, 2.3 Hz, 1H), 2.25 (ddd, *J* = 13.4, 10.9, 2.8 Hz, 1H), 2.11 – 1.96 (m, 4H), 1.95 – 1.87 (m, 2H), 1.82 – 1.22 (m, 11H), 1.12 – 1.03 (m, 24H), 0.70 (s, 3H).
¹³C NMR (201 MHz, CDCl₃) δ 155.6, 150.9, 144.9, 142.9, 141.5, 126.6, 122.1, 121.5, 116.9, 72.8, 56.2, 51.8, 50.9, 43.4, 41.8, 38.5, 37.5, 34.6, 32.4, 31.4, 31.2, 26.0, 24.0, 23.7, 20.1, 18.3, 18.2, 15.1, 12.5.
HRMS (ESI): *m/z* Calc. for $C_{36}H_{56}NOSi^+$ $[M+H]^+$: 546.4126, found: 546.4130.
IR (film): 2957, 2927, 2865, 1596, 1463, 1436, 1379, 1100, 1068, 1013, 996, 882, 861, 795, 764, 750, 680, 659 cm^{-1}.
$[\alpha]_D^{23}$ = − 26.1 (*c* = 0.05, $CHCl_3$).

Compound **25d** (12.2 mg, 49%; 10.1 mg, 41% SM) as a light-yellow oil.
Preparative TLC: hexanes/EtOAc = 9:1.
¹H NMR (600 MHz, CDCl₃) δ 8.28 (s, 1H), 7.46 – 7.40 (m, 2H), 7.40 – 7.35 (m, 1H), 7.34 – 7.30 (m, 2H), 6.10 (d, *J* = 2.7 Hz, 1H), 5.44 (dt, *J* = 5.7, 1.9 Hz, 1H), 3.61 (tt, *J* = 10.9, 4.6 Hz, 1H), 2.76 (dt, *J* = 13.1, 6.6 Hz, 1H), 2.43 (s, 3H), 2.34 (ddd, *J* = 13.2, 4.9, 2.3 Hz, 1H), 2.29 – 2.22 (m, 1H), 2.11 – 2.01 (m, 3H), 1.92 (dt, *J* = 13.1, 3.5 Hz, 1H), 1.90 – 1.73 (m, 4H), 1.67 – 1.43 (m, 8H), 1.32 – 1.24 (m, 1H), 1.11 (s, 3H), 1.10 – 1.03 (m, 21H), 0.75 (s, 3H).
¹³C NMR (151 MHz, CDCl₃) δ 160.6, 146.2, 143.5, 142.8, 142.6, 139.4, 137.3, 129.9, 129.4, 128.5, 127.5, 127.0, 121.8, 72.6, 57.7, 53.5, 52.0, 43.4, 42.6, 39.2, 38.5, 37.8, 32.4, 31.3, 30.3, 26.3, 23.3, 23.1, 20.7, 18.7, 18.2, 14.7, 12.5.
HRMS (ESI): *m/z* Calc. for $C_{42}H_{60}NOSi^+$ $[M+H]^+$: 622.4439, found: 622.4445.
IR (film): 2941, 2866, 1458, 1432, 1376, 1275, 1260, 1104, 1068, 1009, 996, 883, 861, 795, 764, 750, 702, 679, 653 cm^{-1}.
$[\alpha]_D^{23}$ = − 149.1 (*c* = 0.2, $CHCl_3$).

Compound **25ea** (2.4 mg, 10%; 12.3 mg, 52% SM) as a colorless oil and **25eb** (5.2 mg, 22%) as a white solid.
Preparative TLC: hexanes/EtOAc = 7:1.
Compound **25ea**:
^{1}H NMR (400 MHz, CDCl$_3$) δ 8.02 (d, *J* = 8.2 Hz, 1H), 7.91 (dd, *J* = 8.2, 0.7 Hz, 1H), 6.24 (d, *J* = 2.9 Hz, 1H), 5.44 (d, *J* = 5.2 Hz, 1H), 3.98 (s, 3H), 3.62 (tt, *J* = 10.3, 4.7 Hz, 1H), 2.91 (dq, *J* = 13.3, 6.7 Hz, 1H), 2.33 (ddd, *J* = 13.1, 4.9, 2.1 Hz, 1H), 2.29 – 2.19 (m, 1H), 2.12 – 1.98 (m, 4H), 1.98 – 1.38 (m, 12H), 1.34 – 1.18 (m, 1H), 1.13 – 0.99 (m, 24H), 0.74 (s, 3H).
^{13}C NMR (101 MHz, CDCl$_3$) δ 166.2, 160.9, 145.4, 142.6, 142.1, 132.1, 131.7, 122.6, 121.7, 121.2, 72.7, 56.5, 52.9, 51.7, 51.3, 43.3, 41.6, 38.60, 38.56, 37.6, 32.3, 31.3, 31.2, 26.0, 23.8, 20.3, 18.29, 18.26, 15.3, 12.5.
HRMS (ESI): *m/z* Calc. for $C_{37}H_{56}NO_3Si^+$ $[M+H]^+$: 590.4024, found: 590.4033.
IR (film): 2927, 2865, 1745, 1720, 1462, 1442, 1431, 1379, 1309, 1275, 1264, 1246, 1193, 1137, 1097, 1068, 1013, 989, 882, 852, 797, 755, 738, 681, 657 cm^{-1}.
$[\alpha]_D^{23}$ = − 87.5 (*c* = 0.05, $CHCl_3$).
Compound **25eb**:
^{1}H NMR (400 MHz, CDCl$_3$) δ 8.94 (s, 1H), 8.12 (s, 1H), 6.33 (d, *J* = 3.0 Hz, 1H), 5.43 (dt, *J* = 5.8, 1.8 Hz, 1H), 4.00 (s, 3H), 3.62 (tt, *J* = 10.9, 4.7 Hz, 1H), 2.72 (dq, *J* = 13.3, 6.8 Hz, 1H), 2.33 (ddd, *J* = 13.2, 5.1, 2.2 Hz, 1H), 2.29 – 2.19 (m, 1H), 2.12 – 1.98 (m, 4H), 1.98 – 1.38 (m, 9H), 1.36 (d, *J* = 6.7 Hz, 3H), 1.33 – 1.22 (m, 1H), 1.12 – 1.01 (m, 24H), 0.71 (s, 3H).
^{13}C NMR (101 MHz, CDCl$_3$) δ 166.4, 150.7, 146.1, 145.5, 142.8, 141.3, 132.3, 124.5, 121.5, 121.0, 72.7, 56.5, 52.9, 51.7, 50.7, 43.3, 41.8, 38.6, 37.6, 34.8, 32.4, 31.3, 31.2, 25.9, 23.7, 20.3, 18.25, 18.16, 15.2, 12.5.
HRMS (ESI): *m/z* Calc. for $C_{37}H_{56}NO_3Si^+$ $[M+H]^+$: 590.4024, found: 590.4031.
IR (film): 2941, 2865, 1722, 1463, 1436, 1248, 1235, 1103, 1068, 883, 793, 752, 680, 662 cm^{-1}.
$[\alpha]_D^{23}$ = − 105.2 (*c* = 0.1, $CHCl_3$).

Compound **25f** (12.0 mg, 50%; 8.3 mg, 34% SM) as a light-yellow solid.
Preparative TLC: hexanes/EtOAc = 9:1.
^{1}H NMR (400 MHz, CDCl$_3$) δ 9.02 (d, *J* = 2.1 Hz, 1H), 8.46 (d, *J* = 2.1 Hz, 1H), 6.20 (d, *J* = 2.9 Hz, 1H), 5.43 (dt, *J* = 5.7, 1.8 Hz, 1H), 4.42 (q, *J* = 7.1 Hz, 2H), 3.69 – 3.56 (m, 1H), 2.83 (dt, *J* = 13.4, 6.8 Hz, 1H), 2.33 (ddd, *J* = 13.1, 5.0, 2.2 Hz, 1H), 2.29 – 2.20 (m, 1H), 2.16 – 1.98 (m, 4H), 1.97 – 1.35 (m, 15H), 1.32 – 1.18 (m, 1H), 1.13 – 1.00 (m, 24H), 0.73 (s, 3H).
^{13}C NMR (101 MHz, CDCl$_3$) δ 166.0, 164.8, 148.2, 142.8, 141.6, 132.2, 128.7, 123.9, 121.6, 119.6, 72.7, 61.4, 56.4, 51.7, 51.0, 43.3, 41.7, 38.8, 38.6, 37.6, 32.3, 31.3, 31.2, 26.0, 23.8, 20.2, 18.3, 17.9, 15.2, 14.5, 12.5.
HRMS (ESI): *m/z* Calc. for $C_{38}H_{58}NO_3Si^+$ $[M+H]^+$: 604.4180, found: 604.4177.
IR (film): 2957, 2940, 2866, 1724, 1463, 1379, 1368, 1299, 1256, 1249, 1230, 1104, 1068, 1028, 996, 882, 860, 795, 765, 750, 680 cm^{-1}.
$[\alpha]_D^{23}$ = − 78.1 (*c* = 0.2, $CHCl_3$).

Compound **25ga** (1.6 mg, 7%; 4.1 mg, 18% SM) as a colorless oil and **25gb** (12.2 mg, 52%) as a colorless oil.
Preparative TLC: hexanes/EtOAc = 9:1.
Compound **25ga**:
^{1}H NMR (400 MHz, CDCl$_3$) δ 8.34 (s, 1H), 7.99 (d, *J* = 8.4 Hz, 1H), 7.76 (d, *J* = 8.1 Hz, 1H), 7.61 (t, *J* = 7.6 Hz, 1H), 7.45 (t, *J* = 7.5 Hz, 1H), 6.29 (d, *J* = 2.9 Hz, 1H), 5.45 (d, *J* = 5.3 Hz, 1H), 3.65 (tt, *J* = 10.4, 4.7 Hz, 1H), 2.96 (dq, *J* = 12.5, 6.7, 6.0 Hz, 1H), 2.43 – 2.31 (m, 1H), 2.31 – 2.22 (m, 1H), 2.16 (dt, *J* = 13.0, 3.2 Hz, 1H), 2.13 – 2.00 (m, 3H), 2.00 – 1.45 (m, 15H), 1.37 – 1.23 (m, 1H), 1.16 – 1.00 (m, 21H), 0.80 (s, 3H).

^{13}C NMR (151 MHz, CDCl$_3$) δ 162.3, 147.0, 142.8, 142.7, 129.7, 128.94, 128.87, 127.4, 127.3, 126.8, 126.1, 121.7, 118.3, 72.8, 56.4, 52.0, 51.3, 43.4, 41.8, 39.2, 38.6, 37.7, 32.4, 31.4, 31.3, 26.3, 23.8, 20.2, 18.4, 18.3, 15.5, 12.5.

HRMS (ESI): *m/z* Calc. for $C_{39}H_{56}NOSi^+$ $[M+H]^+$: 582.4126, found: 582.4131.

IR (film): 2925, 2865, 1464, 1437, 1379, 1102, 1068, 1014, 996, 884, 861, 795, 789, 753, 687, 682, 654 cm^{-1}.

$[\alpha]_D^{23}$ = − 26.1 (*c* = 0.05, $CHCl_3$).

Compound **25gb**:

^{1}H NMR (400 MHz, CDCl$_3$) δ 9.17 (s, 1H), 8.05 (td, *J* = 8.4, 1.3 Hz, 2H), 7.60 (ddd, *J* = 8.3, 6.8, 1.4 Hz, 1H), 7.50 (ddd, *J* = 8.2, 6.8, 1.4 Hz, 1H), 6.36 (d, *J* = 2.8 Hz, 1H), 5.44 (dt, *J* = 5.6, 1.8 Hz, 1H), 3.63 (td, *J* = 10.8, 5.3 Hz, 1H), 3.30 (dq, *J* = 9.7, 6.6 Hz, 1H), 2.33 (ddd, *J* = 13.1, 5.0, 2.2 Hz, 1H), 2.30 – 2.01 (m, 5H), 1.99 – 1.48 (m, 9H), 1.41 (d, *J* = 6.5 Hz, 3H), 1.36 – 1.21 (m, 1H), 1.17 – 1.04 (m, 21H), 1.03 (s, 3H), 0.69 (s, 3H).

^{13}C NMR (101 MHz, CDCl$_3$) δ 147.9, 147.5, 146.3, 142.9, 142.8, 130.1, 128.0, 127.9, 125.7, 125.64, 125.58, 121.6, 117.7, 72.7, 56.7, 52.1, 51.9, 43.4, 41.3, 38.6, 37.6, 33.4, 32.4, 31.4, 31.2, 26.5, 23.6, 22.7, 20.2, 18.3, 14.4, 12.5.

HRMS (ESI): *m/z* Calc. for $C_{39}H_{56}NOSi^+$ $[M+H]^+$: 582.4126, found: 582.4129.

IR (film): 2958, 2940, 2865, 1497, 1463, 1436, 1380, 1371, 1101, 1068, 1012, 996, 882, 859, 797, 761, 682 cm^{-1}.

$[\alpha]_D^{23}$ = − 107.2 (*c* = 0.2, $CHCl_3$).

Compound **25h** (7.0 mg, 30%; 8.0 mg, 34% SM) as a white solid.

Preparative TLC: hexanes/EtOAc = 9:1.

^{1}H NMR (400 MHz, CDCl$_3$) δ 9.06 (s, 1H), 8.55 (d, *J* = 8.7 Hz, 1H), 7.93 (dd, *J* = 8.2, 1.4 Hz, 1H), 7.66 (ddd, *J* = 8.6, 6.8, 1.5 Hz, 1H), 7.52 (ddd, *J* = 7.9, 6.8, 0.9 Hz, 1H), 6.39 (d, *J* = 2.7 Hz, 1H), 5.47 (dt, *J* = 5.6, 1.8 Hz, 1H), 3.64 (tt, *J* = 10.3, 4.8 Hz, 1H), 2.83 (dq, *J* = 13.4, 6.8 Hz, 1H), 2.44 – 2.26 (m, 2H), 2.16 – 2.02 (m, 4H), 2.01 – 1.89 (m, 2H), 1.87 – 1.40 (m, 10H), 1.37 – 1.20 (m, 4H), 1.15 – 1.00 (m, 21H), 0.82 (s, 3H).

^{13}C NMR (101 MHz, CDCl$_3$) δ 154.7, 150.1, 142.6, 142.4, 135.0, 129.9, 128.3, 128.0, 126.4, 125.9, 125.4, 123.6, 121.8, 72.7, 57.9, 53.6, 53.0, 43.4, 42.6, 39.0, 38.7, 37.9, 32.6, 31.3, 30.5, 26.2, 23.2, 21.1, 18.6, 18.3, 14.4, 12.5.

HRMS (ESI): *m/z* Calc. for $C_{39}H_{56}NOSi^+$ $[M+H]^+$: 582.4126, found: 582.4131.

IR (film): 2941, 2865, 1543, 1463, 1436, 1379, 1102, 1068, 1013, 995, 883, 860, 795, 756, 678, 660 cm^{-1}.

$[\alpha]_D^{23}$ = − 46.2 (*c* = 0.2, $CHCl_3$).

Synthesis of 26

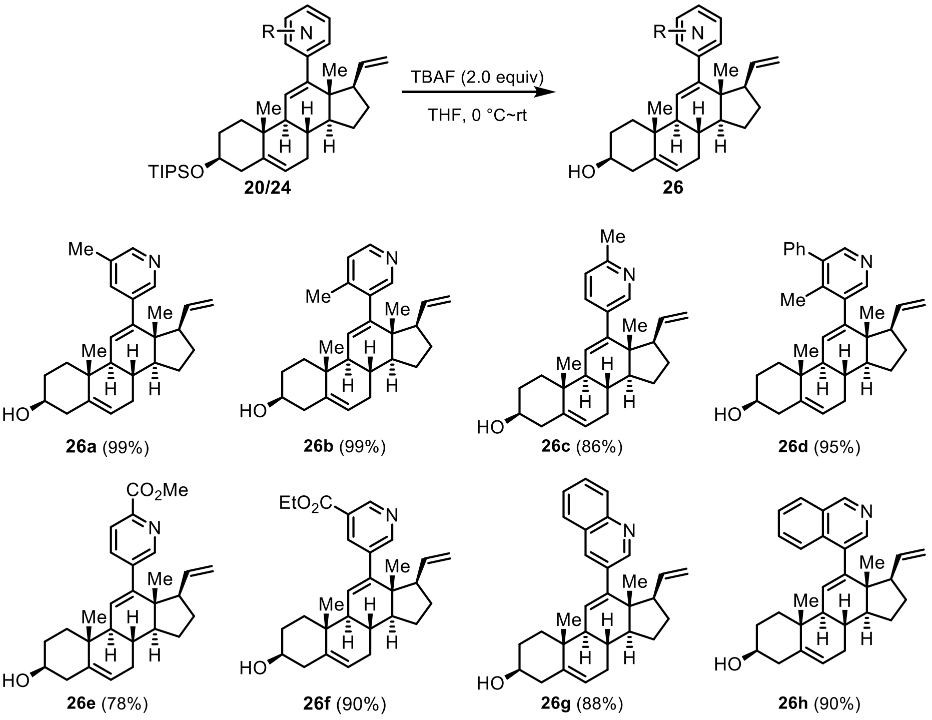

Synthesis of **26**: To a solution of compounds **20** or **24** in THF (1 mL) at 0 °C was added TBAF (1 M in THF, 2 equiv). After stirring at room temperature for 18 h, the reaction mixture was concentrated *in vacuo*. The resulting residue was purified by flash chromatography on silica gel using EtOAc in hexanes as eluent to give secondary alcohol **26**.

Compound **26b** (13.9 mg, 99%, 2:1 rotamers; SM: 19.4 mg, 35.5 μmol) as a white foam.
Purification eluent: EtOAc in hexanes (35%~45%).
^{1}H NMR (800 MHz, $CDCl_3$) δ 8.32 – 8.27 (m, 1H), 8.27 – 8.19 (m, 1H), 7.05 (d, *J* = 5.0 Hz, 1H), 5.47 (d, *J* = 5.4 Hz, 1H), 5.32 (s, 1H), 5.18 (dq, *J* = 19.5, 10.4, 9.7 Hz, 1H), 4.66 – 4.35 (m, 2H), 3.55 (tt, *J* = 10.5, 4.5 Hz, 1H), 2.69 – 2.14 (m, 6H), 2.11 – 1.57 (m, 10H), 1.57 – 1.46 (m, 2H), 1.41 – 0.81 (m, 8H).
^{13}C NMR (201 MHz, $CDCl_3$) δ 151.1, 149.6, 147.5, 147.3, 145.4, 145.0, 141.9, 138.9, 138.3, 138.0, 128.2, 127.7, 124.8, 122.3, 122.1, 115.4, 114.0, 72.0, 54.5, 54.3, 53.7, 53.2, 50.1, 49.7, 48.8, 42.4, 38.0, 37.7, 37.0, 31.5, 31.23, 31.17, 28.2, 23.5, 20.3, 20.2, 19.7, 17.8, 17.0.
HRMS (ESI): *m/z* Calc. for $C_{27}H_{36}NO^+$ $[M+H]^+$: 390.2791, found: 390.2788.
IR (film): 3291, 2959, 2927, 2870, 1592, 1457, 1437, 1375, 1216, 1079, 1057, 995, 911, 828, 807, 749, 664 cm^{-1}.
$[\alpha]_D^{22}$ = − 31.8 (*c* = 0.4, $CHCl_3$).

Compound **26c** (17.0 mg, 86%; SM: 27.8 mg, 50.9 μmol) as a white solid.
Purification eluent: EtOAc in hexanes (30%~40%).
^{1}H NMR (400 MHz, $CDCl_3$) δ 8.23 (d, *J* = 2.2 Hz, 1H), 7.31 (dd, *J* = 7.9, 2.3 Hz, 1H), 7.00 (d, *J* = 7.9 Hz, 1H), 5.45 (dt, *J* = 5.5, 1.9 Hz, 1H), 5.39 – 5.24 (m, 2H), 4.56 (dd, *J* = 10.3, 2.3 Hz, 1H), 4.49 (ddd, *J* = 17.1, 2.0, 1.1 Hz, 1H), 3.55 (tt, *J* = 11.0, 4.6 Hz, 1H), 2.52 (s, 3H), 2.45 – 2.30 (m, 2H), 2.19 (tq, *J* = 13.3, 2.6 Hz, 1H), 2.02 (ddt, *J* = 19.3, 5.6, 2.3 Hz, 1H), 1.97 – 1.64 (m, 9H), 1.53 (dtd, *J* = 14.3, 11.4, 10.3, 2.8 Hz, 2H), 1.40 – 1.26 (m, 1H), 1.17 (td, *J* = 14.5, 13.9, 4.1 Hz, 1H), 1.04 (s, 3H), 0.97 (s, 3H).

^{13}C NMR (101 MHz, $CDCl_3$) δ 156.2, 148.8, 147.1, 141.8, 139.4, 137.1, 135.5, 127.5, 122.1, 121.8, 115.2, 71.9, 54.4, 53.4, 50.5, 47.8, 42.4, 37.7, 36.9, 31.5, 31.2, 30.9, 28.6, 24.1, 23.4, 19.7, 17.1.

HRMS (ESI): *m/z* Calc. for $C_{27}H_{36}NO^+$ $[M+H]^+$: 390.2791, found: 390.2788.

IR (film): 3246, 2959, 2926, 2897, 2861, 1600, 1490, 1454, 1430, 1372, 1292, 1137, 1077, 1036, 991, 917, 833, 802, 751, 737, 687, 633 cm^{-1}.

$[\alpha]_D^{23}$ = − 53.2 (*c* = 0.5, $CHCl_3$).

Compound **26d** (14.6 mg, 95%, ~3:1 rotamers; SM: 20.4 mg, 32.8 μmol) as a white foam.

Purification eluent: EtOAc in hexanes (20%~35%).

^{1}H NMR (800 MHz, $CDCl_3$) δ 8.28 – 8.19 (m, 2H), 7.44 (t, *J* = 7.4 Hz, 2H), 7.38 (t, *J* = 7.4 Hz, 1H), 7.32 – 7.25 (m, 2H), 5.50 – 5.45 (m, 1H), 5.43 – 5.37 (m, 1H), 5.33 – 5.21 (m, 1H), 4.65 – 4.47 (m, 2H), 3.60 – 3.52 (m, 1H), 2.66 – 2.33 (m, 2H), 2.26 – 1.69 (m, 14H), 1.61 – 1.48 (m, 2H), 1.41 – 1.33 (m, *J* = 6.1 Hz, 1H), 1.19 (dt, *J* = 13.6, 6.9 Hz, 1H), 1.08 (s, 3H), 1.07 – 0.91 (m, 3H).

^{13}C NMR (201 MHz, $CDCl_3$) δ 149.9, 148.5, 147.9, 147.8, 145.3, 145.1, 143.2, 141.9, 141.6, 139.1, 138.85, 138.79, 138.2, 138.1, 137.9, 129.6, 129.4, 128.5, 128.4, 128.3, 127.8, 127.6, 122.3, 122.1, 115.4, 113.8, 72.0, 71.9, 54.7, 54.3, 53.8, 53.1, 50.1, 49.8, 49.0, 48.9, 42.45, 42.36, 38.0, 37.8, 37.1, 37.0, 31.54, 31.49, 31.23, 31.19, 31.1, 28.5, 28.3, 23.5, 20.2, 19.7, 18.7, 18.5, 17.9, 17.0.

HRMS (ESI): *m/z* Calc. for $C_{33}H_{40}NO^+$ $[M+H]^+$: 466.3104, found: 466.3104.

IR (film): 3296, 2959, 2927, 2871, 1456,1436, 1408, 1375, 1215, 1075, 1057, 1004, 992, 906, 749, 701, 665, 652 cm^{-1}.

$[\alpha]_D^{22}$ = − 39.3 (*c* = 0.4, $CHCl_3$).

Compound **26e** (16.3 mg, 78%; SM: 28.5 mg, 48.3 μmol) as a colorless oil.

Purification eluent: EtOAc in hexanes (40%~50%).

^{1}H NMR (400 MHz, $CDCl_3$) δ 8.49 (dd, *J* = 2.2, 0.8 Hz, 1H), 7.99 (dd, *J* = 7.9, 0.8 Hz, 1H), 7.57 (dd, *J* = 8.0, 2.2 Hz, 1H), 5.46 (dt, *J* = 5.6, 1.9 Hz, 1H), 5.41 (d, *J* = 1.6 Hz, 1H), 5.30 (ddd, *J* = 17.0, 10.3, 8.5 Hz, 1H), 4.50 (dd, *J* = 10.3, 1.9 Hz, 1H), 4.41 (ddd, *J* = 17.1, 1.9, 0.9 Hz, 1H), 4.00 (s, 3H), 3.55 (tt, *J* = 11.1, 4.5 Hz, 1H), 2.42 – 2.29 (m, 2H), 2.24 – 2.13 (m, 1H), 2.10 – 1.97 (m, 1H), 1.95 – 1.44 (m, 11H), 1.42 – 1.11 (m, 2H), 1.04 (s, 3H), 1.03 (s, 3H).

^{13}C NMR (101 MHz, $CDCl_3$) δ 166.0, 149.8, 146.5, 145.8, 142.0, 141.7, 139.0, 137.4, 128.6, 124.0, 122.1, 115.8, 71.9, 54.5, 53.5, 52.9, 50.8, 47.8, 42.4, 37.7, 36.9, 31.5, 31.1, 30.8, 28.9, 23.3, 19.8, 17.2.

HRMS (ESI): *m/z* Calc. for $C_{28}H_{36}NO_3^+$ $[M+H]^+$: 434.2690, found: 434.2687.

IR (film): 3354, 2927, 2872, 1727, 1436, 1309, 1236, 1133, 1057, 751, 715, 706 cm^{-1}.

$[\alpha]_D^{23}$ = − 35.3 (*c* = 0.5, $CHCl_3$).

Compound **26f** (23.2 mg, 90%; SM: 35.0 mg, 57.9 μmol) as a white solid.

Purification eluent: EtOAc in hexanes (20%~35%).

^{1}H NMR (400 MHz, $CDCl_3$) δ 9.05 (s, 1H), 8.50 (s, 1H), 8.01 (t, *J* = 2.0 Hz, 1H), 5.46 (dt, *J* = 5.6, 1.9 Hz, 1H), 5.40 (d, *J* = 1.6 Hz, 1H), 5.31 (ddd, *J* = 17.0, 10.3, 8.7 Hz, 1H), 4.50 (dd, *J* = 10.3, 1.9 Hz, 1H), 4.47 – 4.34 (m, 3H), 3.55 (tt, *J* = 11.0, 4.5 Hz, 1H), 2.47 – 2.30 (m, 2H), 2.19 (tq, *J* = 13.3, 2.5 Hz, 1H), 2.10 – 1.97 (m, 1H), 1.97 – 1.64 (m, 9H), 1.61 – 1.44 (m, 2H), 1.41 (t, *J* = 7.1 Hz, 3H), 1.33 (dq, *J* = 18.4, 6.6, 6.1 Hz, 1H), 1.18 (td, *J* = 14.4, 13.8, 4.2 Hz, 1H), 1.05 (s, 3H), 1.02 (s, 3H).

^{13}C NMR (101 MHz, $CDCl_3$) δ 165.7, 153.2, 148.8, 146.3, 141.7, 139.2, 138.0, 137.6, 128.6, 125.0, 122.1, 115.6, 71.9, 61.5, 54.4, 53.5, 50.8, 47.8, 42.4, 37.7, 36.9, 31.5, 31.1, 30.8, 29.1, 23.4, 19.8, 17.2, 14.4.

HRMS (ESI): *m/z* Calc. for $C_{29}H_{38}NO_3^+$ $[M+H]^+$: 448.2846, found: 448.2846.

IR (film): 3355, 2961, 2931, 2871, 1722, 1443, 1375, 1297, 1241, 1107, 1051, 1026, 912, 751, 716, 705, 666 cm^{-1}.

$[\alpha]_D^{23}$ = − 38.8 (*c* = 0.5, $CHCl_3$).

Compound **26g** (11.2 mg, 88%; SM: 17.3 mg, 29.7 µmol) as a white solid.
Purification eluent: EtOAc in hexanes (30%~40%).
^{1}H NMR (400 MHz, $CDCl_3$) δ 8.73 (d, *J* = 2.2 Hz, 1H), 8.07 (d, *J* = 8.4 Hz, 1H), 7.85 (d, *J* = 2.2 Hz, 1H), 7.76 (dd, *J* = 8.2, 1.5 Hz, 1H), 7.67 (ddd, *J* = 8.4, 6.8, 1.5 Hz, 1H), 7.52 (ddd, *J* = 8.1, 6.8, 1.2 Hz, 1H), 5.57 – 5.40 (m, 2H), 5.30 (ddd, *J* = 17.0, 10.3, 8.3 Hz, 1H), 4.52 – 4.30 (m, 2H), 3.57 (tt, *J* = 11.2, 4.6 Hz, 1H), 2.50 (q, *J* = 8.9 Hz, 1H), 2.37 (ddd, *J* = 12.9, 4.9, 2.2 Hz, 1H), 2.28 – 2.14 (m, 1H), 2.13 – 1.64 (m, 10H), 1.54 (dddd, *J* = 14.1, 11.8, 9.4, 3.0 Hz, 2H), 1.44 – 1.14 (m, 2H), 1.09 (s, 3H), 1.07 (s, 3H).
^{13}C NMR (101 MHz, $CDCl_3$) δ 151.8, 147.3, 146.9, 141.8, 139.2, 135.8, 135.2, 129.2, 129.0, 128.4, 127.8, 127.5, 126.7, 122.2, 115.4, 71.9, 54.5, 53.6, 50.7, 48.1, 42.4, 37.8, 37.0, 31.5, 31.2, 31.0, 28.8, 23.4, 19.8, 17.3.
HRMS (ESI): *m/z* Calc. for $C_{30}H_{36}NO^+$ $[M+H]^+$: 426.2791, found: 426.2786.
IR (film): 3269, 2958, 2925, 2897, 2853, 1492, 1430, 1370, 1069, 997, 926, 911, 846, 800, 788, 752, 683, 633 cm^{-1}.
$[\alpha]_D^{22}$ = − 44.8 (*c* = 0.2, $CHCl_3$).

Compound **26h** (14.7 mg, 90%, ~3:1 rotamers; SM: 22.3 mg, 38.3 µmol) as a light-yellow oil.
Purification eluent: EtOAc in hexanes (30%~40%).
^{1}H NMR (600 MHz, $CDCl_3$) δ 9.15 – 9.09 (m, 1H), 8.32 – 8.22 (m, 1H), 7.98 – 7.87 (m, 2H), 7.70 – 7.64 (m, 1H), 7.57 (ddt, *J* = 8.8, 6.8, 1.6 Hz, 1H), 5.56 – 5.44 (m, 2H), 5.00 – 4.90 (m, 1H), 4.56 – 3.37 (m, 3H), 2.73 – 2.17 (m, 3H), 2.14 – 0.92 (m, 20H).
^{13}C NMR (151 MHz, $CDCl_3$) δ 151.13, 151.07, 143.9, 143.8, 143.7, 142.04, 141.96, 141.9, 141.6, 138.9, 138.4, 136.4, 135.9, 133.5, 133.4, 130.0, 129.9, 129.4, 128.5, 128.4, 127.8, 126.9, 126.8, 126.0, 125.8, 122.4, 122.2, 115.3, 113.3, 72.03, 71.96, 54.7, 54.4, 53.7, 53.2, 50.1, 49.9, 48.9, 48.8, 42.5, 42.4, 38.0, 37.8, 36.98, 36.97, 31.54, 31.48, 31.32, 31.29, 31.25, 31.17, 28.4, 28.3, 23.7, 23.6, 20.3, 19.8, 18.1, 16.8.
HRMS (ESI): *m/z* Calc. for $C_{30}H_{36}NO^+$ $[M+H]^+$: 426.2791, found: 426.2788.
IR (film): 3318, 2927, 2869, 1456, 1437, 1390, 1375, 1217, 1079, 1056, 912, 749, 666 cm^{-1}.
$[\alpha]_D^{22}$ = − 30.5 (*c* = 0.4, $CHCl_3$).

Synthesis of 27

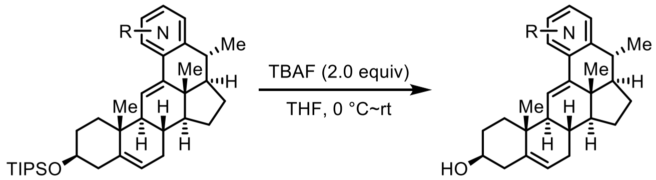

25 **27**

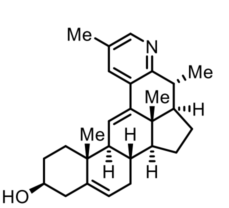

1 (91%)

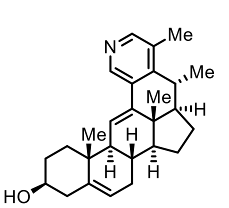

27a (81%)

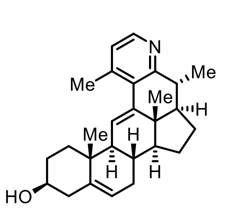

27b (87%)

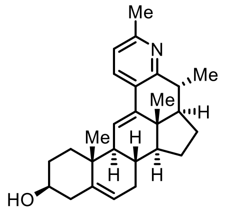

27ca (65%)

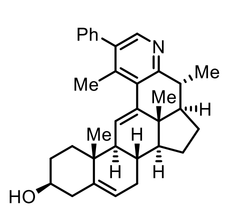

27d (92%)

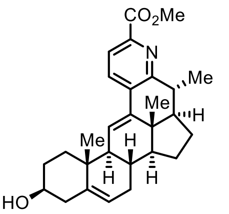

27ea (99%)

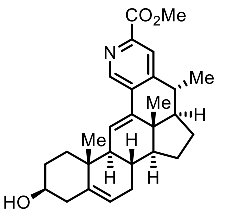

22eb (89%)

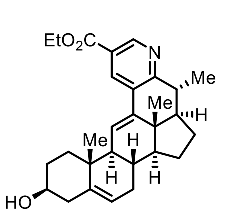

27f (99%)

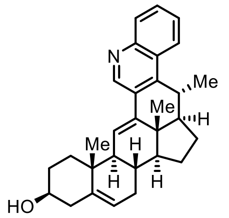

27gb (78%)

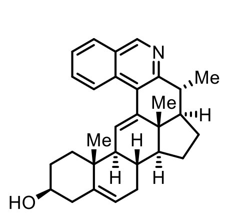

27h (99%)

Synthesis of **27**: To a solution of compounds **25** in THF (1.0 mL) at 0 °C was added TBAF (1 M in THF, 2 equiv). After stirring at room temperature for 18 h, the reaction mixture was concentrated *in vacuo*. The resulting residue was purified by flash chromatography on silica gel using EtOAc in hexanes as eluent to give secondary alcohol **27**.

Compound **27a** (12.9 mg, 81%; SM: 22.4 mg, 41.0 μmol) as a white foam.

Purification eluent: EtOAc in hexanes (50%~70%).

^{1}H NMR (400 MHz, $CDCl_3$) δ 8.69 (br s, 1H), 8.16 (br s, 1H), 6.19 (d, *J* = 2.8 Hz, 1H), 5.46 (dt, *J* = 5.6, 1.8 Hz, 1H), 3.59 (tt, *J* = 11.2, 4.7 Hz, 1H), 2.75 (dq, *J* = 10.2, 6.5 Hz, 1H), 2.45 – 2.15 (m, 6H), 2.15 – 2.00 (m, 4H), 1.96 (dp, *J* = 10.3, 3.6 Hz, 1H), 1.91 – 1.71 (m, 3H), 1.68 – 1.40 (m, 5H), 1.35 – 1.20 (m, 4H), 1.01 (s, 3H), 0.63 (s, 3H).

^{13}C NMR (101 MHz, $CDCl_3$) δ 149.6, 149.3, 144.2, 142.6, 142.1, 132.9, 128.7, 122.1, 116.6, 72.0, 56.3, 52.2, 52.0, 42.5, 41.3, 38.5, 37.4, 34.3, 31.7, 31.3, 31.0, 26.6, 23.4, 20.0, 19.4, 19.0, 14.3.

HRMS (ESI): *m/z* Calc. for $C_{27}H_{36}NO^+$ $[M+H]^+$: 390.2791, found: 390.2788.

IR (film): 2927, 2871, 1463, 1435, 1374, 1263, 1242, 1051, 980, 848, 807, 734, 702 cm^{-1}.

$[\alpha]_D^{22}$ = − 118.5 (*c* = 0.2, $CHCl_3$).

Compound **27b** (8.7 mg, 87%; SM: 14.0 mg, 25.6 μmol) as a white solid.

Purification eluent: EtOAc in hexanes (20%~30%).

^{1}H NMR (400 MHz, $CDCl_3$) δ 8.29 (d, *J* = 4.8 Hz, 1H), 6.97 (d, *J* = 4.8 Hz, 1H), 6.05 (d, *J* = 2.8 Hz, 1H), 5.48 (dt, *J* = 5.6, 1.9 Hz, 1H), 3.67 – 3.52 (m, 1H), 2.74 (dq, *J* = 13.0, 6.6 Hz, 1H), 2.55 (s, 3H), 2.39 (ddd, *J* = 12.8, 4.9, 2.2 Hz, 1H), 2.29 – 2.17 (m, 1H), 2.13 – 1.70 (m, 8H), 1.68 – 1.40 (m, 9H), 1.31 (td, *J* = 14.3, 13.7, 4.0 Hz, 1H), 1.12 (s, 3H), 0.70 (s, 3H).

^{13}C NMR (151 MHz, $CDCl_3$) δ 161.7, 146.0, 145.5, 143.2, 141.8, 128.9, 125.9, 125.3, 122.4, 72.0, 57.4, 53.1, 51.6, 42.6, 42.4, 39.2, 38.4, 37.6, 31.7, 31.3, 30.4, 26.3, 25.6, 23.3, 20.5, 18.9, 14.8.

HRMS (ESI): *m/z* Calc. for $C_{27}H_{36}NO^+$ $[M+H]^+$: 390.2791, found: 390.2789.

IR (film): 3347, 2957, 2926, 2867, 1448, 1376, 1077, 1056, 825, 808, 752 cm^{-1}.

$[\alpha]_D^{23}$ = − 231.3 (c = 0.2, $CHCl_3$).

Compound **27ca** (1.2 mg, 65%; SM: 2.6 mg, 4.76 μmol) as a colorless oil.
Purification eluent: EtOAc in hexanes (10%~20%).
^{1}H NMR (800 MHz, $CDCl_3$) δ 7.79 (d, J = 8.1 Hz, 1H), 6.94 (d, J = 8.1 Hz, 1H), 6.00 (s, 1H), 5.49 – 5.45 (m, 1H), 3.59 (td, J = 11.5, 5.3 Hz, 1H), 2.76 (dt, J = 16.8, 6.5 Hz, 1H), 2.52 (s, 3H), 2.37 (dq, J = 12.9, 2.4 Hz, 1H), 2.24 – 2.18 (m, 1H), 2.12 – 1.99 (m, 4H), 1.97 – 1.87 (m, 2H), 1.83 – 1.71 (m, 2H), 1.64 – 1.42 (m, 6H), 1.40 (d, J = 4.5 Hz, 3H), 1.30 (td, J = 13.7, 6.8 Hz, 1H), 1.04 (s, 3H), 0.74 (s, 3H).
^{13}C NMR (201 MHz, $CDCl_3$) δ 159.7, 156.3, 142.7, 141.9, 131.4, 125.5, 122.4, 120.7, 116.4, 72.1, 56.1, 51.7, 51.6, 42.5, 41.7, 38.4, 38.3, 37.4, 31.6, 31.4, 31.3, 26.1, 24.6, 23.8, 20.0, 18.3, 15.2.
HRMS (ESI): m/z Calc. for $C_{27}H_{36}NO^+$ $[M+H]^+$: 390.2791, found: 390.2789.
IR (film): 3371, 2923, 2852, 1462, 1438, 1376, 1263, 1241, 1076, 1053, 824, 808, 737cm^{-1}.
$[\alpha]_D^{23}$ = − 123.9 (c = 0.02, $CHCl_3$).

Compound **27d** (8.4 mg, 92%; SM: 12.2 mg, 19.6 μmol) as a white foam.
Purification eluent: EtOAc in hexanes (10%~20%).
^{1}H NMR (400 MHz, $CDCl_3$) δ 8.30 (s, 1H), 7.47 – 7.40 (m, 2H), 7.40 – 7.35 (m, 1H), 7.34 – 7.29 (m, 2H), 6.10 (d, J = 2.8 Hz, 1H), 5.48 (dt, J = 5.6, 1.9 Hz, 1H), 3.58 (tt, J = 11.0, 4.5 Hz, 1H), 2.78 (dq, J = 13.0, 6.6 Hz, 1H), 2.44 (s, 3H), 2.38 (ddd, J = 12.9, 5.0, 2.6 Hz, 1H), 2.27 – 2.17 (m, 1H), 2.13 – 2.02 (m, 3H), 2.00 – 1.71 (m, 5H), 1.69 – 1.41 (m, 9H), 1.37 – 1.26 (m, 1H), 1.11 (s, 3H), 0.75 (s, 3H).
^{13}C NMR (151 MHz, $CDCl_3$) δ 160.4, 145.9, 143.9, 142.9, 141.8, 139.2, 137.5, 129.8, 129.6, 128.5, 127.6, 127.0, 122.4, 71.9, 57.5, 53.4, 51.9, 42.6, 42.5, 39.1, 38.4, 37.6, 31.7, 31.2, 30.3, 26.3, 23.3, 23.2, 20.6, 18.8, 14.7.
HRMS (ESI): m/z Calc. for $C_{33}H_{40}NO^+$ $[M+H]^+$: 466.3104, found: 466.3105.
IR (film): 3347, 2926, 2868, 1448, 1432, 1375, 1264, 1076, 1055, 1008, 764, 736, 701, 678 cm^{-1}.
$[\alpha]_D^{23}$ = − 224.8 (c = 0.2, $CHCl_3$).

Compound **27ea** (1.8 mg, 99%; SM: 2.4 mg, 4.07 μmol) as a white solid.
Purification eluent: EtOAc in hexanes (20%~30%).
^{1}H NMR (600 MHz, $CDCl_3$) δ 8.02 (d, J = 8.2 Hz, 1H), 7.91 (d, J = 8.3 Hz, 1H), 6.24 (d, J = 2.9 Hz, 1H), 5.48 (d, J = 5.4 Hz, 1H), 3.99 (s, 3H), 3.59 (td, J = 11.0, 5.4 Hz, 1H), 2.92 (dq, J = 13.0, 6.6 Hz, 1H), 2.38 (ddd, J = 12.6, 4.8, 2.2 Hz, 1H), 2.24 – 2.18 (m, 1H), 2.14 – 1.89 (m, 6H), 1.86 – 1.73 (m, 2H), 1.66 – 1.42 (m, 9H), 1.32 (td, J = 13.5, 3.5 Hz, 1H), 1.05 (s, 3H), 0.75 (s, 3H).
^{13}C NMR (151 MHz, $CDCl_3$) δ 166.2, 160.9, 145.4, 142.2, 141.8, 132.1, 131.8, 122.6, 122.3, 121.0, 72.0, 56.4, 52.9, 51.7, 51.3, 42.4, 41.6, 38.6, 38.5, 37.4, 31.6, 31.3, 31.2, 26.0, 23.8, 20.2, 18.3, 15.3.
HRMS (ESI): m/z Calc. for $C_{28}H_{36}NO_3^+$ $[M+H]^+$: 434.2690, found: 434.2689.
IR (film): 3369, 2924, 2852, 1722, 1431, 1376, 1311, 1263, 1239, 1194, 1138, 1076, 1053, 980, 849, 839, 806, 798, 756, 735 cm^{-1}.
$[\alpha]_D^{23}$ = − 104.5 (c = 0.05, $CHCl_3$).

Compound **27eb** (3.4 mg, 89%; SM: 5.2 mg, 8.82 μmol) as a white solid.
Purification eluent: EtOAc in hexanes (50%~70%~100%).
^{1}H NMR (800 MHz, $CDCl_3$) δ 8.94 (s, 1H), 8.12 (s, 1H), 6.33 (d, J = 2.9 Hz, 1H), 5.48 (dt, J = 4.0, 2.0 Hz, 1H), 4.00 (s, 3H), 3.63 – 3.57 (m, 1H), 2.73 (dq, J = 13.5, 6.9 Hz, 1H), 2.38 (ddd, J = 13.0, 4.8, 2.3 Hz, 1H), 2.24 – 2.19 (m, 1H), 2.13

– 2.00 (m, 4H), 1.99 – 1.90 (m, 2H), 1.85 – 1.80 (m, 1H), 1.76 (ddt, *J* = 16.3, 11.0, 2.4 Hz, 1H), 1.66 – 1.29 (m, 10H), 1.06 (s, 3H), 0.71 (s, 3H).
^{13}C NMR (201 MHz, CDCl$_3$) δ 166.4, 150.8, 146.1, 145.5, 141.9, 141.5, 132.3, 124.5, 122.2, 120.8, 72.0, 56.4, 52.9, 51.6, 50.7, 42.4, 41.8, 38.5, 37.4, 34.8, 31.6, 31.3, 31.2, 25.9, 23.7, 20.2, 18.2, 15.2.
HRMS (ESI): *m/z* Calc. for $C_{28}H_{36}NO_3^+$ $[M+H]^+$: 434.2690, found: 434.2690.
IR (film): 3379, 2957, 2924, 2854, 1721, 1456, 1436, 1284, 1248, 1108, 1077, 1057, 1043, 979, 807, 793, 734, 701 cm^{-1}.
$[\alpha]_D^{23}$ = −103.9 (*c* = 0.1, CHCl$_3$).

Compound **27f** (9.2 mg, 99%; SM: 12.0 mg, 19.9 μmol) as a colorless oil.
Purification eluent: EtOAc in hexanes (20%~35%).
^{1}H NMR (600 MHz, CDCl$_3$) δ 9.03 (d, *J* = 2.1 Hz, 1H), 8.47 (d, *J* = 2.1 Hz, 1H), 6.20 (d, *J* = 2.9 Hz, 1H), 5.47 (dt, *J* = 5.7, 1.9 Hz, 1H), 4.42 (q, *J* = 7.1 Hz, 2H), 3.60 (tt, *J* = 11.3, 4.7 Hz, 1H), 2.85 (dq, *J* = 13.2, 6.7 Hz, 1H), 2.38 (ddd, *J* = 12.8, 4.9, 2.4 Hz, 1H), 2.25 – 2.18 (m, 1H), 2.15 (dt, *J* = 13.3, 3.6 Hz, 1H), 2.10 – 2.00 (m, 3H), 1.97 (dt, *J* = 13.4, 3.5 Hz, 1H), 1.92 (td, *J* = 10.6, 4.3 Hz, 1H), 1.88 – 1.79 (m, 1H), 1.76 (dddd, *J* = 16.5, 11.0, 3.1, 1.8 Hz, 1H), 1.68 – 1.38 (m, 12H), 1.31 (td, *J* = 13.7, 4.1 Hz, 1H), 1.05 (s, 3H), 0.74 (s, 3H).
^{13}C NMR (151 MHz, CDCl$_3$) δ 165.9, 164.7, 148.1, 141.9, 141.8, 132.3, 128.7, 124.0, 122.2, 119.4, 72.0, 61.4, 56.3, 51.7, 51.0, 42.5, 41.7, 38.8, 38.4, 37.4, 31.6, 31.3, 31.2, 26.0, 23.8, 20.1, 17.9, 15.3, 14.5.
HRMS (ESI): *m/z* Calc. for $C_{29}H_{38}NO_3^+$ $[M+H]^+$: 448.2846, found: 448.2846.
IR (film): 3368, 2927, 2869, 1720, 1462, 1396, 1368, 1299, 1249, 1231, 1110, 1052, 1024, 807, 770, 735, 703 cm^{-1}.
$[\alpha]_D^{23}$ = − 118.3 (*c* = 0.2, CHCl$_3$).

Compound **27gb** (5.2 mg, 78%; SM: 9.1 mg, 15.6 μmol) as a white solid.
Purification eluent: EtOAc in hexanes (40%~50%).
^{1}H NMR (400 MHz, CDCl$_3$) δ 9.16 (s, 1H), 8.06 (t, *J* = 8.2 Hz, 2H), 7.61 (ddd, *J* = 8.3, 6.8, 1.4 Hz, 1H), 7.50 (ddd, *J* = 8.2, 6.8, 1.3 Hz, 1H), 6.35 (d, *J* = 2.9 Hz, 1H), 5.49 (dt, *J* = 5.7, 1.9 Hz, 1H), 3.61 (tt, *J* = 11.1, 4.6 Hz, 1H), 3.30 (dq, *J* = 9.8, 6.6 Hz, 1H), 2.39 (ddd, *J* = 12.8, 4.9, 2.3 Hz, 1H), 2.28 – 1.49 (m, 15H), 1.45 – 1.19 (m, 4H), 1.03 (s, 3H), 0.70 (s, 3H).
^{13}C NMR (101 MHz, CDCl$_3$) δ 147.9, 147.6, 146.4, 143.0, 142.0, 130.1, 128.1, 128.0, 125.8, 125.65, 125.55, 122.2, 117.5, 72.0, 56.6, 52.1, 51.9, 42.5, 41.4, 38.5, 37.4, 33.4, 31.7, 31.4, 31.2, 26.5, 23.6, 22.7, 20.1, 14.5.
HRMS (ESI): *m/z* Calc. for $C_{30}H_{36}NO^+$ $[M+H]^+$: 426.2791, found: 426.2788.
IR (film): 3367, 2922, 2852, 1550, 1498, 1463, 1376, 1356, 1077, 1030, 981, 954, 844, 806, 761, 735, 645, 633 cm^{-1}.
$[\alpha]_D^{23}$ = − 156.6 (*c* = 0.1, CHCl$_3$).

Compound **27h** (5.3 mg, 99%; SM: 7.2 mg, 12.4 μmol) as a white solid.
Purification eluent: EtOAc in hexanes (20%~30%).
^{1}H NMR (600 MHz, CDCl$_3$) δ 9.07 (s, 1H), 8.55 (d, *J* = 8.7 Hz, 1H), 7.94 (dd, *J* = 8.1, 1.3 Hz, 1H), 7.69 (ddd, *J* = 8.4, 6.7, 1.4 Hz, 1H), 7.53 (t, *J* = 7.4 Hz, 1H), 6.39 (d, *J* = 2.8 Hz, 1H), 5.51 (dt, *J* = 5.6, 1.9 Hz, 1H), 3.61 (tt, *J* = 11.3, 4.6 Hz, 1H), 2.85 (dq, *J* = 13.2, 6.7 Hz, 1H), 2.42 (ddd, *J* = 12.9, 4.9, 2.4 Hz, 1H), 2.32 – 2.24 (m, 1H), 2.15 – 2.06 (m, 3H), 2.03 – 1.91 (m, 2H), 1.88 – 1.45 (m, 12H), 1.35 (td, *J* = 13.6, 3.8 Hz, 1H), 1.29 (s, 3H), 0.82 (s, 3H).
^{13}C NMR (151 MHz, CDCl$_3$) δ 154.5, 150.0, 142.5, 141.7, 135.0, 130.1, 128.4, 128.0, 126.3, 126.0, 125.4, 123.7, 122.5, 72.0, 57.8, 53.5, 53.0, 42.59, 42.57, 38.9, 38.6, 37.7, 31.8, 31.2, 30.5, 26.2, 23.1, 21.0, 18.6, 14.4.
HRMS (ESI): *m/z* Calc. for $C_{30}H_{36}NO^+$ $[M+H]^+$: 426.2791, found: 426.2788.
IR (film): 3361, 2924, 2854, 1462, 1374, 1263, 1247, 1078, 1052, 853, 807, 795, 758, 736, 673, 633 cm^{-1}.
$[\alpha]_D^{23}$ = − 91.0 (*c* = 0.1, CHCl$_3$).

4. Data Comparison of Synthetic and Natural Product

Table S2. ^{1}H NMR Data Comparison of Synthetic, and Natural (Lai & Luo's group) Veragranine A (**1**) [C_6D_5N (pyridine-*d*$_5$) (ppm, Hz, Amount of Proton)]

entry	**Natural 1**[4]	**Synthetic 1**
1	8.53 (s, 1H)	8.52 (d, *J* = 2.1 Hz, 1H)
2	8.06 (s, 1H)	8.06 (d, *J* = 2.2 Hz, 1H)
3		6.31 (s, 1H, OH)
4	6.26 (d, *J* = 2.4, 1H)	6.26 (d, *J* = 2.8 Hz, 1H)
5	5.53 (d, *J* = 5.2, 1H)	5.53 (d, *J* = 5.5 Hz, 1H)
6	3.92 (m, 1H)	3.93 (tt, *J* = 10.9, 4.6 Hz, 1H)
7	2.88 (dq, *J* = 12.9, 6.4, 1H)	2.87 (dt, *J* = 13.2, 6.7 Hz, 1H)
8	2.73 (dd, *J* = 12.8, 2.9, 1H)	2.72 (ddd, *J* = 12.9, 4.9, 2.2 Hz, 1H)
9	2.59 (t, *J* = 11.3, 1H)	2.64 – 2.54 (m, 1H)
10	2.27 (s, 3H)	2.27 (s, 3H)
11	2.18, (d, *J* = 11.8, 1H)	2.17 (d, *J* = 12.3 Hz, 1H)
12	2.07 (overlap, 1H); 2.06 (overlap, 1H); 2.01 (d, *J* = 16.4, 1H)	2.12 – 1.97 (m, 3H)
13	1.94 (m, 1H); 1.90 (m, 1H); 1.87 (m, 1H)	1.97 – 1.82 (m, 3H)
14	1.78 (d, *J* = 13.9, 1H)	1.78 (d, *J* = 13.8 Hz, 1H)
15	1.72 (m, 1H)	1.75 – 1.67 (m, 1H)
16	1.62, (d, *J* = 6.4, 3H)	1.62 (d, *J* = 6.7 Hz, 3H)
17	1.50 (m, 1H); 1.49 (m, 1H); 1.43 (overlap, 1H); 1.42 (m, , 1H)	1.55 – 1.39 (m, 4H)
18	1.34 (m, 1H)	1.34 (td, *J* = 13.7, 3.8 Hz, 1H)
19	1.08 (s, 3H)	1.08 (s, 3H)
20	0.74 (s, 3H)	0.76 (s, 3H)

Table S3. ^{13}C NMR Data Comparison of Synthetic and Reported Veragranine A (**1**) [C_6D_5N (pyridine-d_5), ppm]

entry	**Lai & Luo's group**[4]	**Our Synthetic 1**	**Δ(Nat-Syn)**
1	158.1	158.2	–0.1
2	149.0	149.0	0
3	143.4	143.5	–0.1
4	143.0	143.1	–0.1
5	132.3	132.3	0
6	131.0	131.0	0
7	128.7	128.8	–0.1
8	122.1	122.2	–0.1
9	118.8	118.8	0
10	71.9	72.0	–0.1
11	56.9	57.0	–0.1
12	52.5 (52.3[a])	52.3	0
13	52.3 (52.2[a])	52.2	0
14	44.0	44.0	0
15	42.3	42.4	–0.1
16	39.1	39.2	–0.1
17	38.8	38.8	0
18	31.8 (38.1[a])	38.1	0
19	32.9	33.0	–0.1
20	31.9	32.0	–0.1
21	31.8	31.8	0
22	26.5	26.6	–0.1
23	24.4	24.4	0
24	20.5	20.5	0
25	18.8	18.8	0
26	18.2	18.3	–0.1
27	15.8	15.9	–0.1

[a]These chemical shifts are from ^{13}C NMR spectrum in Supporting Information of Lai & Luo's paper, and other data are from Table 1 in their article.

5. X-ray Structure and Analysis Data of 15, 26a and 1

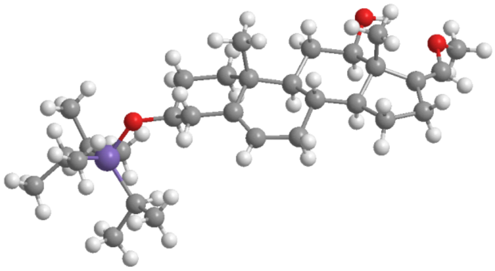

X-ray Structure of **15**

Solid Structure of **15**. A colorless plate shaped crystal of **15** for X-ray diffraction was obtained by slow evaporation of a hexanes/ethyl acetate solution of **15**. The data were collected at 150(2) K on a Bruker AXS D8 Quest CMOS diffractometer with Cu sealed tube and curved triumph monochromator with a 10 cm x 10 cm Photon-100 detector and fixed chi angle. The supplementary crystallographic data was deposited in The Cambridge Crystallographic Data Centre (CCDC 2333187).

X-ray analysis data:

Bond precision: C-C = 0.0059 A Wavelength=1.54178
Cell: a=16.1392(7) b=9.2959(4) c=20.9255(8)
alpha=90 beta=112.253(2) gamma=90
Temperature: 150 K Calculated Reported
Volume 2905.6(2) 2905.6(2)
Space group P 21 P 21
Hall group P 2yb P 2yb
Moiety formula C30 H52 O3 Si ?
Sum formula C30 H52 O3 Si C30 H52 O3 Si
Mr 488.81 488.80
Dx,g cm-3 1.117 1.117
Z 4 4
Mu (mm-1) 0.910 0.910
F000 1080.0 1080.0
F000' 1083.66
h,k,lmax 20,11,26 19,11,26
Nref 12654[6726] 11956
Tmin,Tmax 0.868,0.973 0.603,0.754
Tmin' 0.606
Correction method= # Reported T Limits: Tmin=0.603
Tmax=0.754 AbsCorr = MULTI-SCAN
Data completeness= 1.78/0.94 Theta(max)= 79.672
R(reflections)= 0.0500(9519) wR2(reflections)= 0.1281(11956)
S = 1.053 Npar= 633

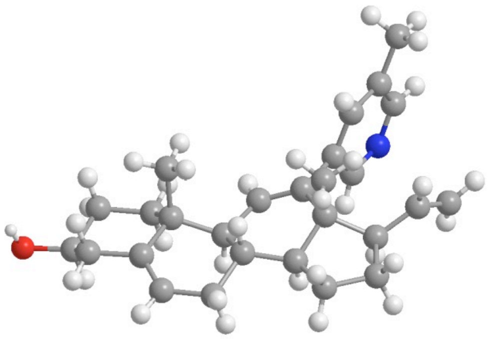

X-ray Structure of **26a**

Solid Structure of **26a**. A light-yellow rod-shaped crystal of **26a** for X-ray diffraction was obtained by slow evaporation of a hexanes/ethyl acetate solution of **26a**. The data were collected at 150(2) K on a Bruker AXS D8 Quest CMOS diffractometer with Cu sealed tube and curved triumph monochromator with a 10 cm x 10 cm Photon-100 detector and fixed chi angle. The supplementary crystallographic data was deposited in The Cambridge Crystallographic Data Centre (CCDC 2333188).

X-ray analysis data:

Bond precision: C-C = 0.0000 A Wavelength=1.54178
Cell: a=8.343(3) b=27.998(7) c=9.612(3)
alpha=90 beta=90.207(15) gamma=90
Temperature: 150 K Calculated Reported
Volume 2245.2(12) 2245.2(11)
Space group P 21 P 21
Hall group P 2yb P 2yb
Moiety formula C27 H35 N O ?
Sum formula C27 H35 N O C27 H35 N O
Mr 389.56 389.56
Dx,g cm-3 1.153 1.152
Z 4 4
Mu (mm-1) 0.522 0.522
F000 848.0 848.0
F000' 850.16
h,k,lmax 10,35,12 10,35,12
Nref 9776[4992] 7850
Tmin,Tmax 0.855,0.910 0.513,0.754
Tmin' 0.855
Correction method= # Reported T Limits: Tmin=0.513
Tmax=0.754 AbsCorr = MULTI-SCAN
Data completeness= 1.52/0.78 Theta(max)= 79.364
R(reflections)= 0.0673(7118) wR2(reflections)= 0.1753(7580)
S = 1.058 Npar= 713

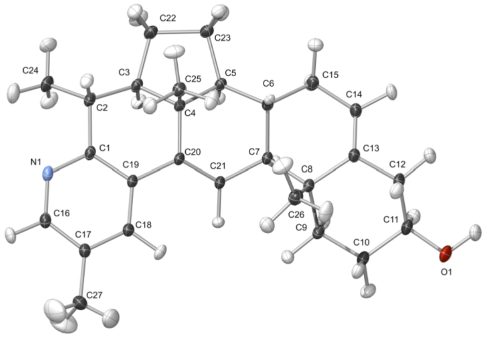

X-ray Structure of Veragranine A **(1)**

Solid Structure of Veragranine A **(1)**. A colorless prism shaped crystal of **1** for X-ray diffraction was obtained by slow evaporation of a $CHCl_3$/Methanol solution of **1**. The data were collected at 173 K on a Bruker APEX II CCD diffractometer with Cu sealed tube and curved triumph monochromator with a 10 cm x 10 cm Photon-100 detector and fixed chi angle. The supplementary crystallographic data was deposited in The Cambridge Crystallographic Data Centre (CCDC 2333189).

X-ray analysis data:

Bond precision: C-C = 0.0014 A Wavelength=1.54184

Cell: a=6.8163(1) b=12.3469(2) c=25.3648(4)

alpha=90 beta=90 gamma=90

Temperature: 173 K Calculated Reported

Volume 2134.71(6) 2134.71(6)

Space group P 21 21 21 P 21 21 21

Hall group P 2ac 2ab P 2ac 2ab

Moiety formula C27 H35 N O C27 H35 N O

Sum formula C27 H35 N O C27 H35 N O

Mr 389.56 389.58

Dx,g cm-3 1.212 1.212

Z 4 4

Mu (mm-1) 0.549 0.549

F000 848.0 850.3

F000' 850.16

h,k,lmax 8,15,31 8,15,31

Nref 4212[2435] 4165

Tmin,Tmax 0.833,0.886 0.428,1.000

Tmin' 0.800

Correction method= # Reported T Limits: Tmin=0.428

Tmax=1.000 AbsCorr = GAUSSIAN

Data completeness= 1.71/0.99 Theta(max)= 72.020

R(reflections)= 0.0245(4026) wR2(reflections)= 0.0553(4165)

S = 1.081 Npar= 578

6. Methods and results for biological evaluations:

Animals and housing:

Rats: Male and female pathogen-free Sprague Dawley rats (Charles River Laboratories, Wilmington MA) were used for the MIA experiments. All animals were pair-housed in transparent IVC cages (37*25*18 cm), in light- (12-h light: 12-h dark cycle; lights on at 07:00 h) and temperature-controlled (23 ± 3 °C) rooms. Each cage was provided with environmental enrichment consisting of paper-wool shavings (Enviropack) for nesting material, Nylabones, and cardboard tube for hiding, and cages were changed twice a week, but never on the days of testing. Food (Purina lab Diet 5053) and water were available ad libitum with water changed on a weekly basis. The animals were 6 weeks old upon arrival from the vendor and were left to acclimatized to the surroundings for at least 1 week before the start of behavioral experiments.

All animal use was conducted in accordance with the National Institutes of Health guidelines, and the study was conducted in strict accordance with recommendations in the Guide for the Care and Use of Laboratory Animals of the New York University (Protocol#: PROTO202100104). All animals were housed and bred at the New York University Kriser Dental Center Animal Facility. All efforts were made to minimize animal suffering.

Preparation of dissociated dorsal root ganglion neurons. Female Sprague-Dawley rats (~75-100g) were euthanized according to institutionally approved procedures. Dorsal root ganglia from all levels were dissociated as described previously.[5] Briefly, DRG were collected, trimmed at their roots, and enzymatically digested in DMEM (Cat#11965, Thermo Fisher Scientific) media with neutral protease (3.125 mg/mL, Cat# LS02104, Worthington, Lakewood, NJ) and collagenase type I (5 mg/mL, Cat# LS004194, Worthington, Lakewood, NJ) for 60 min at 37 ºC under gentle agitation. The dissociated DRG neurons were gently centrifuged to collect cells and resuspended in complete DRG media (DMEM containing 1% penicillin/streptomycin sulfate from 10,000 µg/mL stock, 30 ng/mL of nerve growth factor, and 10% fetal bovine serum (Hyclone)). Cells were seeded on poly-D-lysine-coated coverslips.

Calcium imaging. Changes in depolarization-induced calcium influx in rat DRG neurons were determined by loading neurons with 3 µM Fura-2AM for 30 minutes at 37 °C (Cat# F1221; Thermo Fisher Scientific, Waltham, MA, stock solution prepared at 1 mM in DMSO, 0.02% pluronic acid, Cat# P-3000MP; Life 250 Technologies, Carlsbad, CA) as previously described.[6] DRG neurons were incubated overnight (20 µM or 10 µM), or 30 min (10 µM) with veragrainine A analogs. A standard bath solution containing 139 mM NaCl, 3 mM KCl, 0.8 mM MgCl2, 252 1.8 mM CaCl2, 10 mM Na-HEPES, 5 mM glucose, pH 7.4, was used. Depolarization was evoked with a 15 sec pulse of 40 or 90 mM KCl. The T-type channel selective blocker TTA-P2 (1 µM) was used as a positive control in the stimulus with 40 mM KCl, and the N-type channel selective blocker ω-conotoxin-GVIA (500 nM) was used as a positive control in the stimulus with 90 mM KCl. For the veratridine depolarization experiments, 30 µM veratridine was used as the stimulus in the same standard bath solution. Cells were first examined under bright field to exclude those showing signs of lysis. Fluorescence imaging was achieved with an inverted microscope, Nikon EclipseTi-U (Nikon Instruments Inc., Melville, NY), using objective Nikon Fluor 4X and a Photometrics cooled CCD camera CoolSNAPES2 (Roper Scientific, Tucson, AZ) controlled by NIS Elements software (version 4.20, Nikon Instruments). The excitation light was delivered by a Lambda-LS system (Sutter Instruments, Novato, CA). The excitation filters (340 ± 5 nm and 380 ± 7 nm) were controlled by a Lambda 10 to 2 optical filter change (Sutter Instruments, Novato, CA). Fluorescence was recorded through a 505-nm dichroic mirror at 535 ± 25 nm. Images were taken every ~10 seconds during the time course of the experiment to minimize photobleaching and phototoxicity and to provide acceptable image quality. Changes in $[Ca^{2+}]c$ were monitored following a ratio of F_{340}/F_{380}, calculated after subtracting the background from both channels.

MIA induction. Induction of the Monoiodoacetate Arthritis Model to the knee joint (MIA) was performed using similar techniques to the previously reported one.[7] On the day of injection, the monoiodoacetate solution was freshly prepared in

sterile saline for injection of 2mg Sodium Iodoacetate Bioultra (Sigma, I9148-5G,) in 50µl saline. Animals were anaesthetized in an induction chamber using Isoflurane 2.5% mixed in O_2 at a flowrate of 1.5 L/min, and maintained via facemask at 2.0% during the injection. Anaesthetic depth was confirmed by lack of withdrawal reflex to a pinch to the tail. The animal was then placed on the back in dorsal recumbency, and the fur was shaved in the area around the knee on the left hindleg. The knee was stabilized and fixed in a slightly bend position and the patellar tendon was visualized as a white line below the skin. Using a 30G insulin-syringe (BD Micro-Fine Plus Demi, 0.3ml (30G)) the injection was made intraarticularly in the joint-space by applying it perpendicularly through the skin and tendon just below the patella.

Mechanical allodynia, vF: Low intensity mechanical sensitivity was assessed by using a series of calibrated von Frey monofilaments (North Coast Medical, Inc., Morgan Hill), similar to previous studies.[8] Animals were placed in individual Plexiglas enclosures on an elevated wire grid. They were given ~15min to acclimate to the enclosure and the experimenter's presence and movements below the grid, prior to stimulation of the plantar surface of the hind paw with a series of calibrated von Frey filaments. To initiate testing a filament with a medium bending force (4.0g) was first applied to the hind paw with uniform pressure for 5 seconds. A brisk withdrawal was considered a positive response whereupon the next lower filament in the series was applied. In the absence of a positive response the neighboring higher filament was applied. After the first change in response-pattern, indicating the threshold, 4 additional applications were performed; when no response, the next filament with a higher force was tested, and when positive response, the next lower force filament was tested. The 50% threshold was determined by the following equation: 50% threshold (g) = $10^{\log(\text{last filament})+k*\delta}$. The constant, *k*, was found in the table by Dixon,[9] and determined by the response-pattern.

Cold allodynia, ADT): Cold allodynia was assessed based on previously published protocols.[10] While the animals were still in the Plexiglas chambers following von Frey measurements, cold allodynia was assessed using application of a drop of acetone (Acetone Drop Test, ADT) to the plantar surface of the paw using a plastic syringe without mechanically touching the skin. Following application, the duration of the response was then recorded, with a maximum of 30 seconds. Pain-like behaviors was considered as flinching, licking, stepping, guarding, and/or looking at the paw. The application and assessment were performed two times per animal with 5-10 minutes between each application, and the average of the two measurements was calculated.

Data analysis and statistics

Animals were randomly allocated to treatment, and the experimenter was always blinded to the treatment provided. Statistical analysis was performed using GraphPad Prism version 9 (GraphPad Software, Inc., La Jolla, CA, USA). Full analysis results are displayed in the supplementary table S3. $P < 0.05$ was considered statistically significant. Error bars in the graphs represents mean ± SEM. For behavioural time-course experiments, the data was also transformed into Area Under the Curve (AUC) for the period tested for each individual animal, in order to simplify the treatment comparison.

Table S4. Statistical analysis

Fig	Analysis	Outcome	Post-test
**Fig 3. Administration of 27a and 27eb decreases mechanical and cold allodynia in rats with osteoarthritic pain**			
Fig 3C. Mechanical allodynia - AUC	Two-way ANOVA, sex*treatment	$F_{sex}(1, 18)=4.131$, P=0.0571 (NS)<br>$F_{treatment}(2,18)=5.992$, P=0.0101<br>$F_{sex*treatment}(2,18)=0.27$, P=0.76 (NS)	Tukey<br>N=4
Fig 3E. cold allodynia - AUC	Two-way ANOVA, sex*treatment	$F_{sex}(1, 18)=72.85$, P<0.0001<br>$F_{treatment}(2,18)=15.15$, P=0.0001<br>$F_{sex*treatment}(2,18)=0.79$, P=0.47 (NS)	Tukey<br>N=4

7. References

(1) Giannis, A.; Heretsch, P.; Sarli, V.; Stößel, A. Synthesis of Cyclopamine Using a Biomimetic and Diastereoselective Approach. *Angew. Chem. Int. Ed.* **2009**, *48*, 7911−7914.

(2) (a) Nakayama, Y.; Maser, M. R.; Okita, T.; Dubrovskiy, A. V.; Campbell, T. L.; Reisman, S. E. Total Synthesis of Ritterazine B. *J. Am. Chem. Soc.* **2021**, *143*, 4187−4192. (b) See, Y. Y.; Herrmann, A. T.; Aihara, Y.; Baran, P. S. Scalable C−H Oxidation with Copper: Synthesis of Polyoxypregnanes. *J. Am. Chem. Soc.* **2015**, *137*, 13776−13779. (c) Trammell, R.; See, Y. Y.; Herrmann, A. T.; Xie, N.; Díaz, D. E.; Siegler, M. A.; Baran, P. S.; Garcia-Bosch, I. Decoding the Mechanism of Intramolecular Cu-Directed Hydroxylation of sp^3 C−H Bonds. *J. Org. Chem.* **2017**, *82*, 7887−7904.

(3) Patin, A.; Kanazawa, A.; Philouze, C.; Greene, A. E.; Muri, E.; Barreiro, E.; Costa, P. C. C. Highly Stereocontrolled Synthesis of Natural Barbacenic Acid, Novel Bisnorditerpene from *Barbacenia flava*. *J. Org. Chem.* **2003**, *68*, 3831−3837.

(4) Xie, T.-Z.; Luo, L.; Zhao, Y.-L.; Li, H.; Xiang, M.-L.; Qin, X.-J.; He, Y.-J.; Zhu, Y.-Y.; Dai, Z.; Wang, Z.-J.; Wei, X.; Liu, Y.-P.; Zhao, L.-X.; Lai, R.; Luo, X.-D. Steroidal Alkaloids with a Potent Analgesic Effect Based on N-type Calcium Channel Inhibition. *Org. Lett.* **2022**, *24*, 467−471.

(5) (a) Moutal, A.; Cai, S.; Yu, J.; Stratton, H. J.; Chefdeville, A.; Gomez, K.; Ran, D.; Madura, C. L.; Boinon, L.; Soto, M.; Zhou, Y.; Shan, Z.; Chew, L. A.; Rodgers, K. E.; Khanna, R. Studies on CRMP2 SUMOylation-deficient transgenic mice identify sex-specific $Na_V1.7$ regulation in the pathogenesis of chronic neuropathic pain. *PAIN* **2020**, *161*, 2629−2651. (b) Moutal, A.; Chew, L. A.; Yang, X.; Wang, Y.; Yeon, S. K.; Telemi, E.; Meroueh, S.; Park, K. D.; Shrinivasan, R.; Gilbraith, K. B.; Qu, C.; Xie, J. Y.; Patwardhan, A.; Vanderah, T. W.; Khanna, M.; Porreca, F.; Khanna, R. (*S*)-Lacosamide inhibition of CRMP2 phosphorylation reduces postoperative and neuropathic pain behaviors through distinct classes of sensory neurons identified by constellation pharmacology. *PAIN* **2016**, *157*, 1448−1463. (c) François-Moutal, L.; Wang, Y.; Moutal, A.; Cottier, K. E.; Melemedjian, O. K.; Yang, X.; Wang, Y.; Ju, W.; Largent-Milnes, T. M.; Khanna, M.; Vanderah, T. W.; Khanna, R. A memembrane-delimited N-myristoylated CRMP2 peptide aptamer inhibits CaV2.2 trafficking and reverses inflammatory and postoperative pain behaviors. *PAIN* **2015**, *156*, 1247−1264.

(6) Gomez, K.; Santiago, U.; Nelson, T. S.; Allen, H. N.; Calderon-Rivera, A.; Hestehave, S.; Palma, E. J. R.; Zhou, Y.; Duran, P.; Loya-Lopez, S.; Zhu, E.; Kumar, U.; Shields, R.; Koseli, E.; McKiver, B.; Giuvelis, D.; Zuo, W.; Inyang, K. E.; Dorame, A.; Chefdeville, A.; Ran, D.; Perez-Miller, S.; Lu, Y.; Liu, X.; Handoko, Arora, P. S.; Patek, M.; Moutal, A.; Khanna, M.; Hu, H.; Laumet, G.; King, T.; Wang, J.; Damaj, M. I.; Korczeniewska, O. A.; Camacho, C. J.; Khanna, R. *Proc. Natl. Acad. Sci. U. S. A.* **2023**, *120*, e2305215120.

(7) Hestehave, S.; Florea, R.; Fedorec, A. J. H.; Jevic, M.; Mercy, L.; Wright, A.; Morgan, O. B.; Brown, L. A.; Peirson, S. N.; Géranton, S. M. Predicting hypersensitivity and comorbid depressive-like behavior in late stages of joint disease using early weight bearing deficit. *bioRxiv*, 2023.2011.2029.569246, doi:10.1101/2023.11.29.569246 (**2023**).

(8) Hestehave, S.; Abelson, K. S. P.; Pedersen, T. B.; Finn, D. P.; Andersson, D. R.; Munro, G. The influence of rat strain on the development of neuropathic pain and comorbid anxio-depressive behaviour after nerve injury. *Sci. Rep.* **2020**, *10,* 20981.

(9) Dixon, W. J. Efficient analysis of experimental observations. *Annu. Rev. Pharmacol. Toxicol.* **1980**, *20*, 441−462.

(10) Moriarty, O.; Gorman, C. L.; McGowan, F.; Ford, G. K.; Roche, M.; Thompson, K.; Dockery, P.; McGuire, B. E.; Finn, D. P.; Impaired recognition memory and cognitive flexibility in the rat L5-L6 spinal nerve ligation model of neuropathic pain. *Scand J. Pain* **2016**, *10*, 61−73.

8. ^{1}H, and ^{13}C NMR Spectra

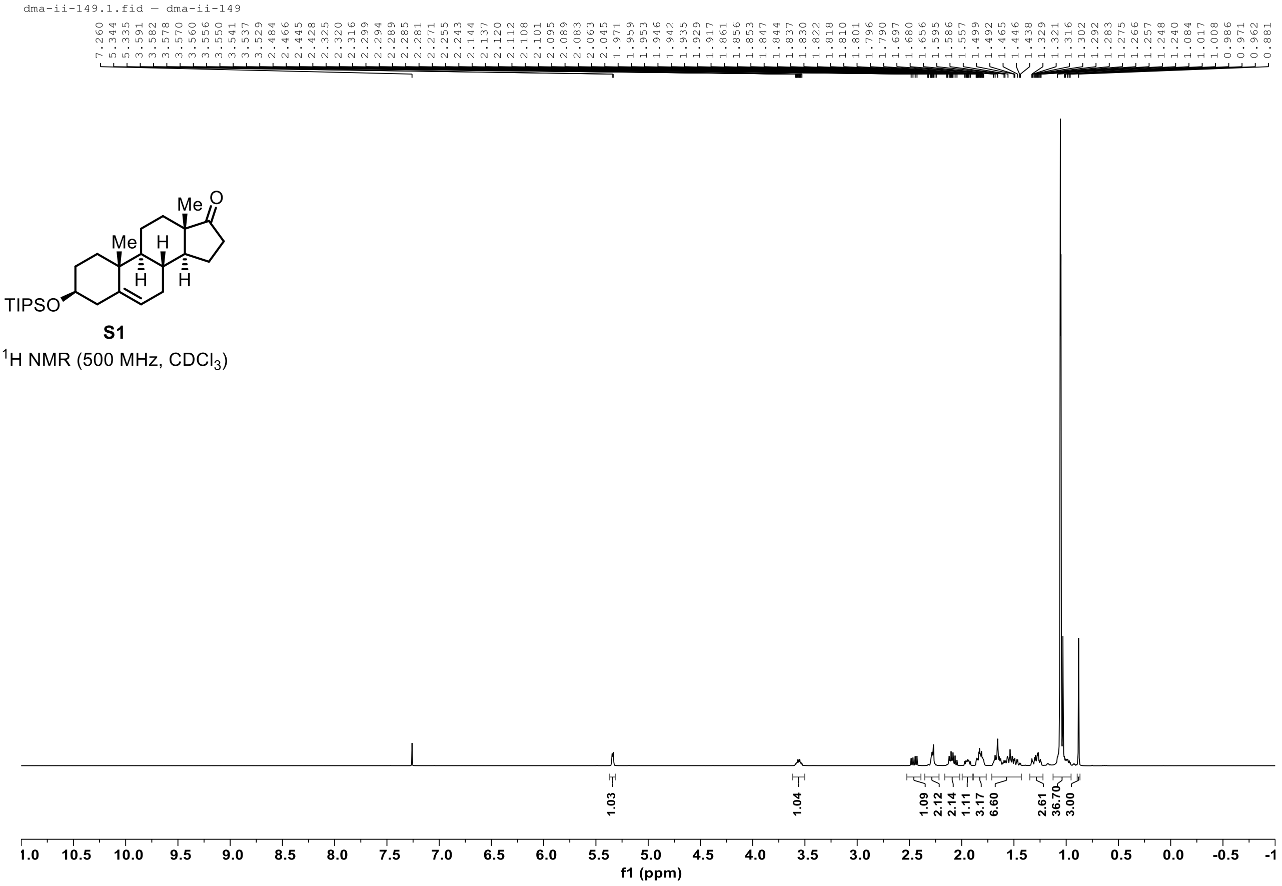

S1

^{1}H NMR (500 MHz, $CDCl_3$)

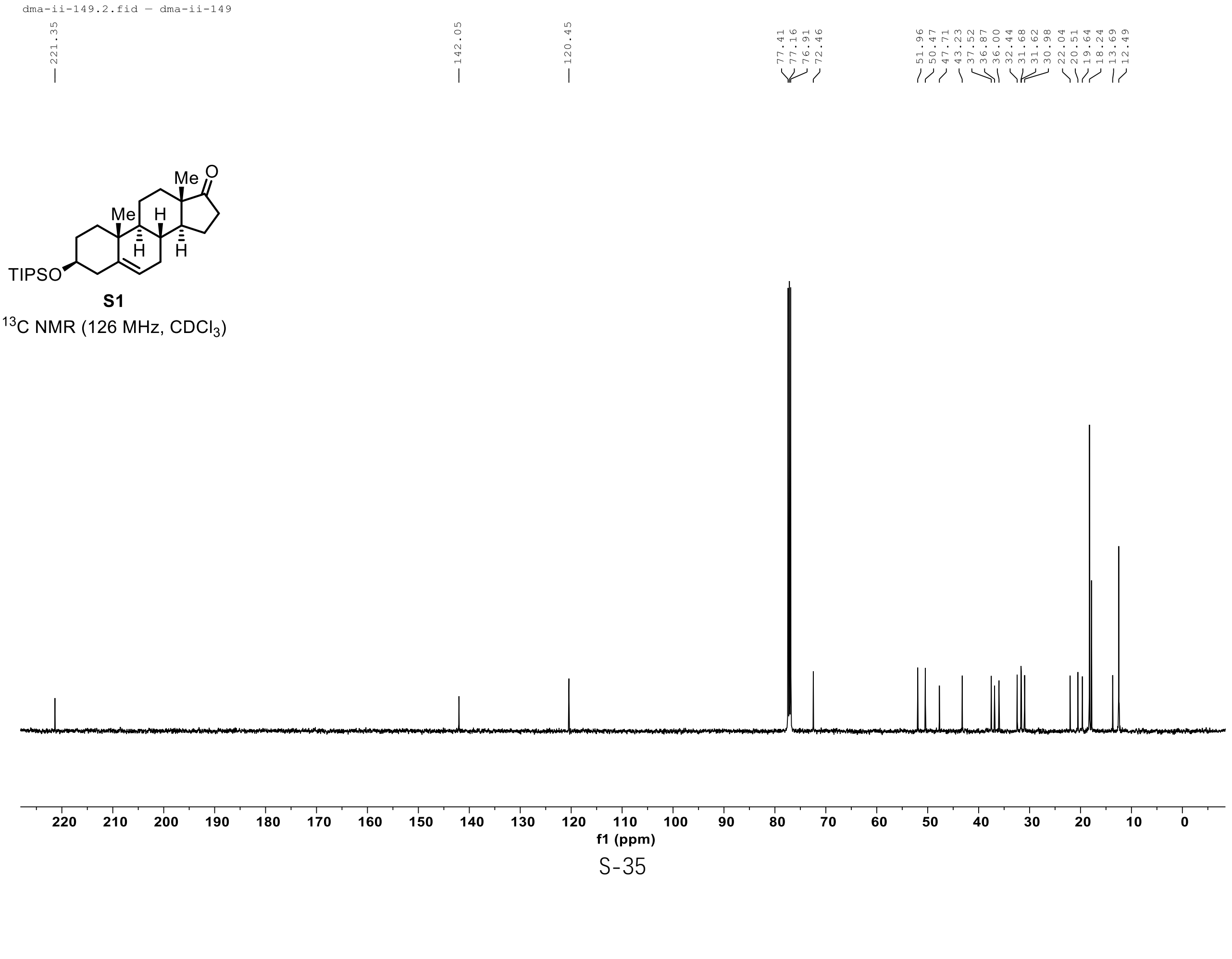

S1

^{13}C NMR (126 MHz, $CDCl_3$)

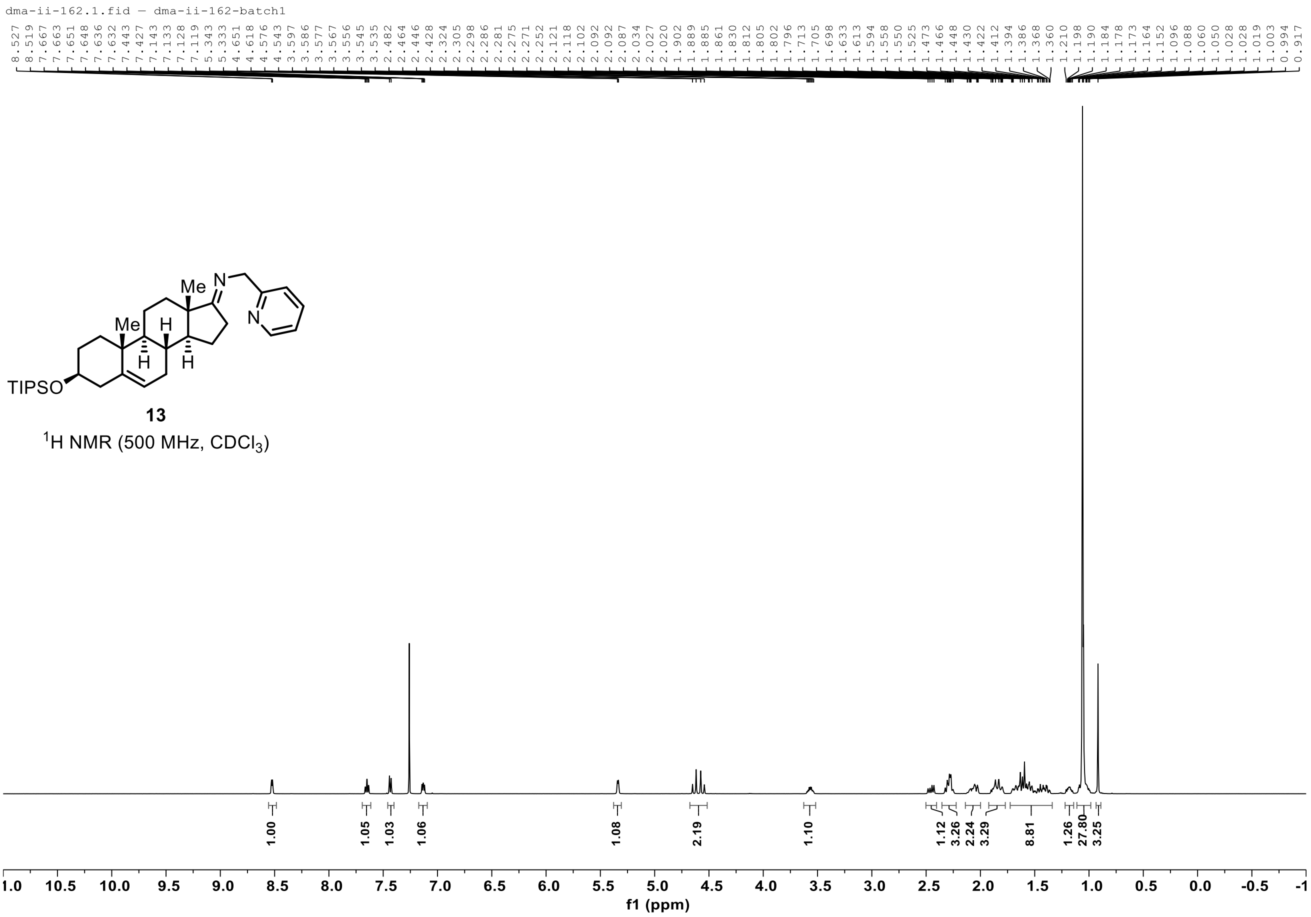

13

^{1}H NMR (500 MHz, $CDCl_3$)

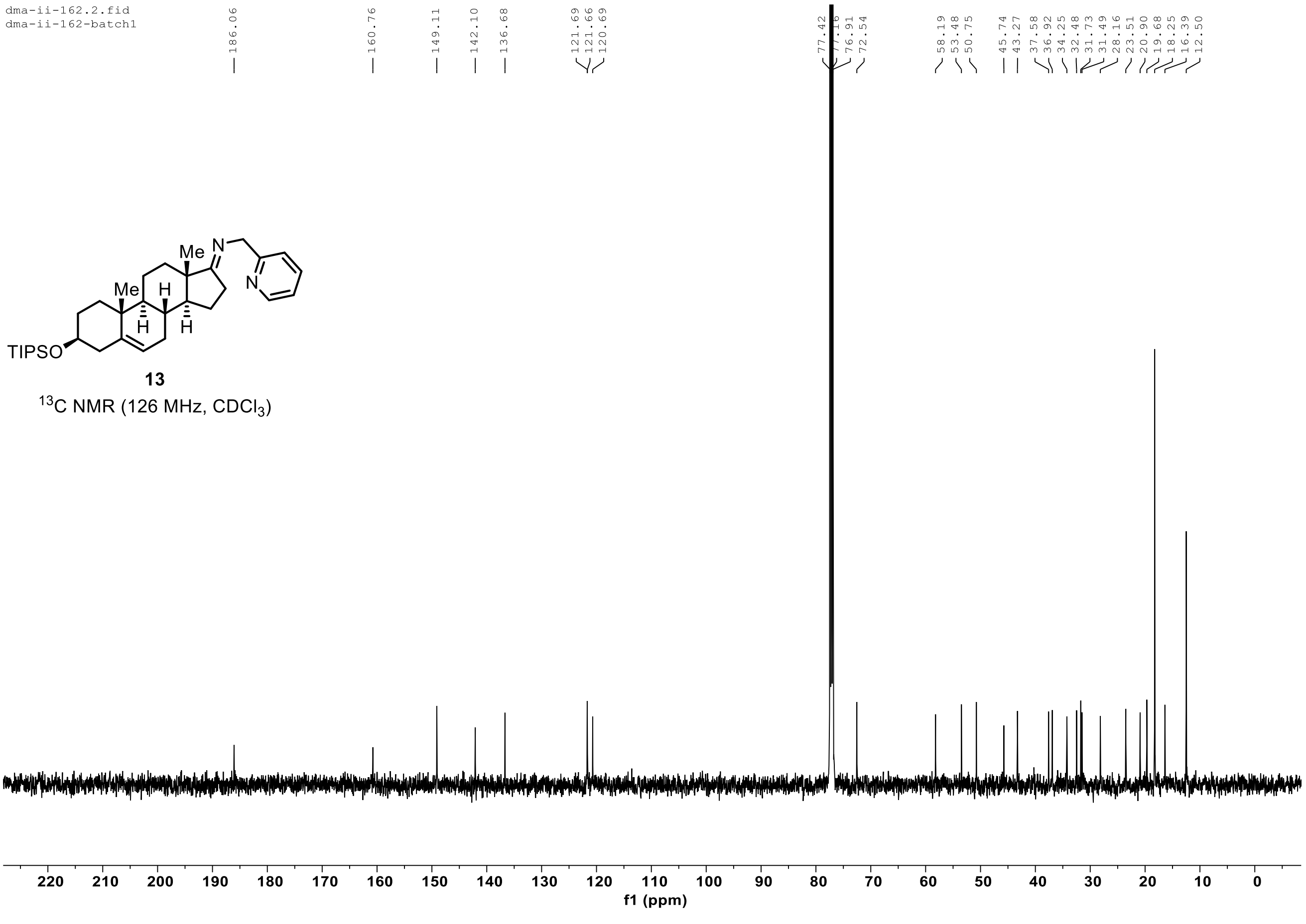

13

^{13}C NMR (126 MHz, $CDCl_3$)

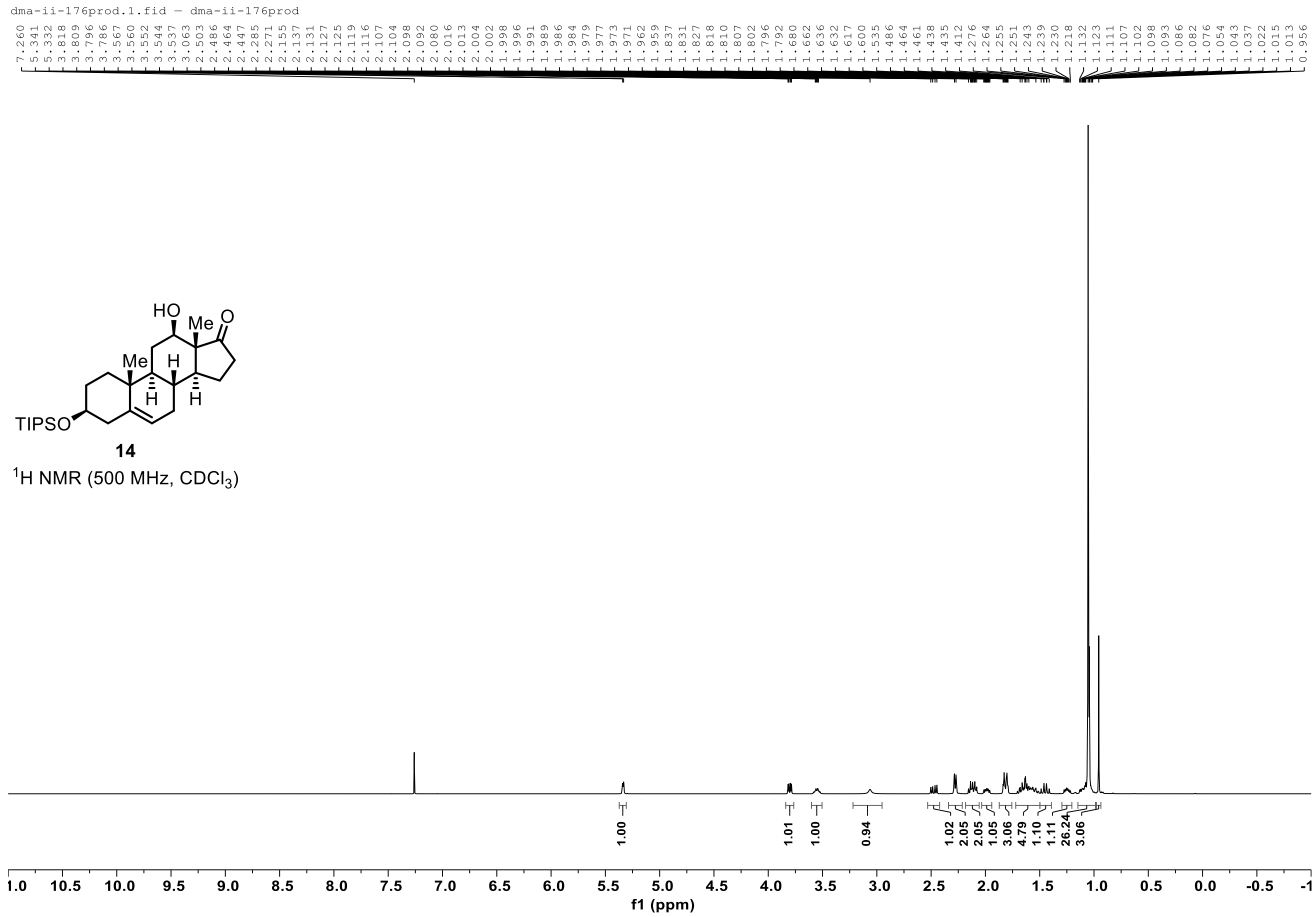

14

^{1}H NMR (500 MHz, $CDCl_3$)

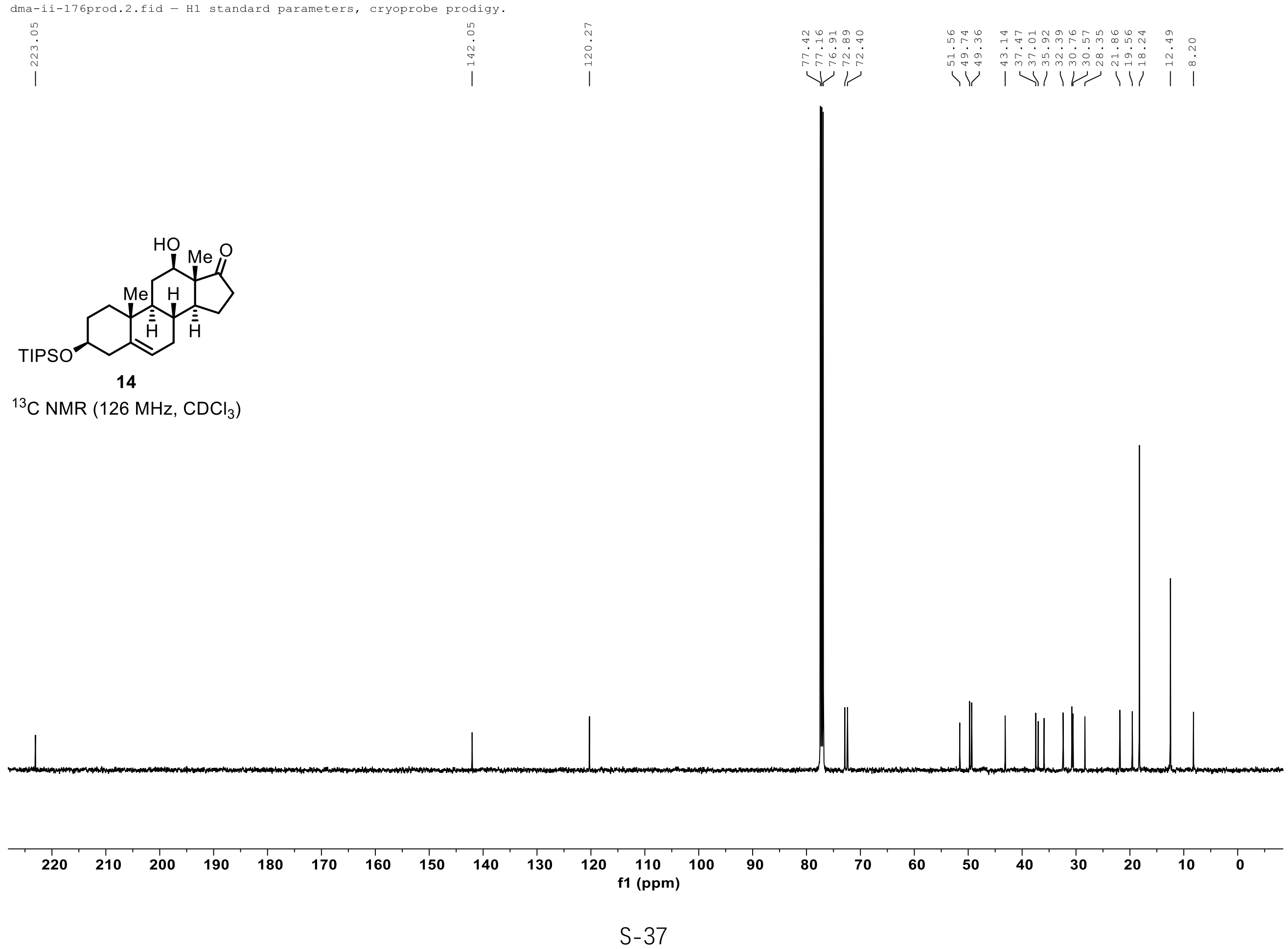

14

^{13}C NMR (126 MHz, $CDCl_3$)

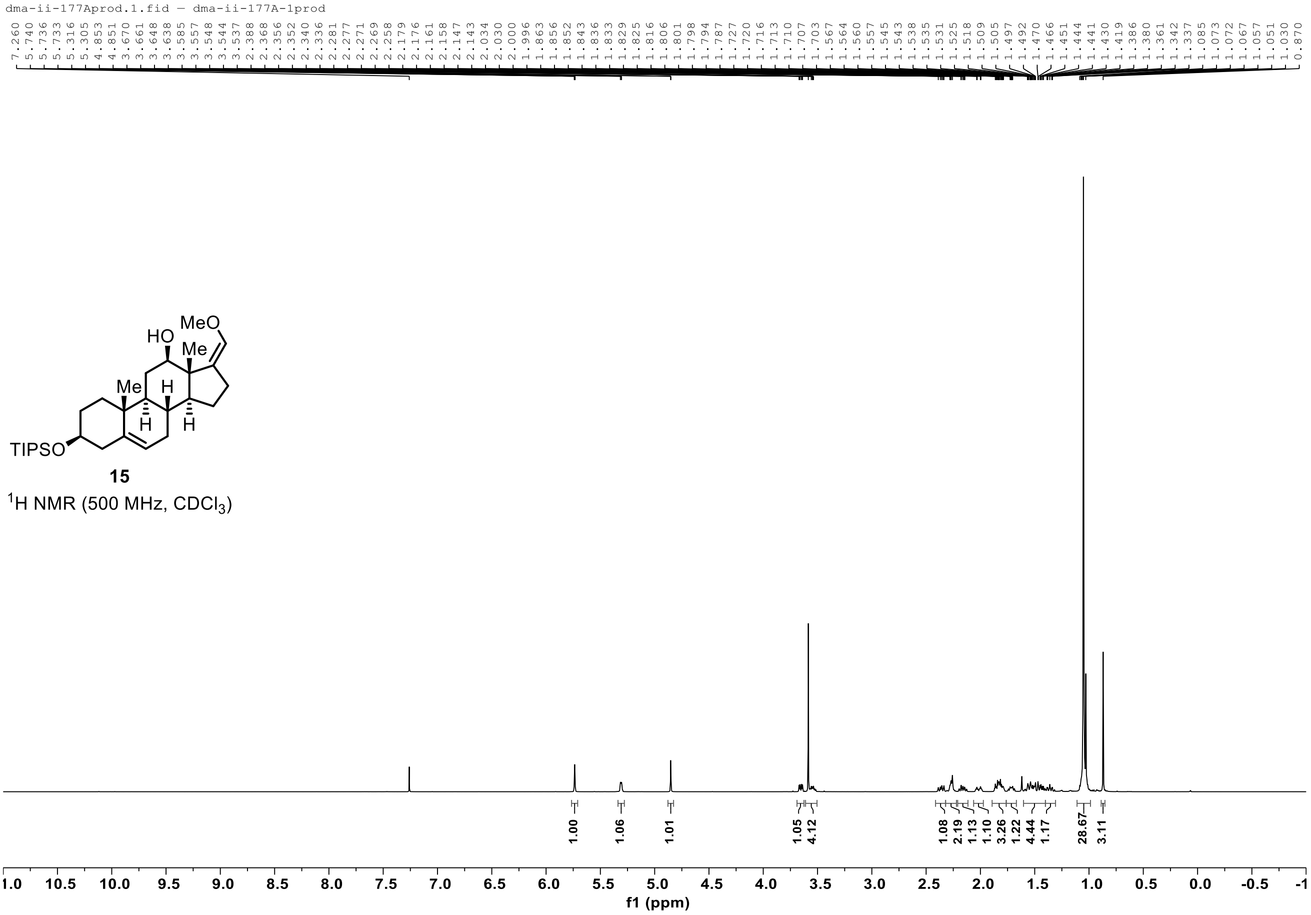

15

^{1}H NMR (500 MHz, $CDCl_3$)

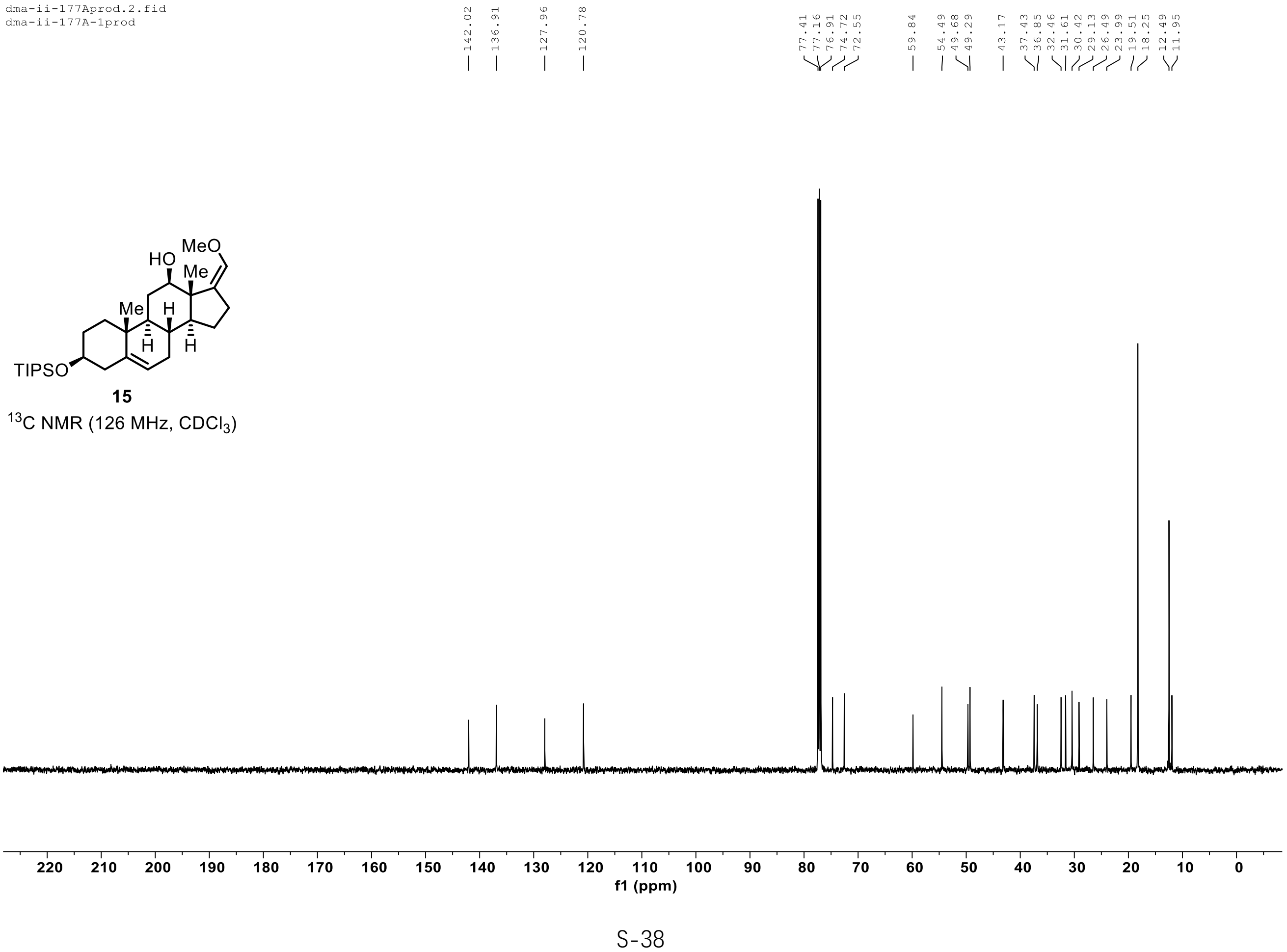

15

^{13}C NMR (126 MHz, $CDCl_3$)

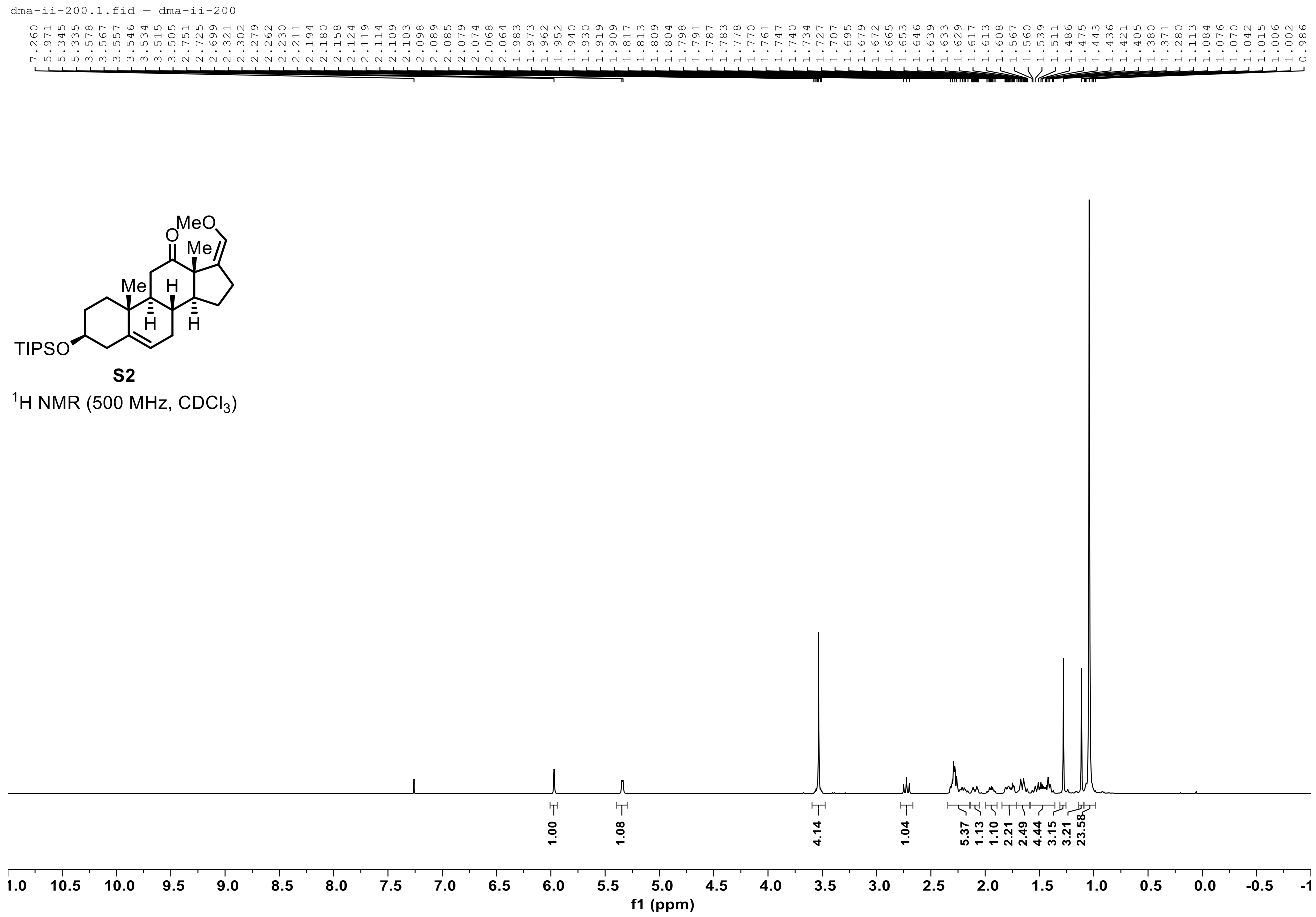
dma-ii-200.1.fid — dma-ii-200
7.260 5.971 5.345 5.335 3.578 3.567 3.557 3.546 3.534 3.515 3.505 2.751 2.725 2.699 2.321 2.302 2.279 2.262 2.230 2.211 2.194 2.180 2.158 2.124 2.119 2.114 2.109 2.103 2.098 2.089 2.085 2.079 2.074 2.068 2.064 1.983 1.973 1.962 1.952 1.940 1.930 1.919 1.909 1.817 1.813 1.809 1.804 1.798 1.791 1.787 1.783 1.778 1.770 1.761 1.747 1.740 1.734 1.727 1.707 1.695 1.679 1.672 1.665 1.653 1.646 1.639 1.633 1.629 1.617 1.613 1.608 1.567 1.560 1.539 1.511 1.486 1.475 1.443 1.436 1.421 1.405 1.380 1.371 1.280 1.113 1.084 1.076 1.070 1.042 1.015 1.006 1.002 0.986
MeO
O
Me
Me
H
H
H
TIPSO
S2
^{1}H NMR (500 MHz, $CDCl_3$)
1.00
1.08
4.14
1.04
5.37
1.13
1.10
2.21
2.49
4.44
3.15
3.21
23.58
f1 (ppm)

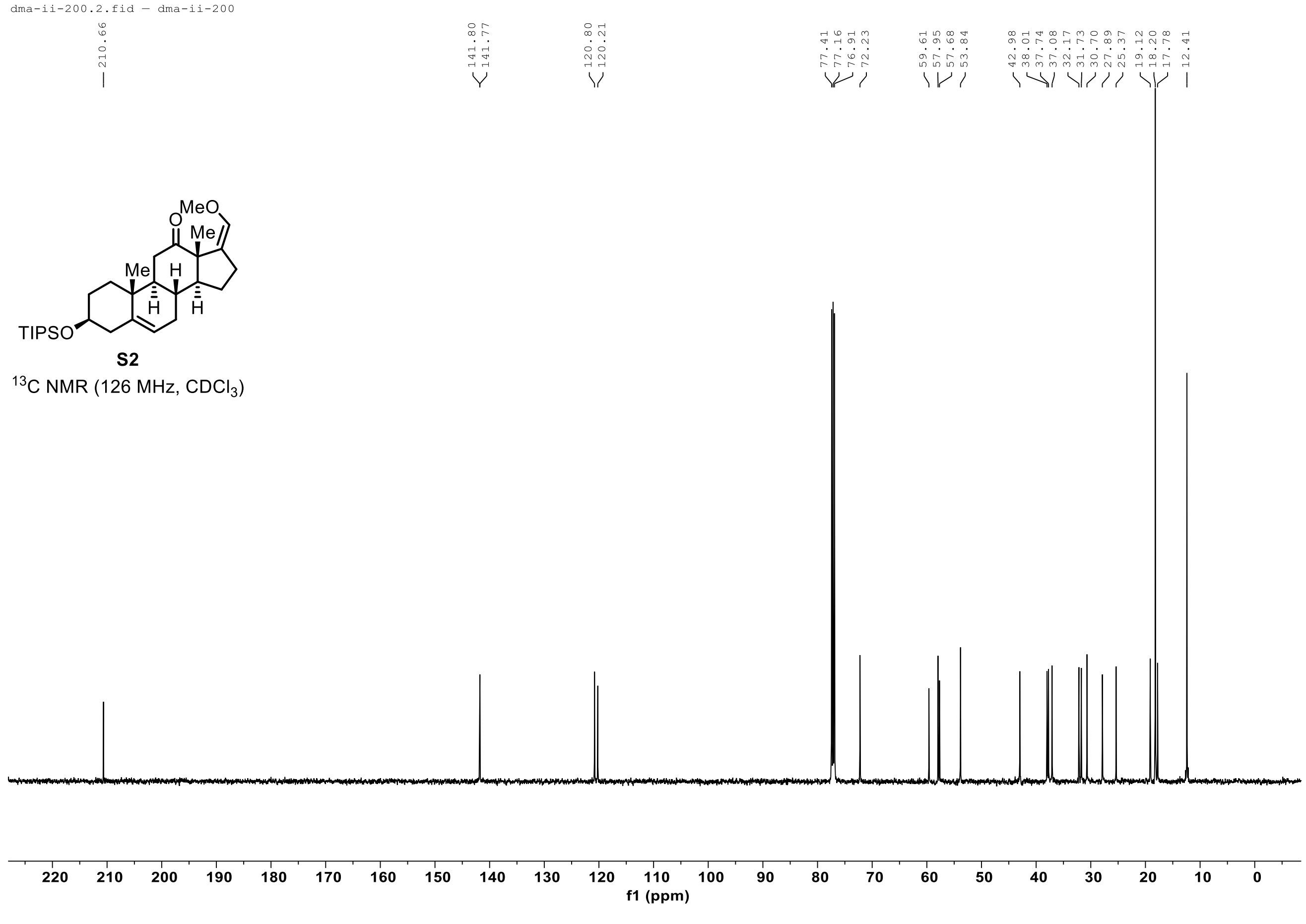
dma-ii-200.2.fid — dma-ii-200
210.66 141.80 141.77 120.80 120.21 77.41 77.16 76.91 72.23 59.61 57.95 57.68 53.84 42.98 38.01 37.74 37.08 32.17 31.73 30.70 27.89 25.37 19.12 18.20 17.78 12.41
MeO
O
Me
Me
H
H
H
TIPSO
S2
^{13}C NMR (126 MHz, $CDCl_3$)
f1 (ppm)

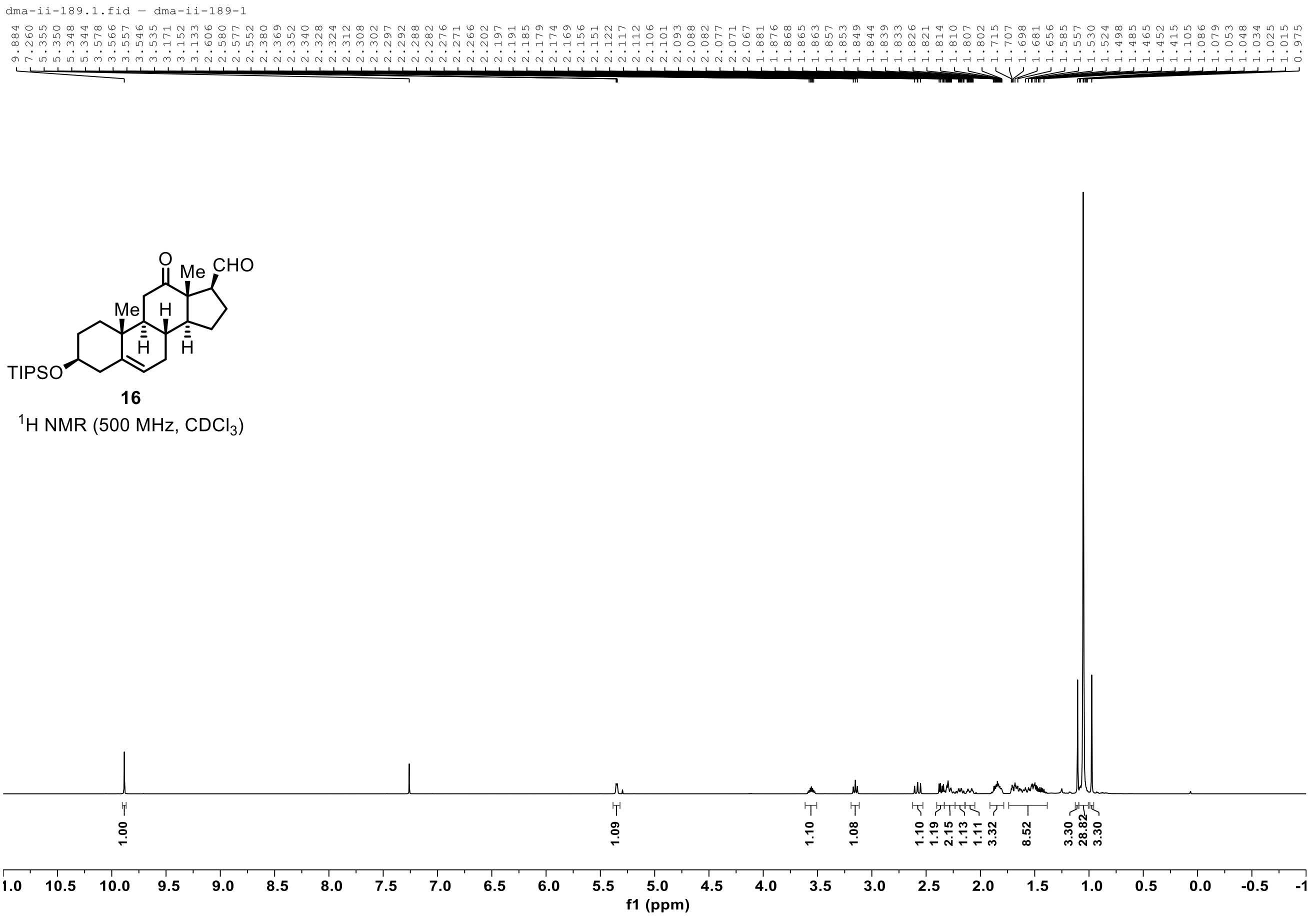
dma-ii-189.1.fid — dma-ii-189-1
9.884 7.260 5.355 5.350 5.348 5.344 3.578 3.566 3.557 3.546 3.535 3.171 3.152 3.133 2.606 2.580 2.577 2.552 2.380 2.369 2.352 2.340 2.328 2.324 2.312 2.308 2.302 2.297 2.292 2.288 2.282 2.276 2.271 2.266 2.202 2.197 2.191 2.185 2.179 2.174 2.169 2.156 2.151 2.122 2.117 2.112 2.106 2.101 2.093 2.088 2.082 2.077 2.071 2.067 1.881 1.876 1.868 1.865 1.863 1.857 1.853 1.849 1.844 1.839 1.833 1.826 1.821 1.814 1.810 1.807 1.802 1.715 1.707 1.698 1.681 1.656 1.585 1.557 1.530 1.524 1.498 1.485 1.465 1.452 1.415 1.105 1.086 1.079 1.053 1.048 1.034 1.025 1.015 0.975
O
Me
CHO
Me
H
H
H
TIPSO
16
¹H NMR (500 MHz, CDCl₃)
1.00
1.09
1.10
1.08
1.10
1.19
2.15
1.13
1.11
3.32
8.52
3.30
28.82
3.30
f1 (ppm)

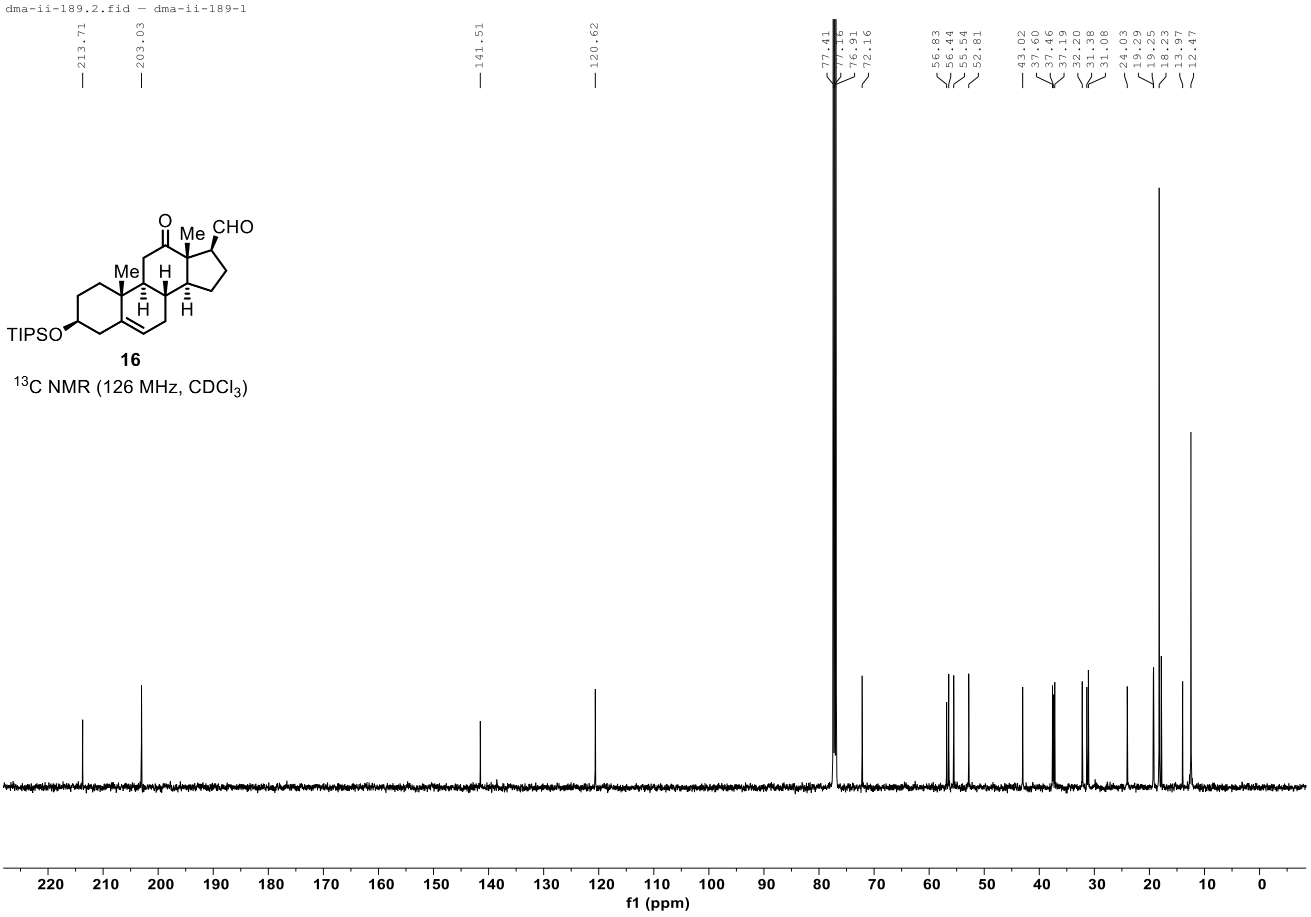
dma-ii-189.2.fid — dma-ii-189-1
213.71
203.03
141.51
120.62
77.41
77.16
76.91
72.16
56.83
56.44
55.54
52.81
43.02
37.60
37.46
37.19
32.20
31.38
31.08
24.03
19.29
19.25
18.23
13.97
12.47
O
Me
CHO
Me
H
H
H
TIPSO
16
¹³C NMR (126 MHz, CDCl₃)
f1 (ppm)

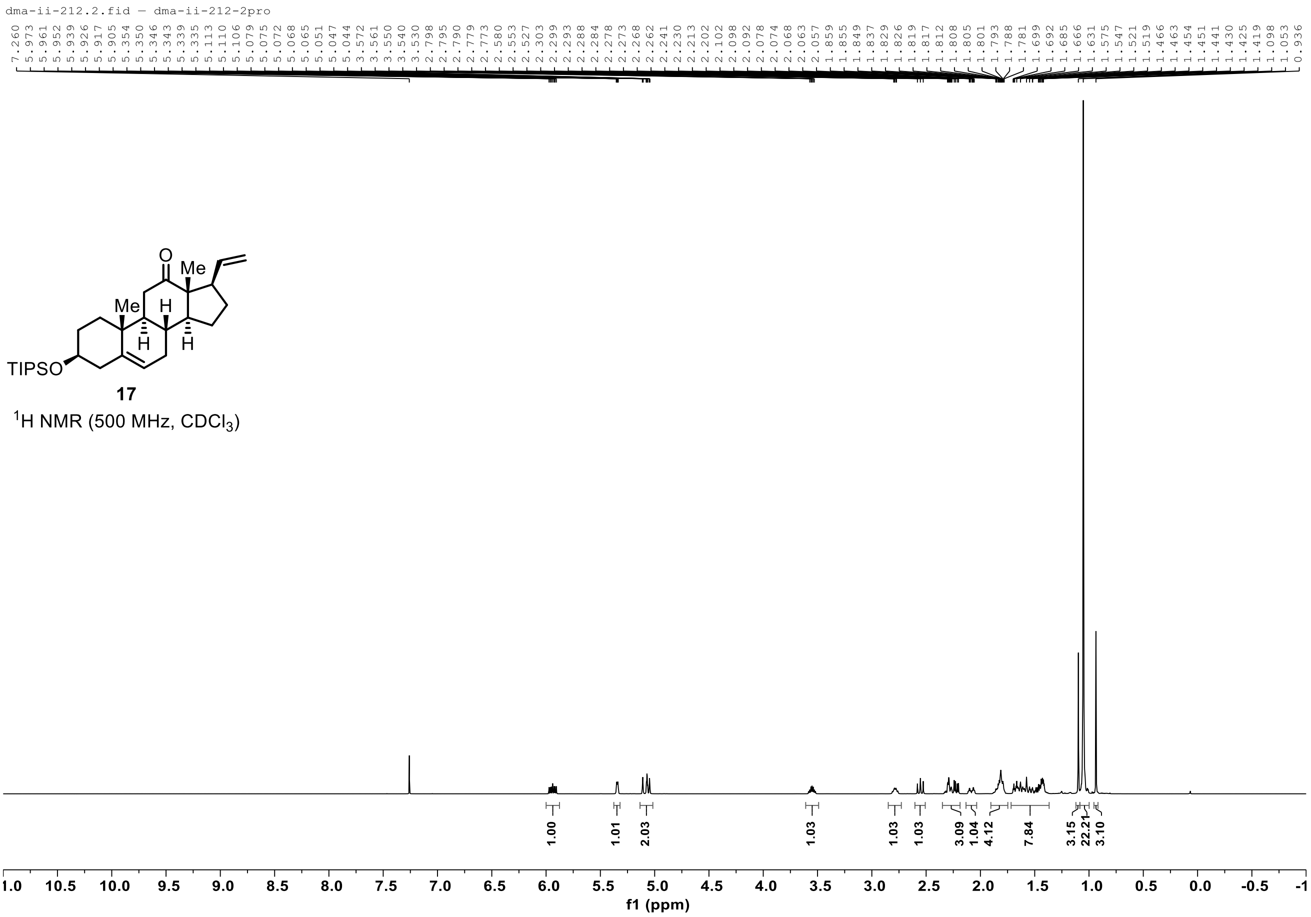

17

^{1}H NMR (500 MHz, $CDCl_3$)

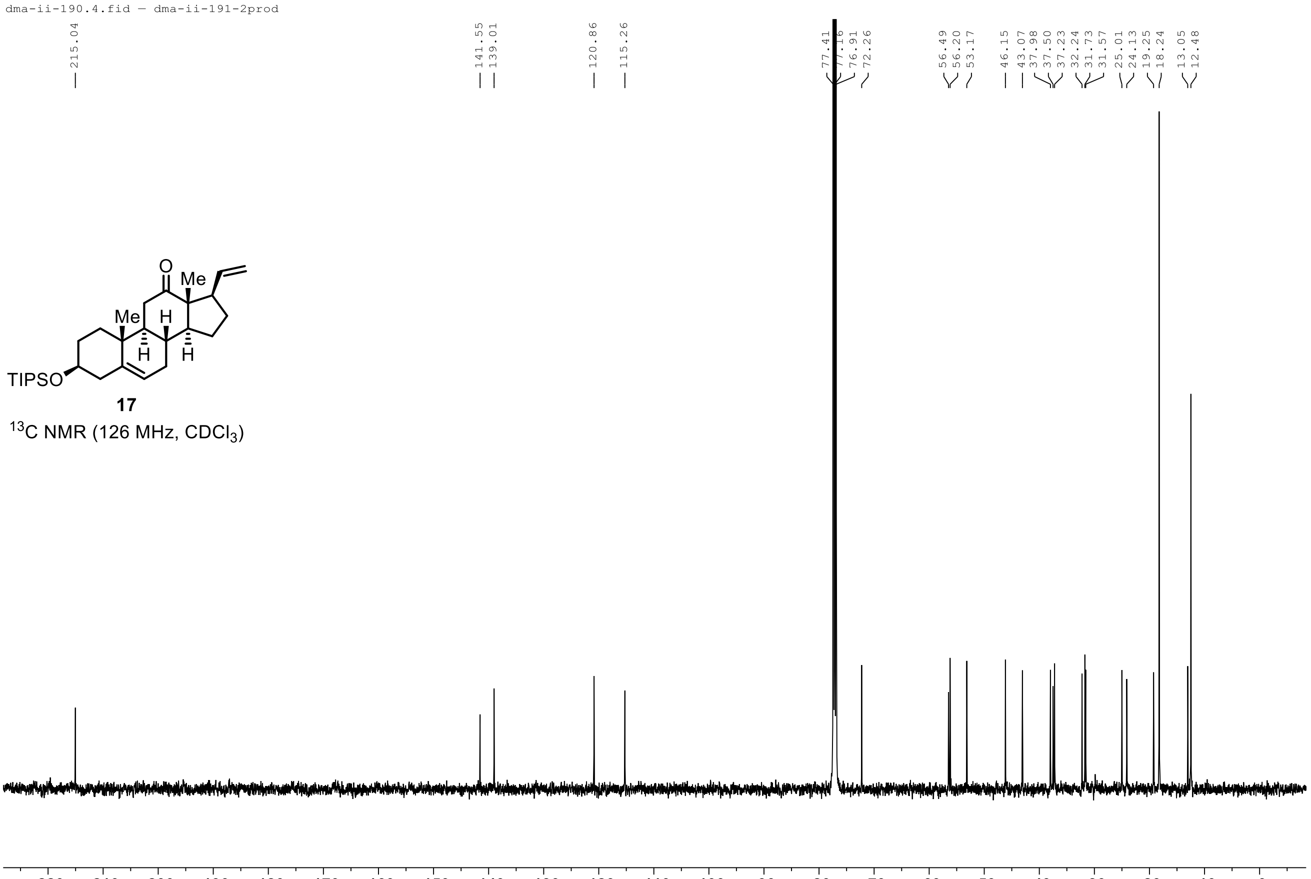

17

^{13}C NMR (126 MHz, $CDCl_3$)

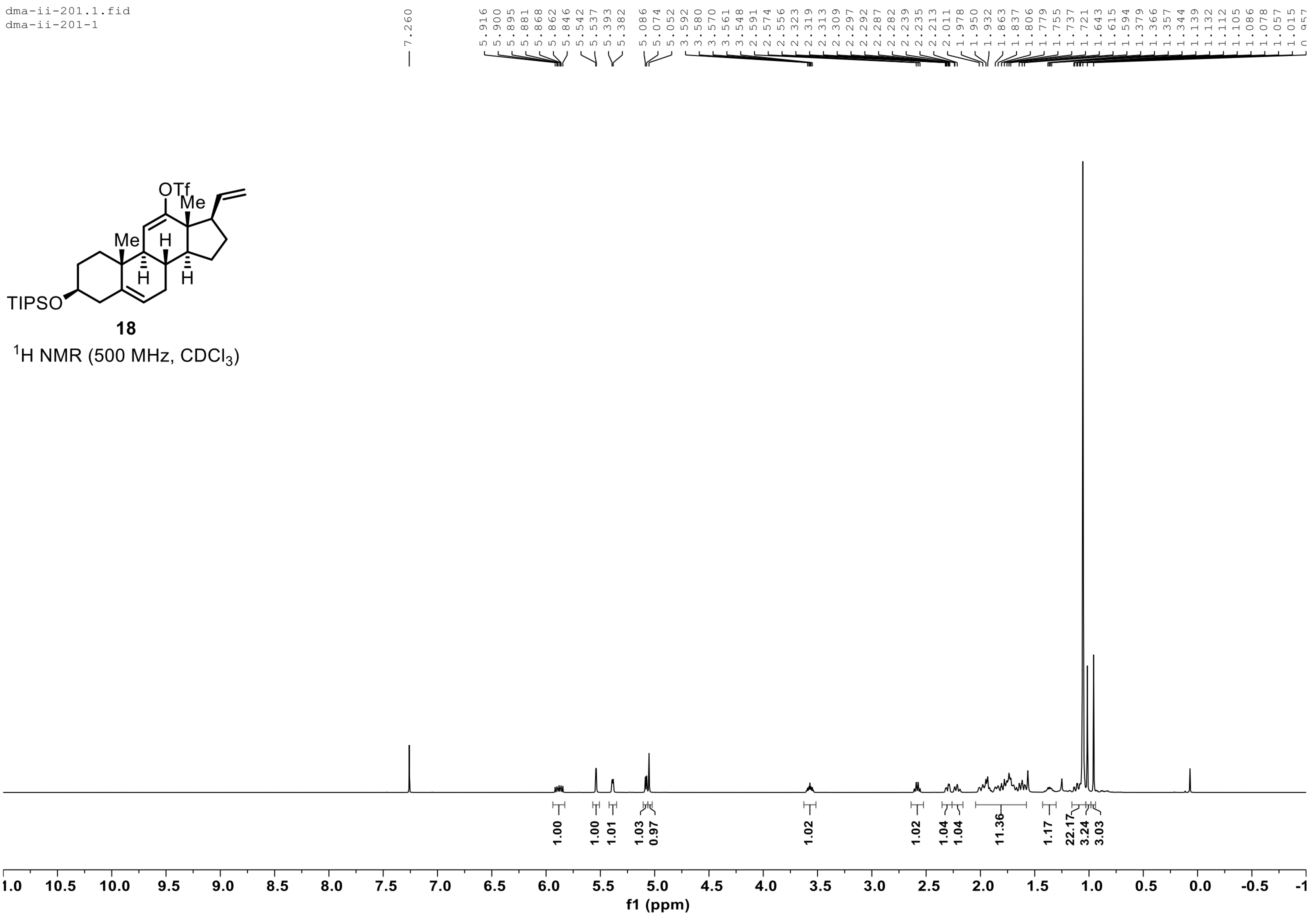

18

^{1}H NMR (500 MHz, $CDCl_3$)

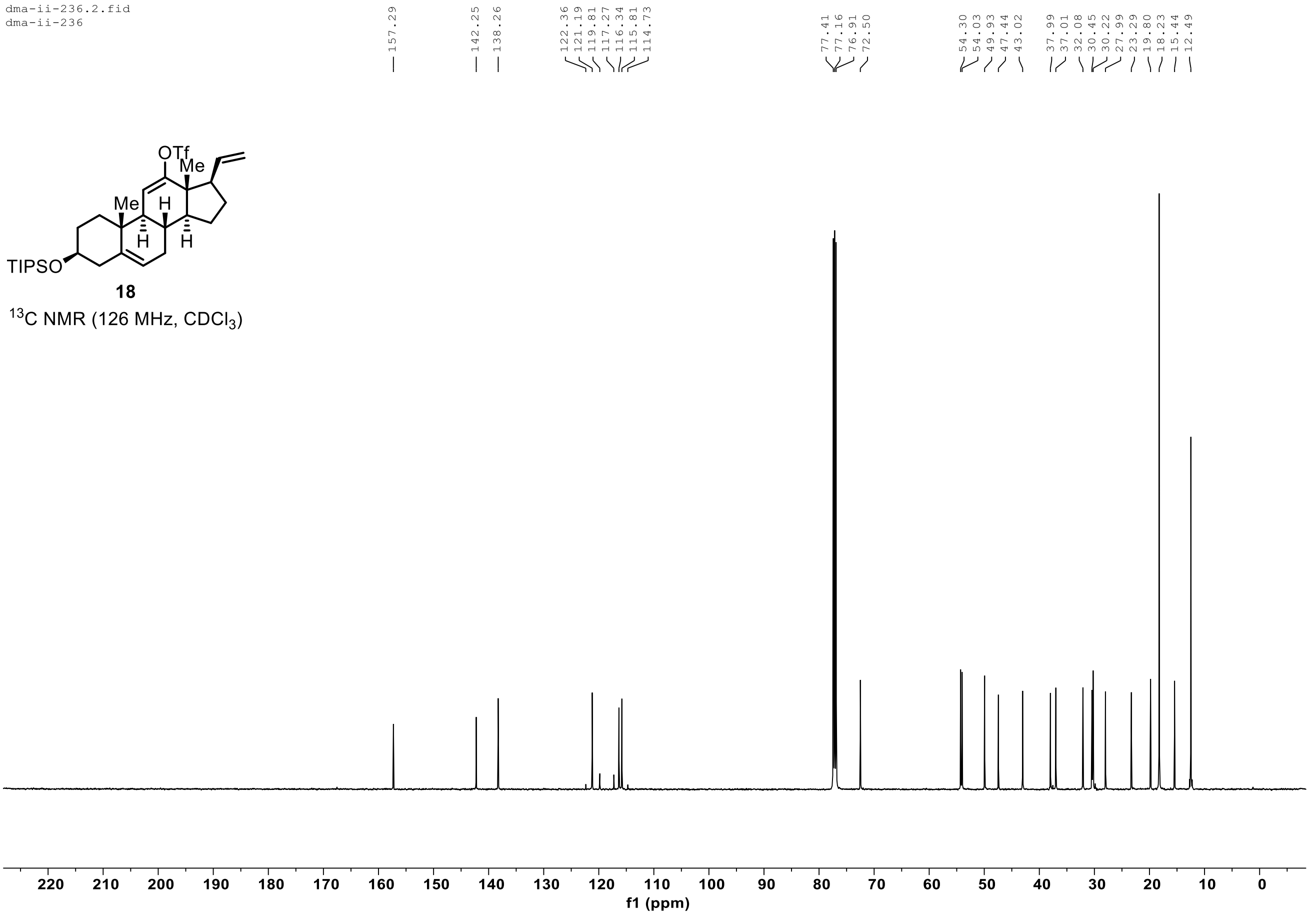

18

^{13}C NMR (126 MHz, $CDCl_3$)

dma-ii-236.3.fid
dma-ii-236

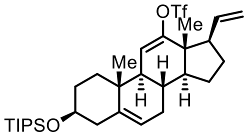

18

^{19}F NMR (471 MHz, $CDCl_3$)

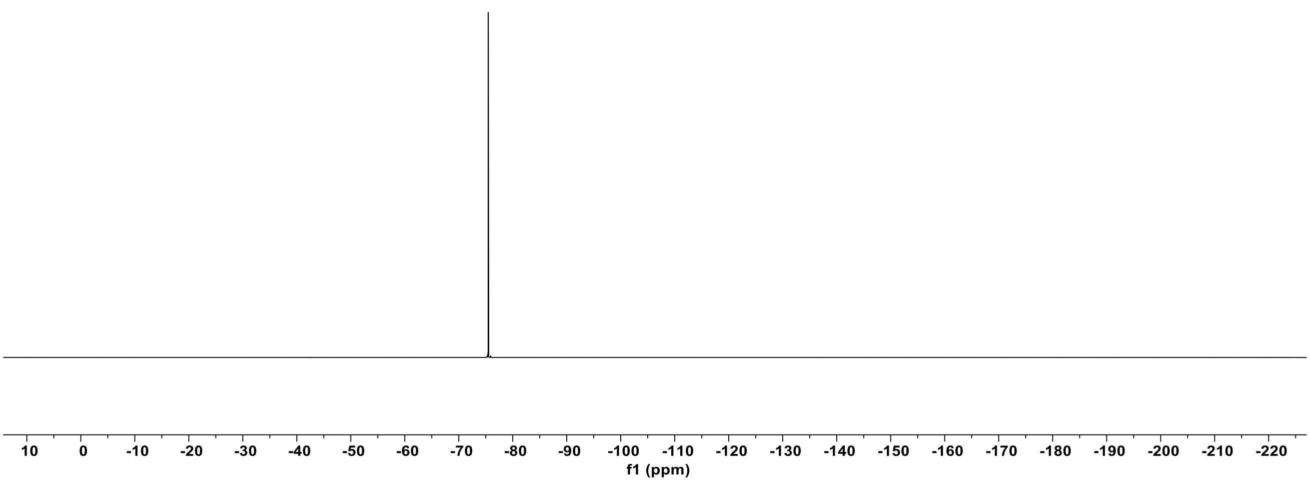

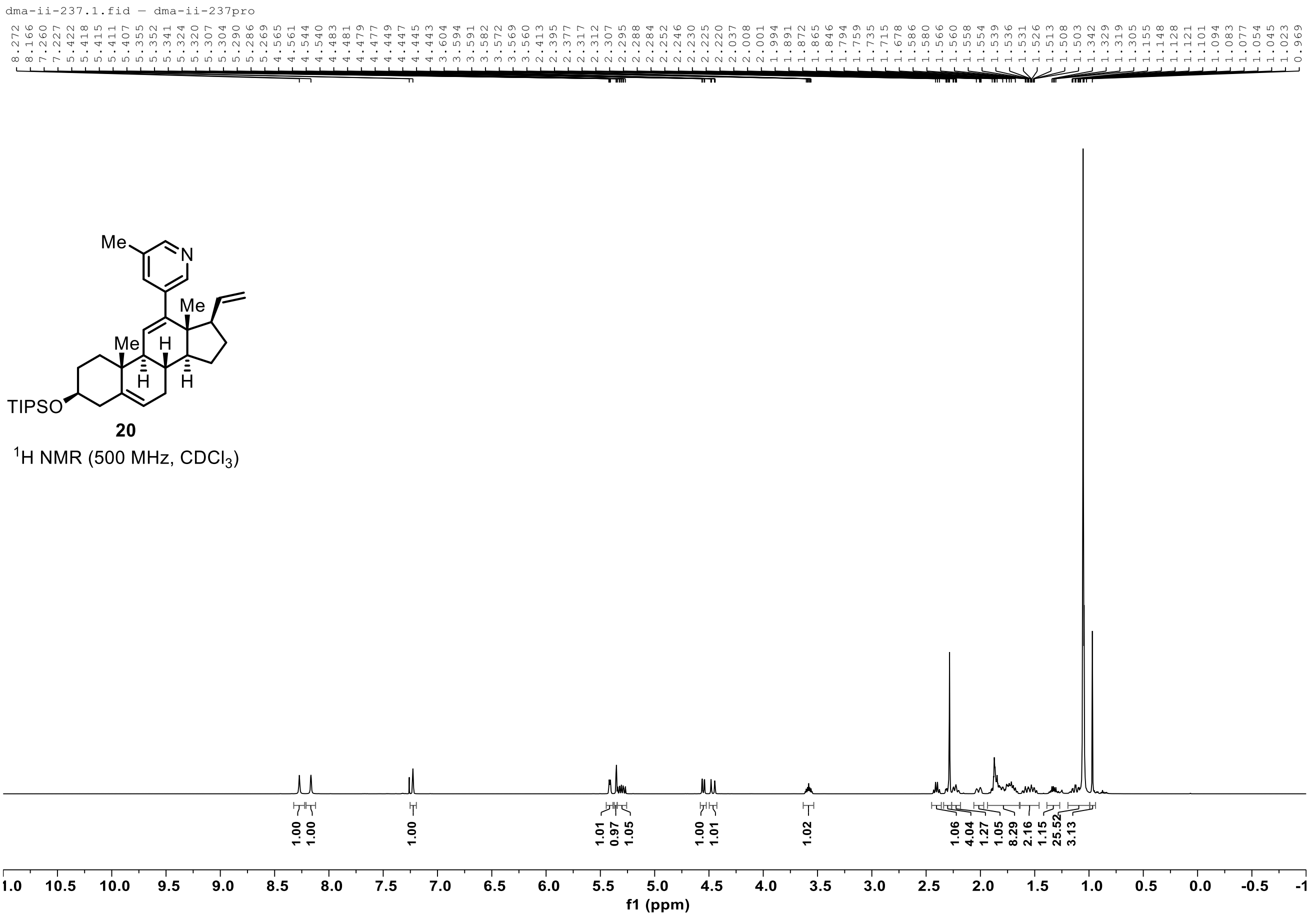
dma-ii-237.1.fid — dma-ii-237pro
8.272 8.166 7.260 7.227 7.222 5.422 5.418 5.415 5.411 5.407 5.355 5.352 5.341 5.324 5.320 5.307 5.304 5.290 5.286 5.269 4.565 4.561 4.544 4.540 4.483 4.481 4.479 4.477 4.449 4.447 4.445 4.443 3.604 3.594 3.591 3.582 3.572 3.569 3.560 2.413 2.395 2.377 2.317 2.312 2.307 2.295 2.288 2.284 2.252 2.246 2.230 2.225 2.220 2.037 2.008 2.001 1.994 1.891 1.872 1.865 1.846 1.794 1.759 1.735 1.715 1.678 1.586 1.580 1.566 1.560 1.558 1.554 1.539 1.536 1.531 1.526 1.513 1.508 1.503 1.342 1.329 1.319 1.305 1.155 1.148 1.128 1.121 1.101 1.094 1.083 1.077 1.054 1.045 1.023 0.969
Me
N
Me
Me
H
H
H
TIPSO
20
^{1}H NMR (500 MHz, CDCl$_3$)
1.00 1.00 1.00 1.01 0.97 1.05 1.00 1.01 1.02 1.06 4.04 1.27 1.05 8.29 2.16 1.15 25.52 3.13
1.0 10.5 10.0 9.5 9.0 8.5 8.0 7.5 7.0 6.5 6.0 5.5 5.0 4.5 4.0 3.5 3.0 2.5 2.0 1.5 1.0 0.5 0.0 -0.5 -1
f1 (ppm)

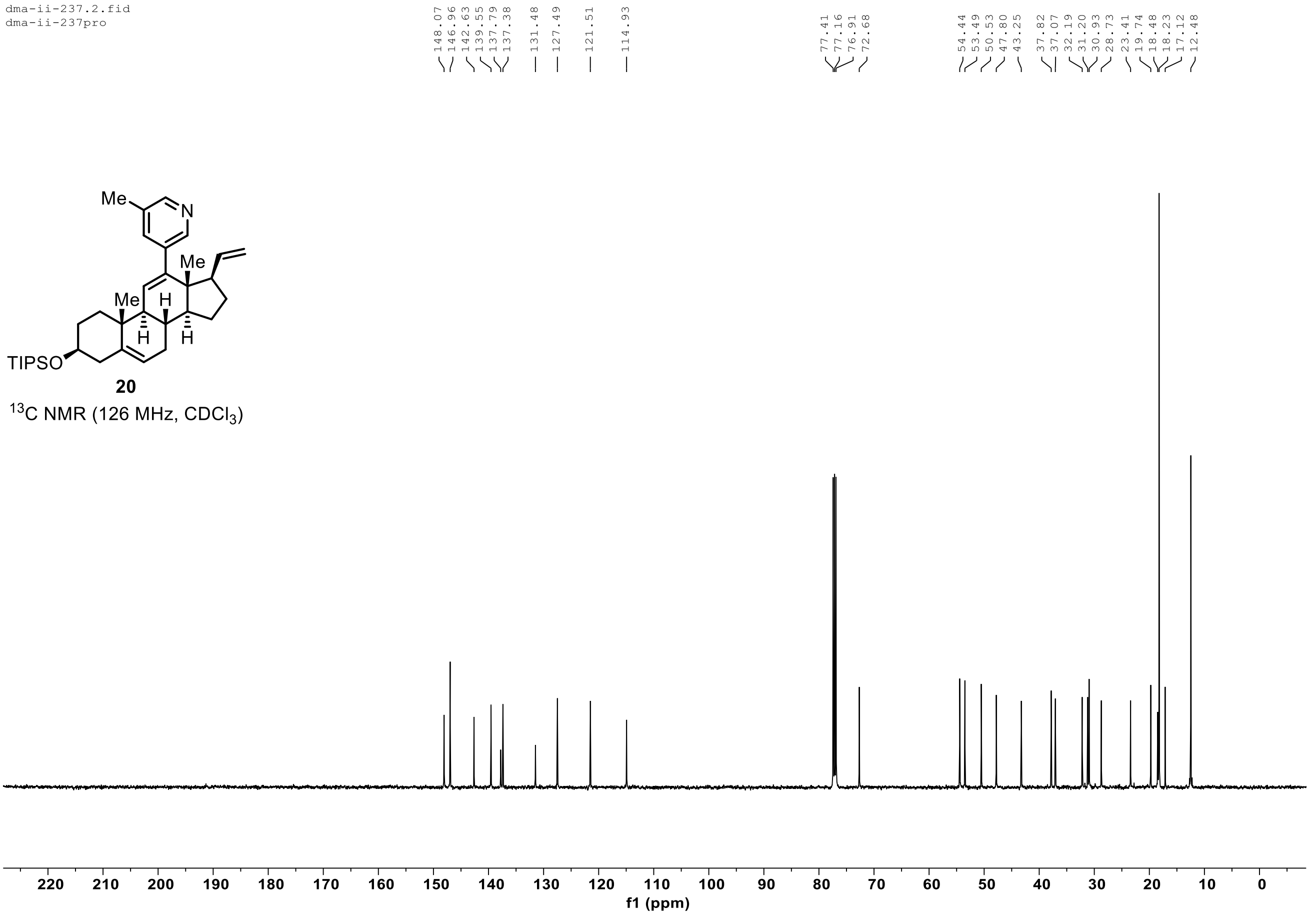
dma-ii-237.2.fid
dma-ii-237pro
148.07 146.96 142.63 139.55 137.79 137.38 131.48 127.49 121.51 114.93 77.41 77.16 76.91 72.68 54.44 53.49 50.53 47.80 43.25 37.82 37.07 32.19 31.20 30.93 28.73 23.41 19.74 18.48 18.23 17.12 12.48
Me
N
Me
Me
H
H
H
TIPSO
20
^{13}C NMR (126 MHz, CDCl$_3$)
220 210 200 190 180 170 160 150 140 130 120 110 100 90 80 70 60 50 40 30 20 10 0
f1 (ppm)

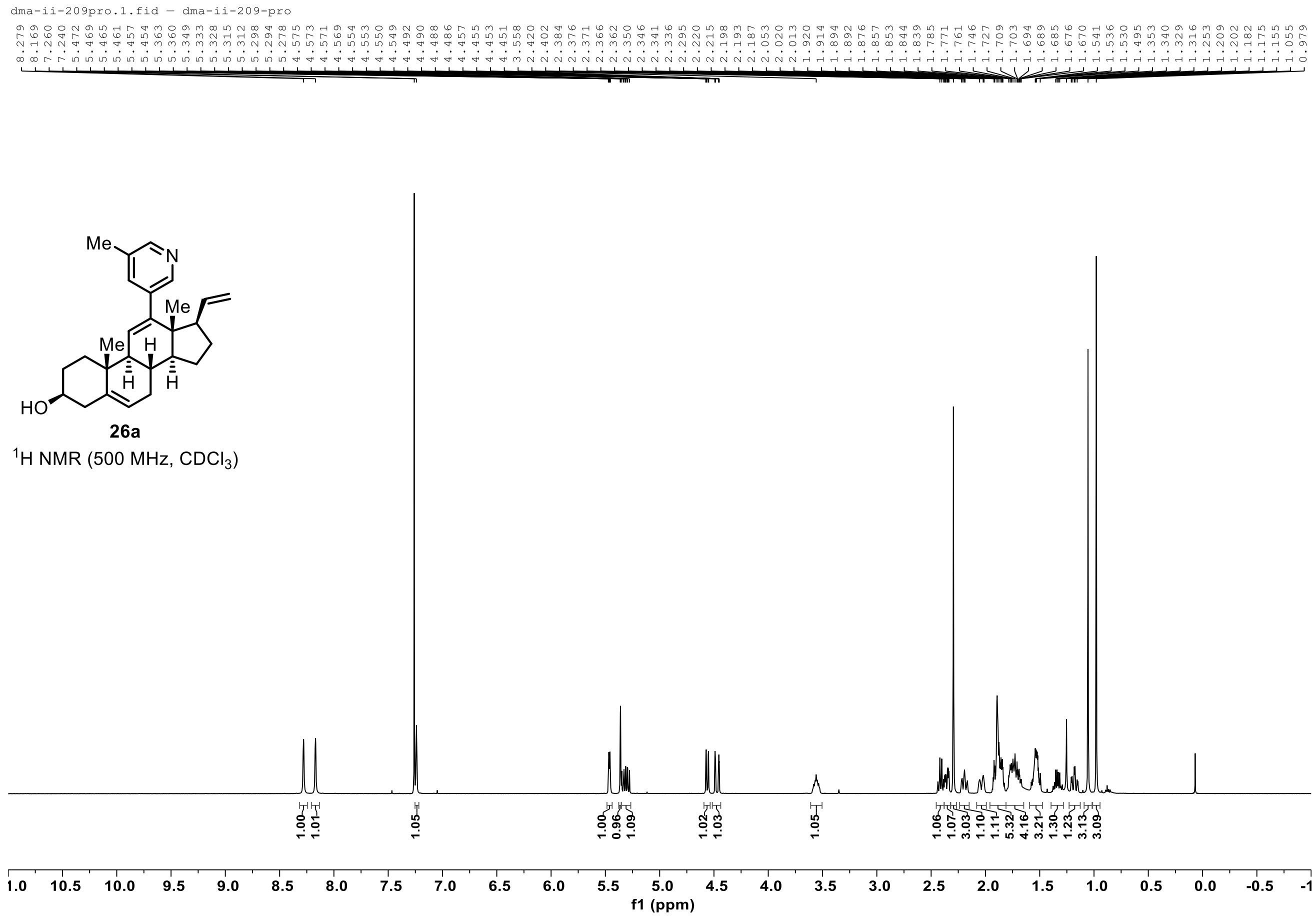
dma-ii-209pro.1.fid — dma-ii-209-pro
Me
N
Me
Me
H
H
H
HO
26a
^{1}H NMR (500 MHz, $CDCl_3$)
f1 (ppm)

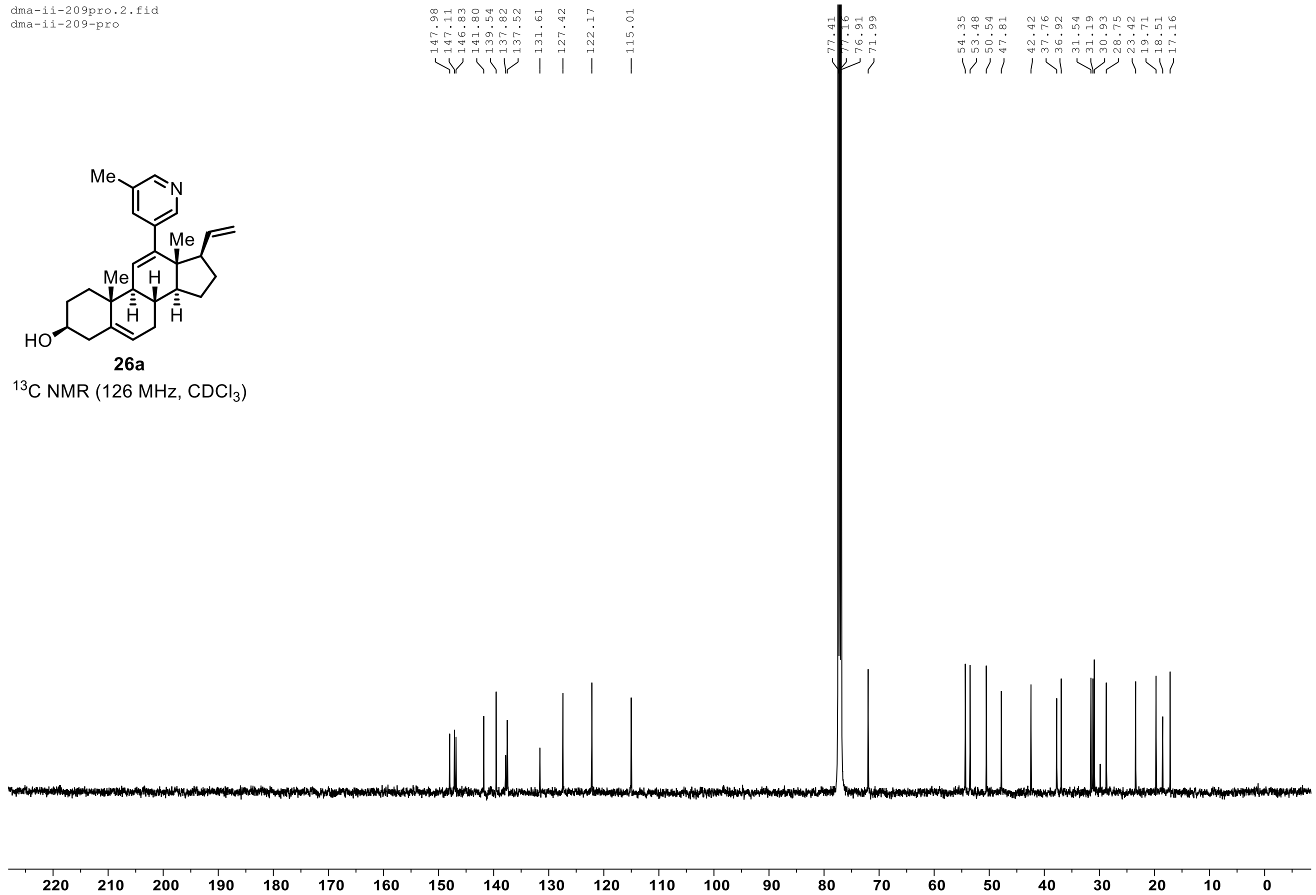
dma-ii-209pro.2.fid
dma-ii-209-pro
147.98
147.11
146.83
141.80
139.54
137.82
137.52
131.61
127.42
122.17
115.01
77.41
77.16
76.91
71.99
54.35
53.48
50.54
47.81
42.42
37.76
36.92
31.54
31.19
30.93
28.75
23.42
19.71
18.51
17.16
Me
N
Me
Me
H
H
H
HO
26a
^{13}C NMR (126 MHz, $CDCl_3$)
f1 (ppm)

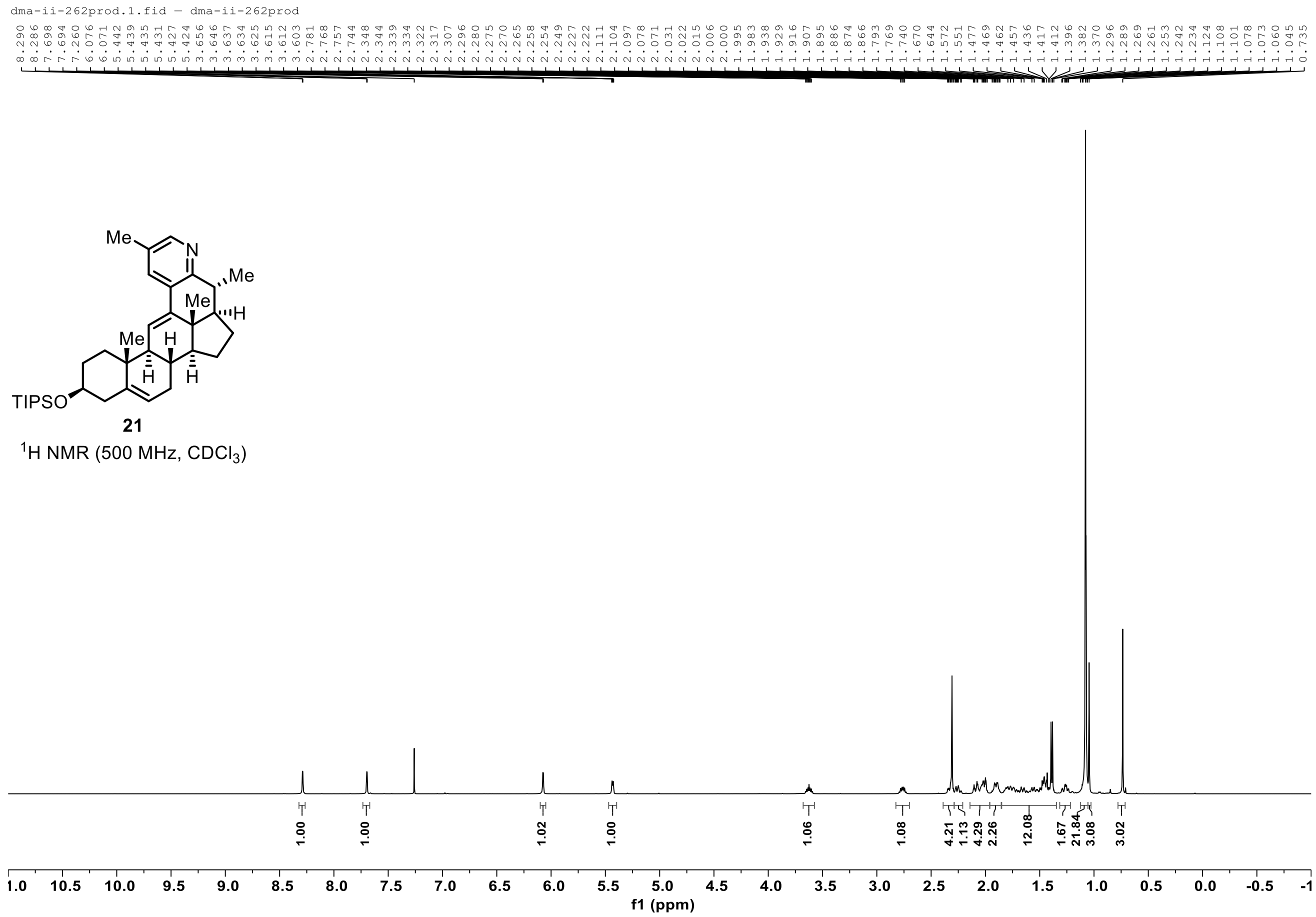
dma-ii-262prod.1.fid — dma-ii-262prod
Me
N
Me
Me
H
Me
H
H
H
TIPSO
21
^{1}H NMR (500 MHz, CDCl$_3$)
f1 (ppm)

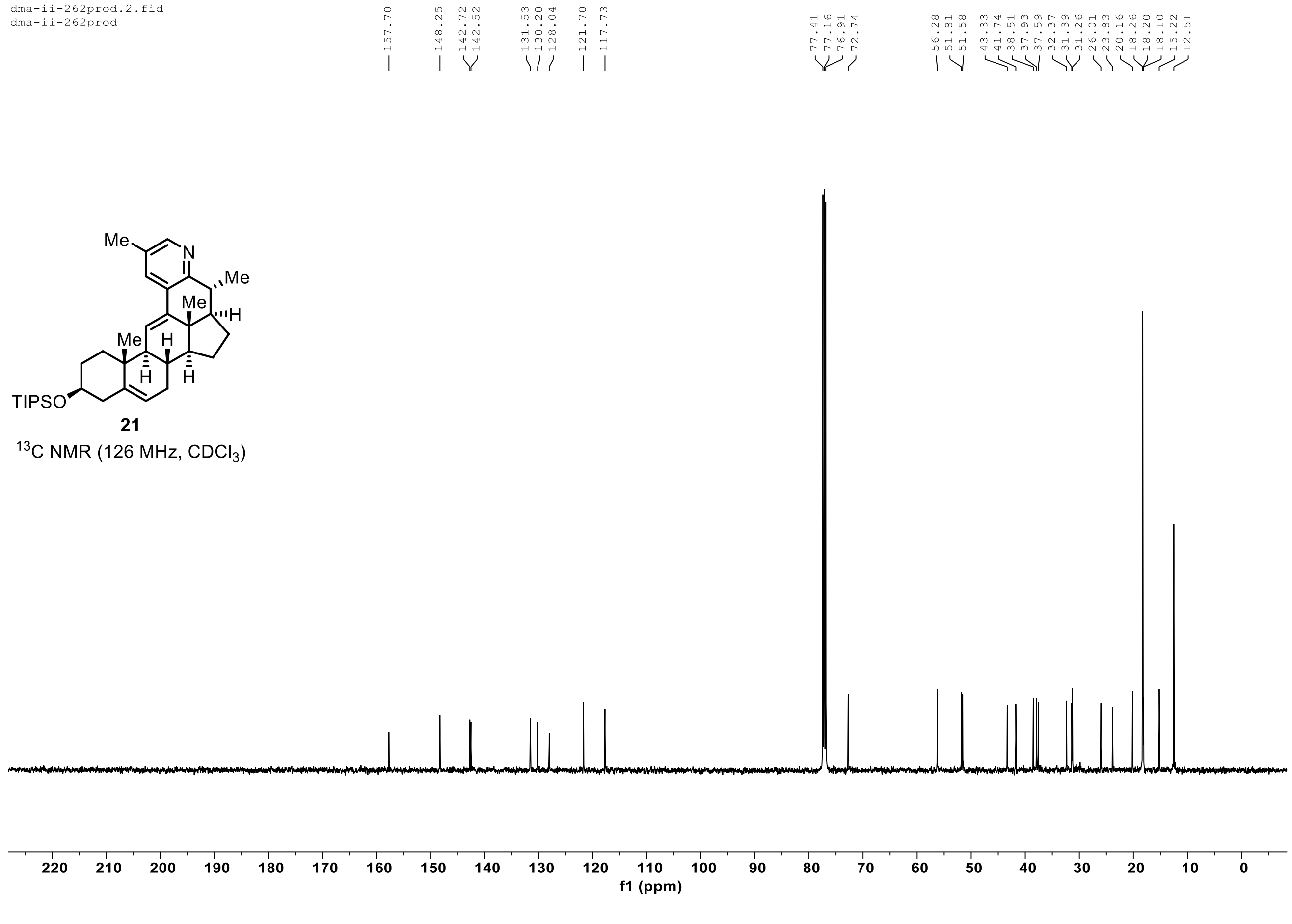
dma-ii-262prod.2.fid
dma-ii-262prod
157.70
148.25
142.72
142.52
131.53
130.20
128.04
121.70
117.73
77.41
77.16
76.91
72.74
56.28
51.81
51.58
43.33
41.74
38.51
37.93
37.59
32.37
31.39
31.26
26.01
23.83
20.16
18.26
18.20
18.10
15.22
12.51
Me
N
Me
Me
H
Me
H
H
H
TIPSO
21
^{13}C NMR (126 MHz, CDCl$_3$)
f1 (ppm)

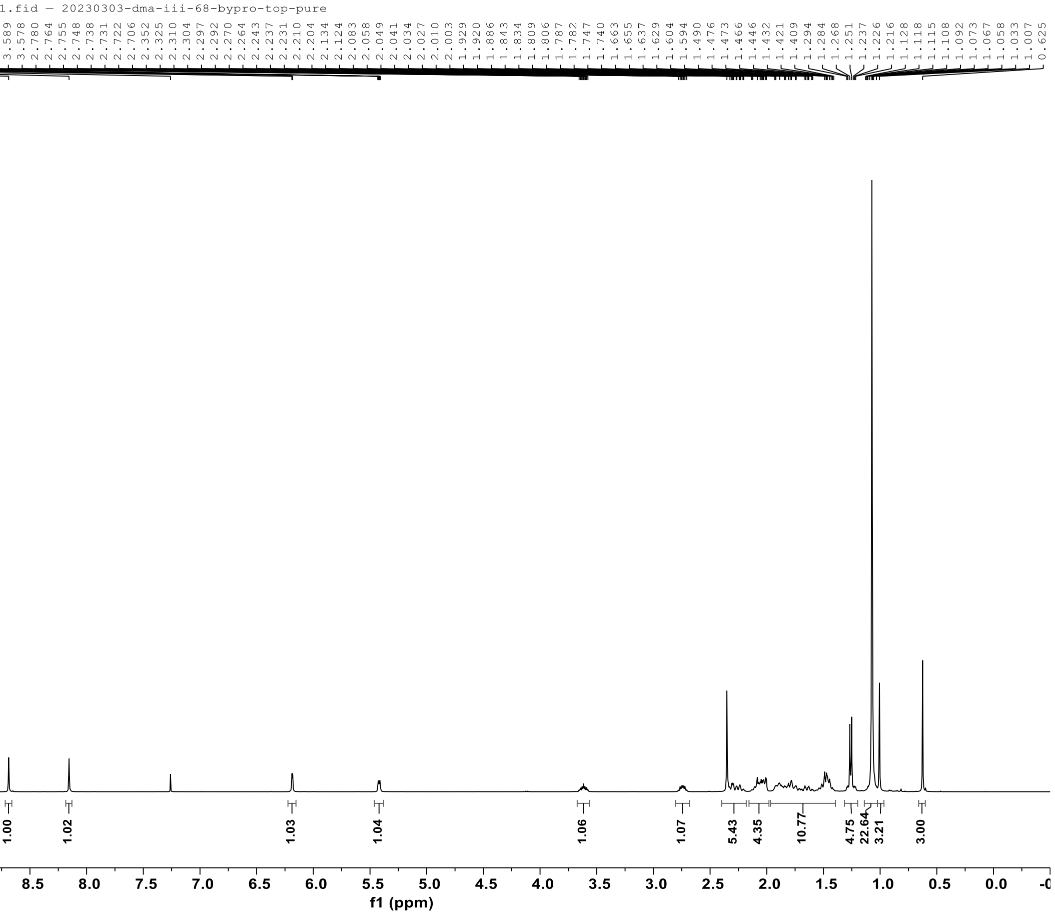

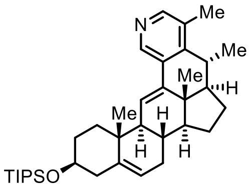

22

^{1}H NMR (400 MHz, $CDCl_3$)

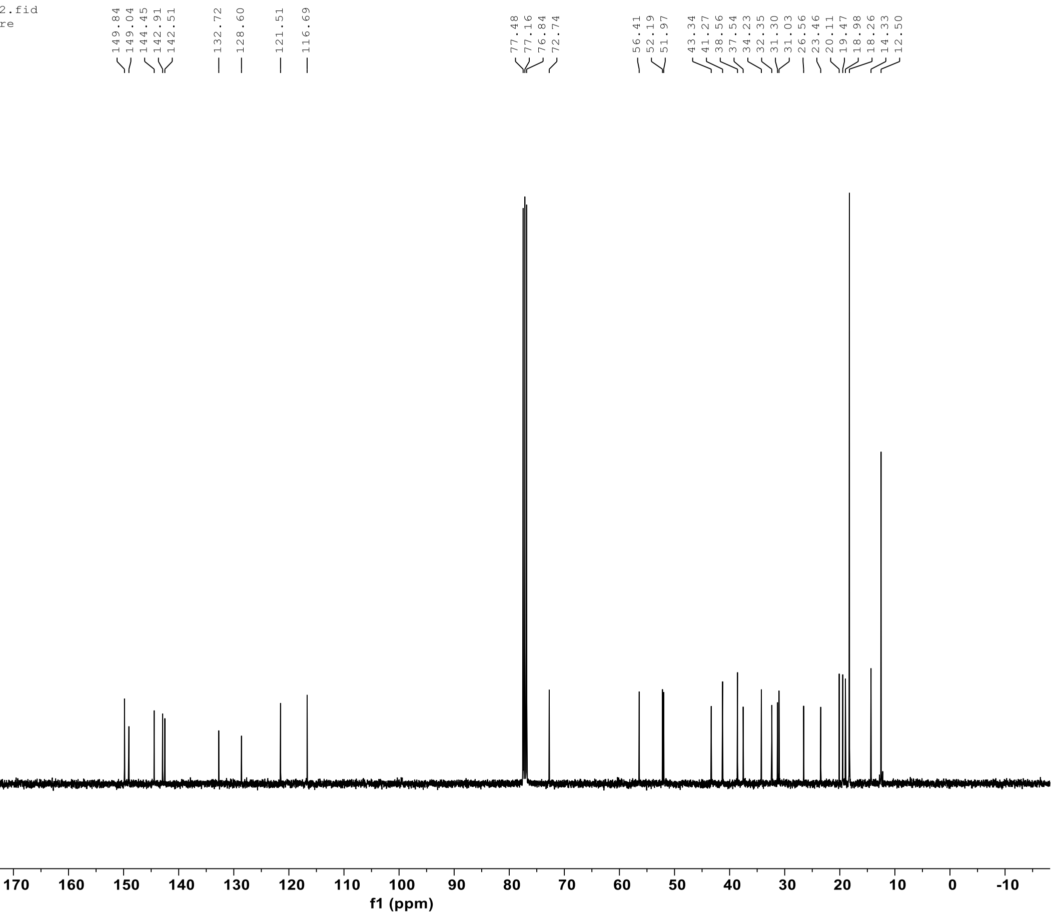

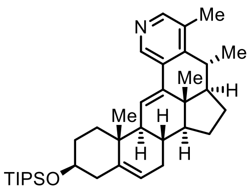

22

^{13}C NMR (101 MHz, $CDCl_3$)

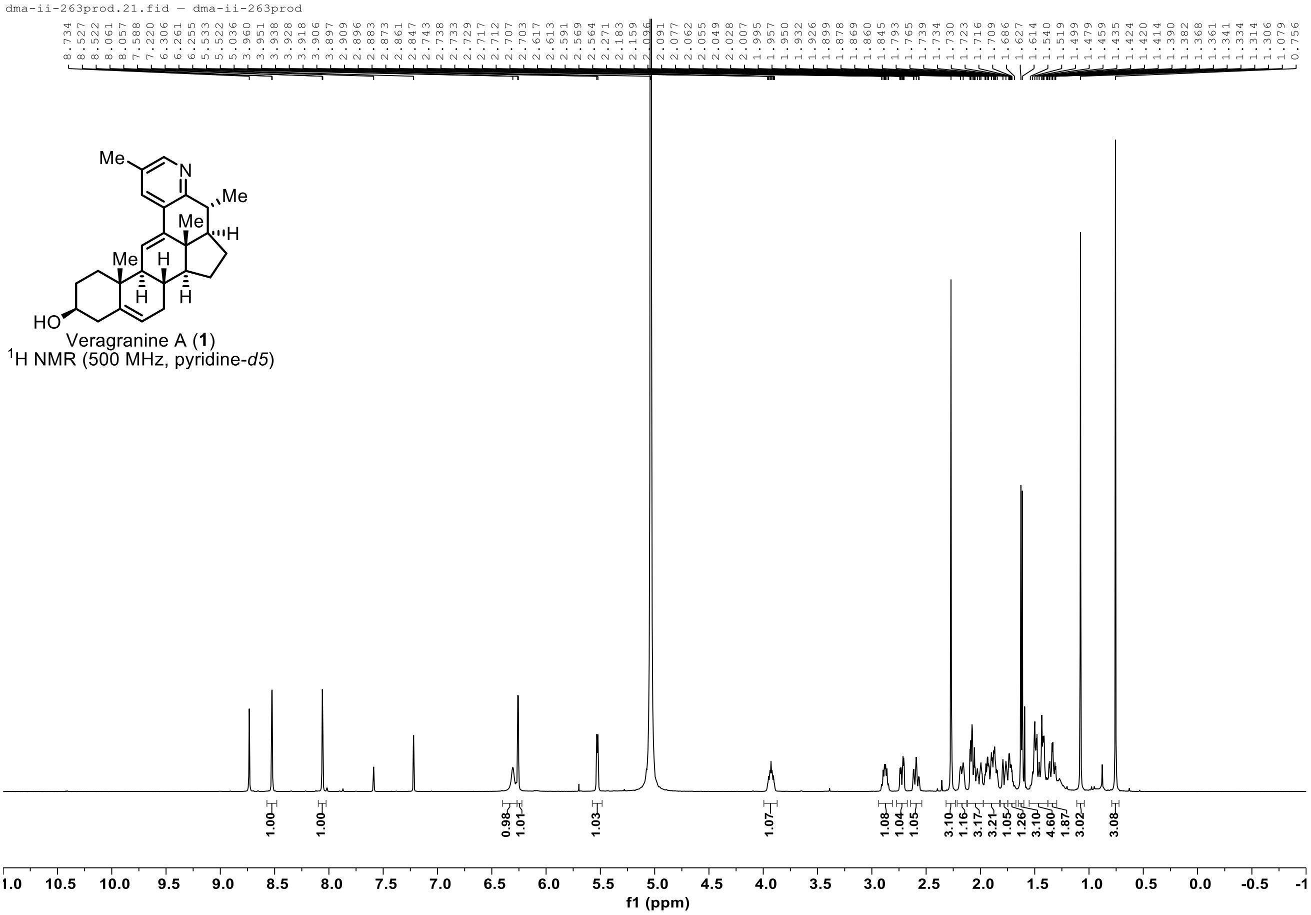
dma-ii-263prod.21.fid — dma-ii-263prod
Me
N
Me
Me
H
Me
H
H
H
HO
Veragranine A (1)
1H NMR (500 MHz, pyridine-d5)
f1 (ppm)

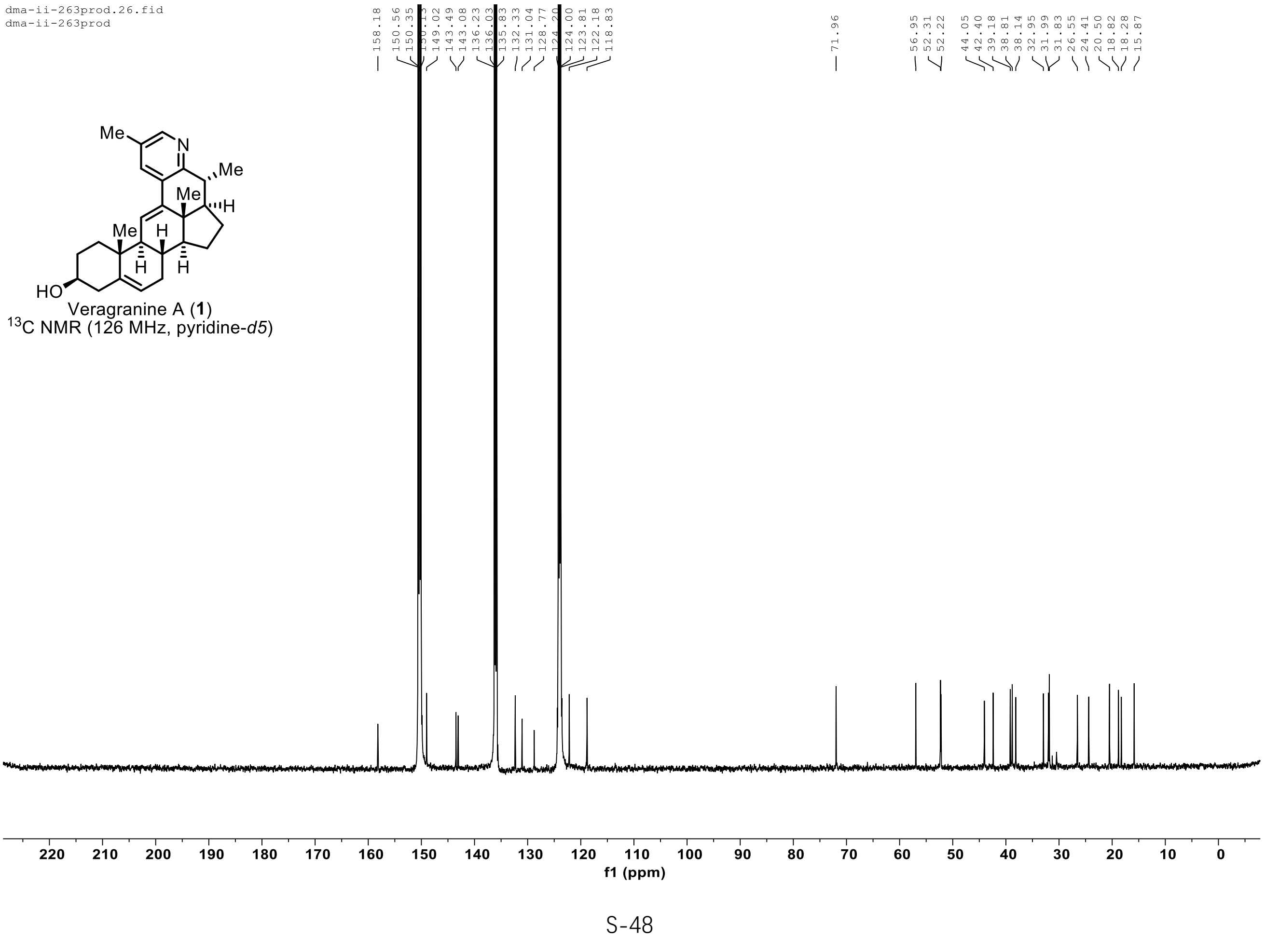
dma-ii-263prod.26.fid
dma-ii-263prod
Me
N
Me
Me
H
Me
H
H
H
HO
Veragranine A (1)
13C NMR (126 MHz, pyridine-d5)
f1 (ppm)

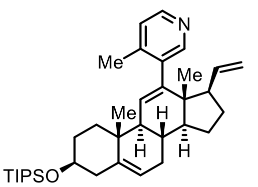

24b

^{1}H NMR (400 MHz, $CDCl_3$)

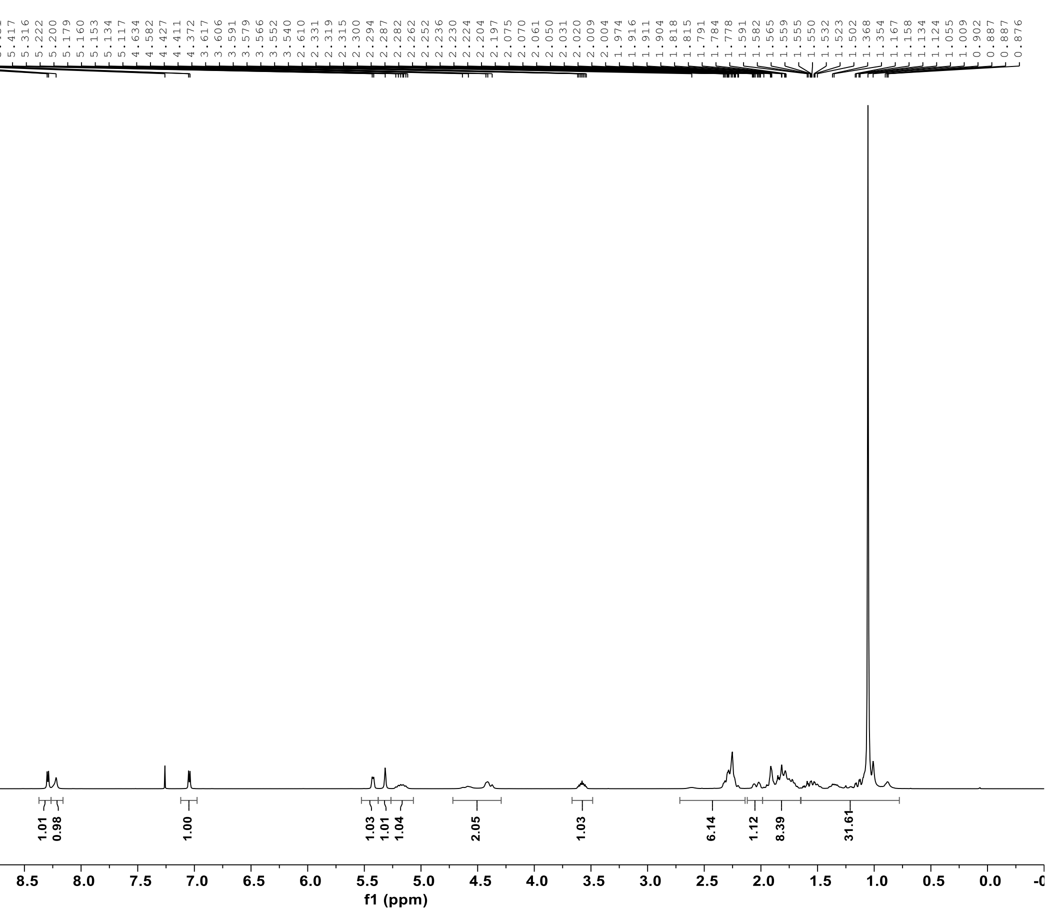

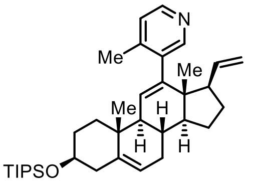

24b

^{13}C NMR (101 MHz, $CDCl_3$)

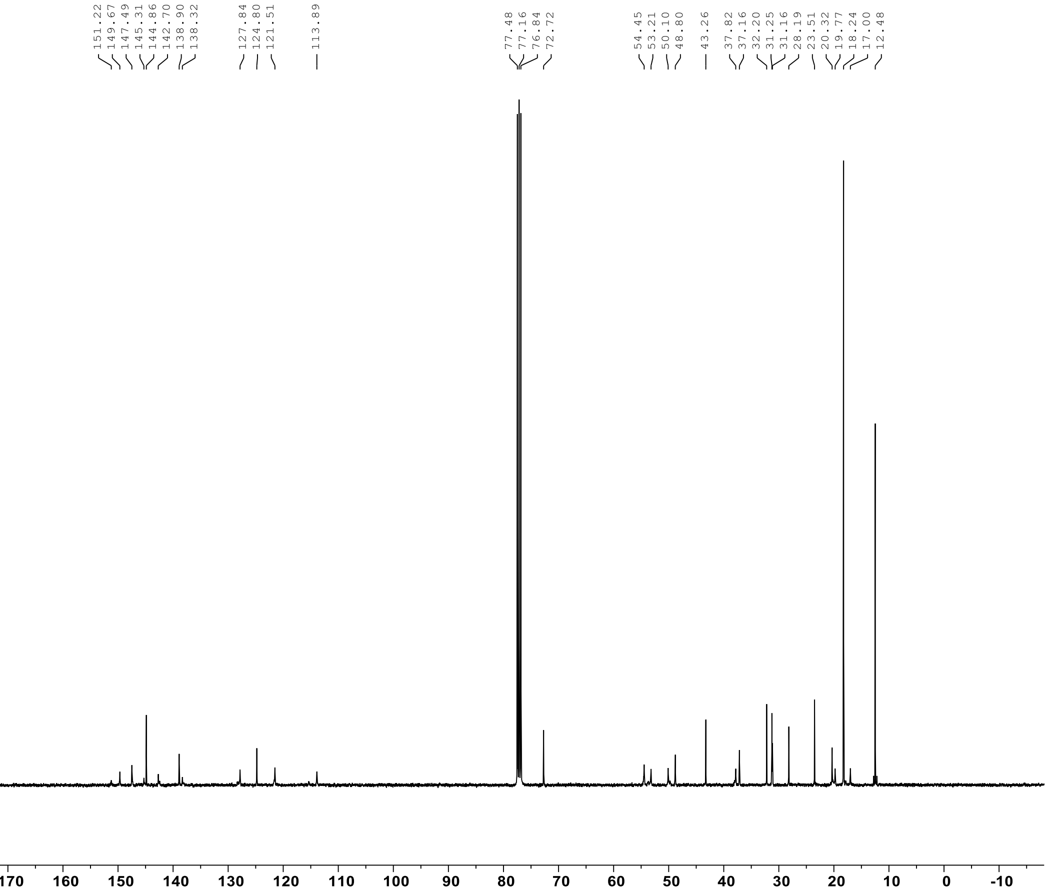

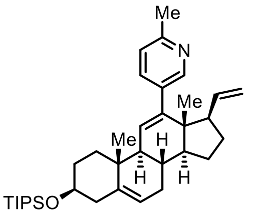

24c

^{1}H NMR (400 MHz, $CDCl_3$)

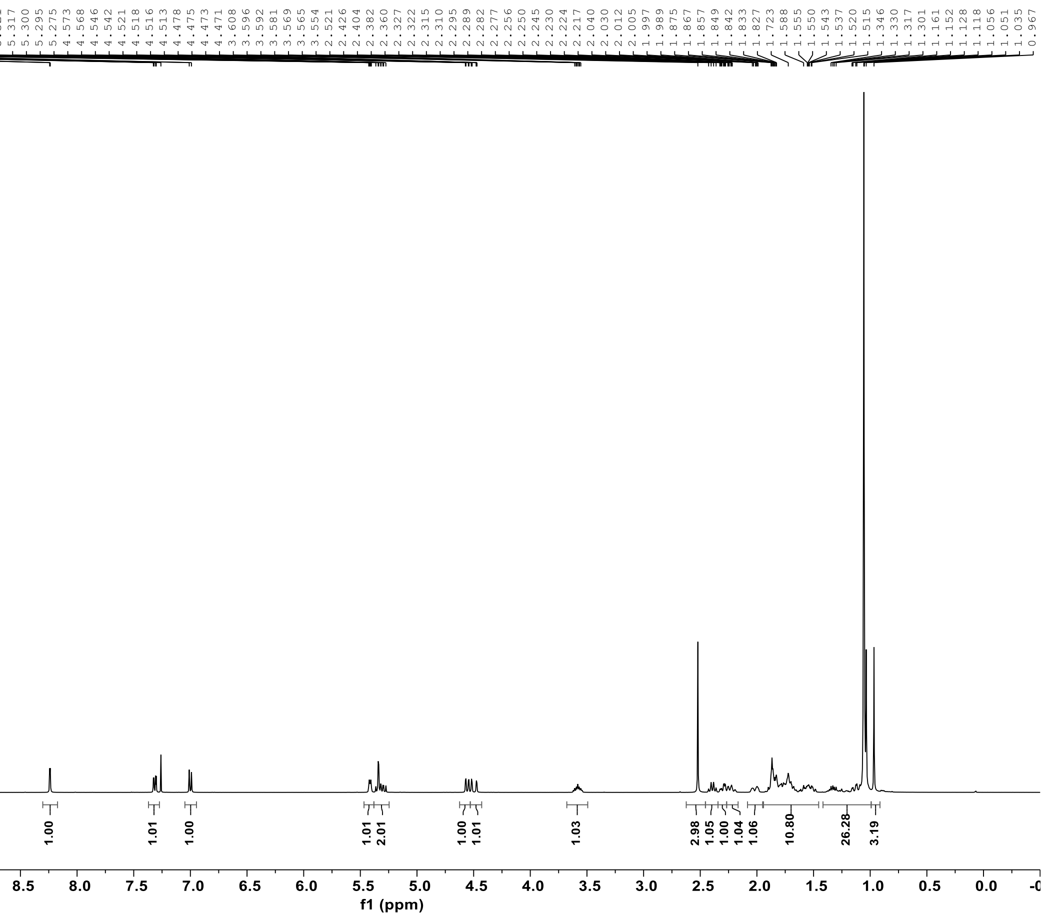

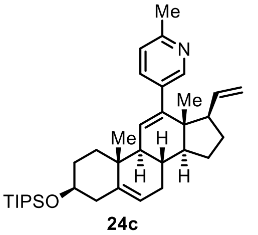

24c

^{13}C NMR (101 MHz, $CDCl_3$)

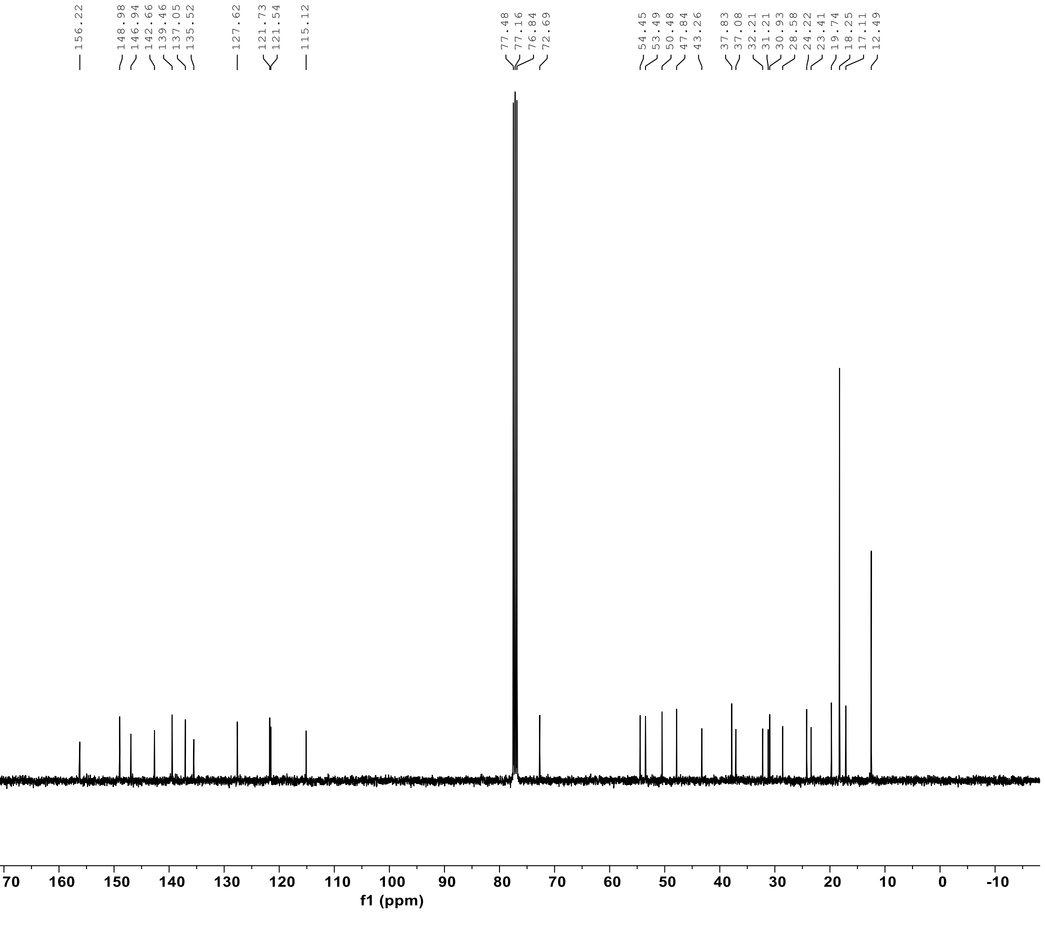

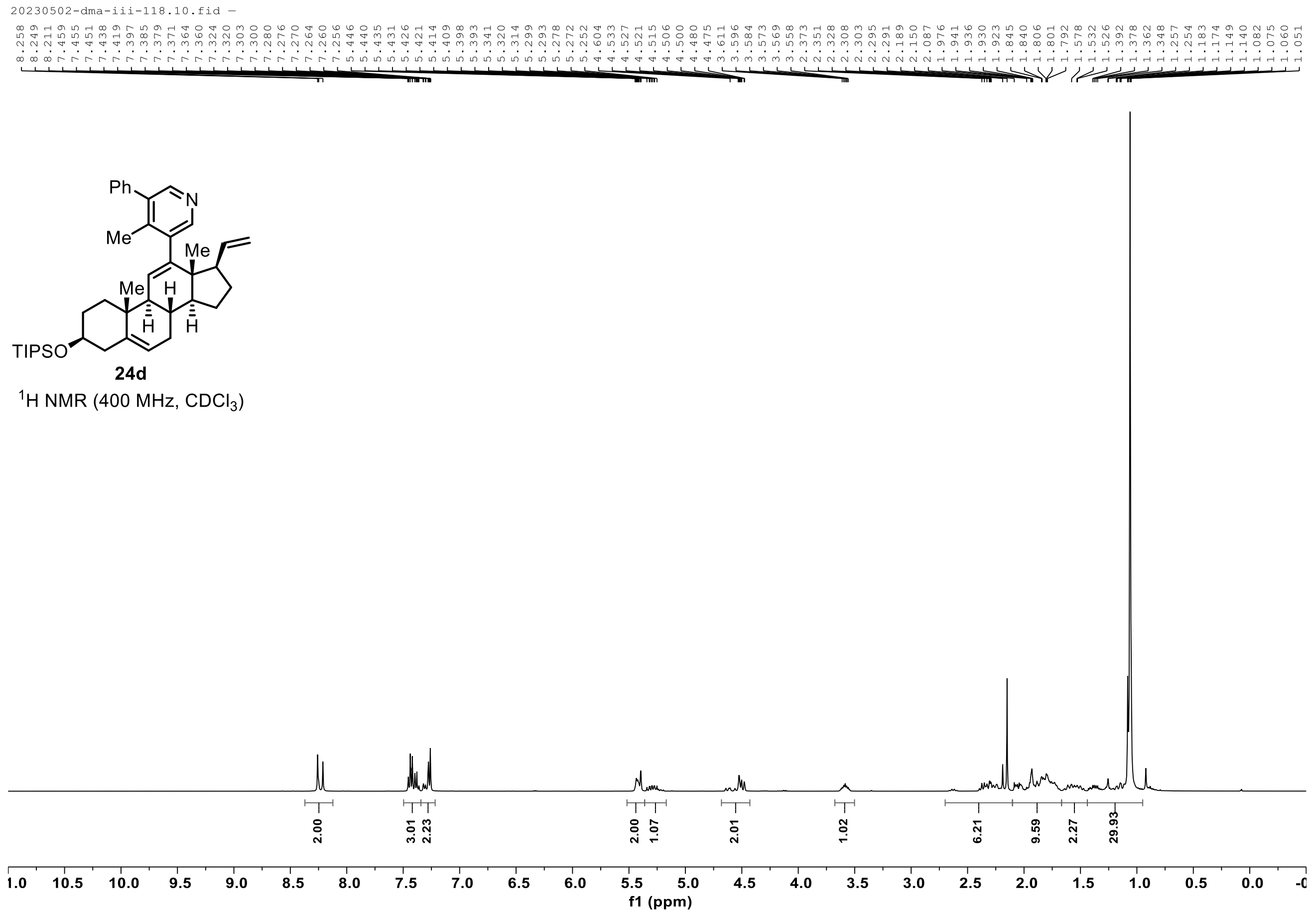

24d
^{1}H NMR (400 MHz, $CDCl_3$)

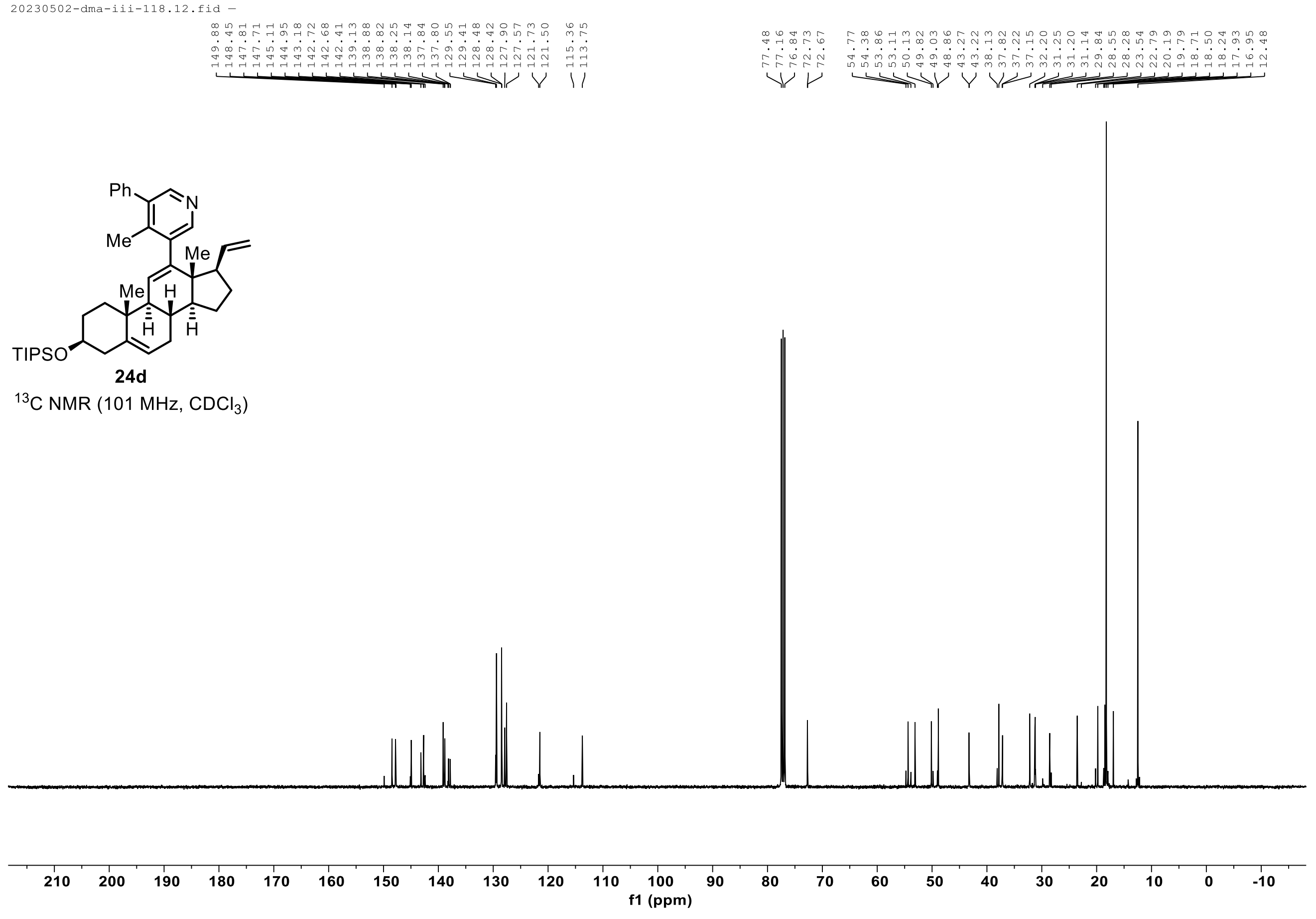

24d
^{13}C NMR (101 MHz, $CDCl_3$)

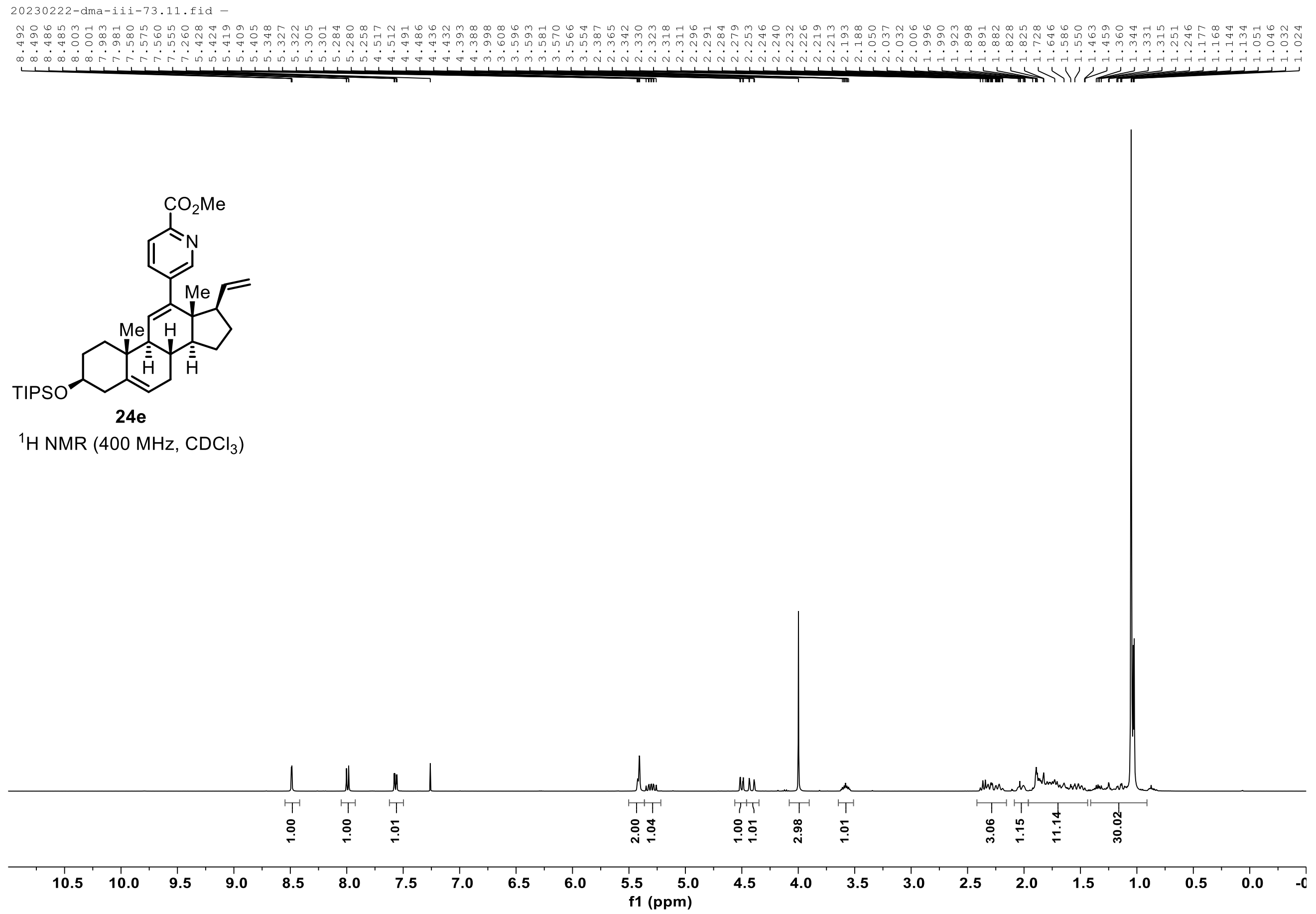
20230222-dma-iii-73.11.fid
8.492 8.490 8.486 8.485 8.003 8.001 7.983 7.981 7.580 7.575 7.560 7.555 7.260 5.428 5.424 5.419 5.409 5.405 5.348 5.327 5.322 5.305 5.301 5.284 5.280 5.258 4.517 4.512 4.491 4.486 4.436 4.432 4.393 4.388 3.998 3.608 3.596 3.593 3.581 3.570 3.566 3.554 2.387 2.365 2.342 2.330 2.323 2.318 2.311 2.296 2.291 2.284 2.279 2.253 2.246 2.240 2.232 2.226 2.219 2.213 2.193 2.188 2.050 2.037 2.032 2.006 1.996 1.990 1.923 1.898 1.891 1.882 1.828 1.825 1.728 1.646 1.586 1.550 1.463 1.459 1.360 1.344 1.331 1.315 1.251 1.246 1.177 1.168 1.144 1.134 1.051 1.046 1.032 1.024
CO2Me
N
Me
Me
H
H
H
TIPSO
24e
1H NMR (400 MHz, CDCl3)
1.00 1.00 1.01 2.00 1.04 1.00 1.01 2.98 1.01 3.06 1.15 11.14 30.02
10.5 10.0 9.5 9.0 8.5 8.0 7.5 7.0 6.5 6.0 5.5 5.0 4.5 4.0 3.5 3.0 2.5 2.0 1.5 1.0 0.5 0.0
f1 (ppm)

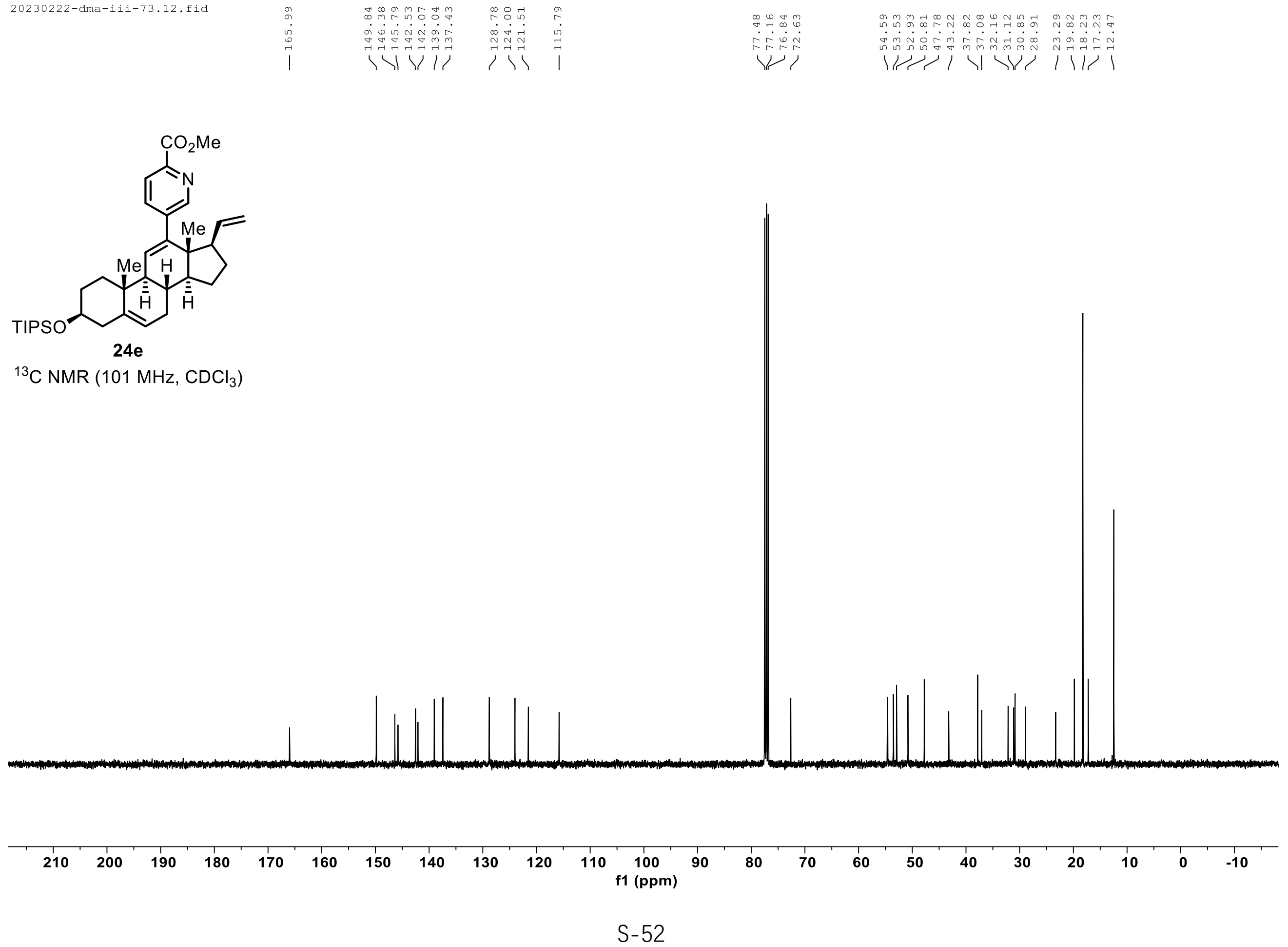
20230222-dma-iii-73.12.fid
165.99
149.84 146.38 145.79 142.53 142.07 139.04 137.43
128.78 124.00 121.51
115.79
77.48 77.16 76.84 72.63
54.59 53.53 52.93 50.81 47.78 43.22 37.82 37.08 32.16 31.12 30.85 28.91 23.29 19.82 18.23 17.23 12.47
CO2Me
N
Me
Me
H
H
H
TIPSO
24e
13C NMR (101 MHz, CDCl3)
210 200 190 180 170 160 150 140 130 120 110 100 90 80 70 60 50 40 30 20 10 0 -10
f1 (ppm)

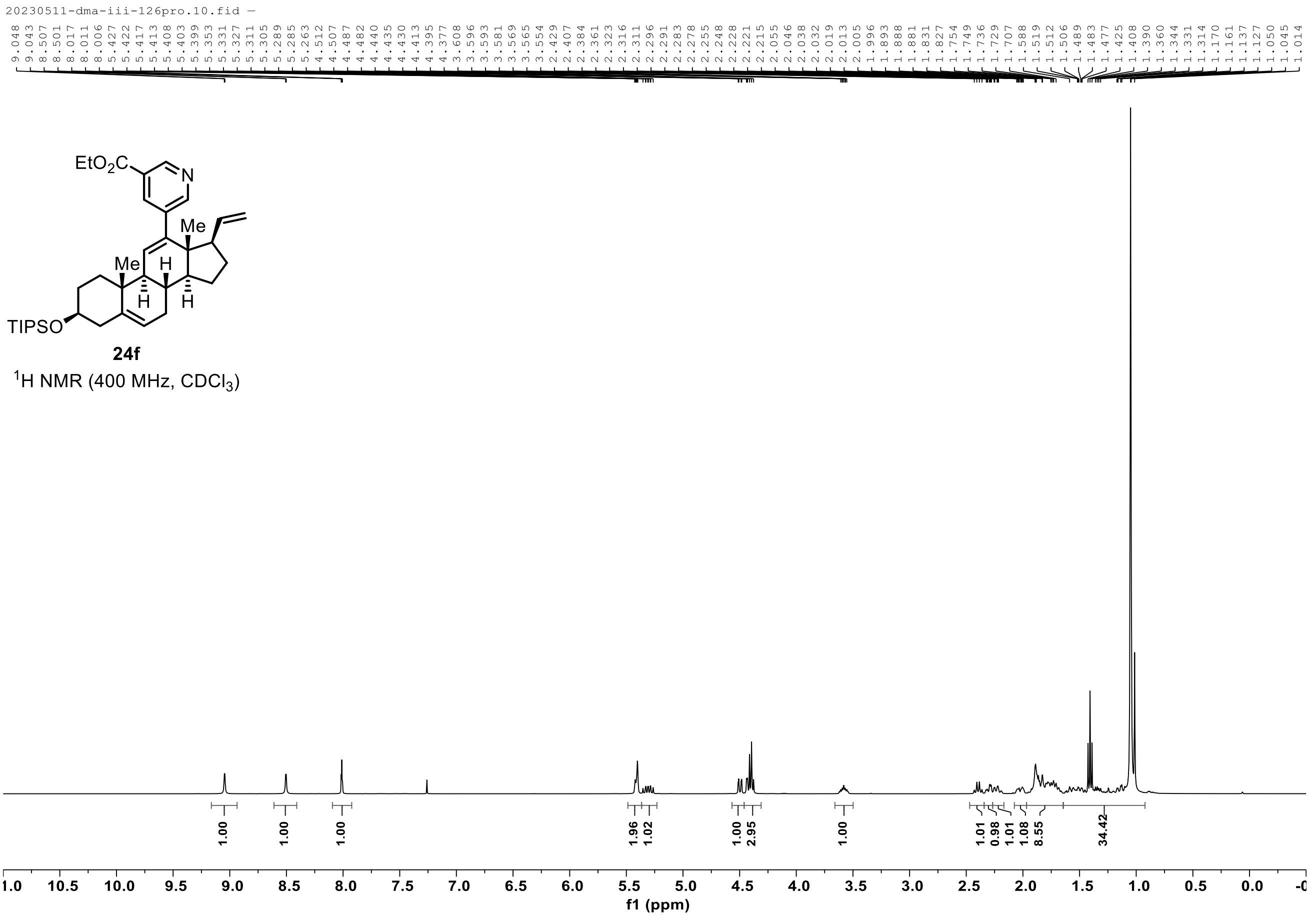
20230511-dma-iii-126pro.10.fid
9.048 9.043 8.507 8.501 8.017 8.011 8.006 5.427 5.422 5.417 5.413 5.408 5.403 5.399 5.353 5.331 5.327 5.311 5.305 5.289 5.285 5.263 4.512 4.507 4.487 4.482 4.440 4.435 4.430 4.413 4.395 4.377 3.608 3.596 3.593 3.581 3.569 3.565 3.554 2.429 2.407 2.384 2.361 2.323 2.316 2.311 2.296 2.291 2.283 2.278 2.255 2.248 2.228 2.221 2.215 2.055 2.046 2.038 2.032 2.019 2.013 2.005 1.996 1.893 1.888 1.881 1.831 1.827 1.754 1.749 1.736 1.729 1.707 1.588 1.519 1.512 1.506 1.489 1.483 1.477 1.425 1.408 1.390 1.360 1.344 1.331 1.314 1.170 1.161 1.137 1.127 1.050 1.045 1.014
EtO2C
N
Me
Me
H
H
H
TIPSO
24f
1H NMR (400 MHz, CDCl3)
1.00 1.00 1.00 1.96 1.02 1.00 2.95 1.00 1.01 0.98 1.01 1.08 8.55 34.42
1.0 10.5 10.0 9.5 9.0 8.5 8.0 7.5 7.0 6.5 6.0 5.5 5.0 4.5 4.0 3.5 3.0 2.5 2.0 1.5 1.0 0.5 0.0 -0
f1 (ppm)

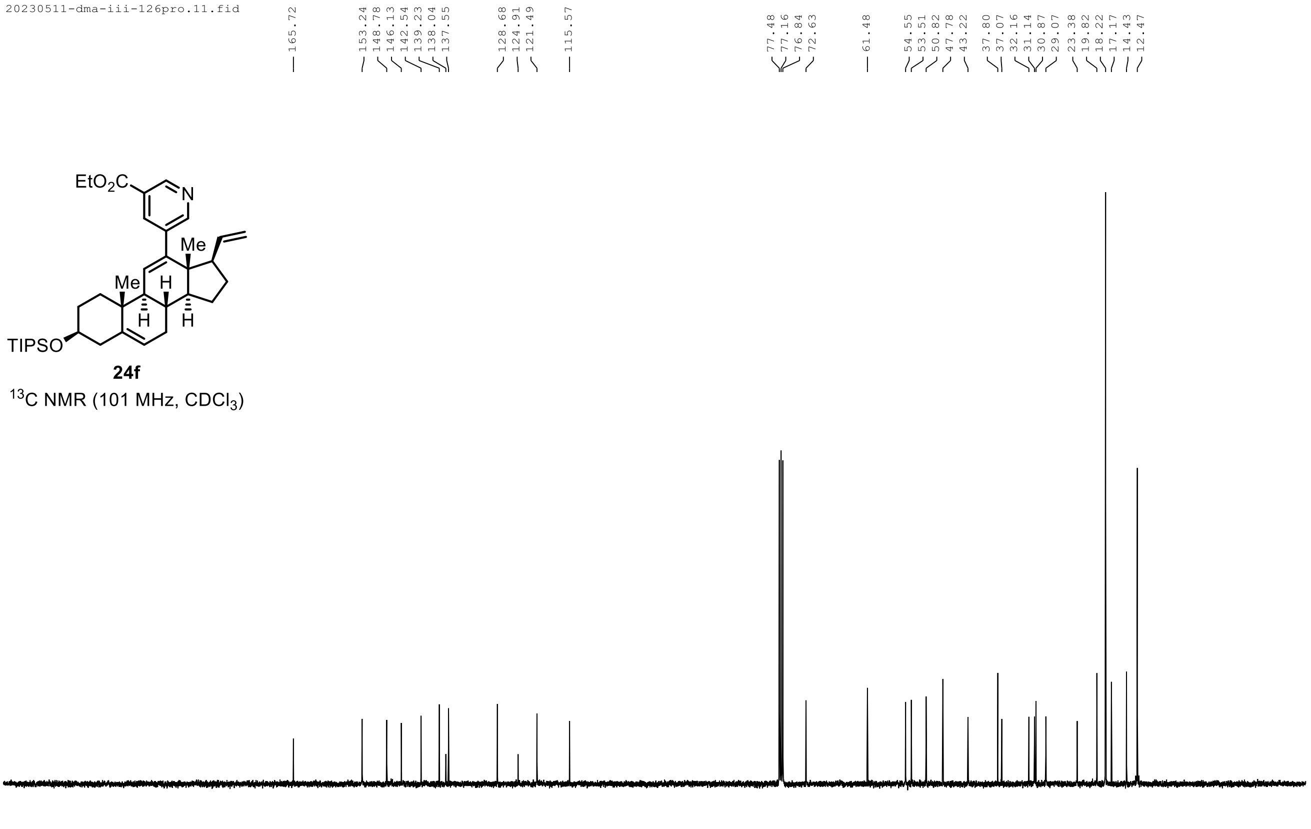
20230511-dma-iii-126pro.11.fid
165.72 153.24 148.78 146.13 142.54 139.23 138.04 137.55 128.68 124.91 121.49 115.57 77.48 77.16 76.84 72.63 61.48 54.55 53.51 50.82 47.78 43.22 37.80 37.07 32.16 31.14 30.87 29.07 23.38 19.82 18.22 17.17 14.43 12.47
EtO2C
N
Me
Me
H
H
H
TIPSO
24f
13C NMR (101 MHz, CDCl3)
210 200 190 180 170 160 150 140 130 120 110 100 90 80 70 60 50 40 30 20 10 0 -10
f1 (ppm)

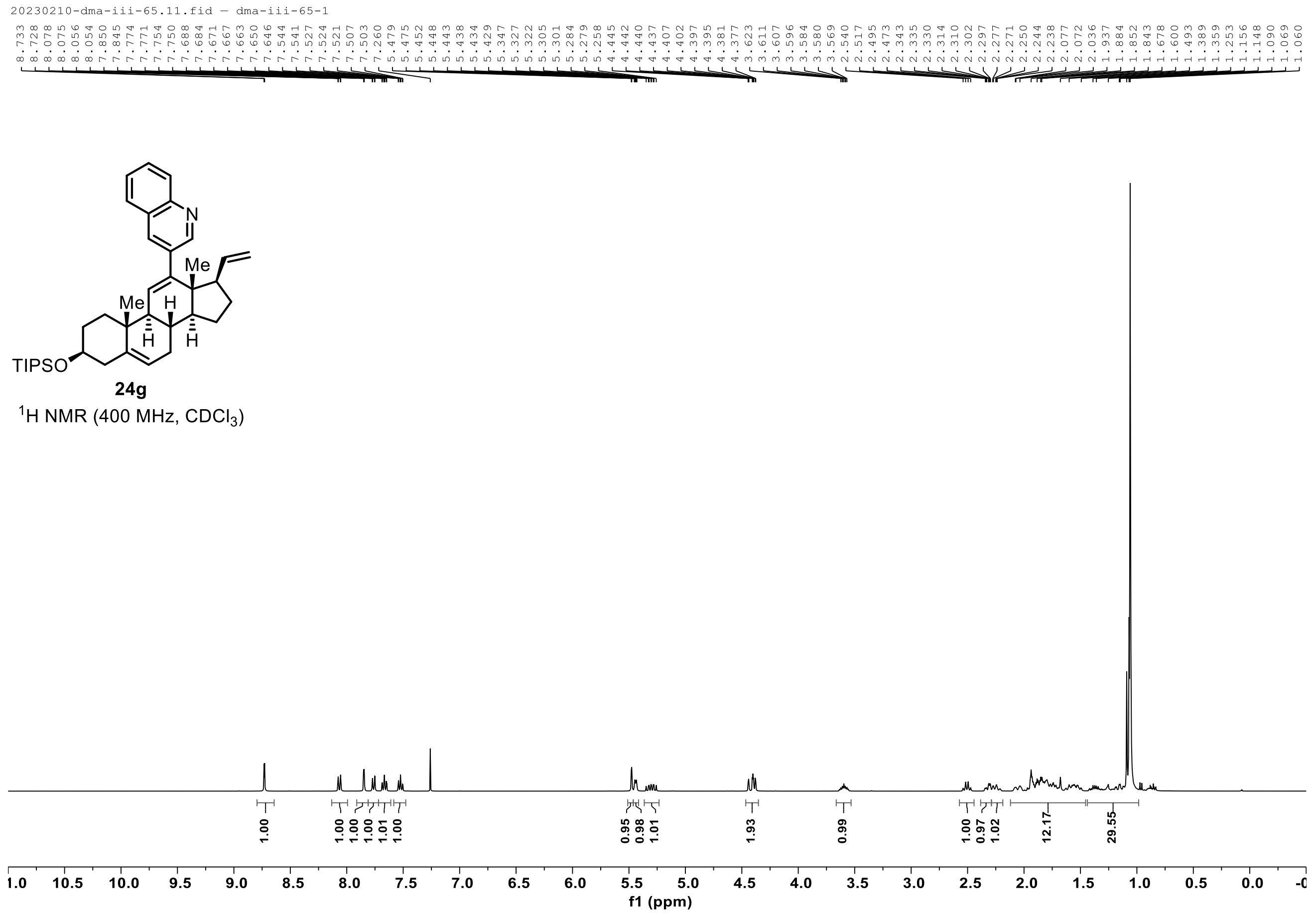
20230210-dma-iii-65.11.fid — dma-iii-65-1
8.733 8.728 8.075 8.056 8.054 7.850 7.845 7.774 7.771 7.754 7.750 7.688 7.684 7.671 7.667 7.663 7.650 7.646 7.544 7.541 7.527 7.524 7.521 7.507 7.503 7.260 5.479 5.475 5.452 5.448 5.443 5.438 5.434 5.429 5.347 5.327 5.322 5.305 5.301 5.284 5.279 5.258 4.445 4.442 4.440 4.437 4.407 4.402 4.397 4.395 4.381 4.377 3.623 3.611 3.607 3.596 3.584 3.580 3.569 2.540 2.517 2.495 2.473 2.343 2.335 2.330 2.314 2.310 2.302 2.297 2.277 2.271 2.250 2.244 2.238 2.077 2.072 2.036 1.937 1.884 1.852 1.843 1.678 1.600 1.493 1.389 1.359 1.253 1.156 1.148 1.090 1.069 1.060
N
Me
Me
H
H
H
TIPSO
24g
1H NMR (400 MHz, CDCl3)
1.00 1.00 1.00 1.00 1.01 1.00 0.95 0.98 1.01 1.93 0.99 1.00 0.97 1.02 12.17 29.55
f1 (ppm)

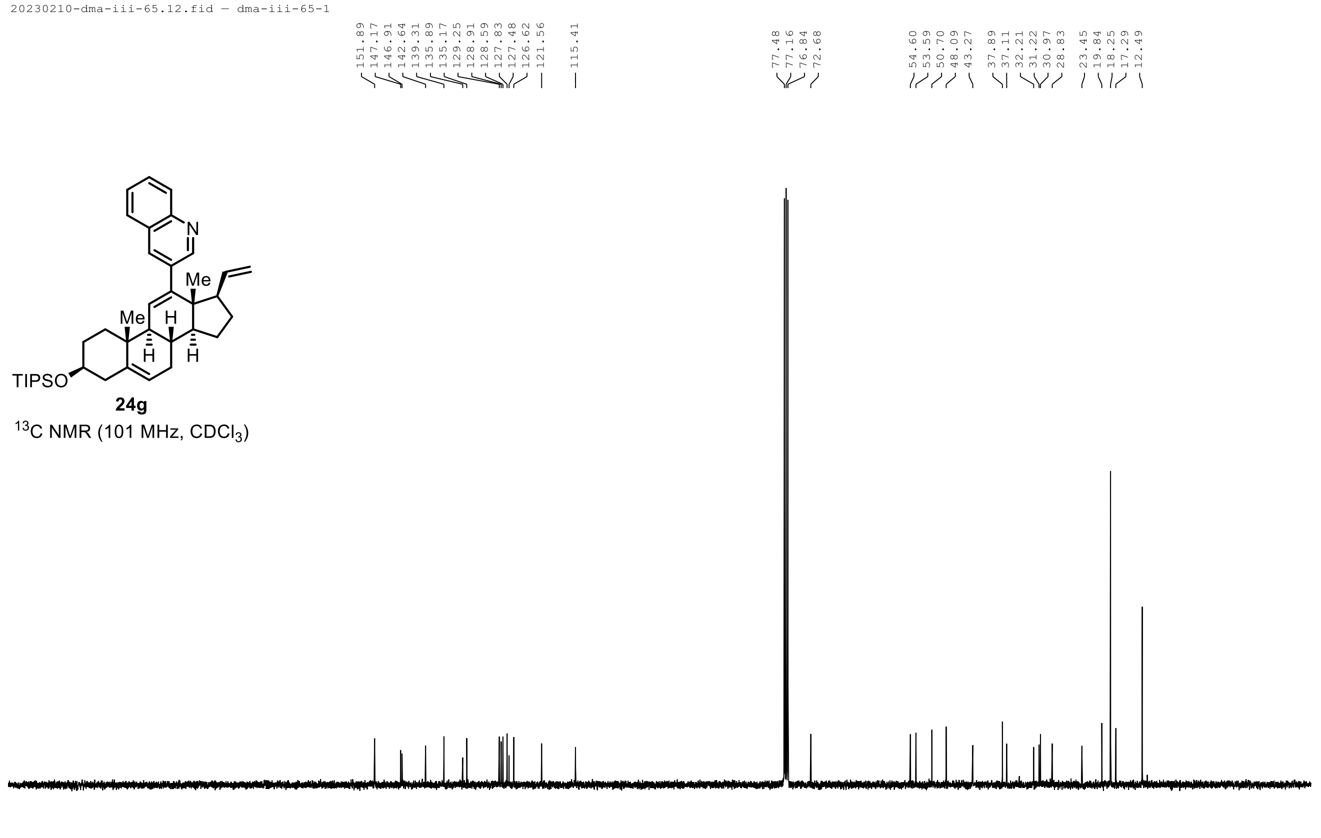
20230210-dma-iii-65.12.fid — dma-iii-65-1
151.89 147.17 146.91 142.64 139.31 135.89 135.17 129.25 128.91 128.59 127.83 127.48 126.62 121.56 115.41
77.48 77.16 76.84 72.68
54.60 53.59 50.70 48.09 43.27 37.89 37.11 32.21 31.22 30.97 28.83 23.45 19.84 18.25 17.29 12.49
N
Me
Me
H
H
H
TIPSO
24g
13C NMR (101 MHz, CDCl3)
f1 (ppm)

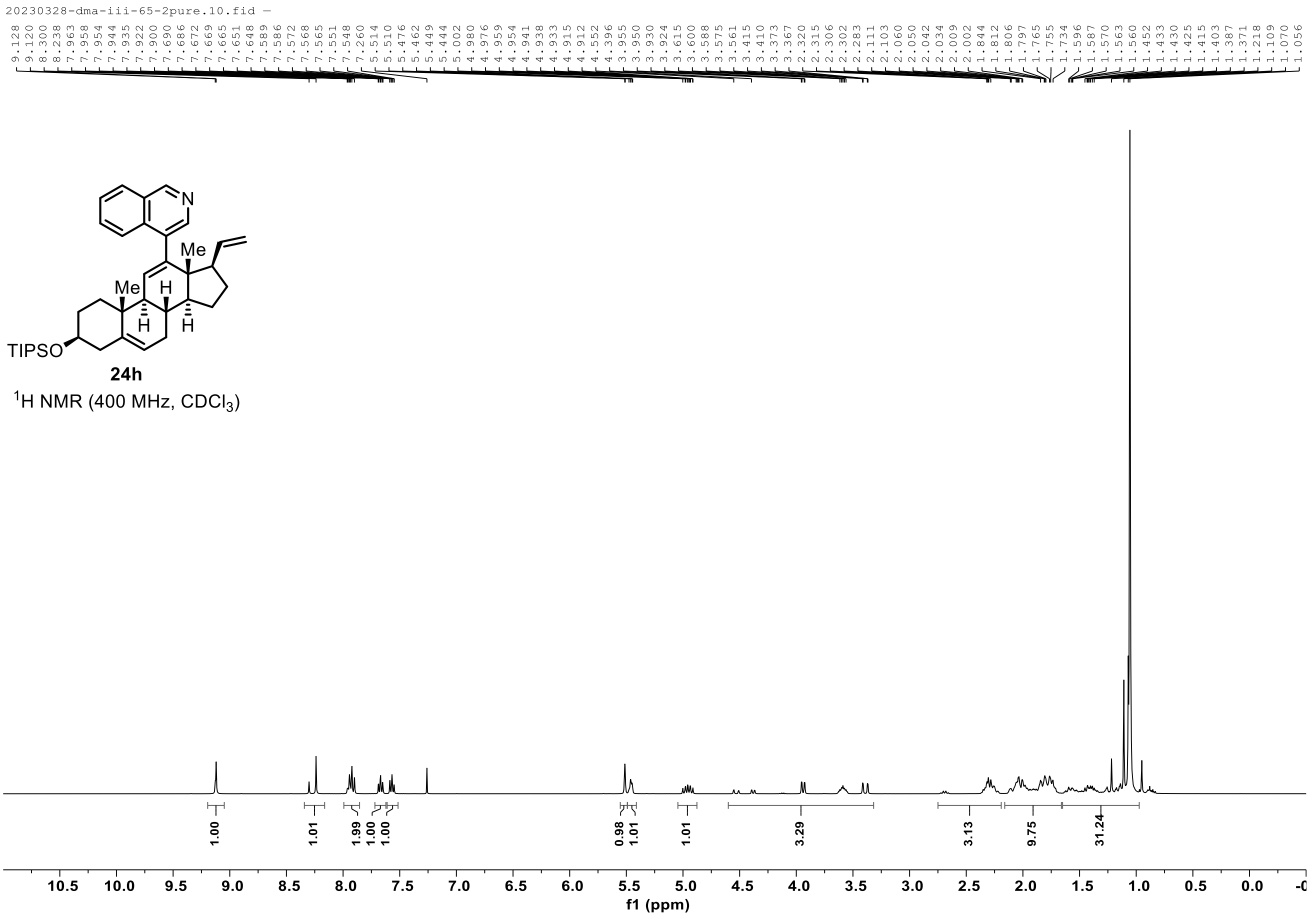

24h

^{1}H NMR (400 MHz, $CDCl_3$)

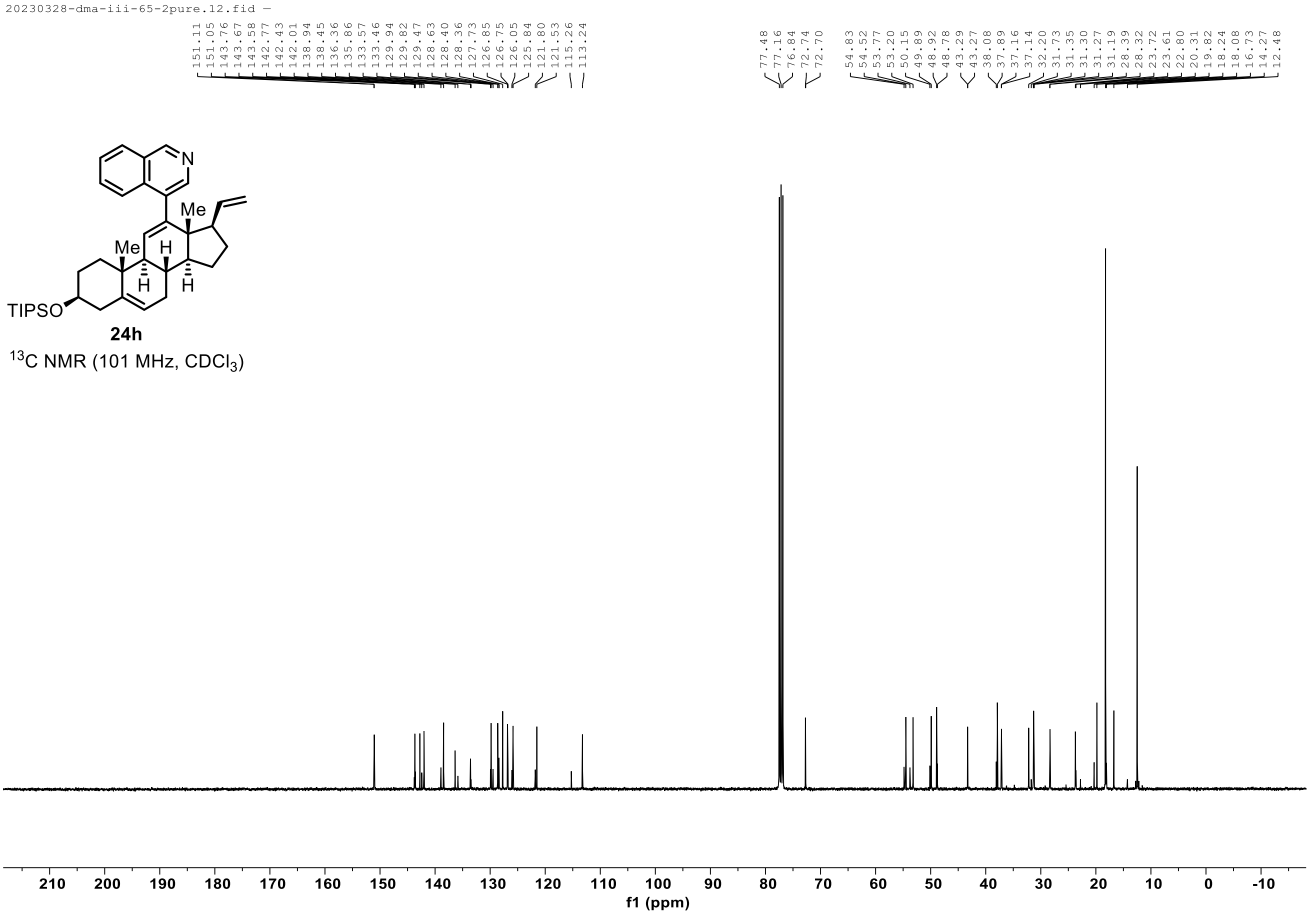

24h

^{13}C NMR (101 MHz, $CDCl_3$)

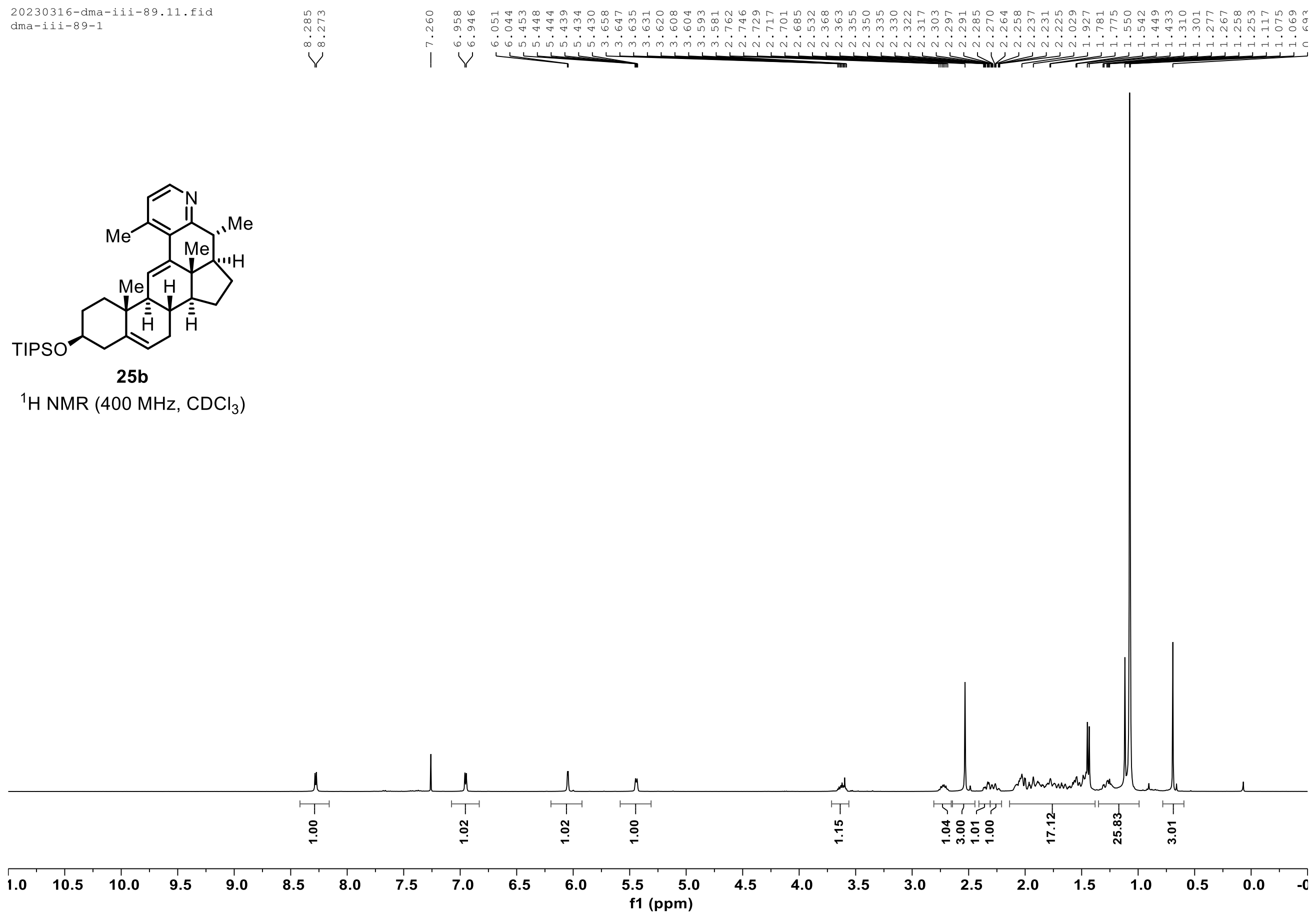

25b

^{1}H NMR (400 MHz, $CDCl_3$)

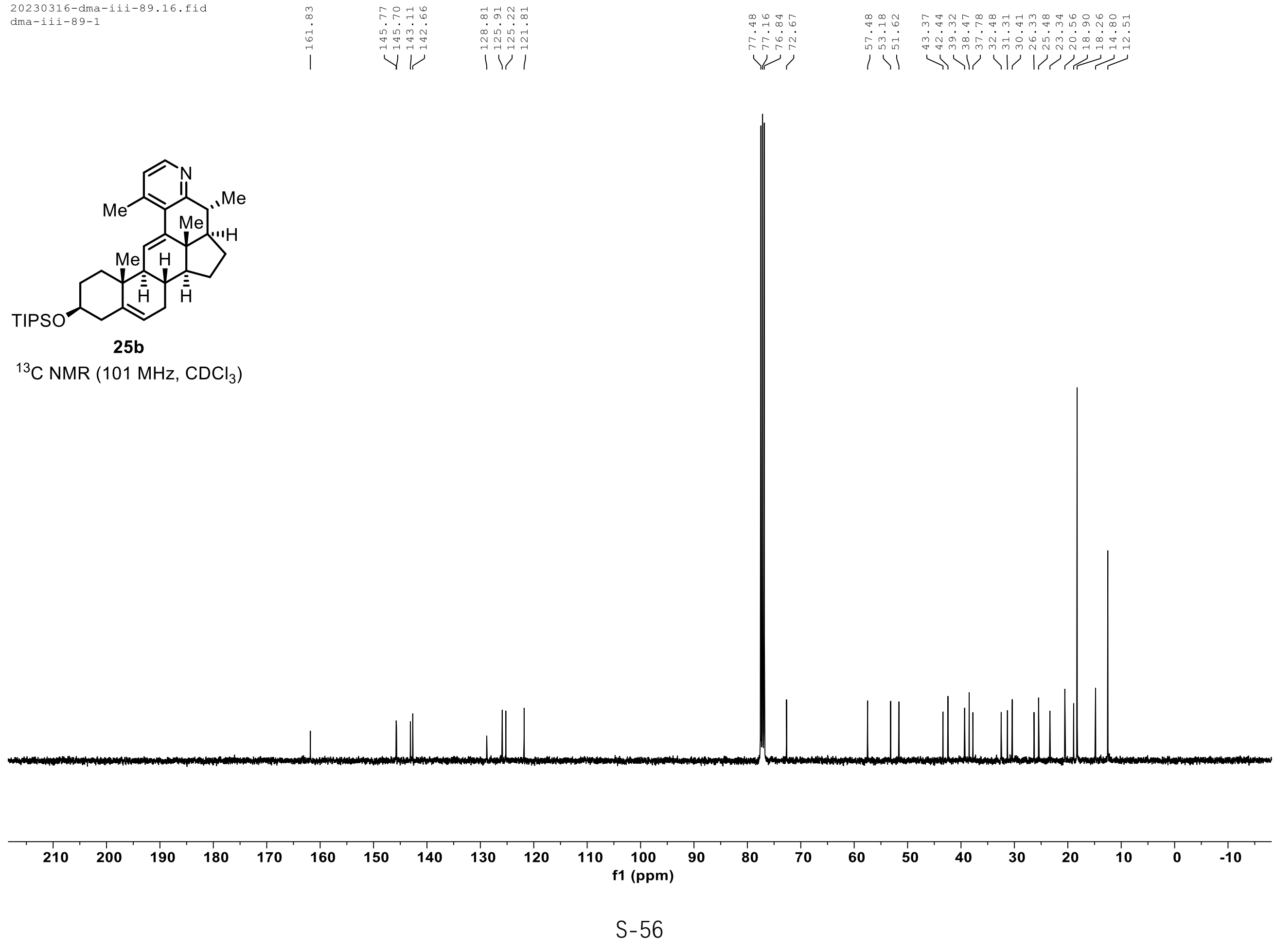

25b

^{13}C NMR (101 MHz, $CDCl_3$)

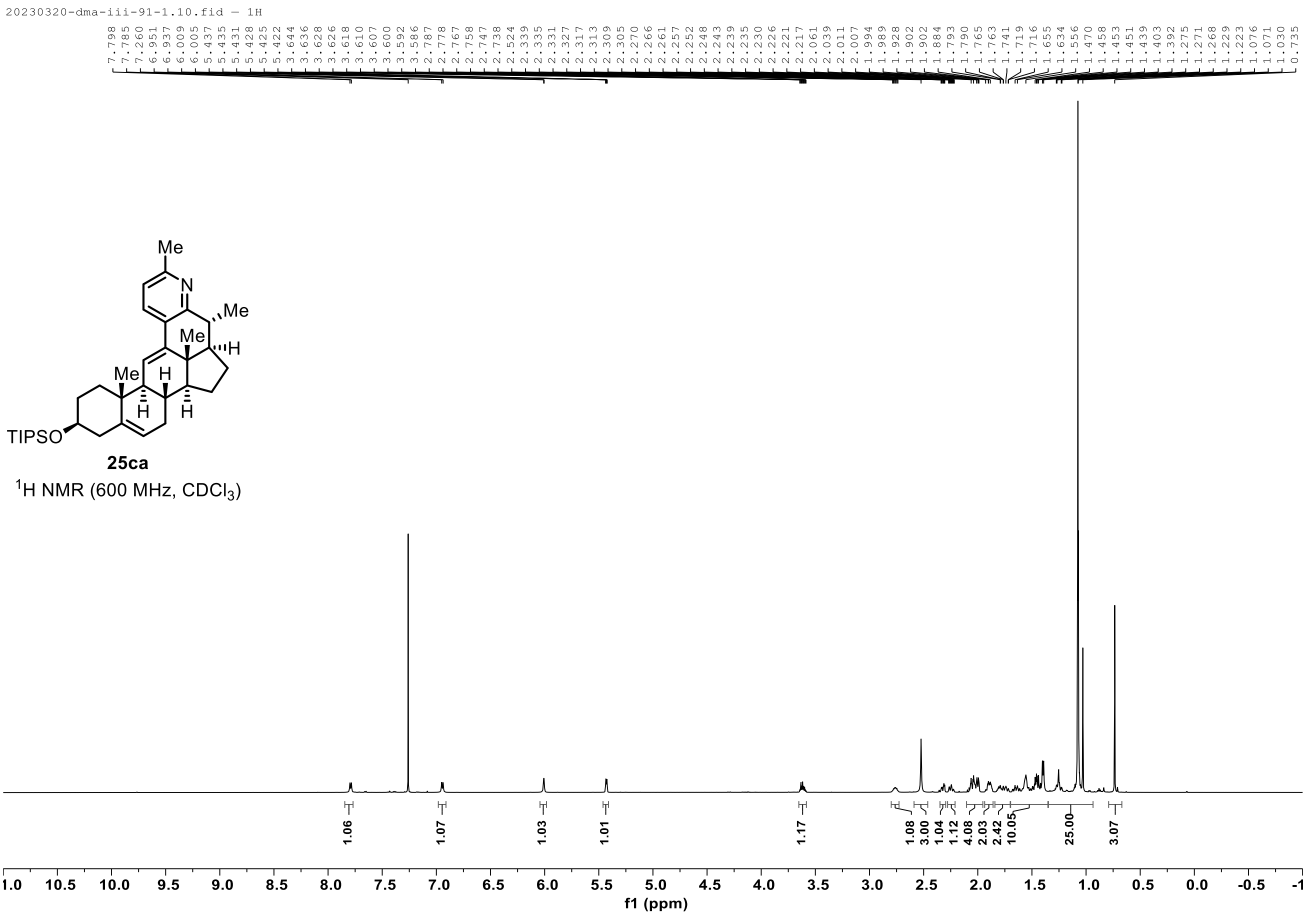

20230320-dma-iii-91-1.10.fid — 1H
Me
N
Me
Me
H
Me
H
H
H
TIPSO
25ca
¹H NMR (600 MHz, CDCl₃)
1.06
1.07
1.03
1.01
1.17
1.08
3.00
1.04
1.12
4.08
2.03
2.42
10.05
25.00
3.07
f1 (ppm)

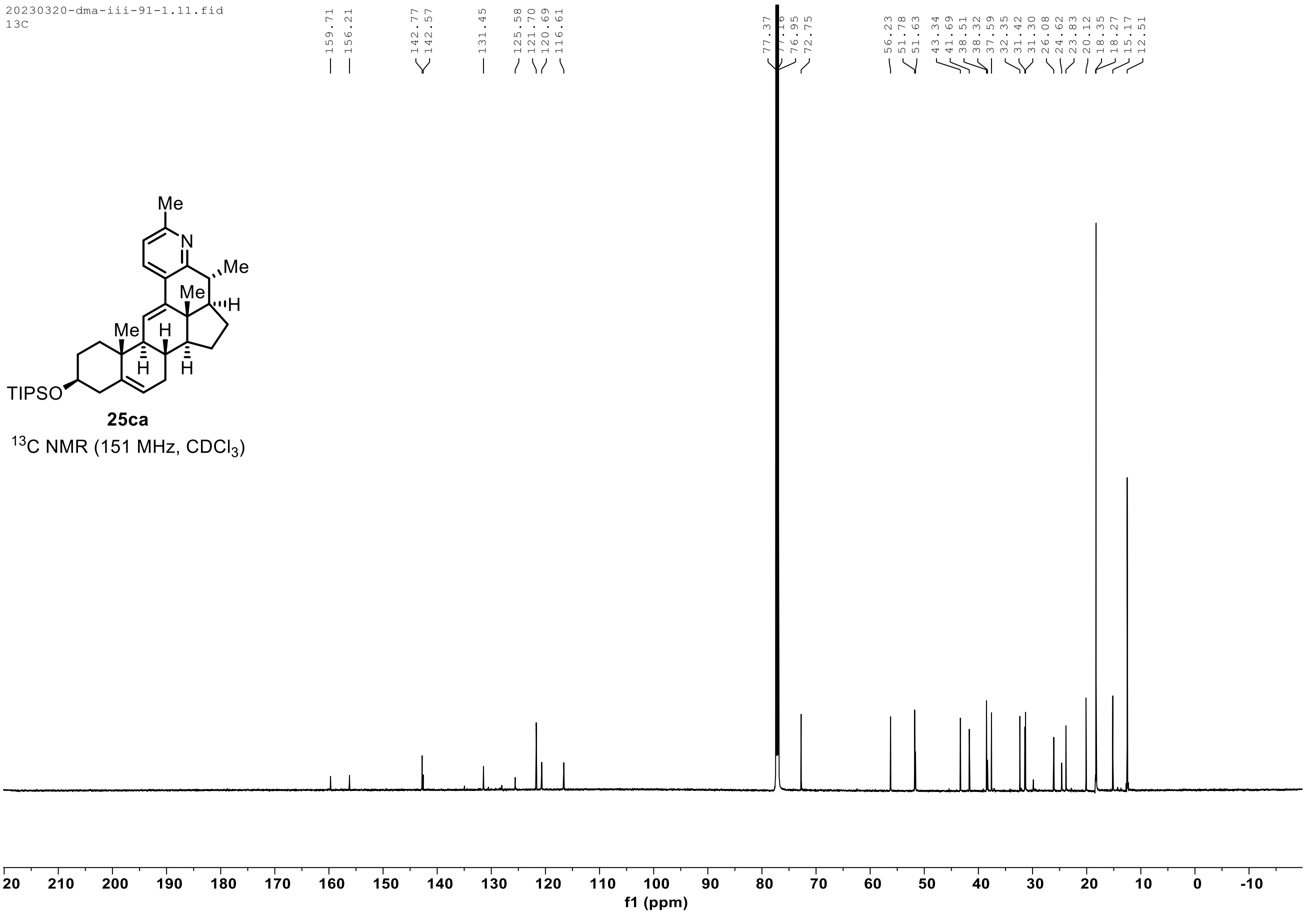

20230320-dma-iii-91-1.11.fid
13C
159.71
156.21
142.77
142.57
131.45
125.58
121.70
120.69
116.61
77.37
77.16
76.95
72.75
56.23
51.78
51.63
43.34
41.69
38.51
38.32
37.59
32.35
31.42
31.30
26.08
24.62
23.83
20.12
18.35
18.27
15.17
12.51
Me
N
Me
Me
H
Me
H
H
H
TIPSO
25ca
¹³C NMR (151 MHz, CDCl₃)
f1 (ppm)

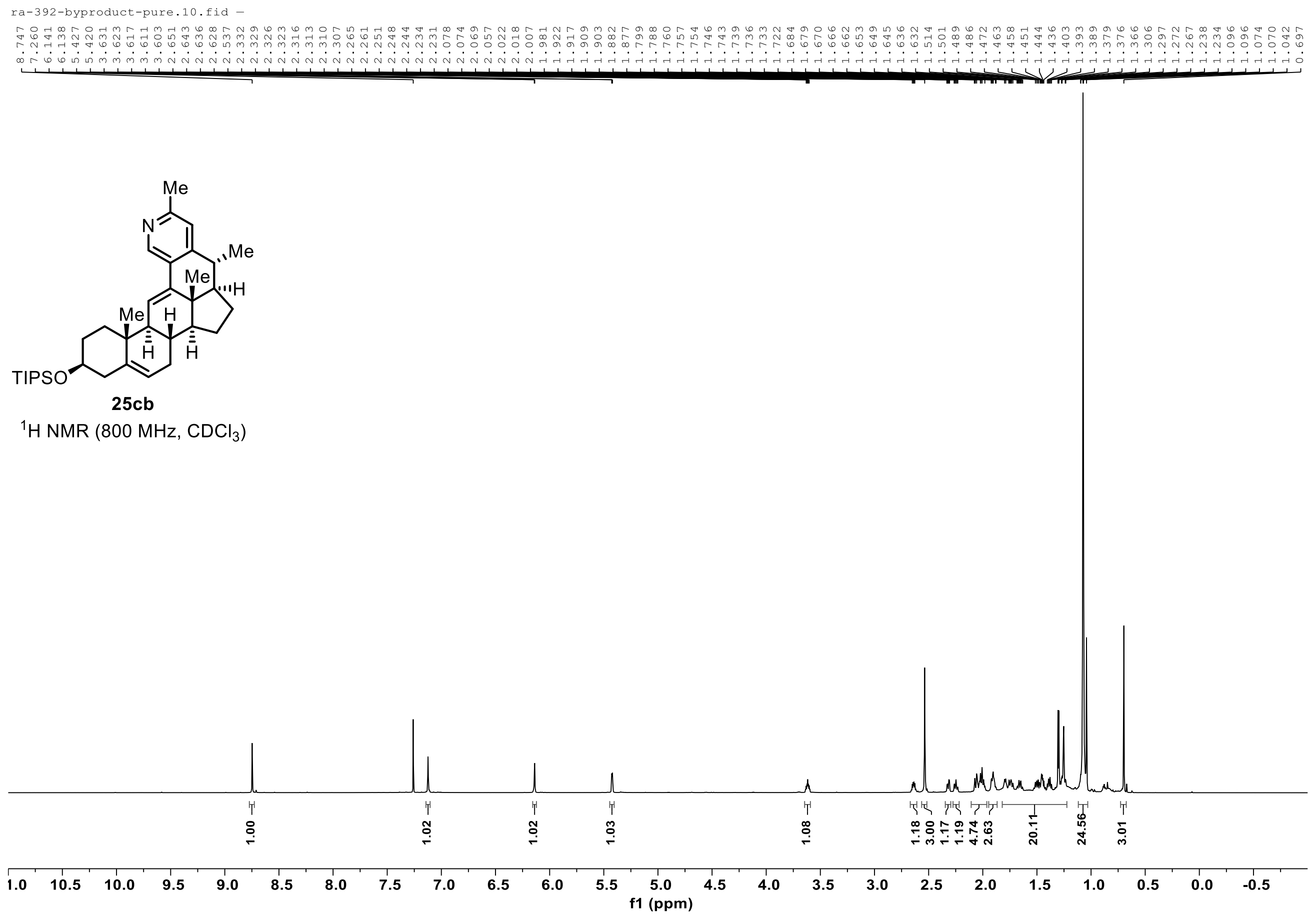
ra-392-byproduct-pure.10.fid
8.747 7.260 6.141 6.138 5.427 5.420 3.631 3.623 3.617 3.611 3.603 2.651 2.643 2.636 2.628 2.537 2.332 2.329 2.326 2.323 2.316 2.313 2.310 2.307 2.265 2.261 2.251 2.248 2.244 2.234 2.231 2.078 2.074 2.069 2.057 2.022 2.018 2.007 1.981 1.922 1.917 1.909 1.903 1.882 1.877 1.799 1.788 1.760 1.757 1.754 1.746 1.743 1.739 1.736 1.733 1.722 1.684 1.679 1.670 1.666 1.662 1.653 1.649 1.645 1.636 1.632 1.514 1.501 1.489 1.486 1.472 1.463 1.458 1.451 1.444 1.436 1.403 1.393 1.389 1.379 1.376 1.366 1.306 1.297 1.272 1.267 1.238 1.234 1.096 1.074 1.070 1.042 0.697
Me
N
Me
Me
H
Me
H
H
H
TIPSO
25cb
1H NMR (800 MHz, CDCl3)
1.00
1.02
1.02
1.03
1.08
1.18
3.00
1.17
1.19
4.74
2.63
20.11
24.56
3.01
f1 (ppm)

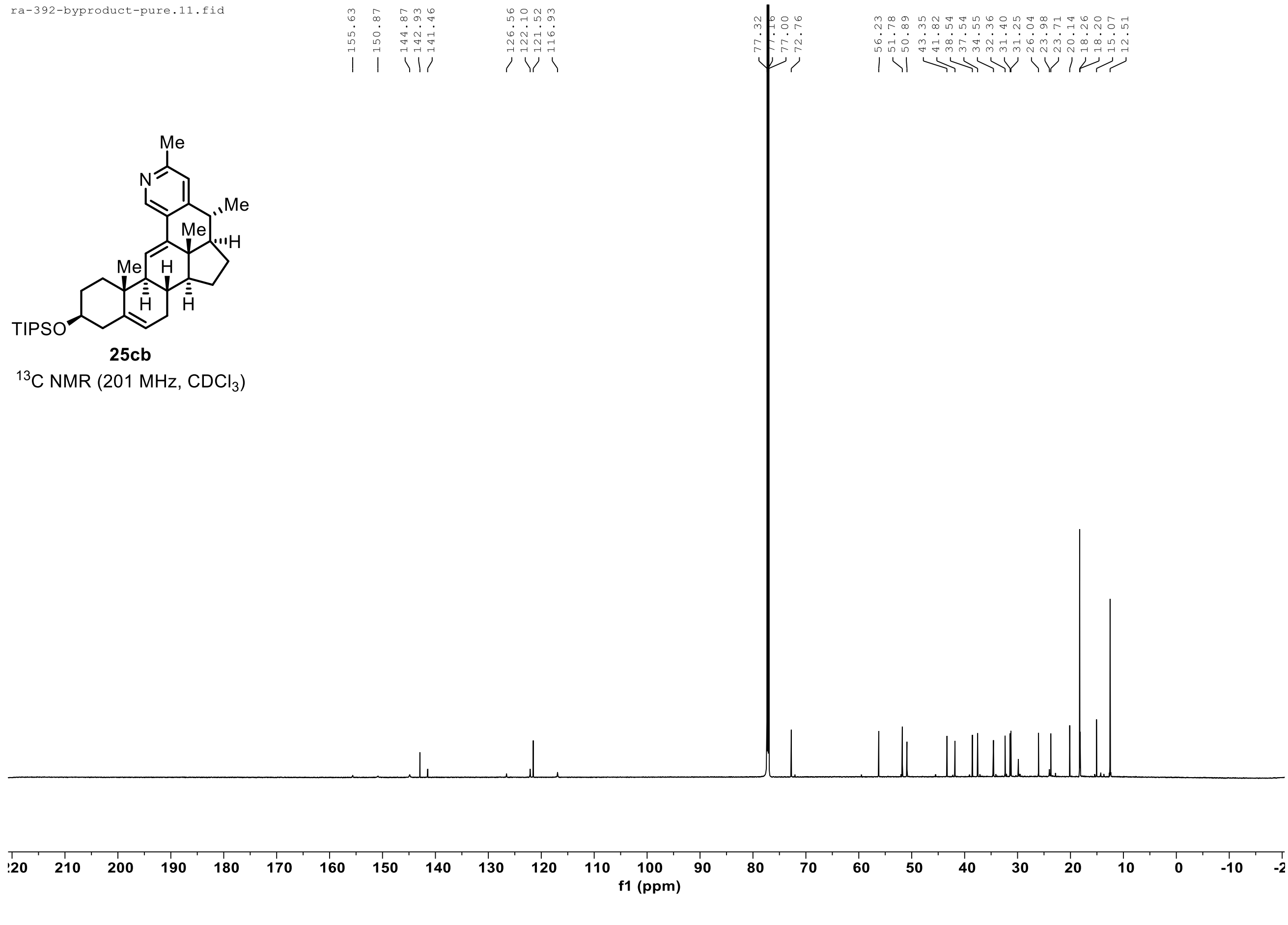
ra-392-byproduct-pure.11.fid
155.63 150.87 144.87 142.93 141.46 126.56 122.10 121.52 116.93 77.32 77.16 77.00 72.76 56.23 51.78 50.89 43.35 41.82 38.54 37.54 34.55 32.36 31.40 31.25 26.04 23.98 23.71 20.14 18.26 18.20 15.07 12.51
Me
N
Me
Me
H
Me
H
H
H
TIPSO
25cb
13C NMR (201 MHz, CDCl3)
f1 (ppm)

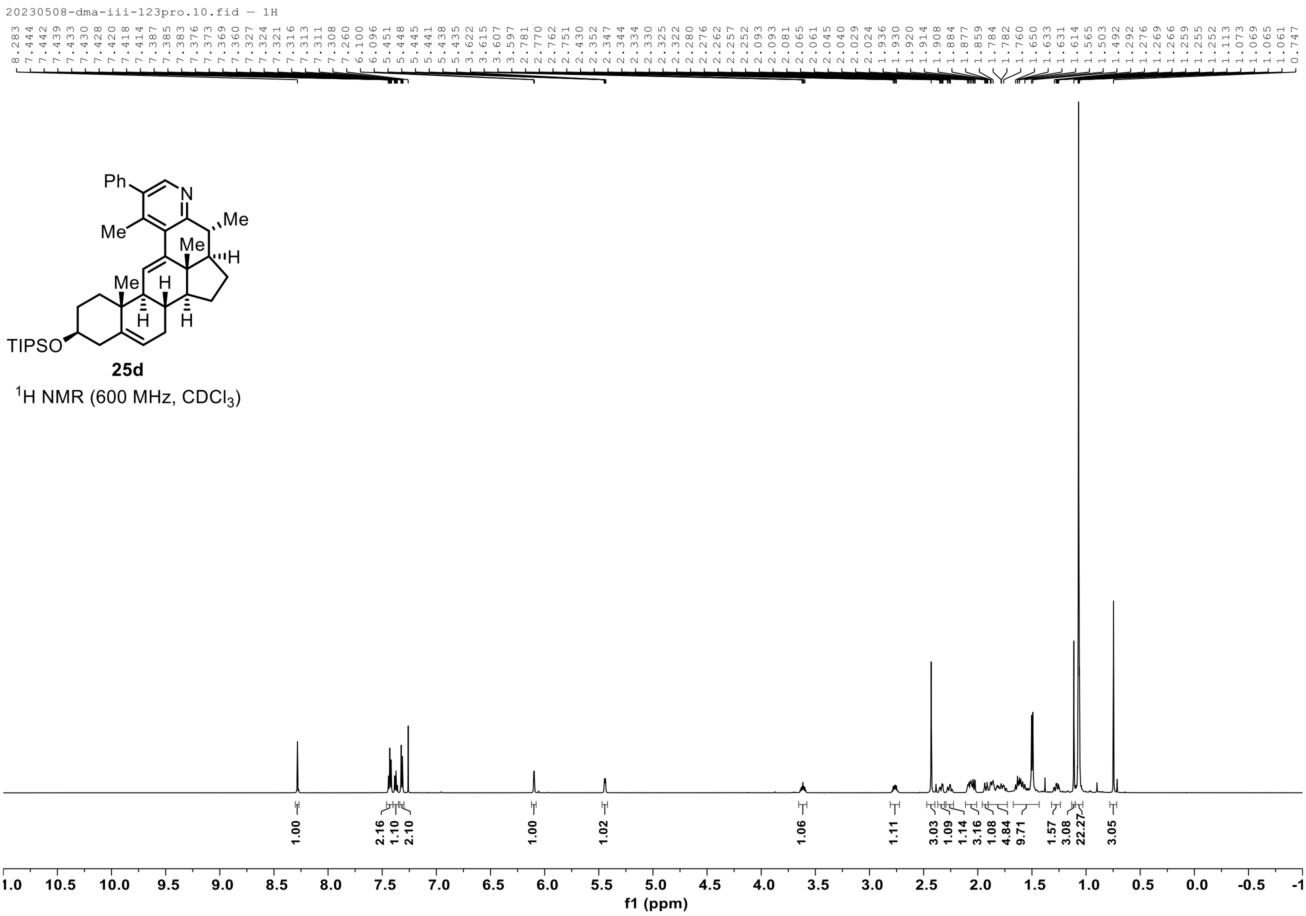
20230508-dma-iii-123pro.10.fid — 1H
8.283 7.444 7.442 7.439 7.433 7.430 7.428 7.420 7.418 7.414 7.387 7.385 7.383 7.376 7.373 7.369 7.360 7.327 7.324 7.321 7.316 7.313 7.311 7.308 7.260 6.100 6.096 5.451 5.448 5.445 5.441 5.438 5.435 3.622 3.615 3.607 3.597 2.781 2.770 2.762 2.751 2.430 2.352 2.347 2.344 2.334 2.330 2.325 2.322 2.280 2.276 2.262 2.257 2.252 2.093 2.093 2.081 2.065 2.061 2.045 2.040 2.029 2.024 1.936 1.930 1.920 1.914 1.908 1.884 1.877 1.859 1.784 1.782 1.760 1.650 1.633 1.631 1.614 1.565 1.503 1.492 1.292 1.276 1.269 1.266 1.259 1.255 1.252 1.113 1.073 1.069 1.065 1.061 0.747
Ph
N
Me
Me
Me
H
Me
H
H
H
TIPSO
25d
^{1}H NMR (600 MHz, CDCl$_3$)
1.00 2.16 1.10 2.10 1.00 1.02 1.06 1.11 3.03 1.09 1.14 3.16 1.08 4.84 9.71 1.57 3.08 22.27 3.05
1.0 10.5 10.0 9.5 9.0 8.5 8.0 7.5 7.0 6.5 6.0 5.5 5.0 4.5 4.0 3.5 3.0 2.5 2.0 1.5 1.0 0.5 0.0 -0.5 -1
f1 (ppm)

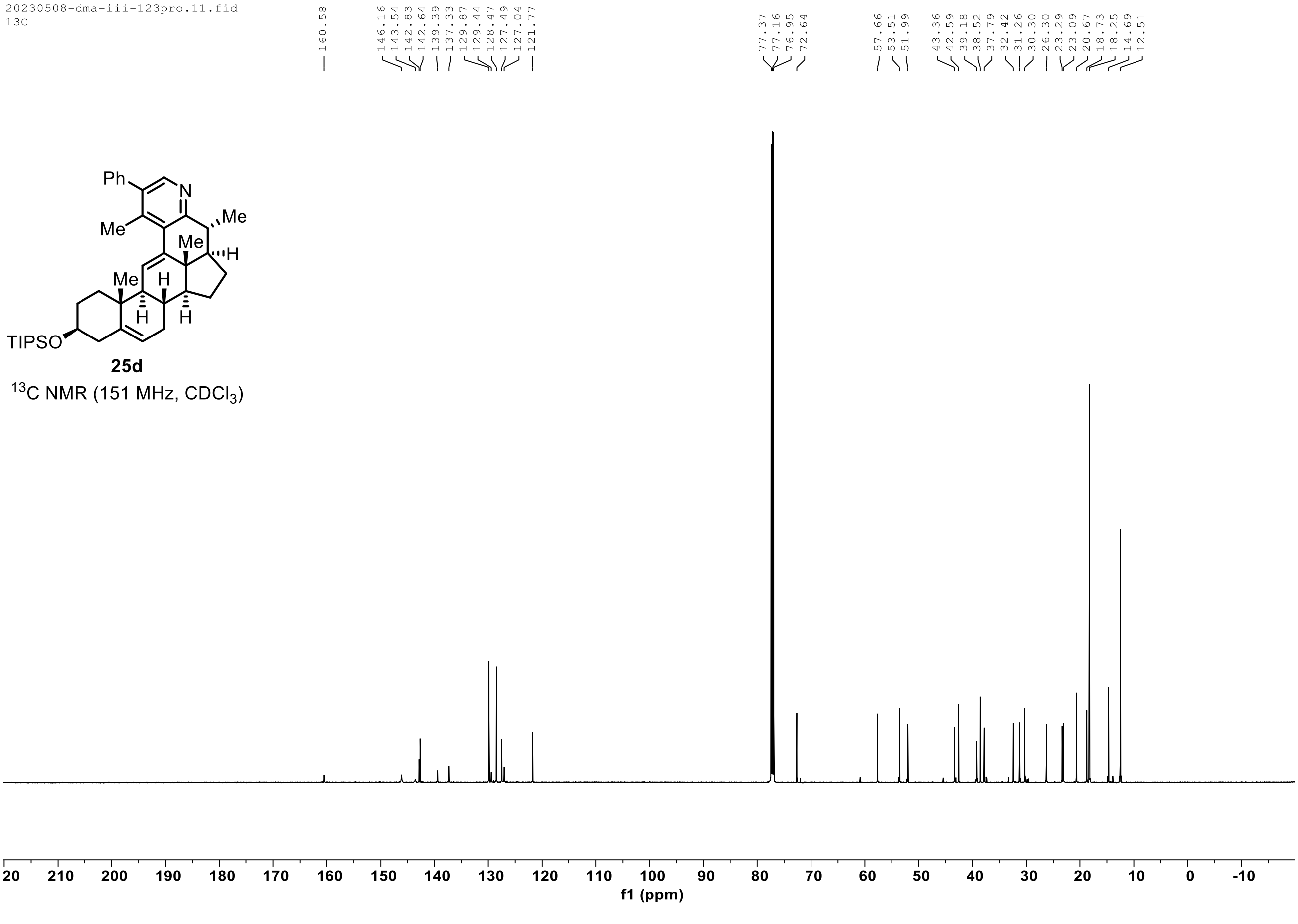
20230508-dma-iii-123pro.11.fid
13C
160.58 146.16 143.54 142.83 142.64 139.39 137.33 129.87 129.44 128.47 127.49 127.04 121.77 77.37 77.16 76.95 72.64 57.66 53.51 51.99 43.36 42.59 39.18 38.52 37.79 32.42 31.26 30.30 26.30 23.29 23.09 20.67 18.73 18.25 14.69 12.51
Ph
N
Me
Me
Me
H
Me
H
H
H
TIPSO
25d
^{13}C NMR (151 MHz, CDCl$_3$)
20 210 200 190 180 170 160 150 140 130 120 110 100 90 80 70 60 50 40 30 20 10 0 -10
f1 (ppm)

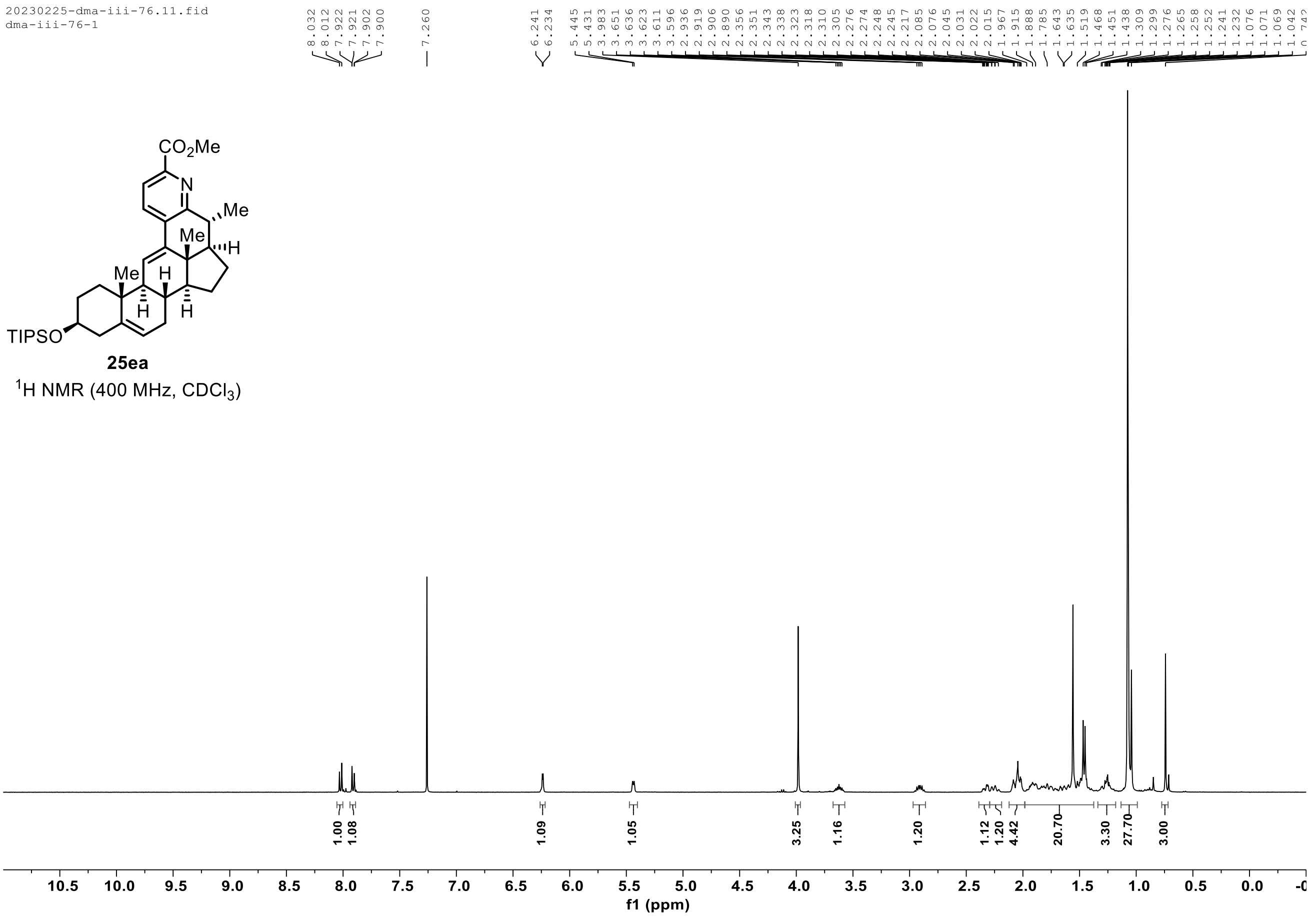

25ea

^{1}H NMR (400 MHz, $CDCl_3$)

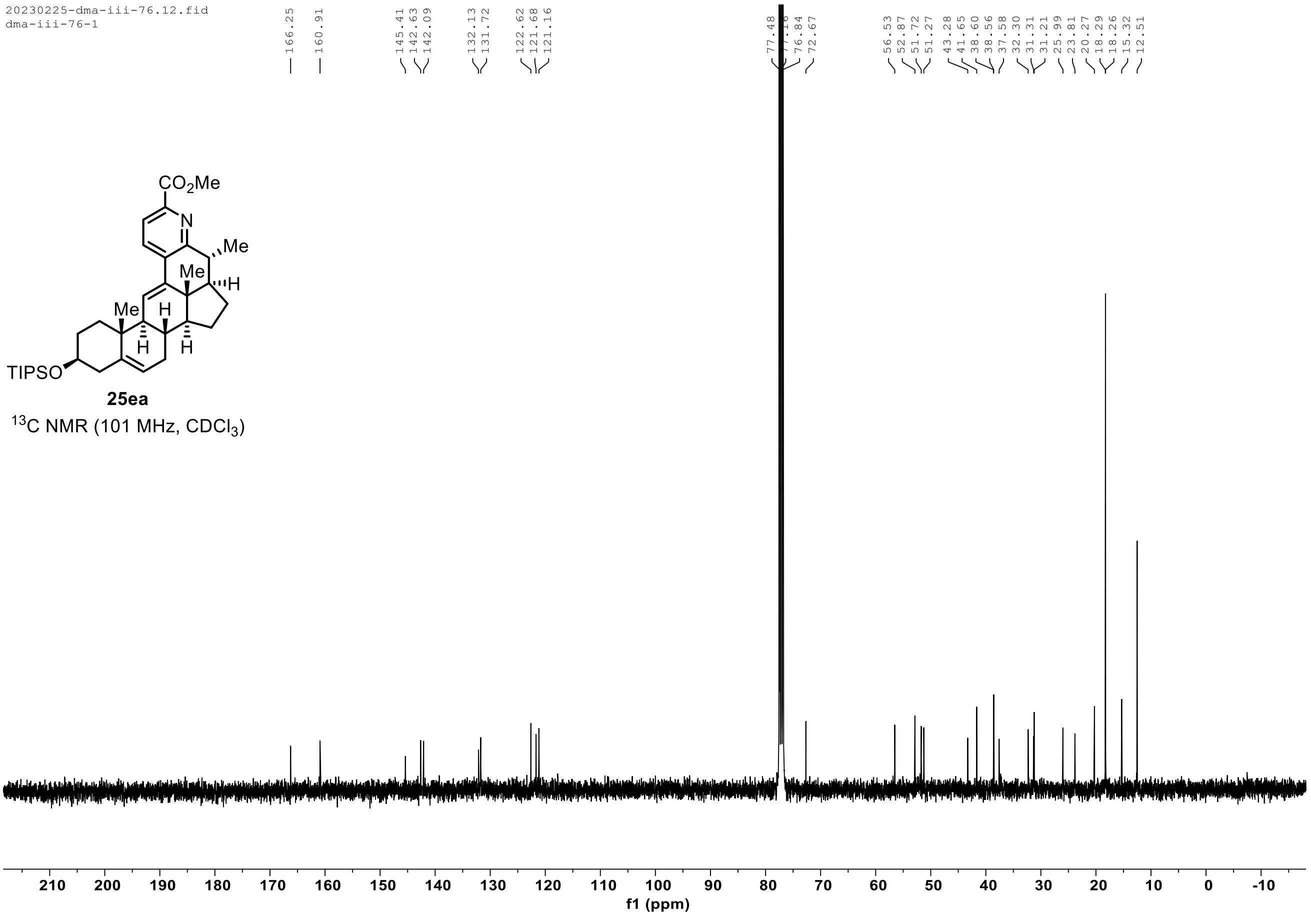

25ea

^{13}C NMR (101 MHz, $CDCl_3$)

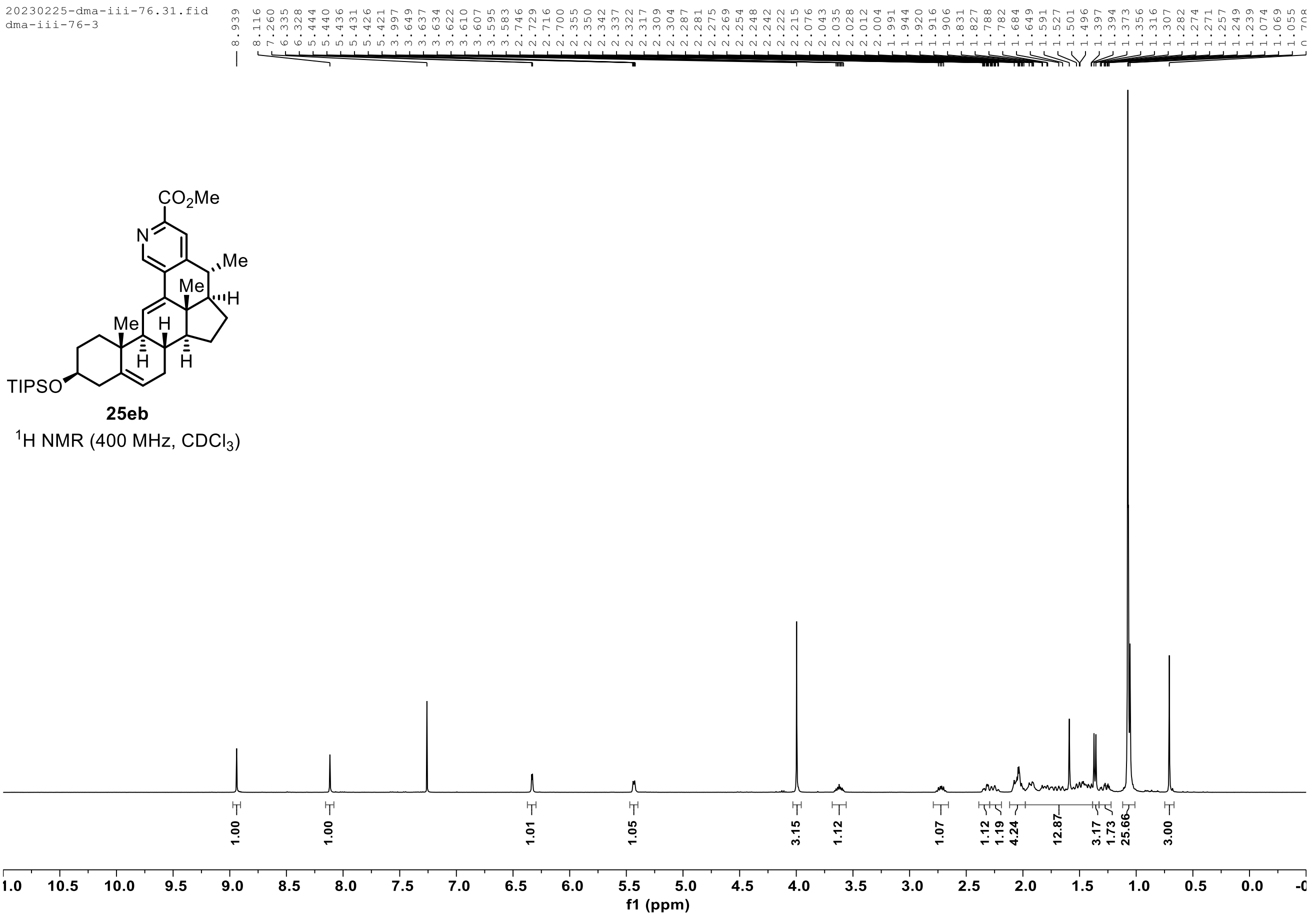

25eb

^{1}H NMR (400 MHz, $CDCl_3$)

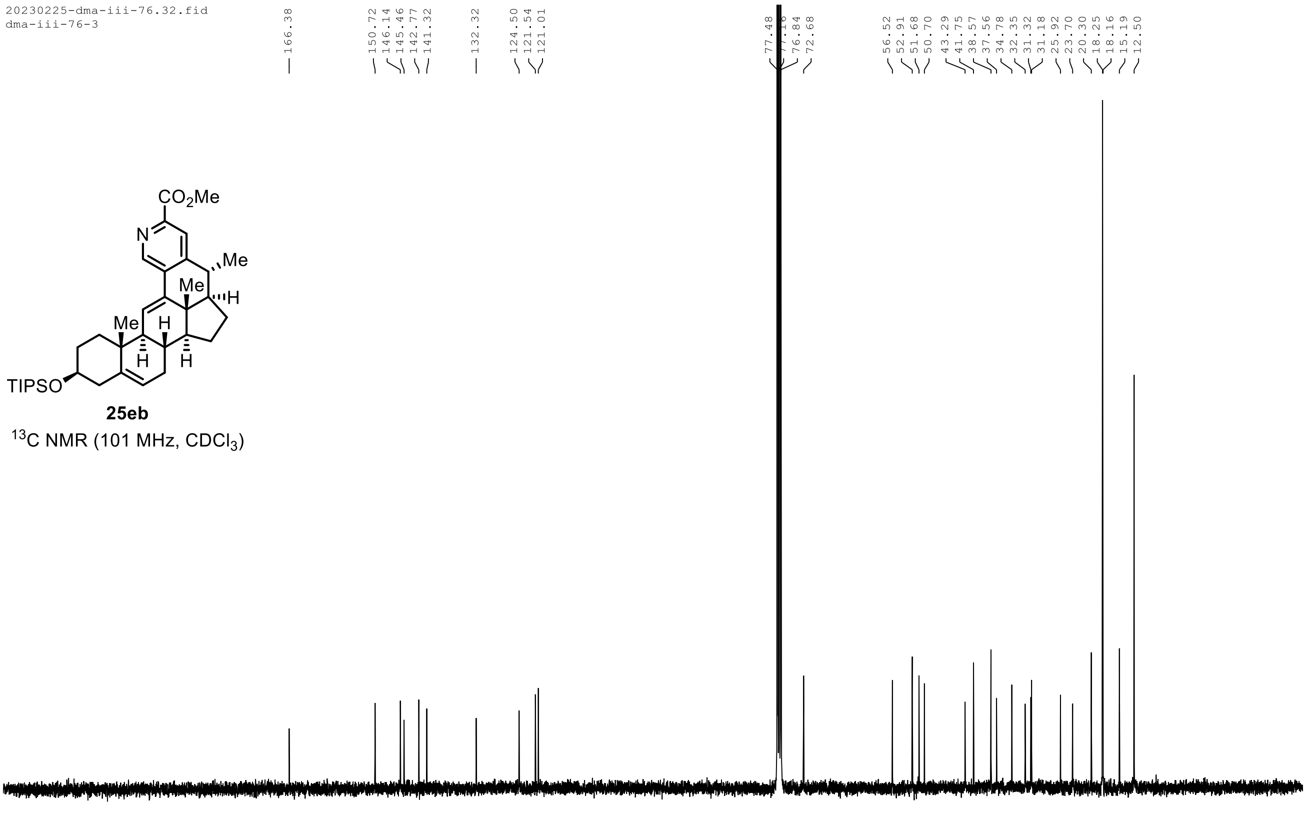

25eb

^{13}C NMR (101 MHz, $CDCl_3$)

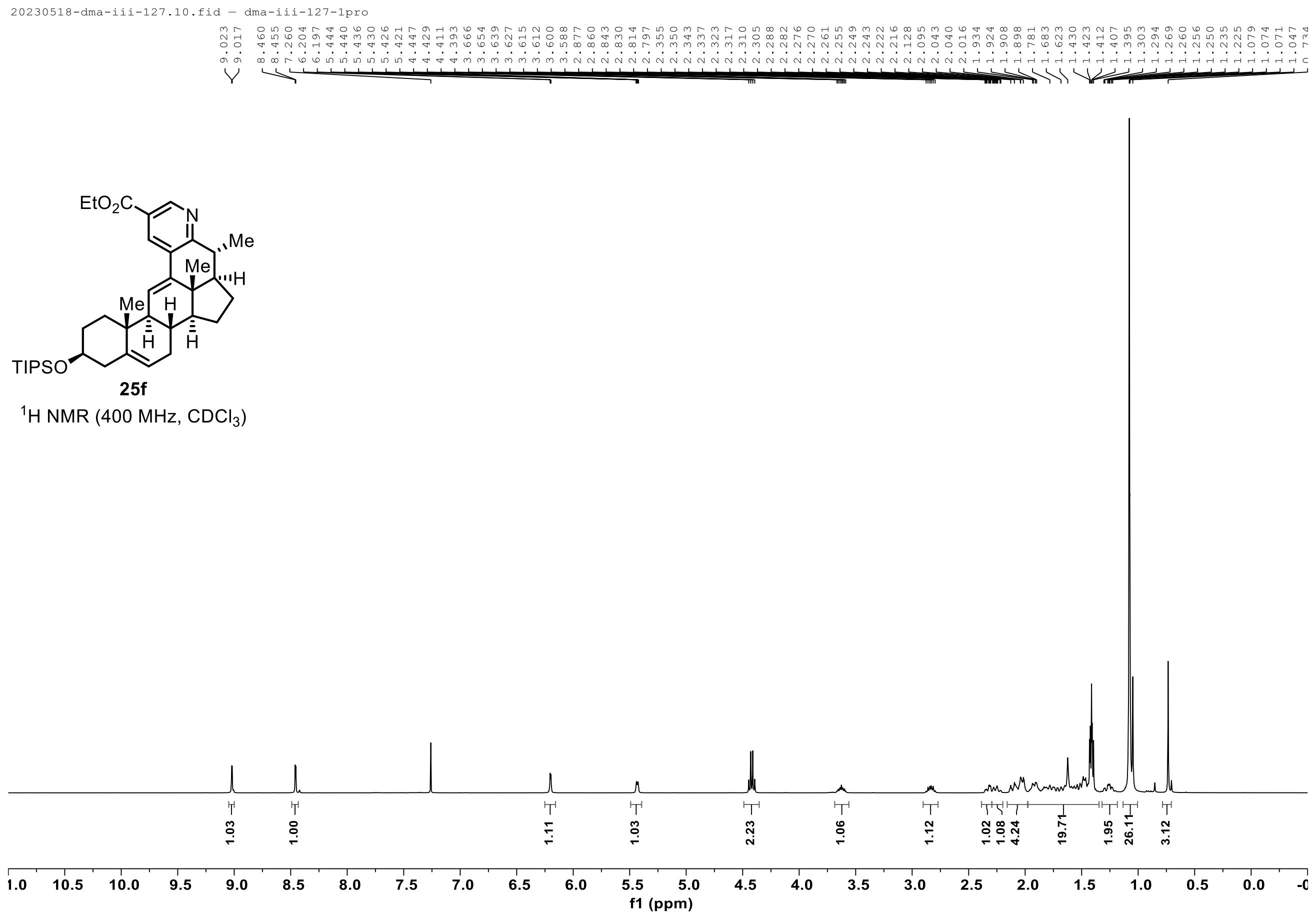

25f

^{1}H NMR (400 MHz, $CDCl_3$)

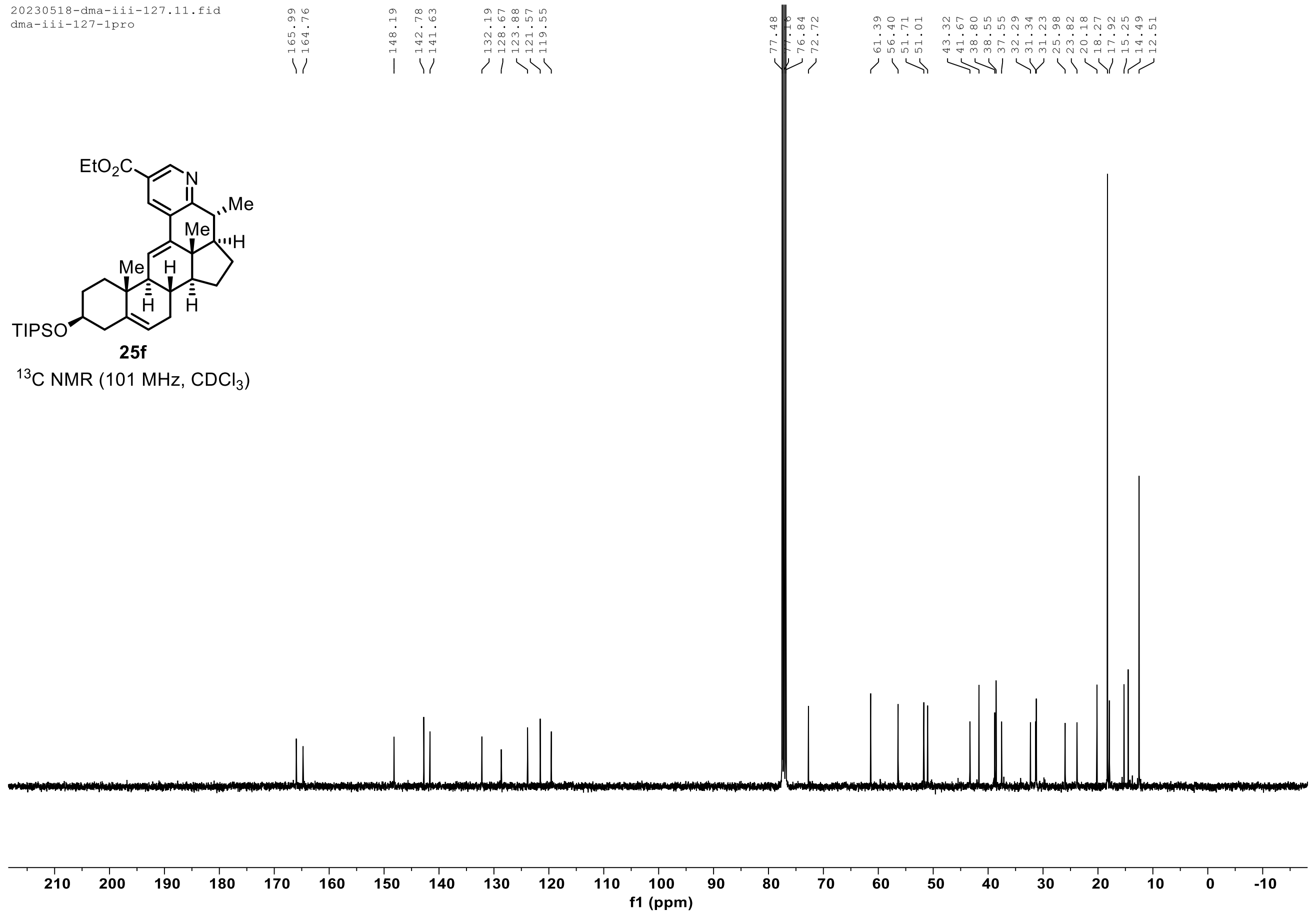

25f

^{13}C NMR (101 MHz, $CDCl_3$)

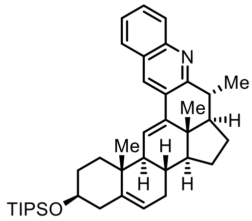

25ga

^{1}H NMR (400 MHz, $CDCl_3$)

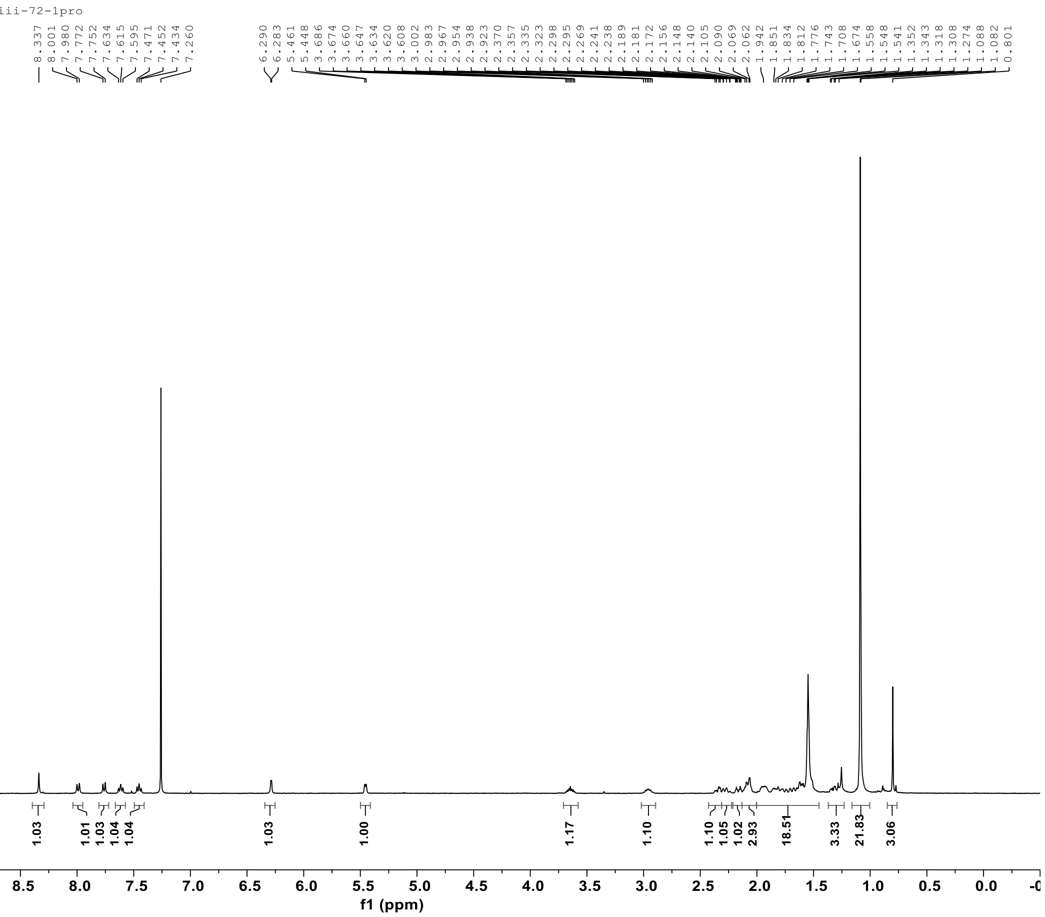

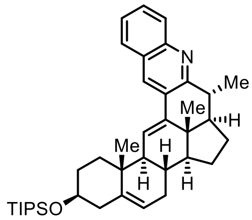

25ga

^{13}C NMR (151 MHz, $CDCl_3$)

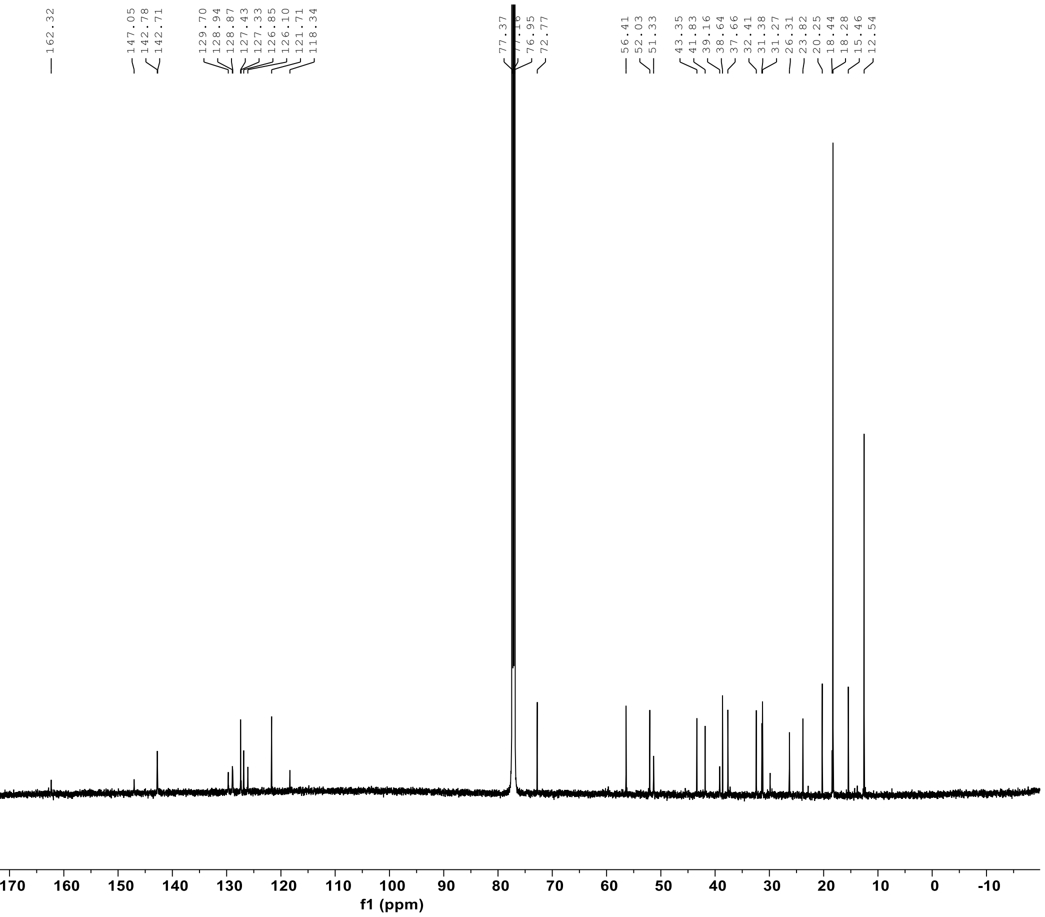

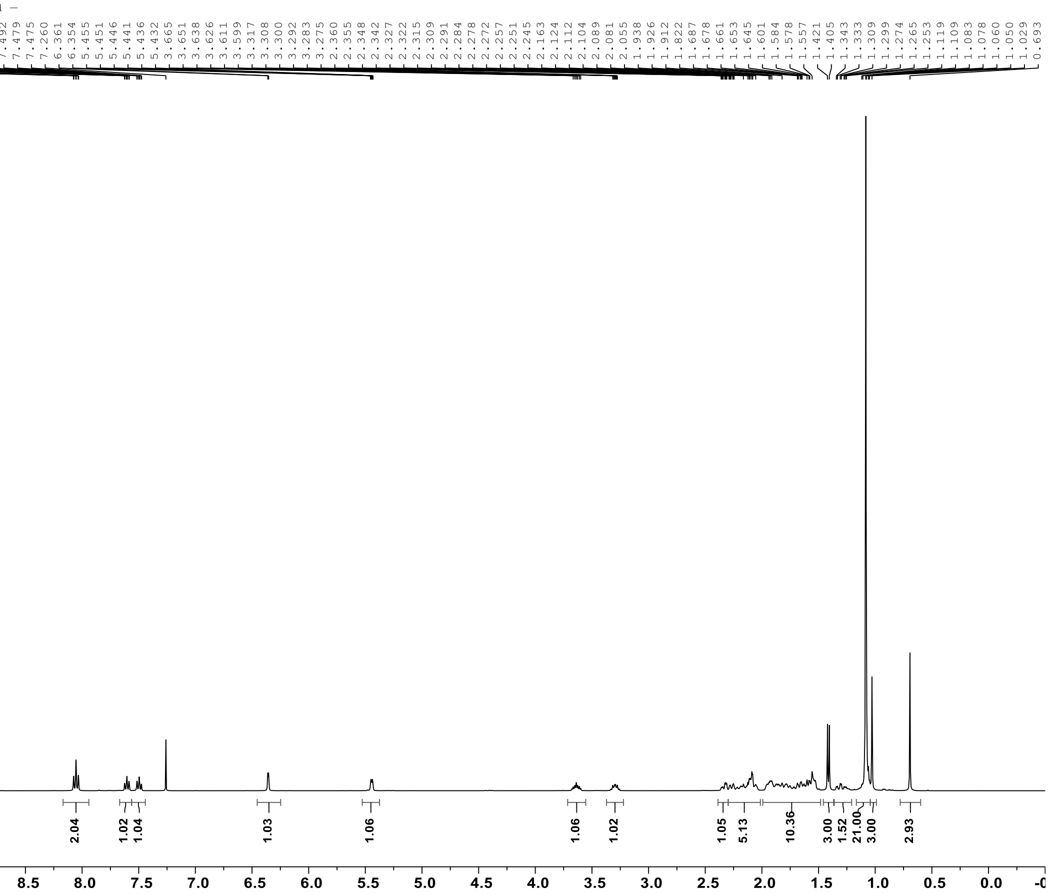

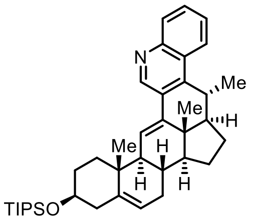

25gb

^{1}H NMR (400 MHz, $CDCl_3$)

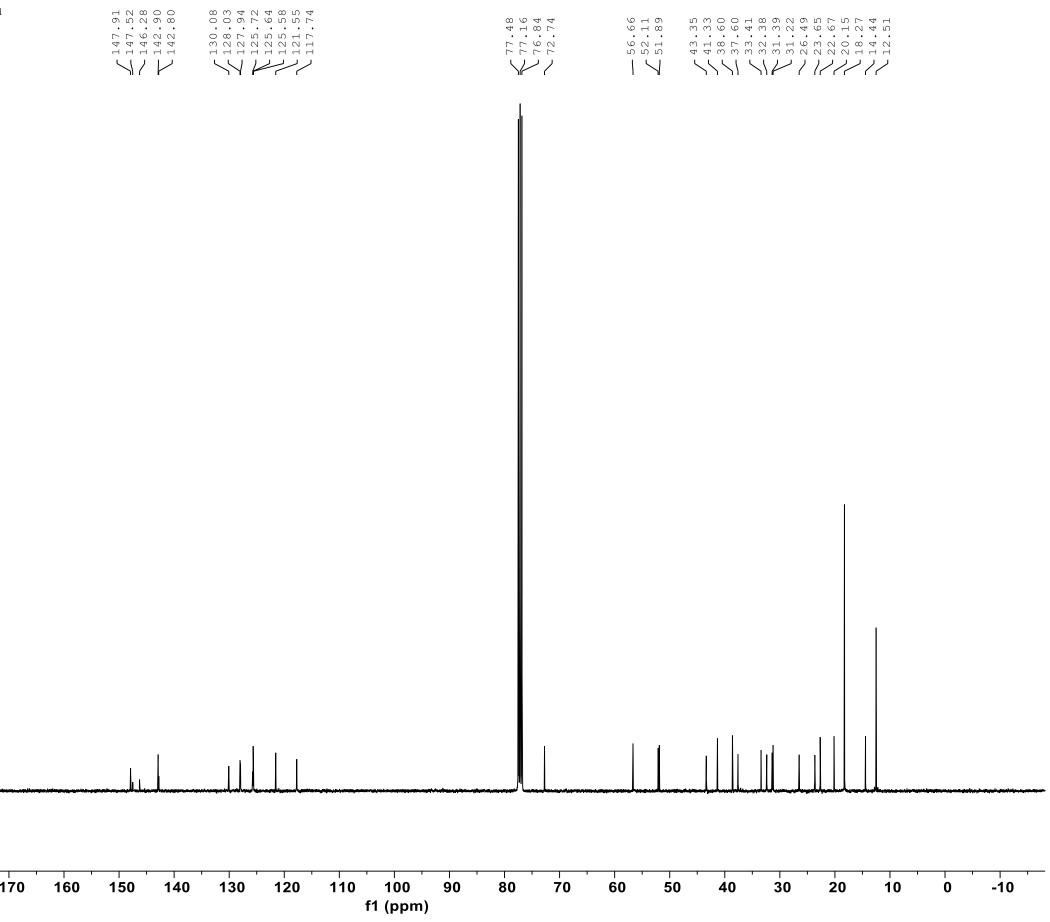

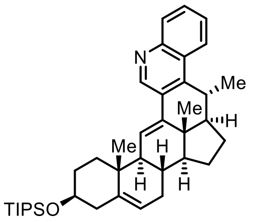

25gb

^{13}C NMR (101 MHz, $CDCl_3$)

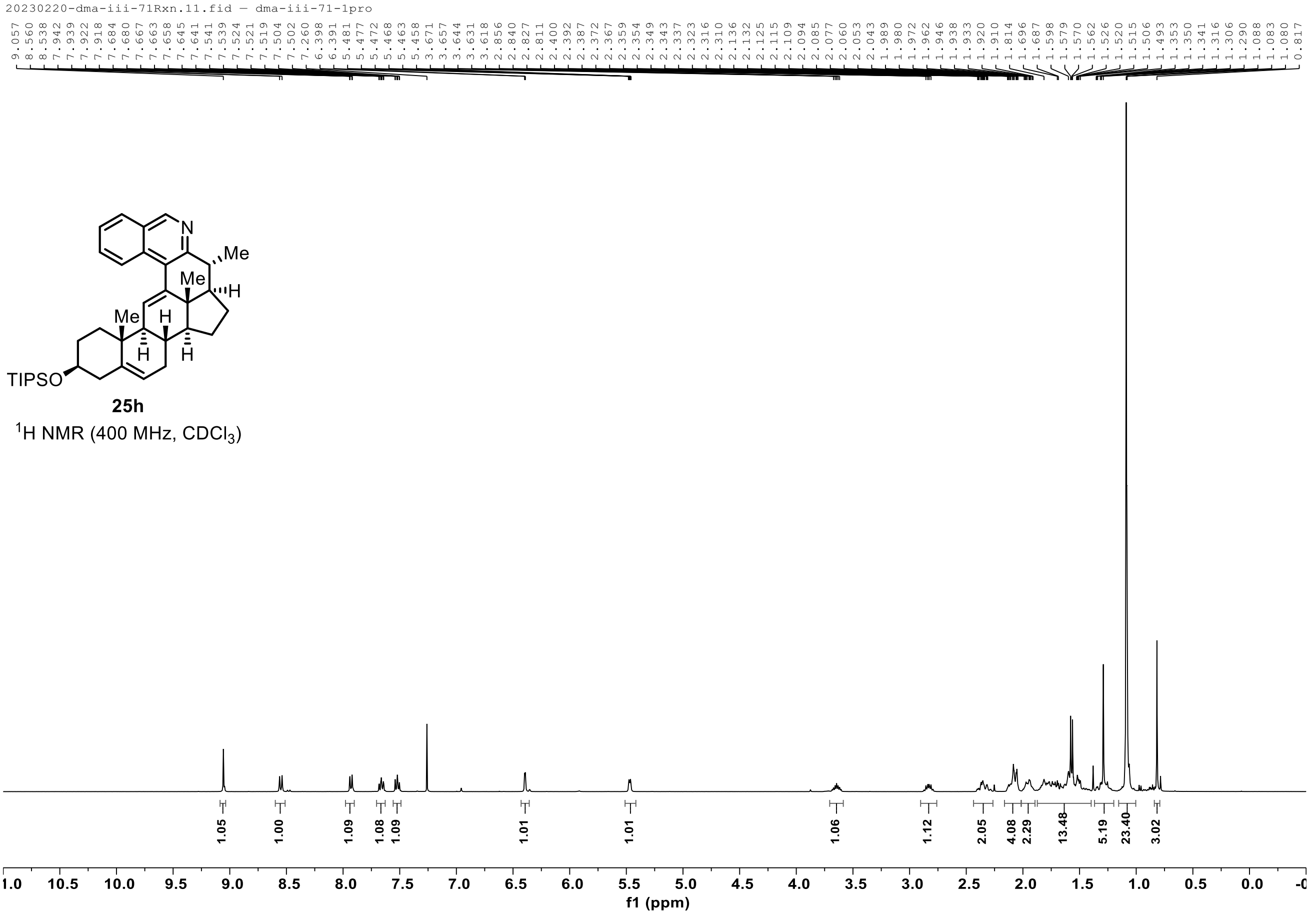

20230220-dma-iii-71Rxn.11.fid — dma-iii-71-1pro
9.057 8.560 8.538 7.942 7.939 7.922 7.918 7.684 7.680 7.667 7.663 7.658 7.645 7.641 7.541 7.539 7.524 7.521 7.519 7.504 7.502 7.260 6.398 6.391 5.481 5.477 5.472 5.468 5.463 5.458 3.671 3.657 3.644 3.631 3.618 2.856 2.840 2.827 2.811 2.400 2.392 2.387 2.372 2.367 2.359 2.354 2.349 2.343 2.337 2.323 2.316 2.310 2.136 2.132 2.125 2.115 2.109 2.094 2.085 2.077 2.060 2.053 2.043 1.989 1.980 1.972 1.962 1.946 1.938 1.933 1.920 1.910 1.814 1.696 1.687 1.598 1.579 1.570 1.562 1.526 1.520 1.515 1.506 1.493 1.353 1.350 1.341 1.316 1.306 1.290 1.088 1.083 1.080 0.817
Me
N
Me
H
Me
H
H
H
TIPSO
25h
¹H NMR (400 MHz, CDCl₃)
1.05 1.00 1.09 1.08 1.09 1.01 1.01 1.06 1.12 2.05 4.08 2.29 13.48 5.19 23.40 3.02
1.0 10.5 10.0 9.5 9.0 8.5 8.0 7.5 7.0 6.5 6.0 5.5 5.0 4.5 4.0 3.5 3.0 2.5 2.0 1.5 1.0 0.5 0.0 -0
f1 (ppm)

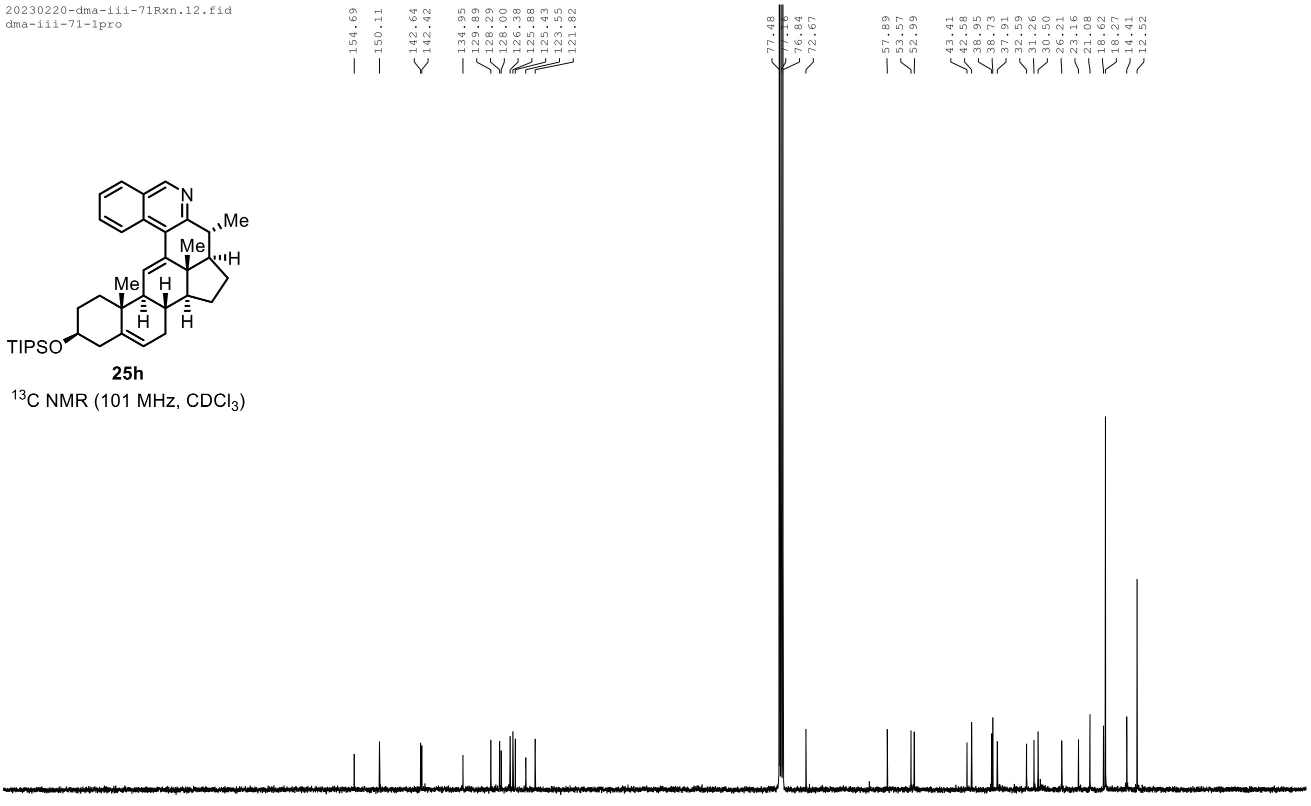

20230220-dma-iii-71Rxn.12.fid
dma-iii-71-1pro
154.69 150.11 142.64 142.42 134.95 129.89 128.29 128.00 126.38 125.88 125.43 123.55 121.82 77.48 77.16 76.84 72.67 57.89 53.57 52.99 43.41 42.58 38.95 38.73 37.91 32.59 31.26 30.50 26.21 23.16 21.08 18.62 18.27 14.41 12.52
Me
N
Me
H
Me
H
H
H
TIPSO
25h
¹³C NMR (101 MHz, CDCl₃)
210 200 190 180 170 160 150 140 130 120 110 100 90 80 70 60 50 40 30 20 10 0 -10
f1 (ppm)

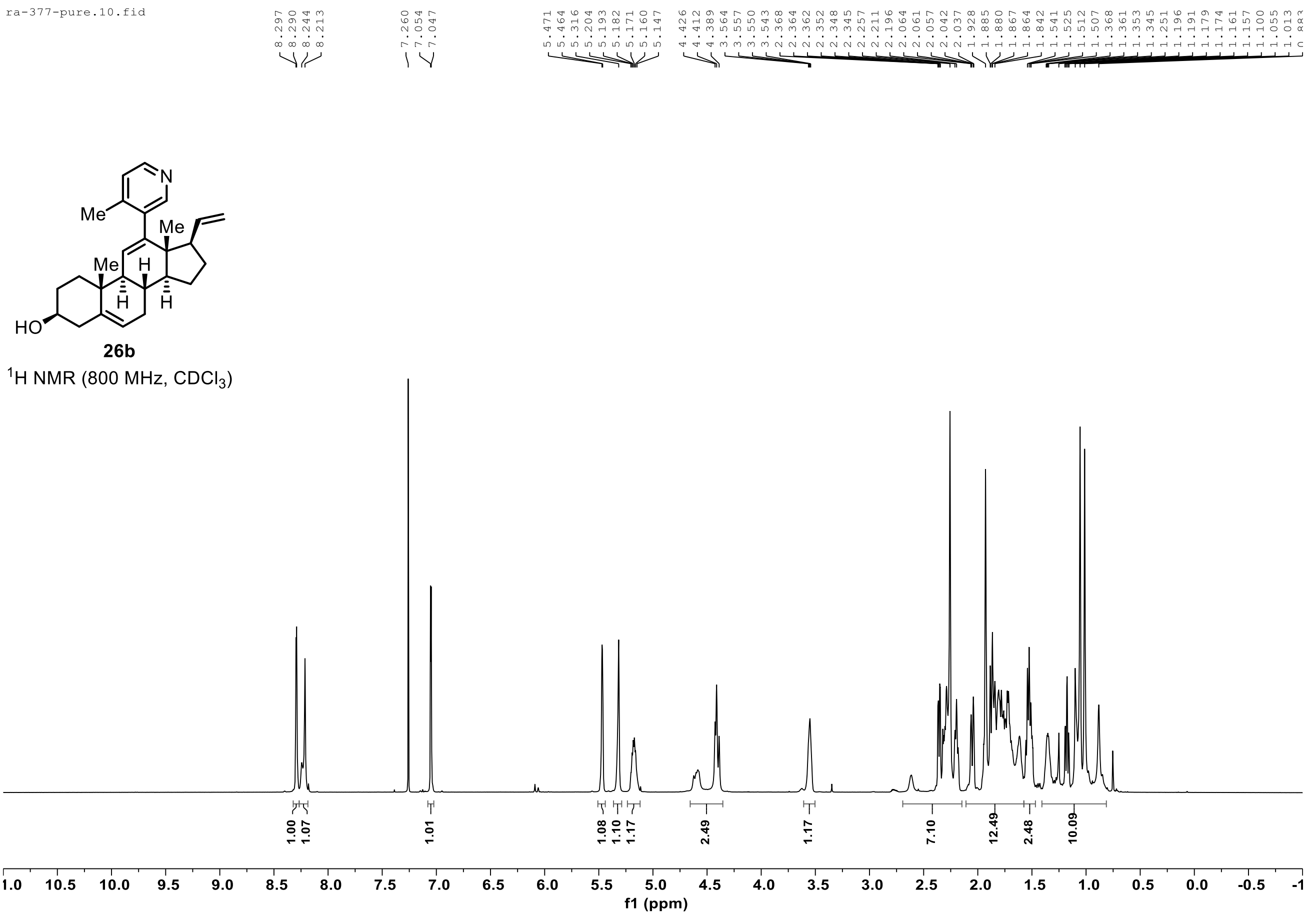

26b

^{1}H NMR (800 MHz, $CDCl_3$)

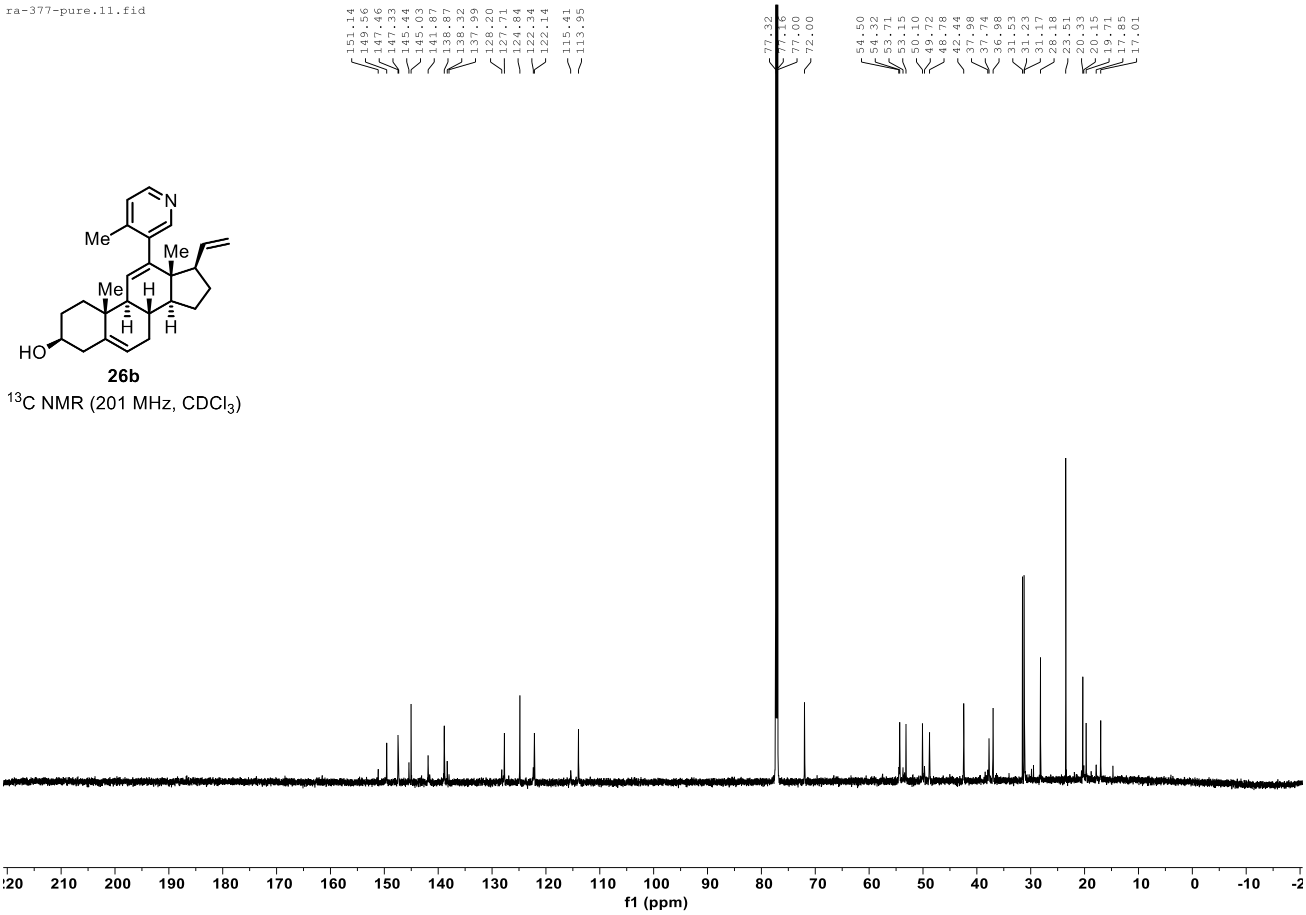

26b

^{13}C NMR (201 MHz, $CDCl_3$)

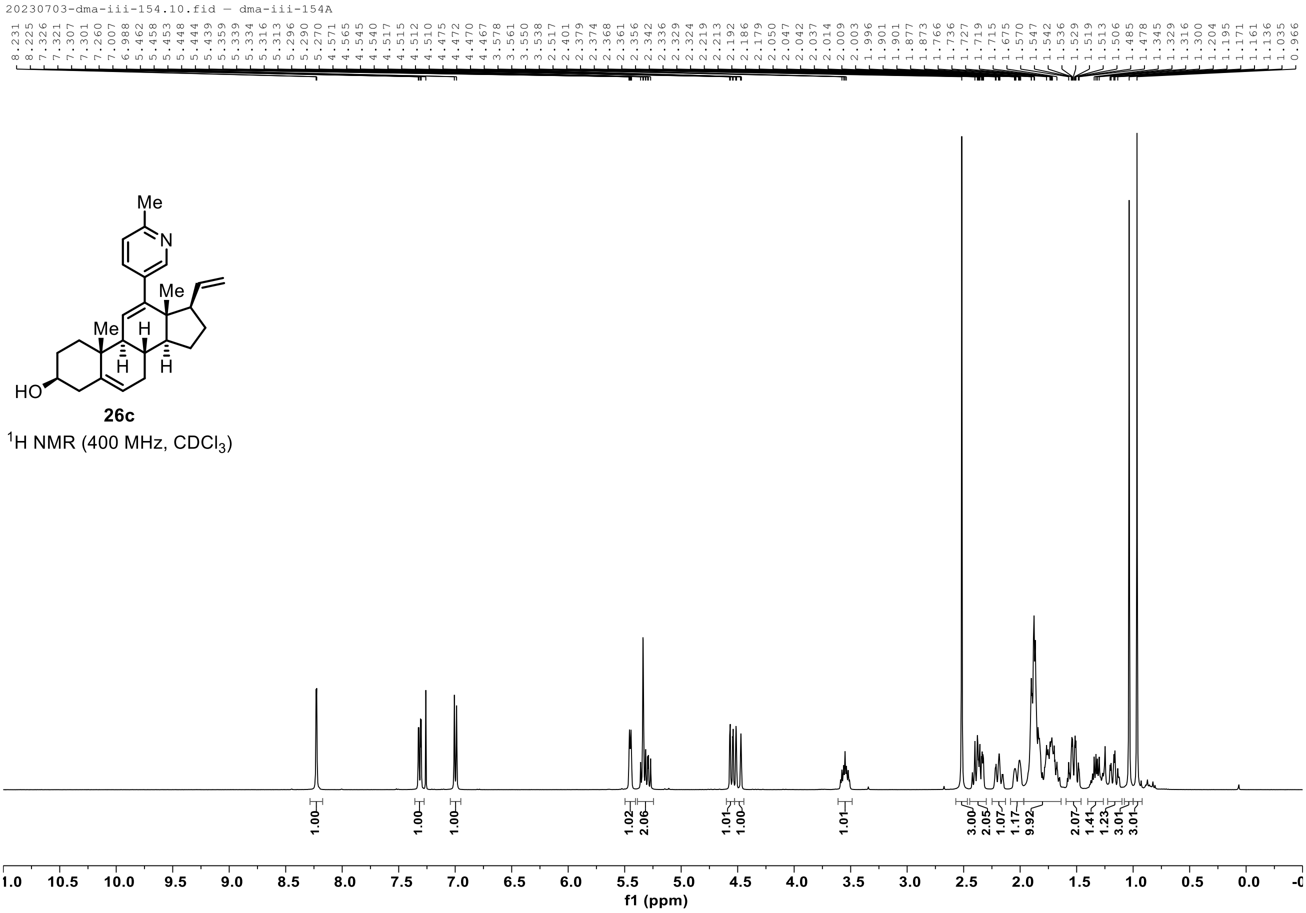

26c

^{1}H NMR (400 MHz, $CDCl_3$)

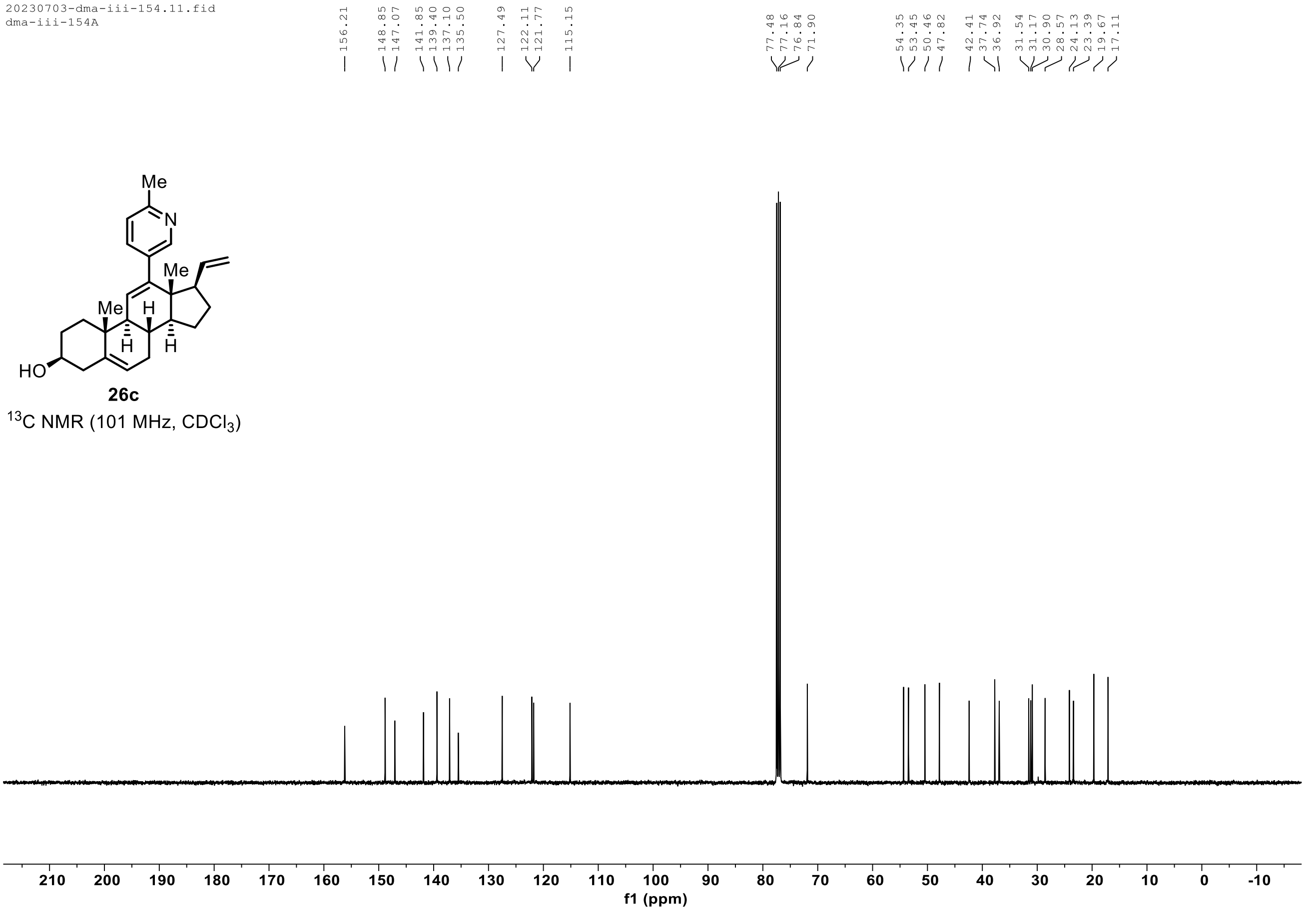

26c

^{13}C NMR (101 MHz, $CDCl_3$)

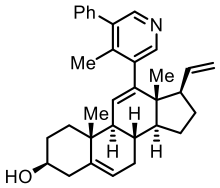

26d

^{1}H NMR (800 MHz, $CDCl_3$)

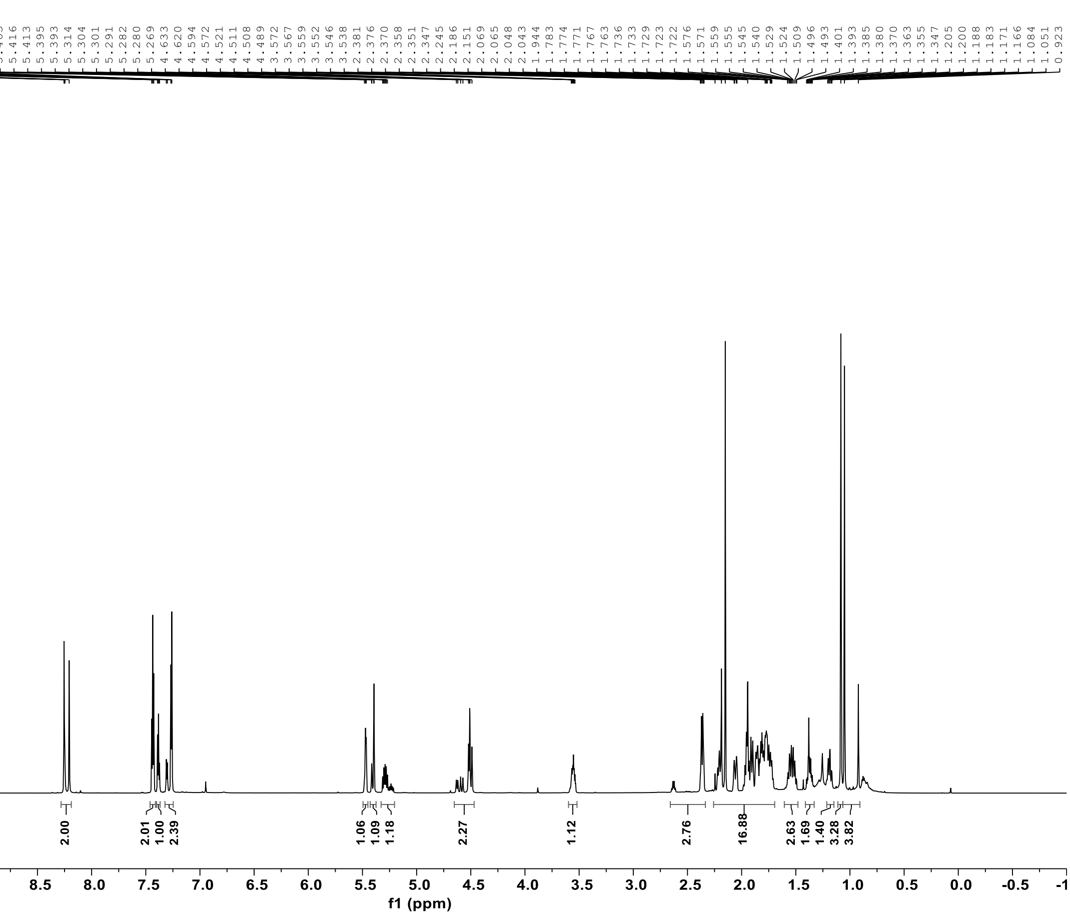

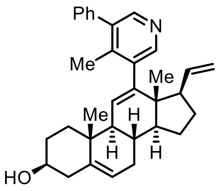

26d

^{13}C NMR (201 MHz, $CDCl_3$)

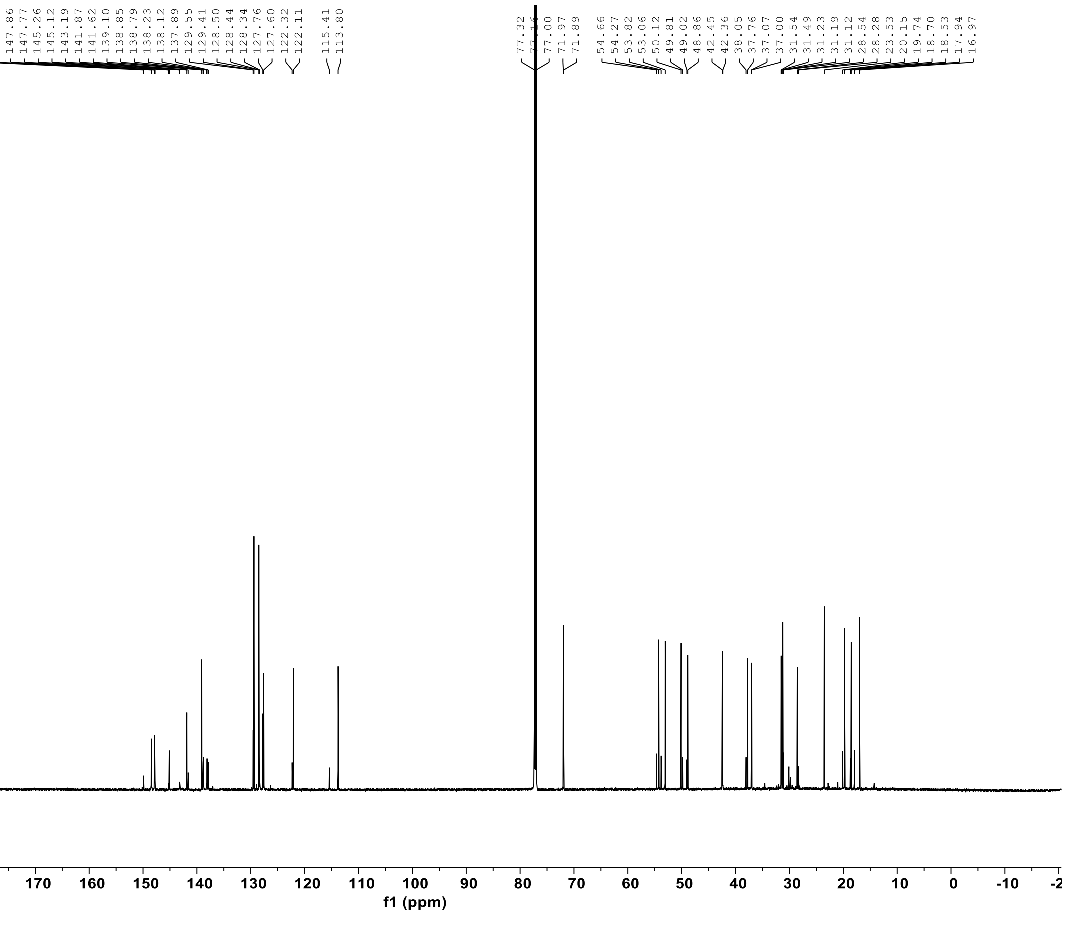

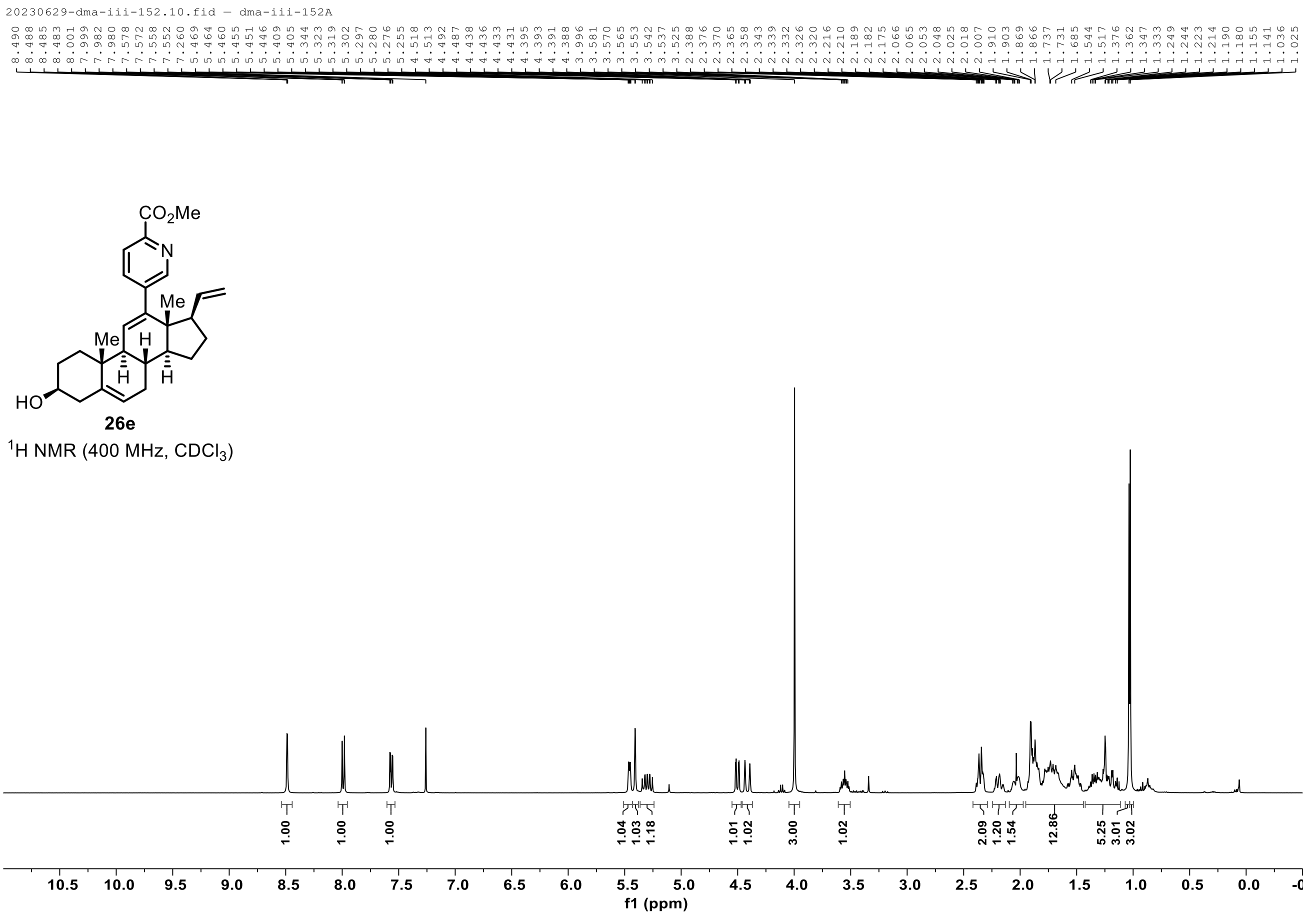

26e

^{1}H NMR (400 MHz, $CDCl_3$)

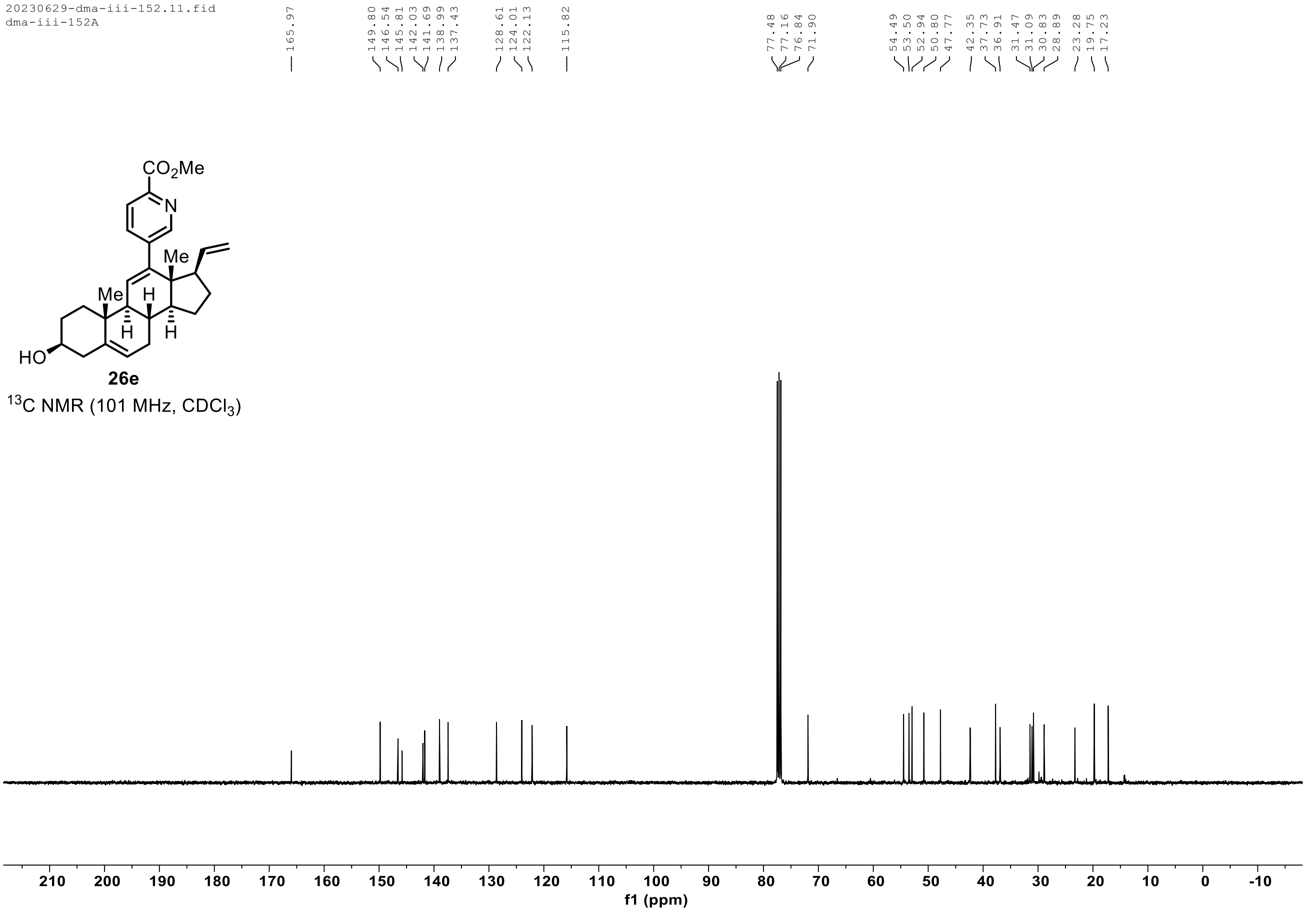

26e

^{13}C NMR (101 MHz, $CDCl_3$)

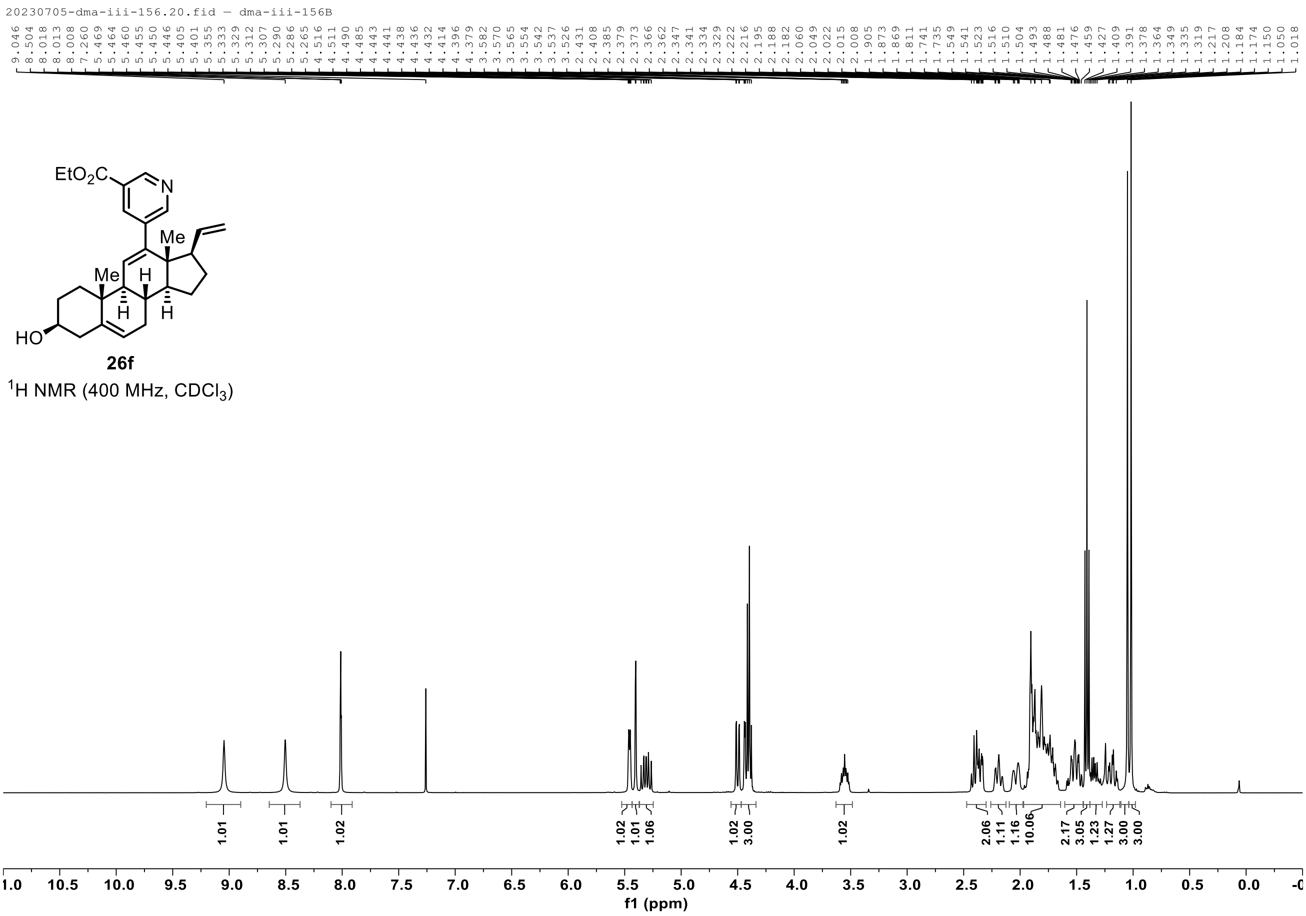
20230705-dma-iii-156.20.fid — dma-iii-156B
9.046 8.504 8.018 8.013 8.008 7.260 5.469 5.464 5.460 5.455 5.450 5.446 5.405 5.401 5.355 5.333 5.329 5.312 5.307 5.290 5.286 5.265 4.516 4.511 4.490 4.485 4.443 4.441 4.438 4.436 4.432 4.414 4.396 4.379 3.582 3.570 3.565 3.554 3.542 3.537 3.526 2.431 2.408 2.385 2.379 2.373 2.366 2.362 2.347 2.341 2.334 2.329 2.222 2.216 2.195 2.188 2.182 2.060 2.049 2.022 2.015 2.008 1.905 1.873 1.869 1.811 1.741 1.735 1.549 1.541 1.523 1.516 1.510 1.504 1.493 1.488 1.481 1.476 1.459 1.427 1.409 1.391 1.378 1.364 1.349 1.335 1.319 1.217 1.208 1.184 1.174 1.150 1.018
EtO2C
N
Me
Me
H
H
H
HO
26f
1H NMR (400 MHz, CDCl3)
1.01 1.01 1.02 1.02 1.01 1.06 1.02 3.00 1.02 2.06 1.11 1.16 10.06 2.17 3.05 1.23 1.27 3.00 3.00
1.0 10.5 10.0 9.5 9.0 8.5 8.0 7.5 7.0 6.5 6.0 5.5 5.0 4.5 4.0 3.5 3.0 2.5 2.0 1.5 1.0 0.5 0.0
f1 (ppm)

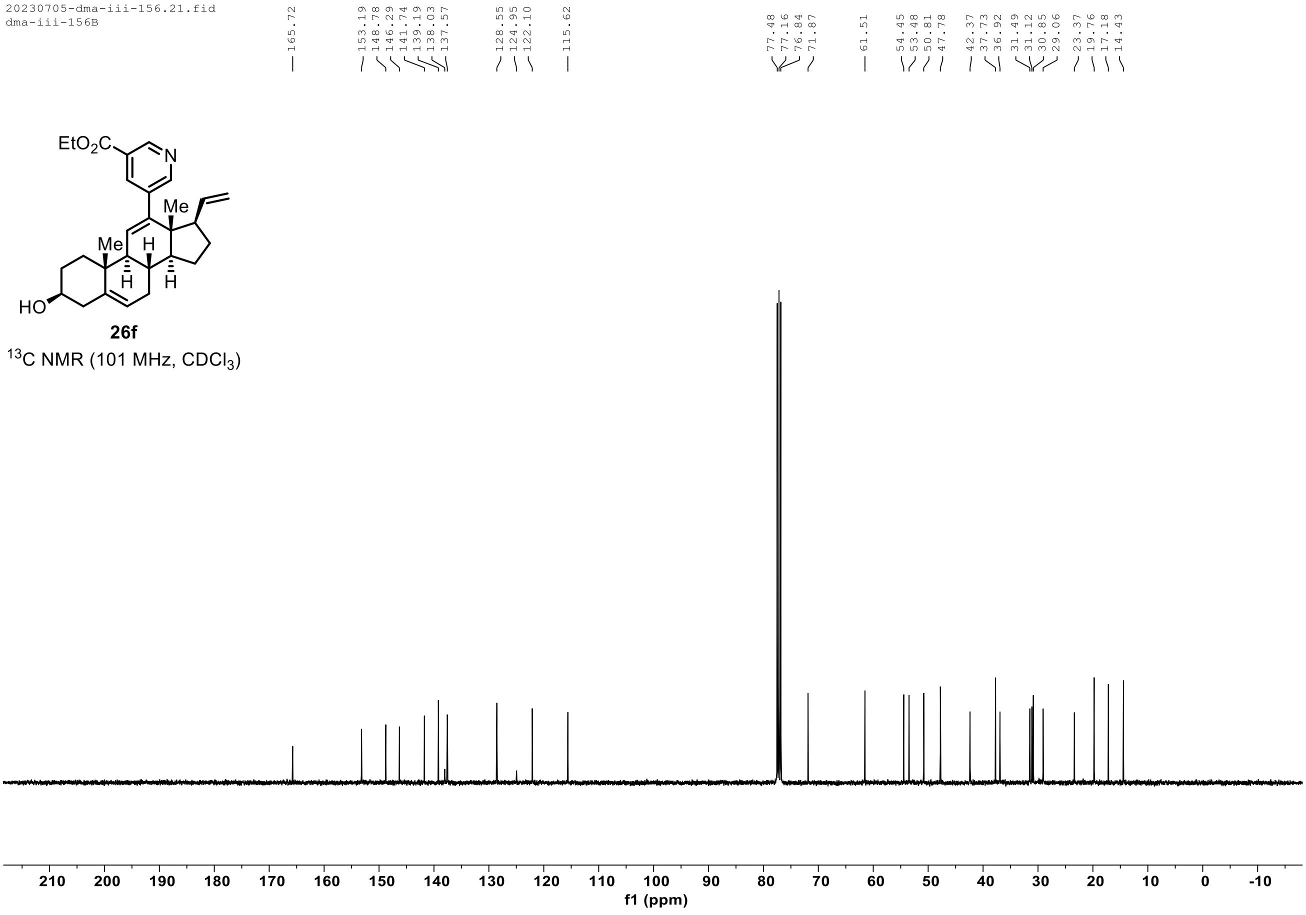
20230705-dma-iii-156.21.fid
dma-iii-156B
165.72 153.19 148.78 146.29 141.74 139.19 138.03 137.57 128.55 124.95 122.10 115.62 77.48 77.16 76.84 71.87 61.51 54.45 53.48 50.81 47.78 42.37 37.73 36.92 31.49 31.12 30.85 29.06 23.37 19.76 17.18 14.43
EtO2C
N
Me
Me
H
H
H
HO
26f
13C NMR (101 MHz, CDCl3)
210 200 190 180 170 160 150 140 130 120 110 100 90 80 70 60 50 40 30 20 10 0 -10
f1 (ppm)

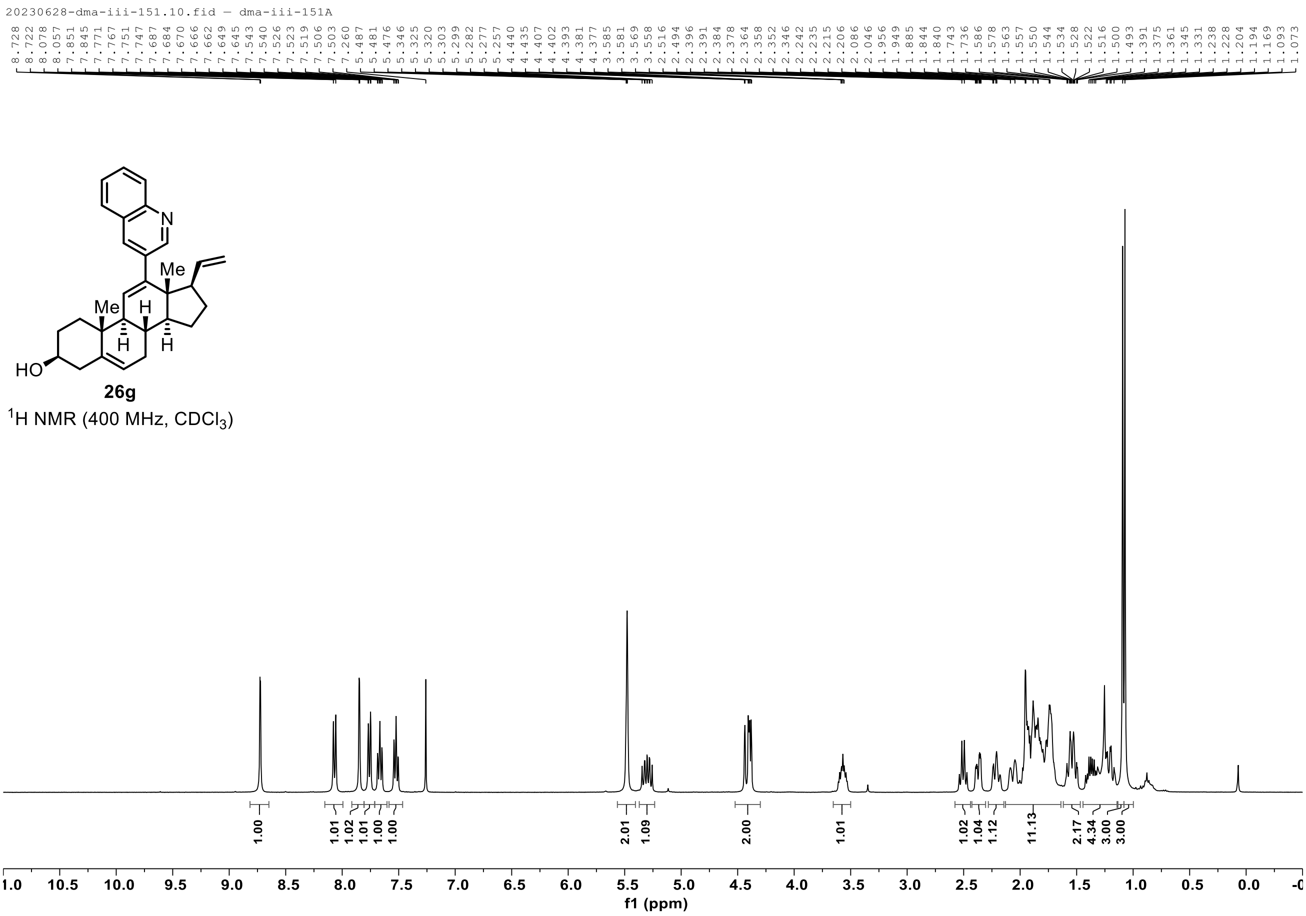

26g

^{1}H NMR (400 MHz, $CDCl_3$)

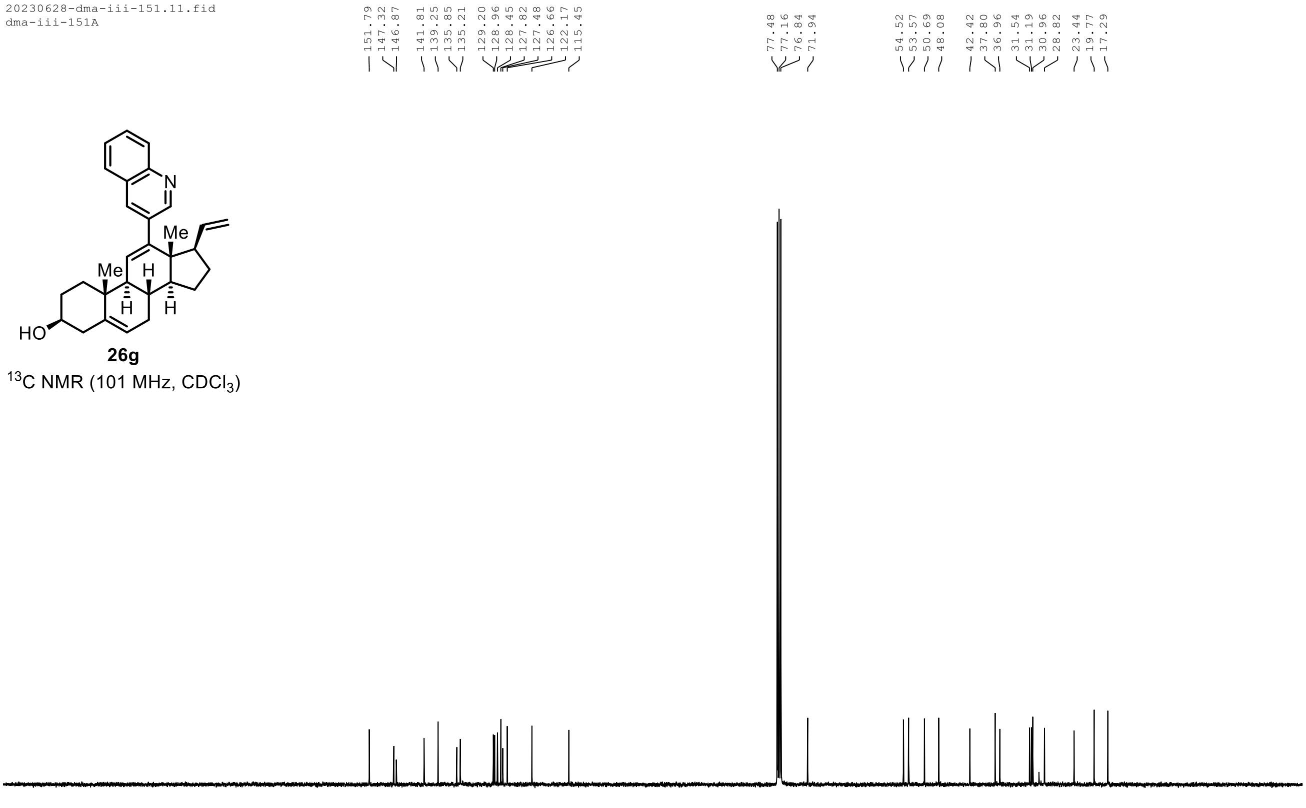

26g

^{13}C NMR (101 MHz, $CDCl_3$)

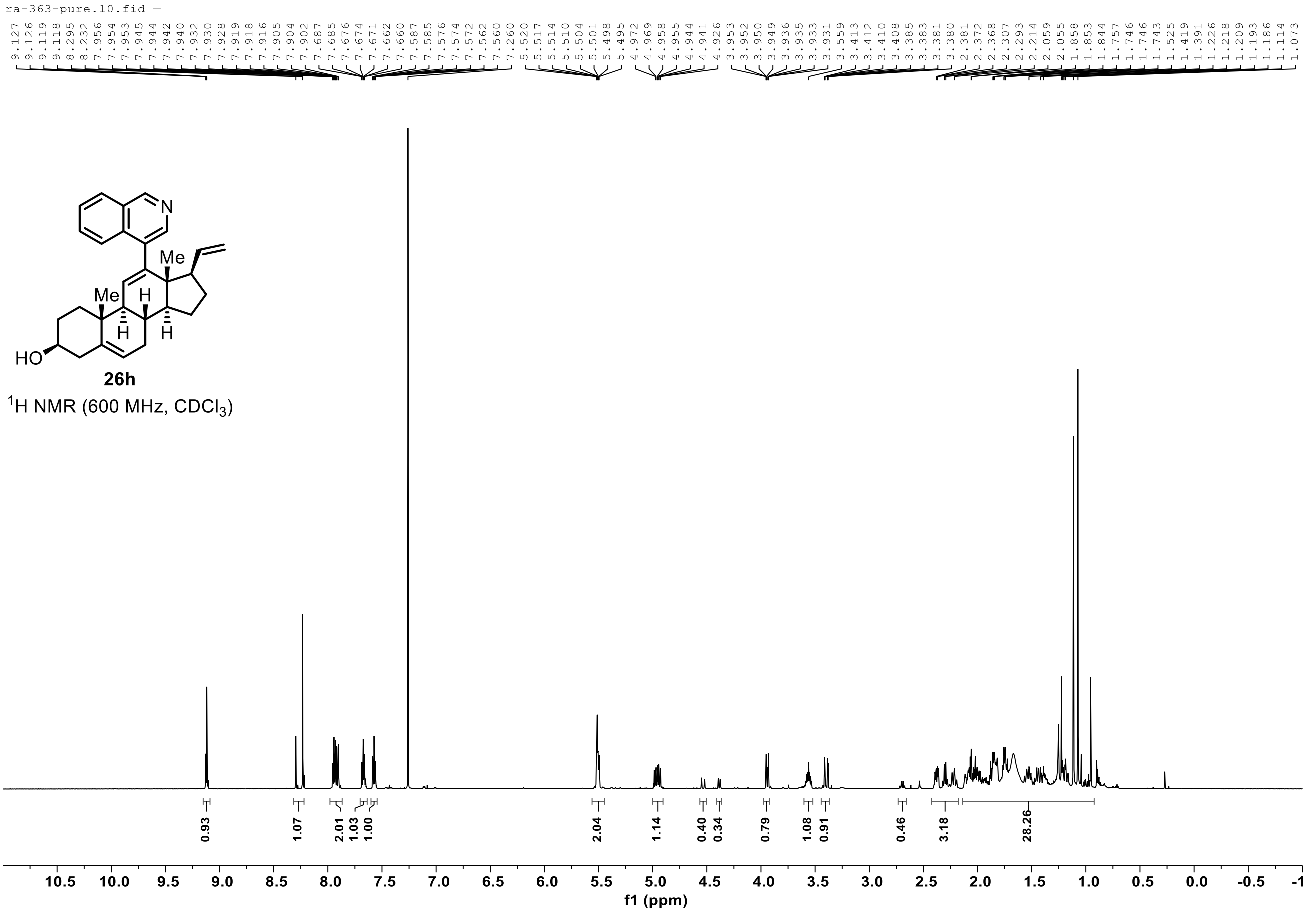

26h

^1H NMR (600 MHz, $CDCl_3$)

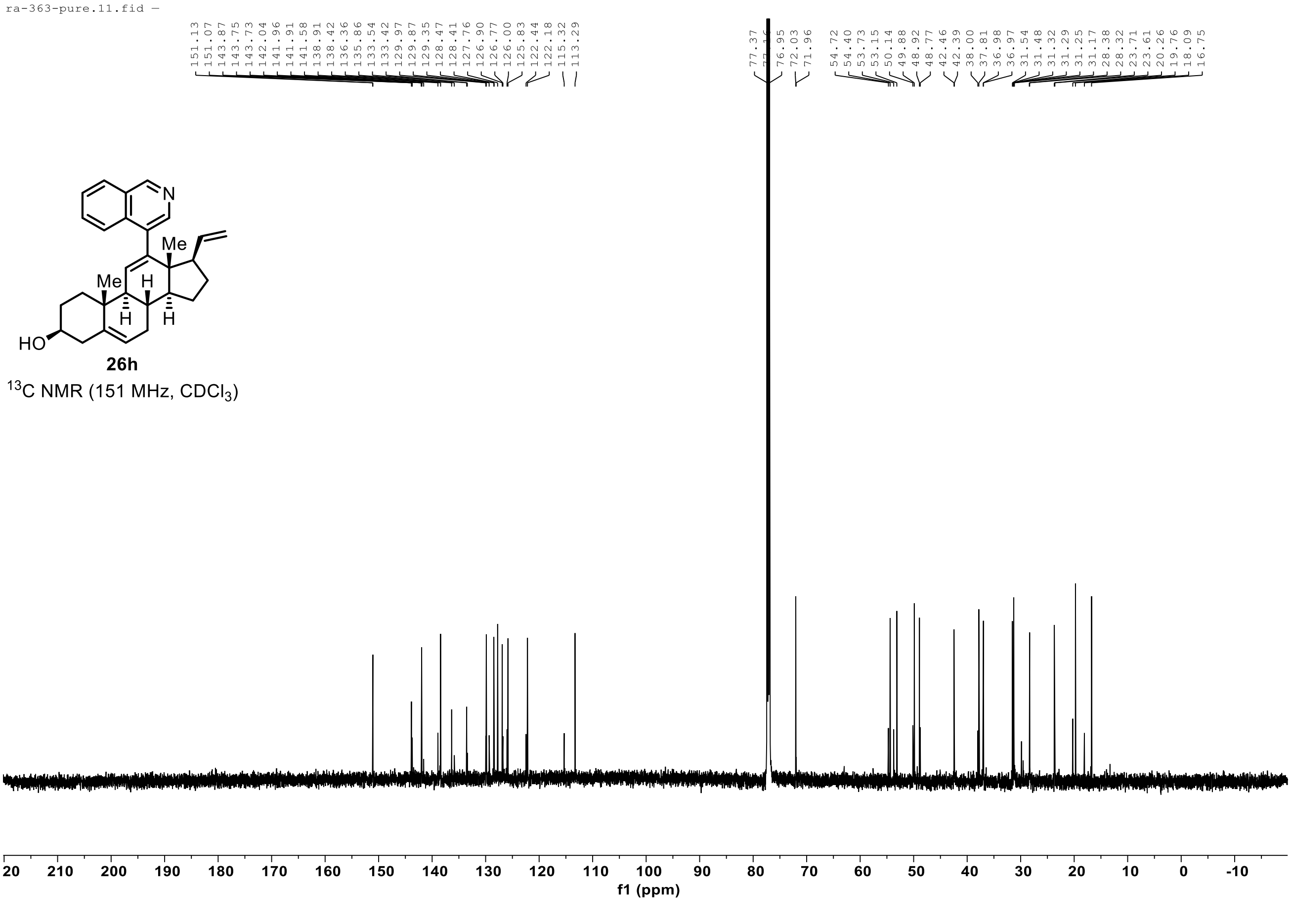

26h

^{13}C NMR (151 MHz, $CDCl_3$)

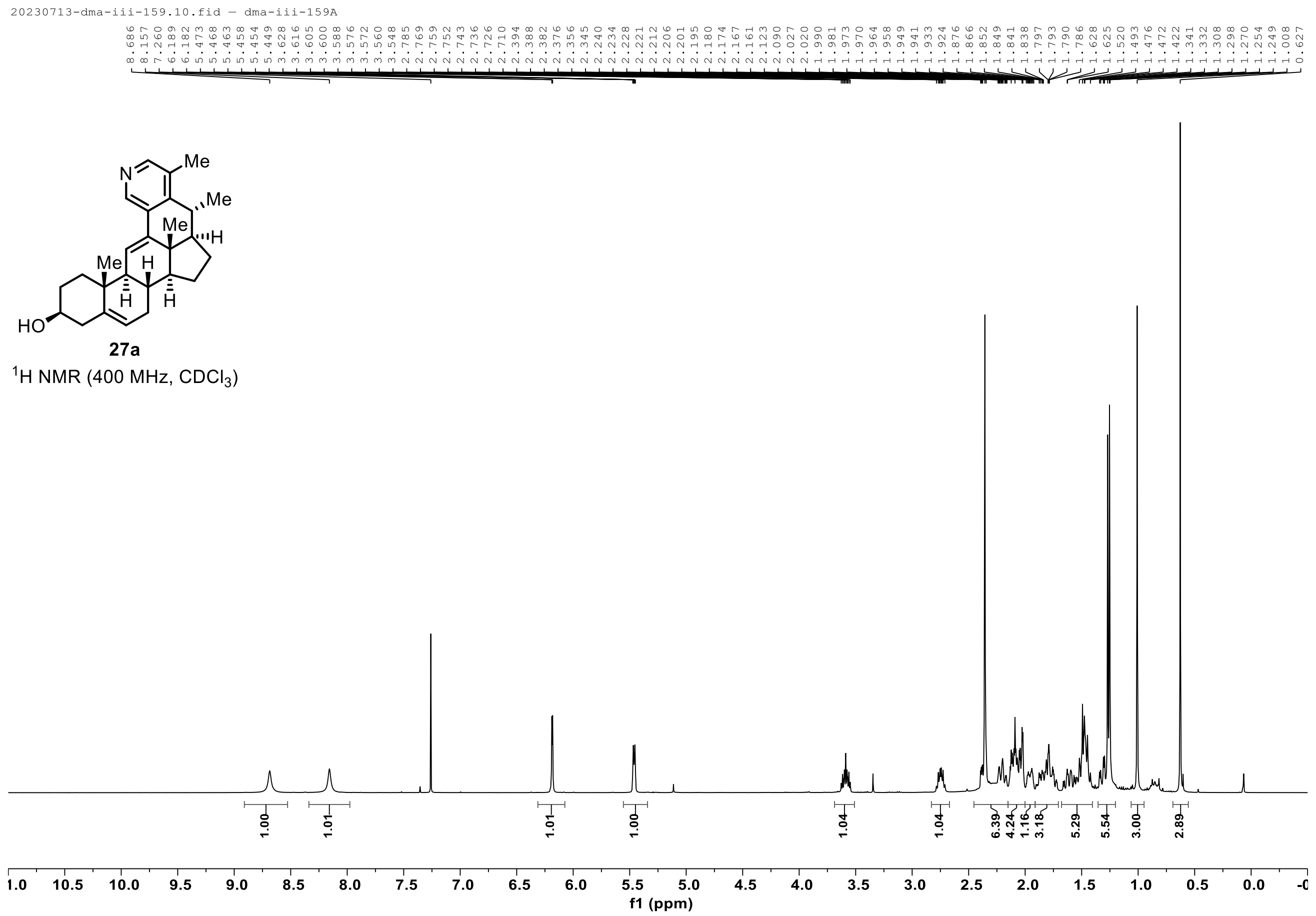

27a

^{1}H NMR (400 MHz, $CDCl_3$)

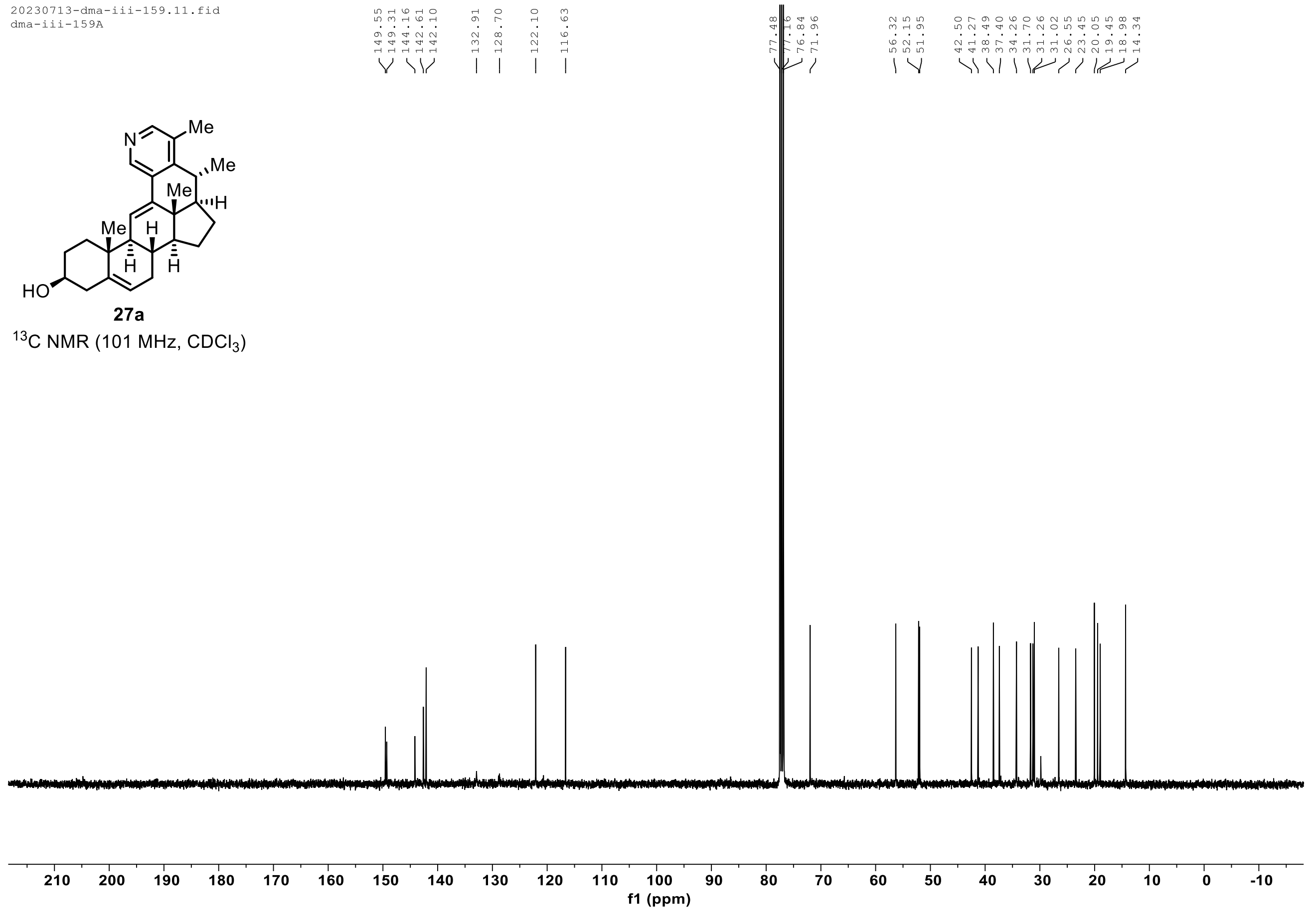

27a

^{13}C NMR (101 MHz, $CDCl_3$)

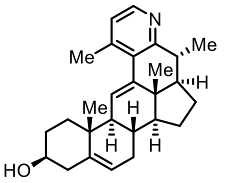

27b

^{1}H NMR (400 MHz, $CDCl_3$)

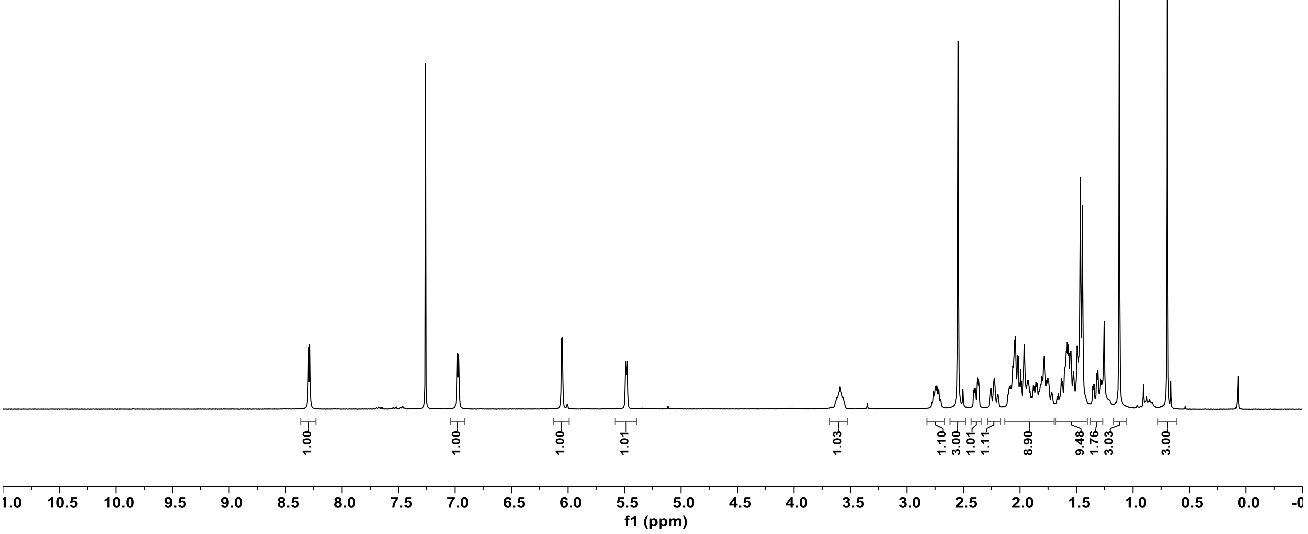

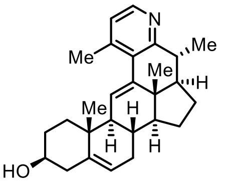

27b

^{13}C NMR (151 MHz, $CDCl_3$)

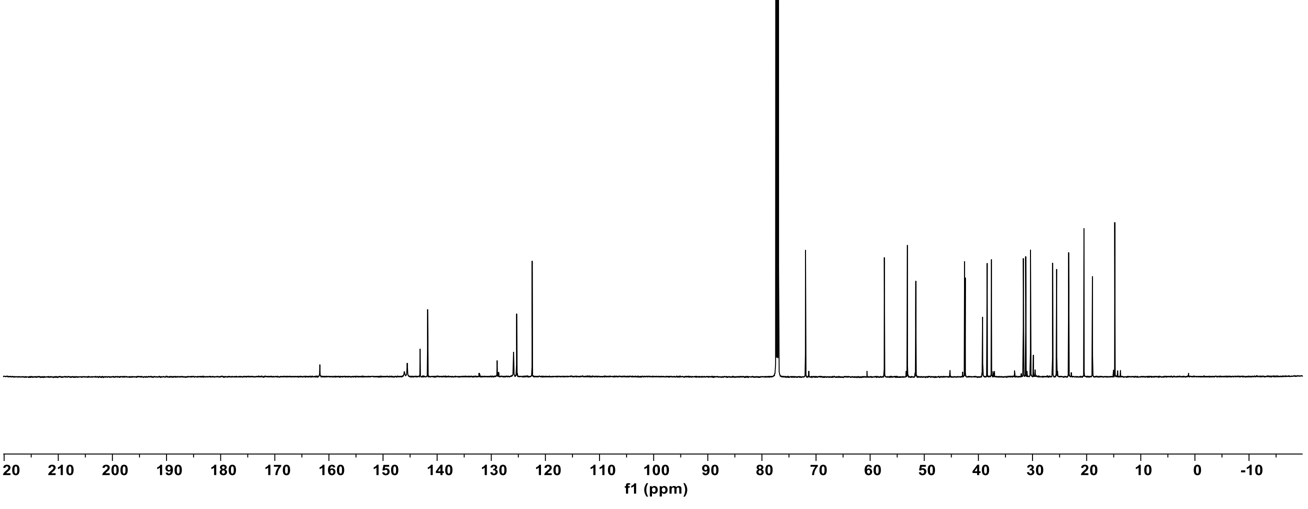

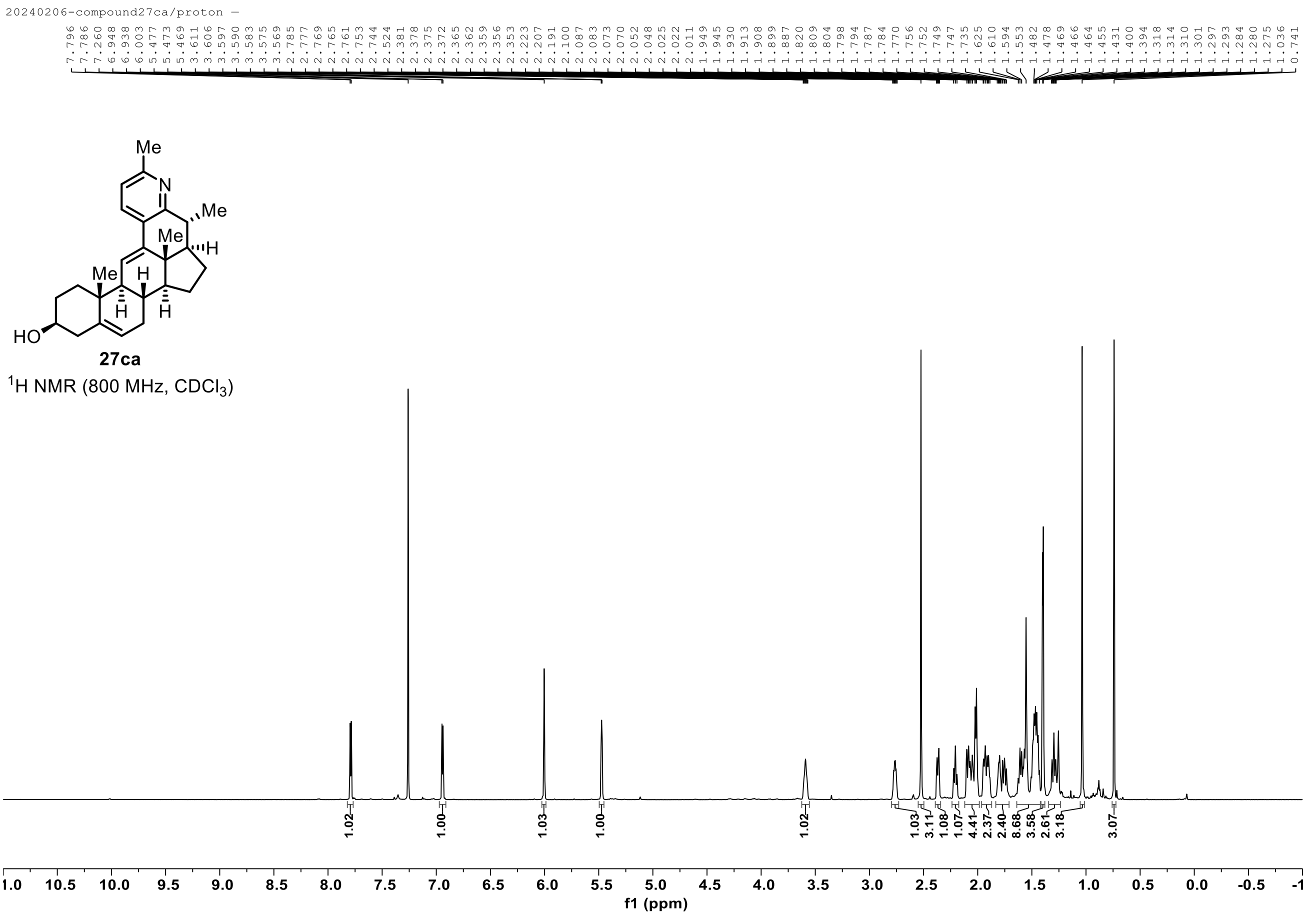
20240206-compound27ca/proton
7.796 7.786 7.260 6.948 6.938 6.003 5.477 5.473 5.469 3.611 3.606 3.597 3.590 3.583 3.575 3.569 2.785 2.777 2.769 2.765 2.761 2.753 2.744 2.524 2.381 2.378 2.375 2.372 2.365 2.362 2.359 2.356 2.353 2.223 2.207 2.191 2.100 2.087 2.083 2.073 2.070 2.052 2.048 2.025 2.022 2.011 1.949 1.945 1.930 1.913 1.908 1.899 1.887 1.820 1.809 1.804 1.798 1.794 1.787 1.784 1.770 1.756 1.752 1.749 1.747 1.735 1.625 1.610 1.594 1.553 1.482 1.478 1.469 1.466 1.464 1.455 1.431 1.400 1.394 1.318 1.314 1.310 1.301 1.297 1.293 1.284 1.280 1.275 1.036 0.741
Me
N
Me
Me
H
Me
H
H
H
HO
27ca
1H NMR (800 MHz, CDCl3)
1.02 1.00 1.03 1.00 1.02 1.03 3.11 1.08 1.07 4.41 2.37 2.40 8.68 3.58 2.61 3.18 3.07
f1 (ppm)

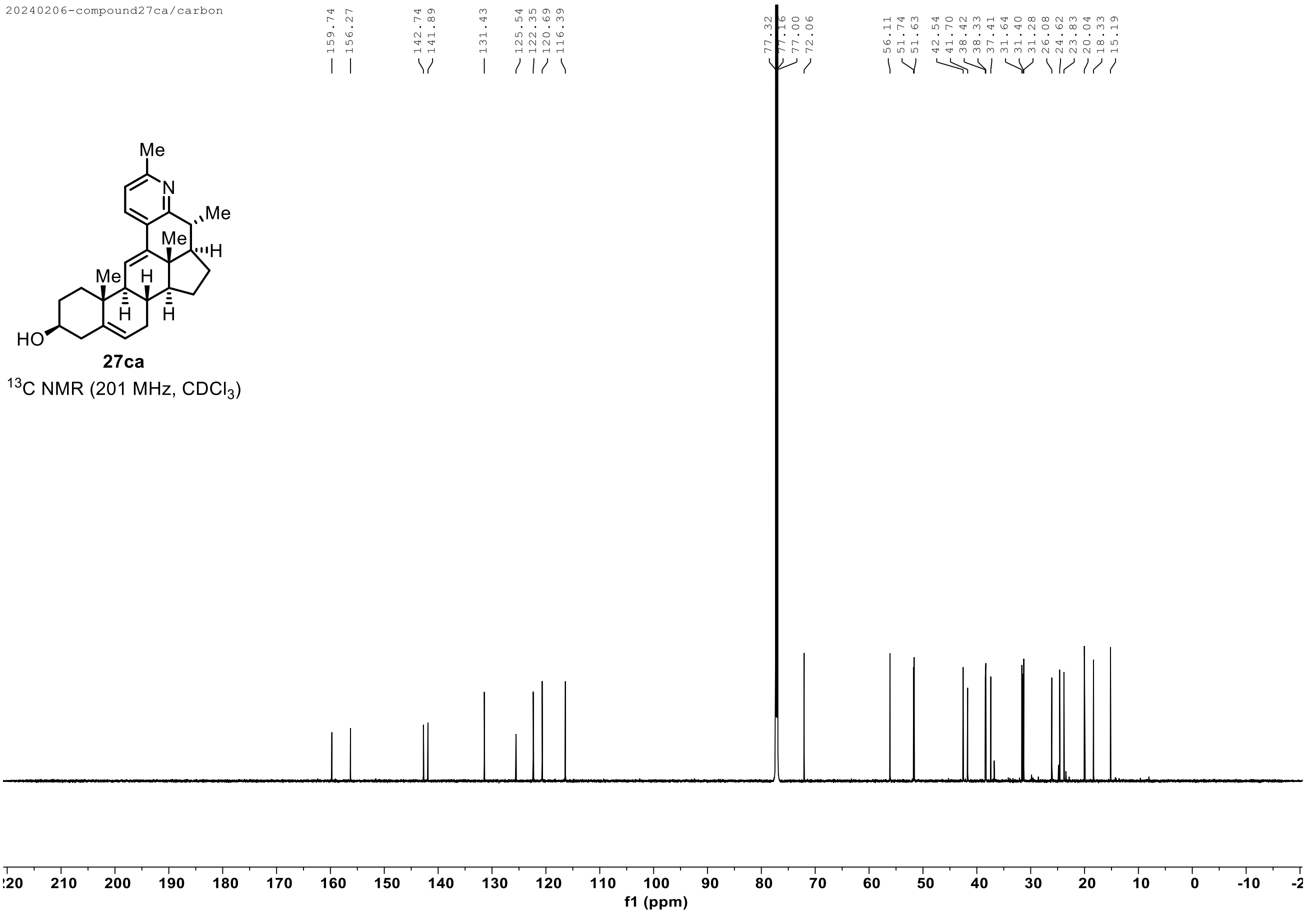
20240206-compound27ca/carbon
159.74 156.27 142.74 141.89 131.43 125.54 122.35 120.69 116.39 77.32 77.16 77.00 72.06 56.11 51.74 51.63 42.54 41.70 38.42 38.33 37.41 31.64 31.40 31.28 26.08 24.62 23.83 20.04 18.33 15.19
Me
N
Me
Me
H
Me
H
H
H
HO
27ca
13C NMR (201 MHz, CDCl3)
f1 (ppm)

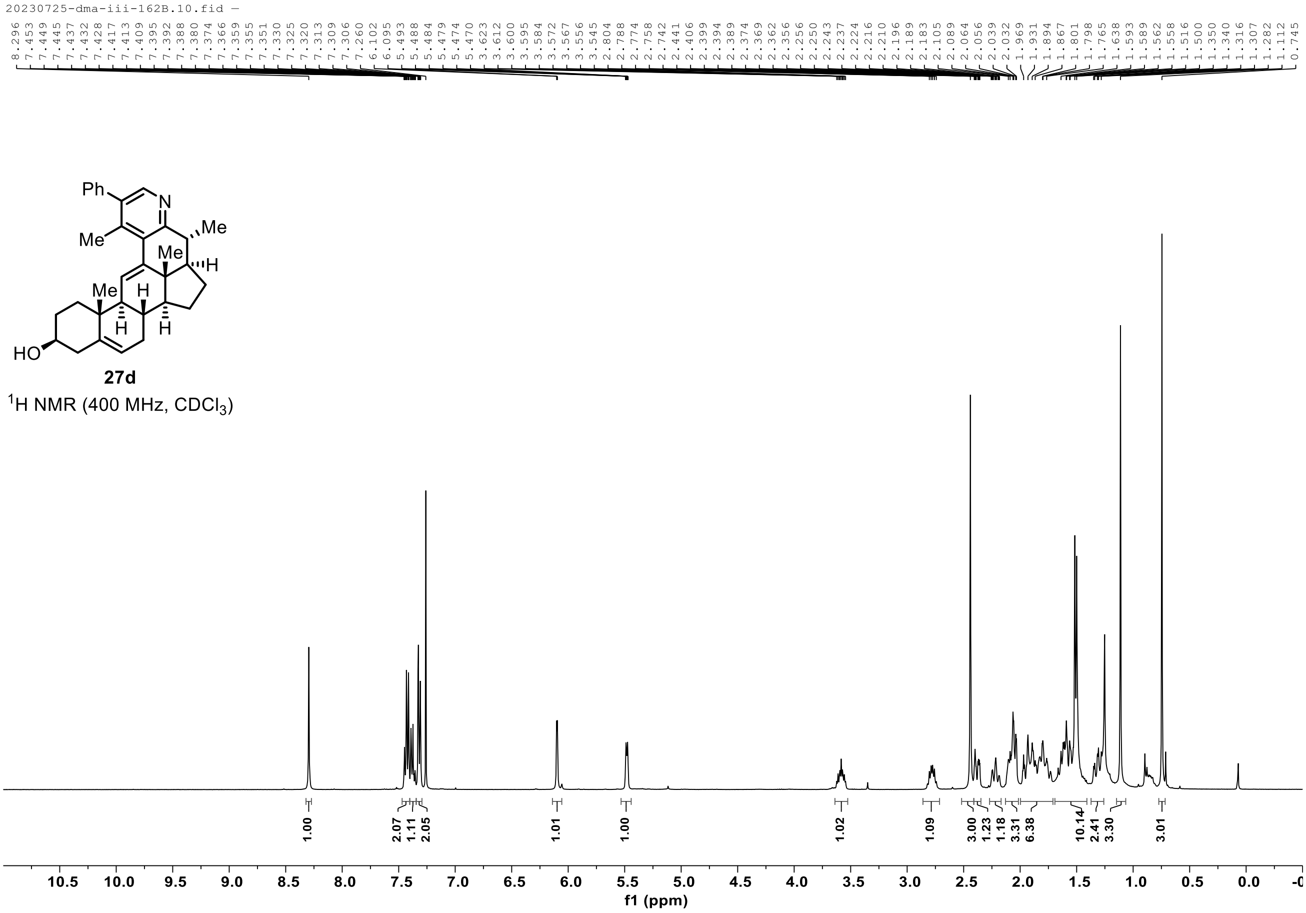

27d

^{1}H NMR (400 MHz, $CDCl_3$)

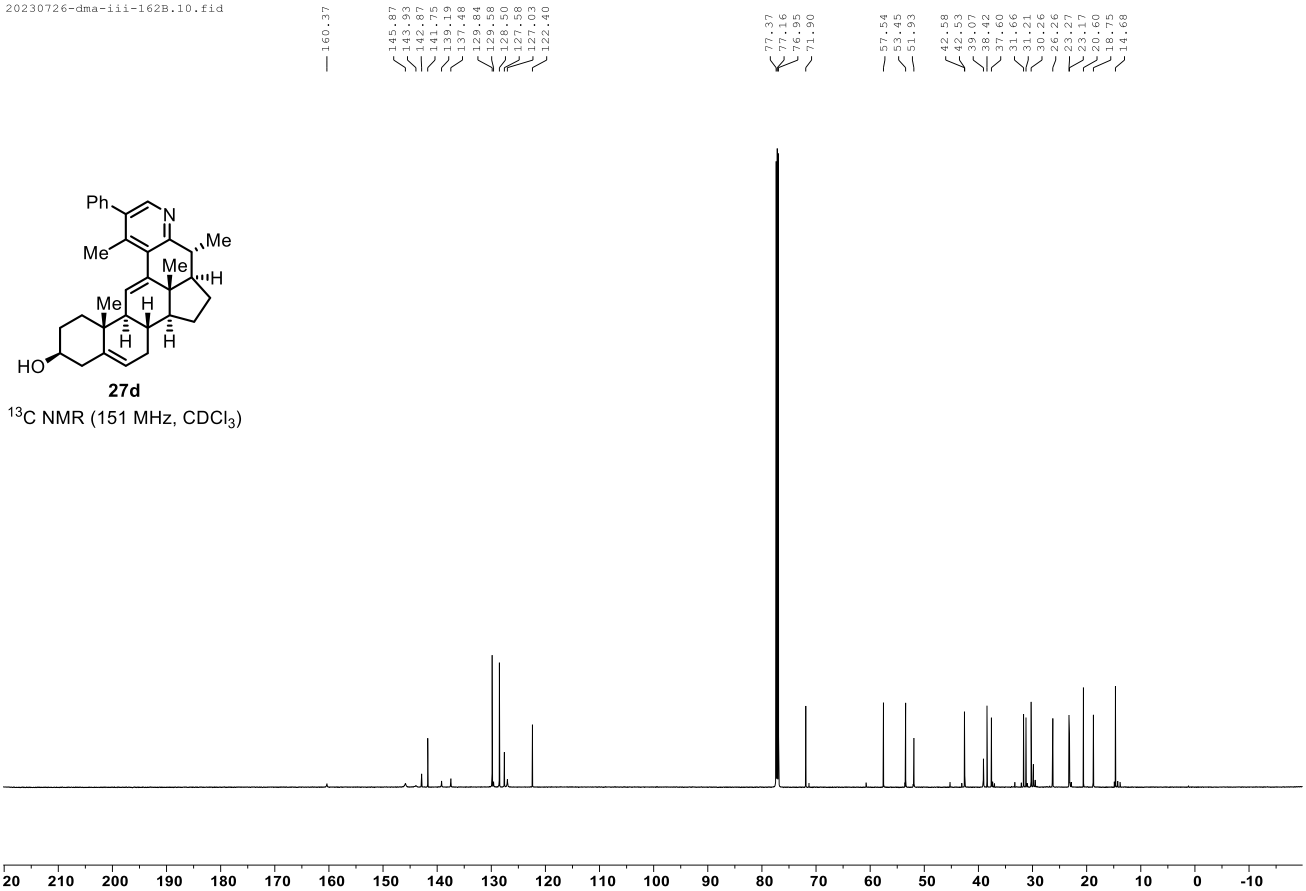

27d

^{13}C NMR (151 MHz, $CDCl_3$)

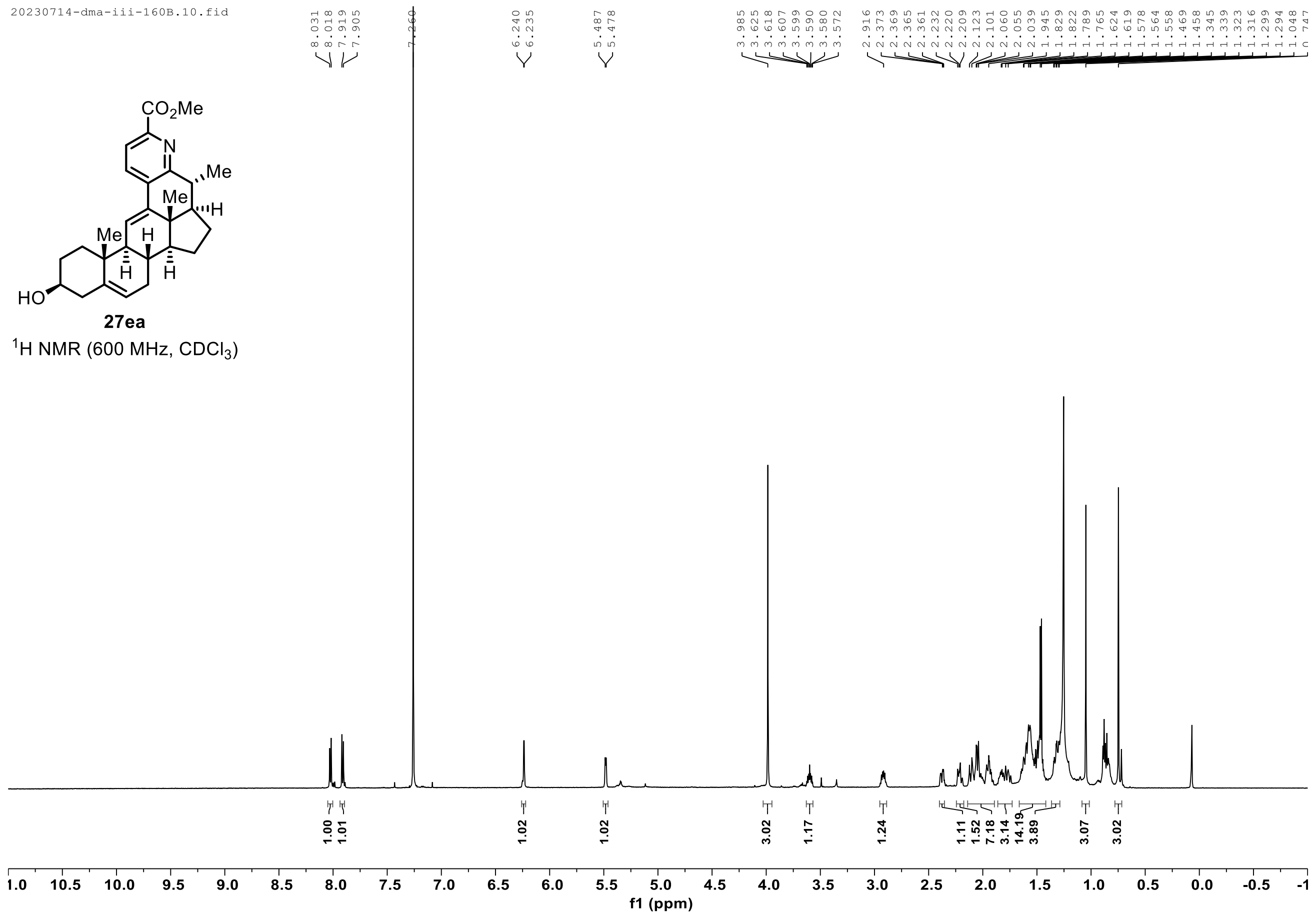
20230714-dma-iii-160B.10.fid
8.031 8.018 7.919 7.905 7.260 6.240 6.235 5.487 5.478 3.985 3.625 3.618 3.607 3.599 3.590 3.580 3.572 2.916 2.373 2.369 2.365 2.361 2.232 2.220 2.209 2.123 2.101 2.060 2.055 2.039 1.945 1.829 1.822 1.789 1.765 1.624 1.619 1.578 1.564 1.558 1.469 1.458 1.345 1.339 1.323 1.316 1.299 1.294 1.048 0.747
CO2Me
N
Me
Me
H
Me
H
H
H
HO
27ea
1H NMR (600 MHz, CDCl3)
1.00 1.01 1.02 1.02 3.02 1.17 1.24 1.11 1.52 7.18 3.14 14.19 3.89 3.07 3.02
f1 (ppm)
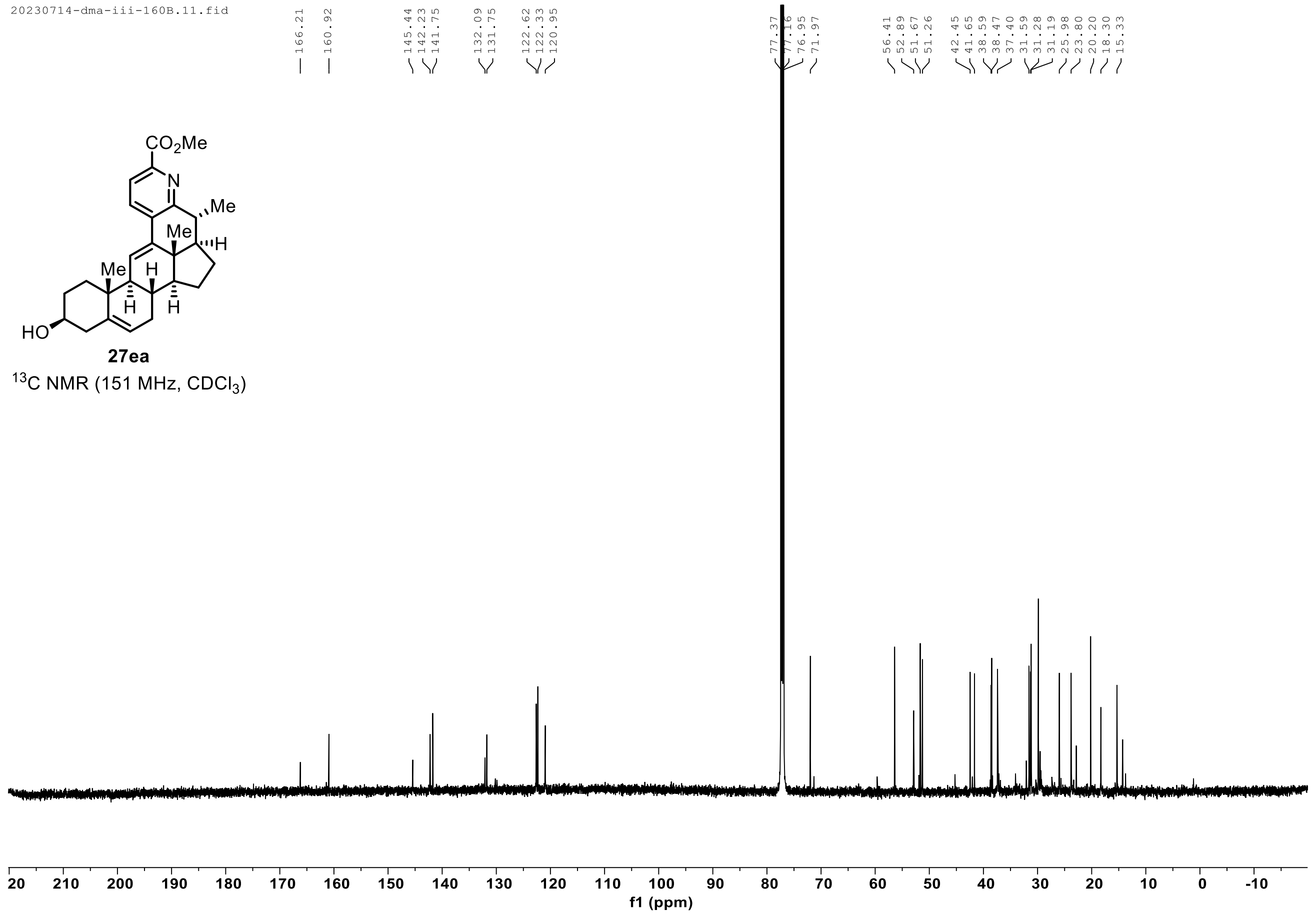
20230714-dma-iii-160B.11.fid
166.21 160.92 145.44 142.23 141.75 132.09 131.75 122.62 122.33 120.95 77.37 77.16 76.95 71.97 56.41 52.89 51.67 51.26 42.45 41.65 38.59 38.47 37.40 31.59 31.28 31.19 25.98 23.80 20.20 18.30 15.33
CO2Me
N
Me
Me
H
Me
H
H
H
HO
27ea
13C NMR (151 MHz, CDCl3)
f1 (ppm)

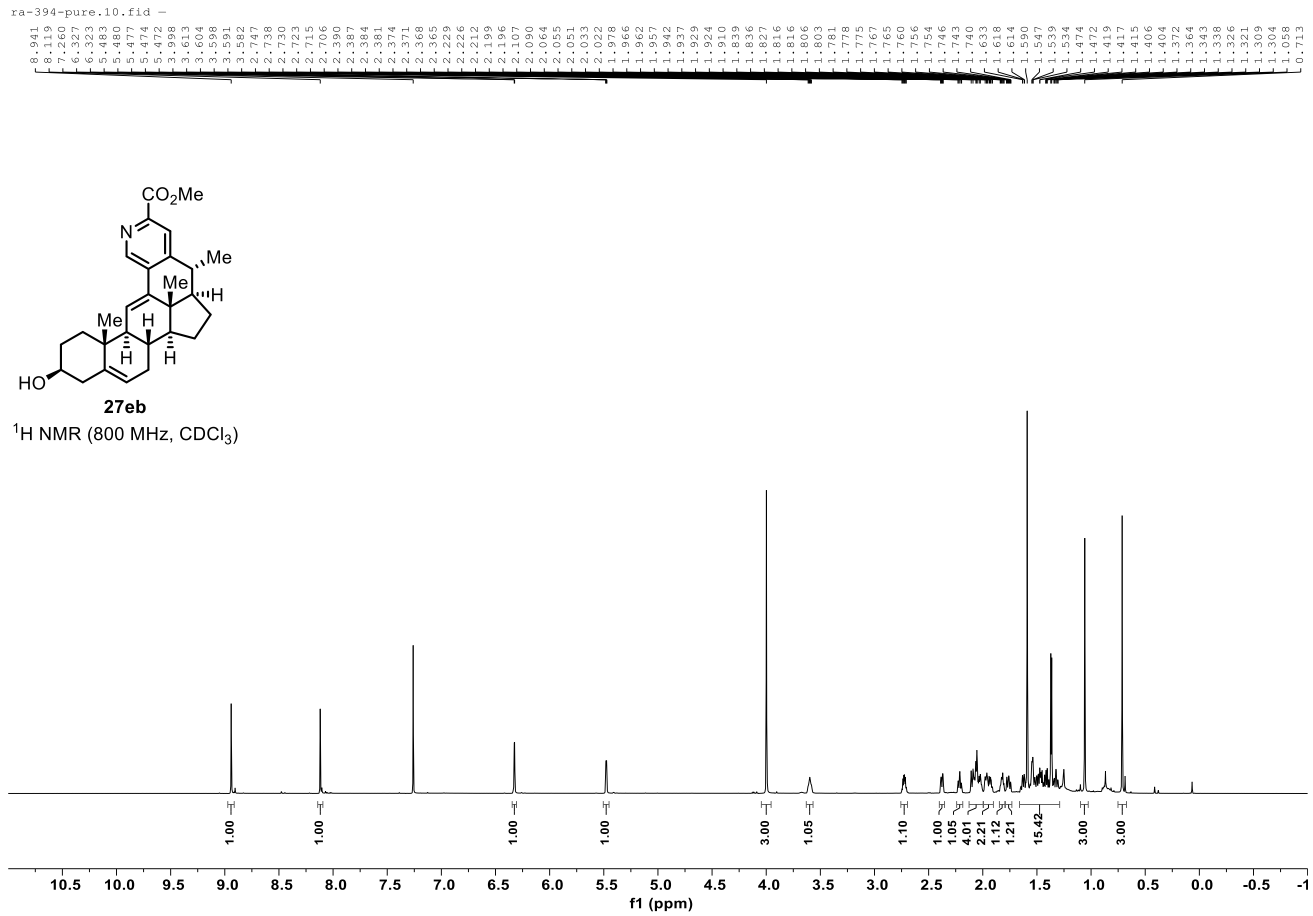
ra-394-pure.10.fid
8.941 8.119 7.260 6.327 6.323 5.483 5.480 5.477 5.474 5.472 3.998 3.613 3.604 3.598 3.591 3.582 2.747 2.738 2.730 2.723 2.715 2.706 2.390 2.387 2.384 2.381 2.374 2.371 2.368 2.365 2.229 2.226 2.212 2.199 2.196 2.107 2.090 2.064 2.055 2.051 2.033 2.022 1.978 1.966 1.962 1.957 1.942 1.937 1.929 1.924 1.910 1.839 1.836 1.827 1.816 1.816 1.806 1.803 1.781 1.778 1.775 1.767 1.765 1.760 1.756 1.754 1.746 1.743 1.740 1.633 1.618 1.614 1.590 1.547 1.539 1.534 1.474 1.472 1.419 1.417 1.415 1.406 1.404 1.372 1.364 1.343 1.338 1.326 1.321 1.309 1.304 1.058 0.713
CO2Me
N
Me
Me
H
Me
H
H
H
HO
27eb
1H NMR (800 MHz, CDCl3)
1.00
1.00
1.00
1.00
3.00
1.05
1.10
1.00
1.05
4.01
2.21
1.12
1.21
15.42
3.00
3.00
10.5 10.0 9.5 9.0 8.5 8.0 7.5 7.0 6.5 6.0 5.5 5.0 4.5 4.0 3.5 3.0 2.5 2.0 1.5 1.0 0.5 0.0 -0.5 -1
f1 (ppm)

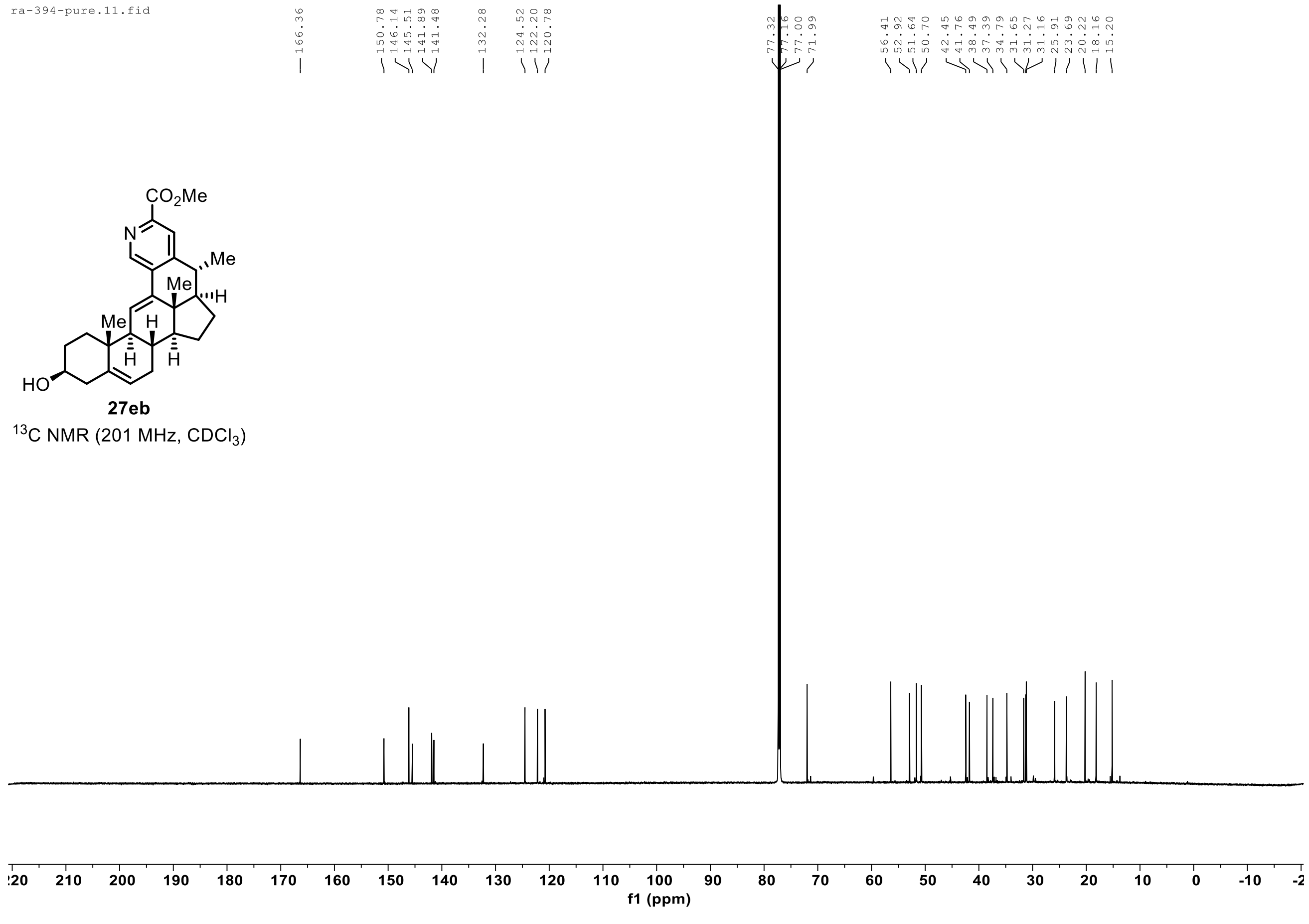
ra-394-pure.11.fid
166.36
150.78 146.14 145.51 141.89 141.48
132.28
124.52 122.20 120.78
77.32 77.16 77.00 71.99
56.41 52.92 51.64 50.70
42.45 41.76 38.49 37.39 34.79 31.65 31.27 31.16 25.91 23.69 20.22 18.16 15.20
CO2Me
N
Me
Me
H
Me
H
H
H
HO
27eb
13C NMR (201 MHz, CDCl3)
220 210 200 190 180 170 160 150 140 130 120 110 100 90 80 70 60 50 40 30 20 10 0 -10 -20
f1 (ppm)

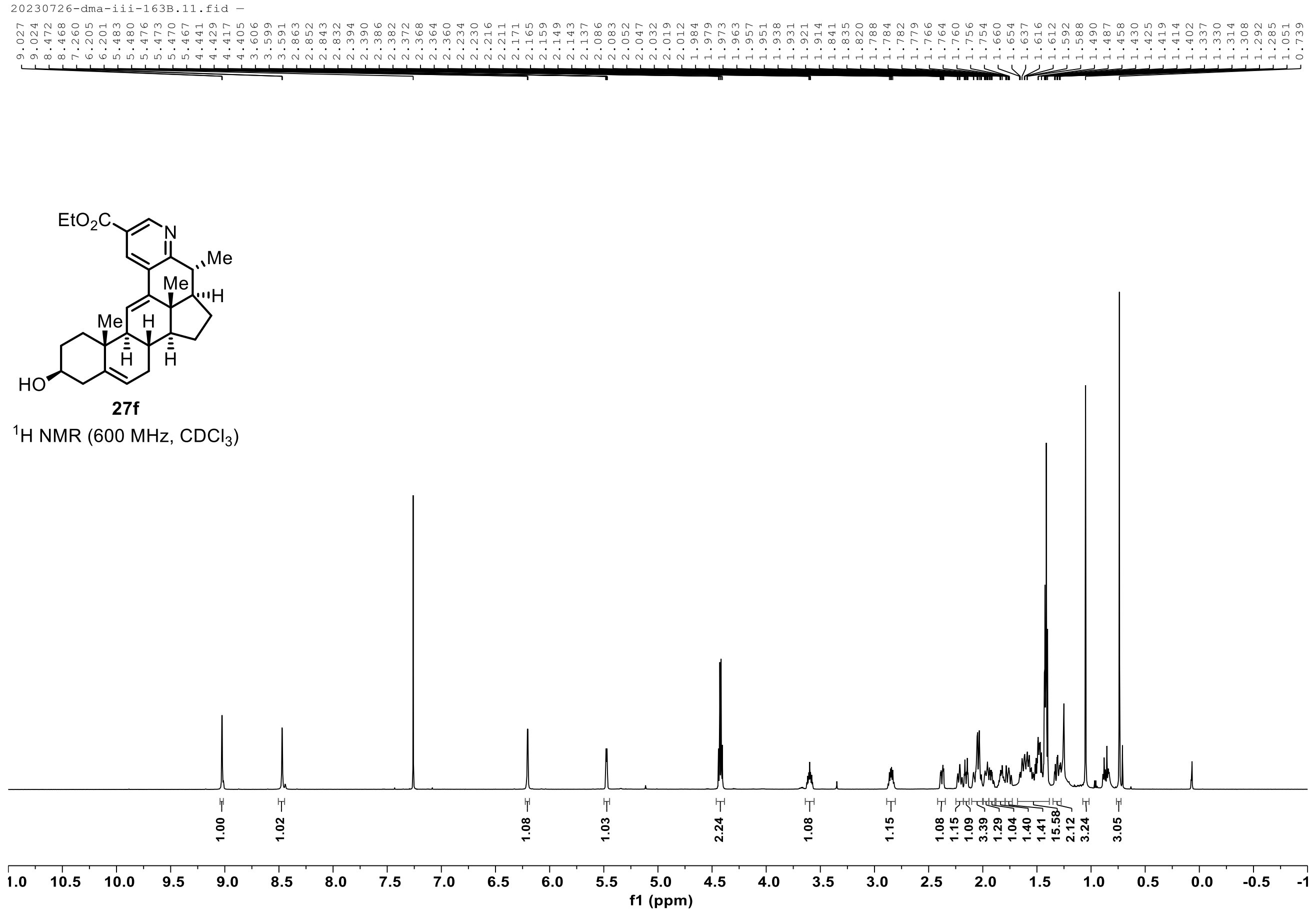
20230726-dma-iii-163B.11.fid
9.027 9.024 8.472 8.468 7.260 6.205 6.201 5.483 5.480 5.476 5.473 5.470 5.467 4.441 4.429 4.417 4.405 3.606 3.599 3.591 2.863 2.852 2.843 2.832 2.394 2.390 2.386 2.382 2.372 2.368 2.364 2.360 2.234 2.230 2.216 2.211 2.171 2.165 2.159 2.149 2.143 2.137 2.086 2.083 2.052 2.047 2.032 2.019 2.012 1.984 1.979 1.973 1.963 1.957 1.951 1.938 1.931 1.921 1.914 1.841 1.835 1.820 1.788 1.784 1.782 1.779 1.766 1.764 1.760 1.756 1.754 1.660 1.654 1.637 1.616 1.612 1.592 1.588 1.490 1.487 1.458 1.430 1.425 1.419 1.414 1.402 1.337 1.330 1.314 1.308 1.292 1.285 1.051 0.739
EtO2C
N
Me
Me
H
Me
H
H
H
HO
27f
1H NMR (600 MHz, CDCl3)
1.00 1.02 1.08 1.03 2.24 1.08 1.15 1.08 1.15 1.09 3.39 1.29 1.04 1.40 1.41 15.58 2.12 3.24 3.05
1.0 10.5 10.0 9.5 9.0 8.5 8.0 7.5 7.0 6.5 6.0 5.5 5.0 4.5 4.0 3.5 3.0 2.5 2.0 1.5 1.0 0.5 0.0 -0.5 -1
f1 (ppm)

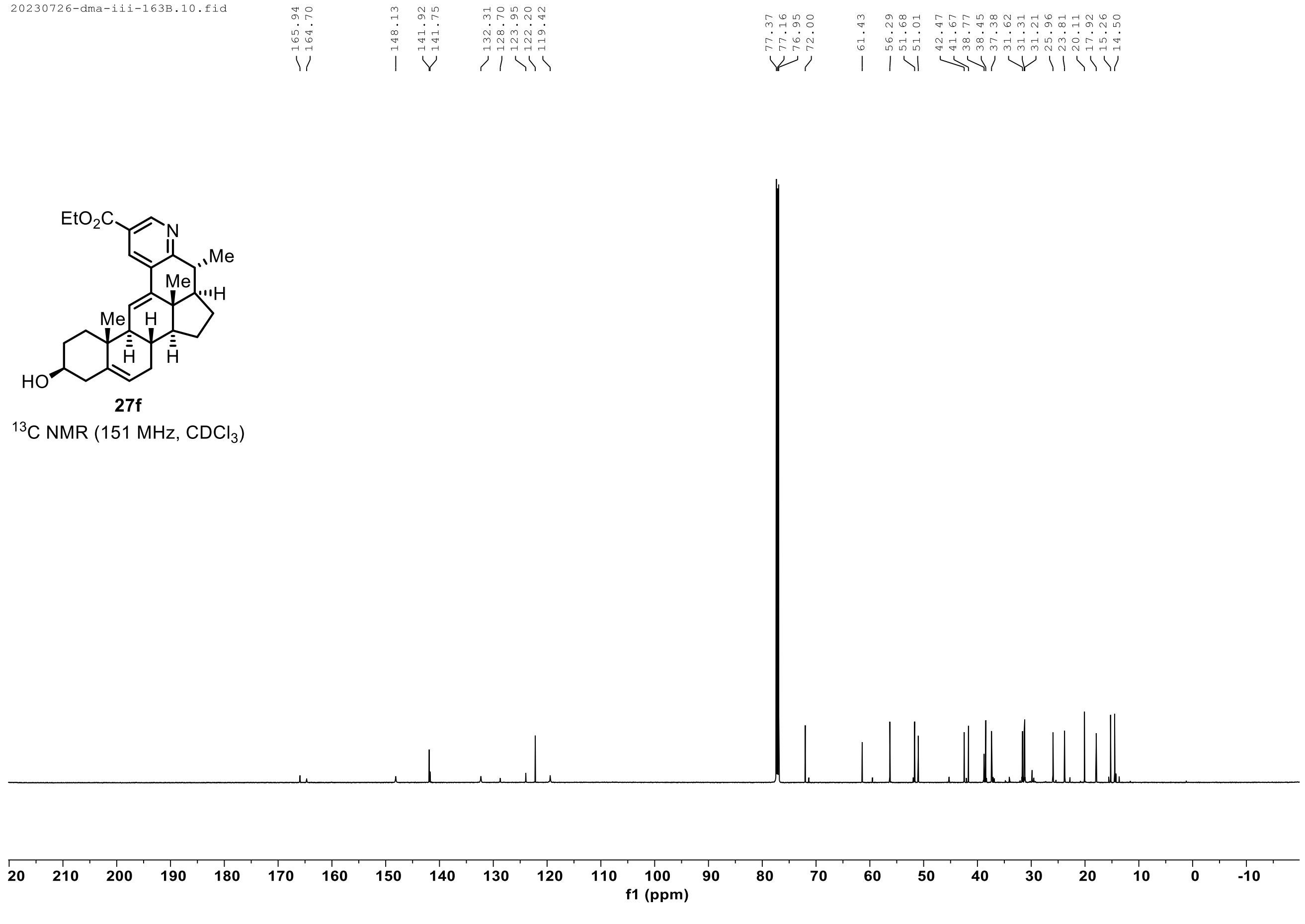
20230726-dma-iii-163B.10.fid
165.94 164.70 148.13 141.92 141.75 132.31 128.70 123.95 122.20 119.42 77.37 77.16 76.95 72.00 61.43 56.29 51.68 51.01 42.47 41.67 38.77 38.45 37.38 31.62 31.31 31.21 25.96 23.81 20.11 17.92 15.26 14.50
EtO2C
N
Me
Me
H
Me
H
H
H
HO
27f
13C NMR (151 MHz, CDCl3)
20 210 200 190 180 170 160 150 140 130 120 110 100 90 80 70 60 50 40 30 20 10 0 -10
f1 (ppm)

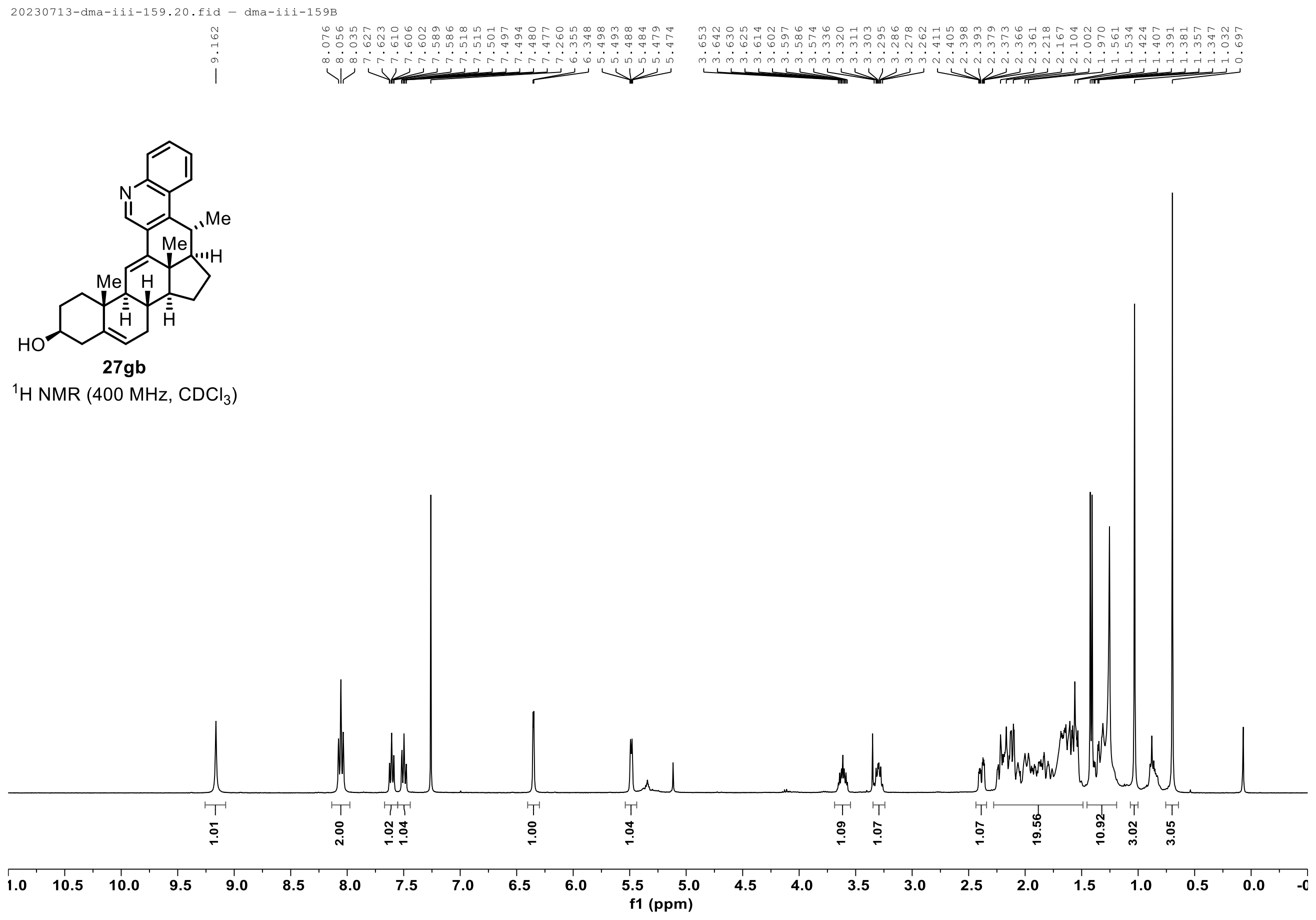
20230713-dma-iii-159.20.fid — dma-iii-159B
27gb
¹H NMR (400 MHz, CDCl₃)
f1 (ppm)

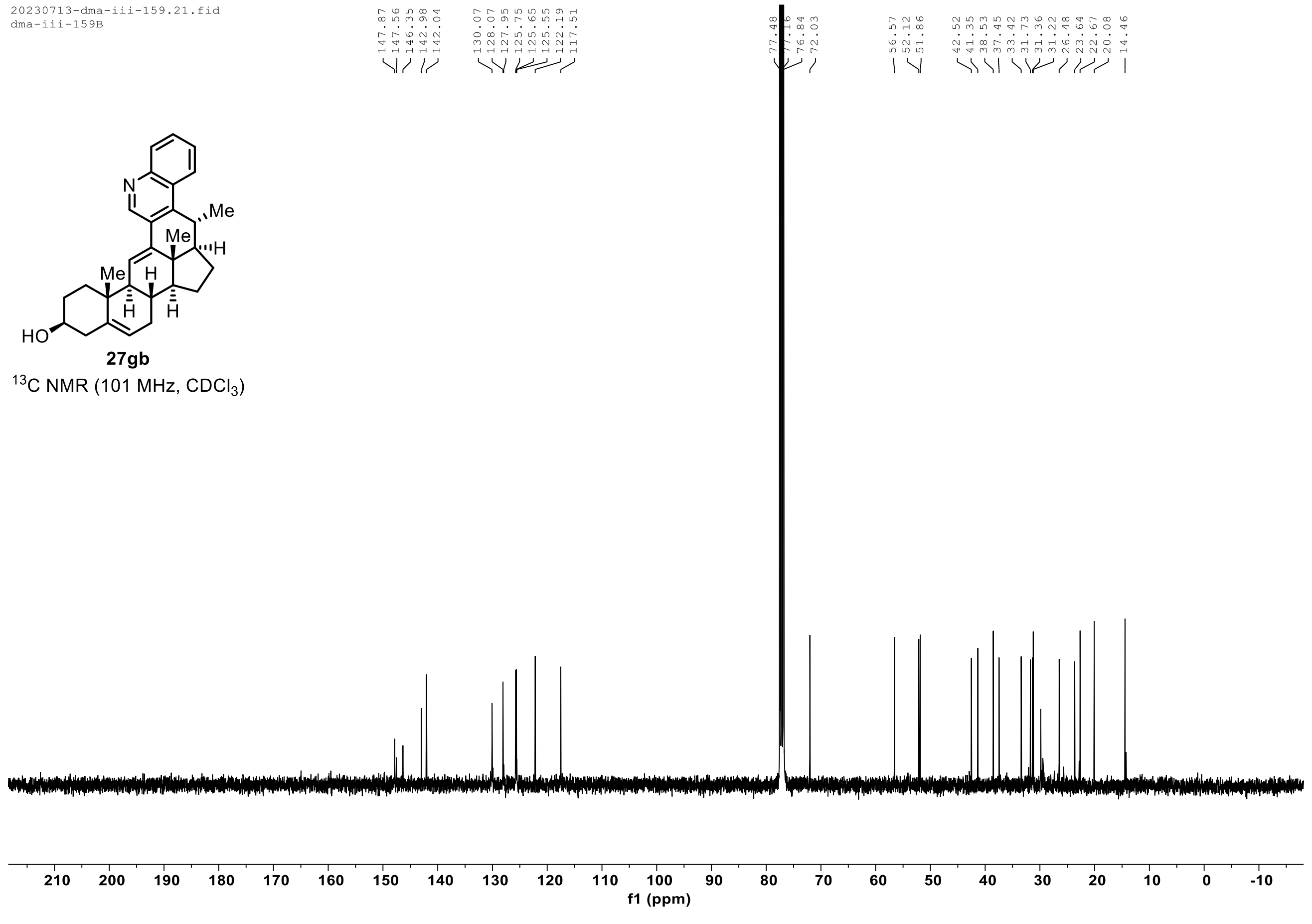
20230713-dma-iii-159.21.fid
dma-iii-159B
27gb
¹³C NMR (101 MHz, CDCl₃)
f1 (ppm)

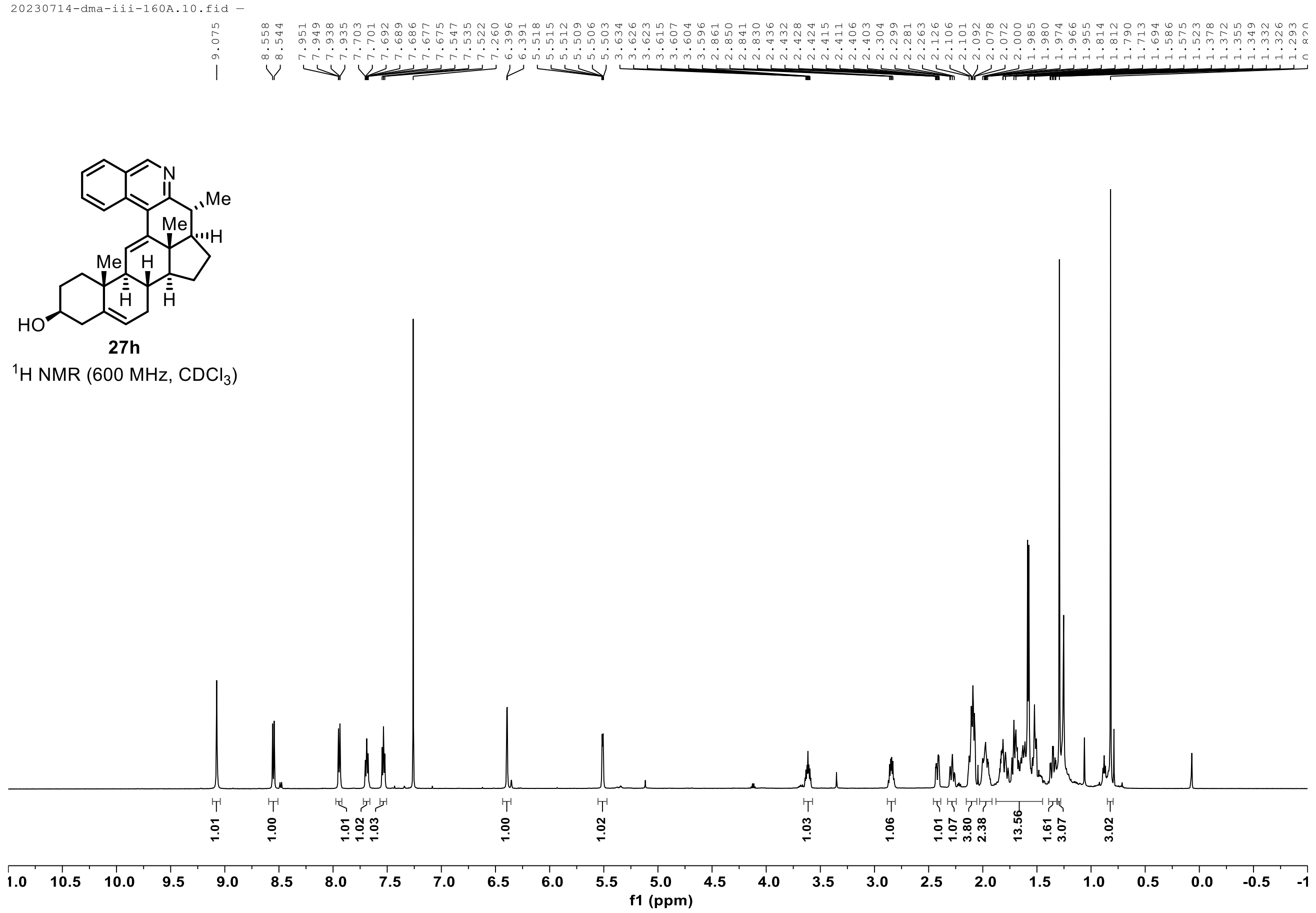
20230714-dma-iii-160A.10.fid
9.075
8.558
8.544
7.951
7.949
7.938
7.935
7.703
7.701
7.692
7.689
7.686
7.677
7.675
7.547
7.535
7.522
7.260
6.396
6.391
5.518
5.515
5.512
5.509
5.506
5.503
3.634
3.626
3.623
3.615
3.607
3.604
3.596
2.861
2.850
2.841
2.830
2.436
2.432
2.428
2.424
2.415
2.411
2.406
2.403
2.304
2.299
2.281
2.263
2.126
2.106
2.101
2.092
2.078
2.072
2.000
1.985
1.980
1.974
1.966
1.955
1.814
1.812
1.790
1.713
1.694
1.586
1.575
1.523
1.378
1.372
1.355
1.349
1.332
1.326
1.293
N
Me
Me
H
Me
H
H
H
HO
27h
^{1}H NMR (600 MHz, CDCl$_3$)
1.01
1.00
1.01
1.02
1.03
1.00
1.02
1.03
1.06
1.01
1.07
3.80
2.38
13.56
1.61
3.07
3.02
11.0 10.5 10.0 9.5 9.0 8.5 8.0 7.5 7.0 6.5 6.0 5.5 5.0 4.5 4.0 3.5 3.0 2.5 2.0 1.5 1.0 0.5 0.0 -0.5 -1
f1 (ppm)

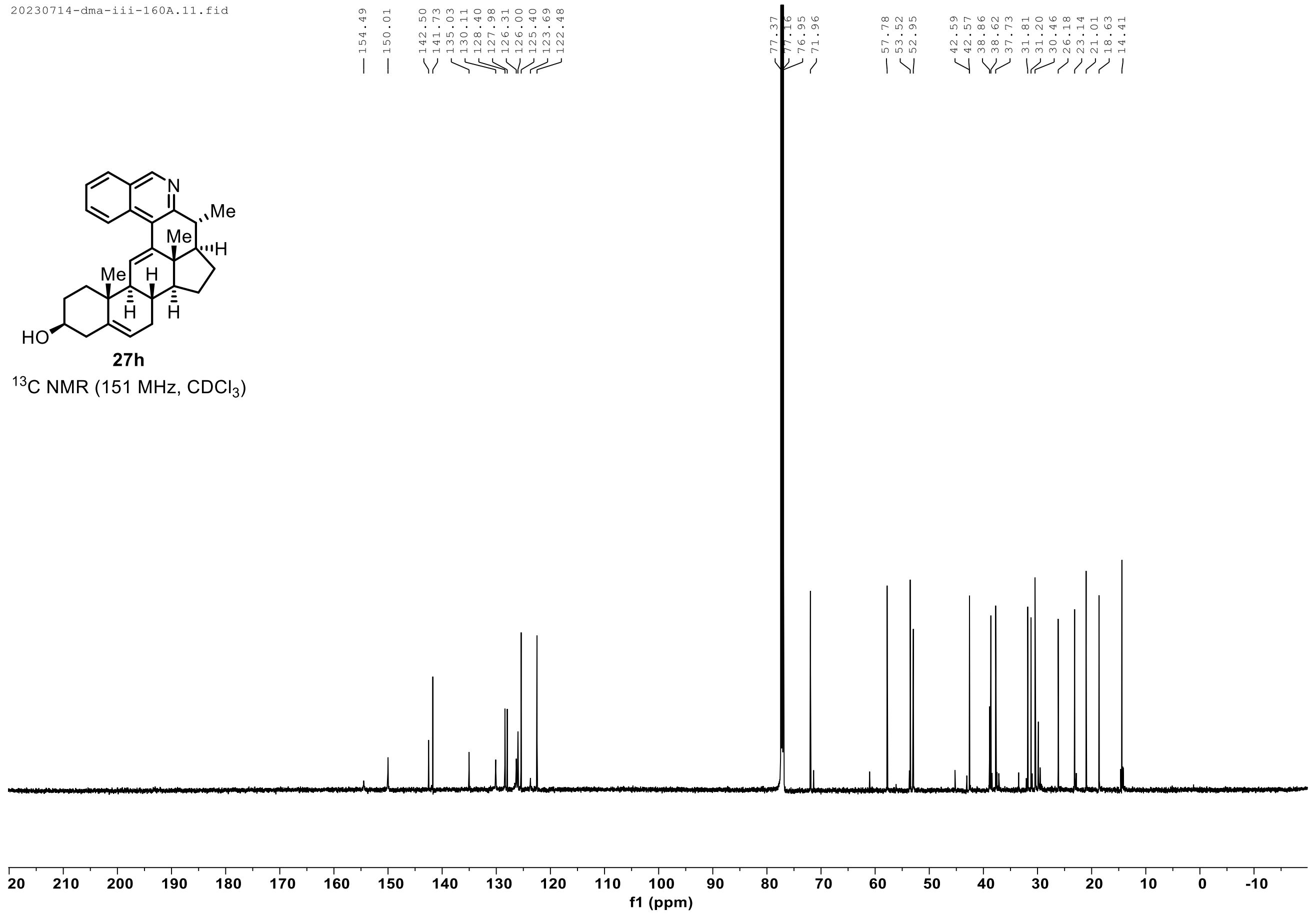
20230714-dma-iii-160A.11.fid
154.49
150.01
142.50
141.73
135.03
130.11
128.40
127.98
126.31
126.00
125.40
123.69
122.48
77.37
77.16
76.95
71.96
57.78
53.52
52.95
42.59
42.57
38.86
38.62
37.73
31.81
31.20
30.46
26.18
23.14
21.01
18.63
14.41
N
Me
Me
H
Me
H
H
H
HO
27h
^{13}C NMR (151 MHz, CDCl$_3$)
220 210 200 190 180 170 160 150 140 130 120 110 100 90 80 70 60 50 40 30 20 10 0 -10
f1 (ppm)
